# Outcomes Following Adherence to a Randomized Stroke Trial Protocol

**DOI:** 10.1001/jamanetworkopen.2023.49730

**Published:** 2024-01-02

**Authors:** Rolf Ankerlund Blauenfeldt, Claus Z. Simonsen, Jan B. Valentin, Søren P. Johnsen, Niels Hjort, Grethe Andersen

**Affiliations:** 1 Department of Neurology, Aarhus University Hospital, Aarhus, Denmark; 2 Department of Clinical Medicine, Aarhus University, Aarhus, Denmark; 3 Danish Center for Health Services Research, Department of Clinical Medicine, Aalborg University, Aalborg, Denmark

## Abstract

This secondary analysis of a randomized clinical trial assesses whether compliance with the study protocol is associated with a better functional outcome even among participants in the sham-control group.

## Introduction

The placebo effect is often associated with a drug and describes a well-established psychobiological phenomenon that is mediated by expectancies of future outcomes.^1^ The same, but sometimes stronger, effect can be observed when a physical intervention or device is tested.^2^ Pragmatic trials are increasingly being used, and open-label, blinded, end-point trials represent feasible, cost-effective alternatives to double-blinded and placebo- or sham-controlled randomized trials.^3^ Open-label studies may be relevant alternatives when the placebo or sham effect is small and “hard” end points are used.

Compliance with protocol (Supplement 1), even when the treatment is placebo or sham, may be associated with better outcome. Here, we present an example in an acute stroke trial using a physical intervention.

## Methods

This post hoc secondary analysis of a randomized clinical trial (NCT03481777) investigating the effect of remote ischemic conditioning (RIC) in acute stroke^4^ was conducted at 4 sites in Denmark. The trial included 1500 patients with prehospital stroke symptoms for less than 4 hours who underwent prehospital randomization between March 16, 2018, and November 11, 2022. The RIC was applied in the prehospital phase and continued in the hospital. It consisted of 5 cycles, each with 5 minutes of cuff inflation and 5 minutes with no cuff pressure. The RIC device exerted a cuff pressure of at least 200 mm Hg on 1 upper extremity; the sham device, 20 mm Hg. This study followed the CONSORT reporting guideline and was approved by the Danish regional research ethics committees and Danish Medicines Agency. Written informed consent was provided by all participants or trial guardians and next of kin.^4^ Data on compliance was stored on each RIC device and was stratified into 4 categories (<50%, 50%-79%, 80%-99%, and ≥100%). Ordinal logistic regression analysis was used to analyze the association between compliance and functional outcome after 90 days, which was measured using the modified Rankin Scale (range, 0-6, with 0 representing no symptoms; 6, death). Data were analyzed using STATA, version 17.0. A 2-sided *P* < .05 was considered statistically significant.

## Results

In total, 902 patients received a diagnosis of acute stroke, and 874 (97%) patients had available adherence data. Of them, 424 patients were allocated to RIC and 450 to sham. The mean (SD) age was 71 (13) years, 551 (63%) were male, and 323 (37%) were female. An association was found between treatment compliance of at least 80% (Table) and increased odds for improved functional outcome at 90 days for both RIC (eg, 80%-99% compliance odds ratio [OR], 3.16; 95% CI, 1.96-5.10) and sham (OR, 2.85; 95% CI, 1.67-4.87) (Figure, A), with no evidence of treatment benefit between the 2 groups with the same degree of compliance. Patients with high compliance were younger, more likely to receive acute reperfusion therapy, and had less frequent atrial fibrillation and less severe stroke (Figure, B).

**Table.** Reasons for Incomplete Adherence to RIC Protocol

Registered deviations in study treatment^a^	Participants in each compliance category, No. (%)			
	<50% (n = 163)	50%-79% (n = 146)	80%-99% (n = 303)	≥100% (n = 262)
Unknown reason for incomplete treatment	42 (25.8)	120 (82.2)	182 (60.1)	22 (8.4)
Death during treatment period or withdrawn life support	30 (18.4)	5 (3.4)	2 (0.7)	3 (1.1)
Patient or family did not want further study treatment (eg, due to pain, unable to cope with treatment)	63 (38.7)	1 (0.7)	0	4 (1.5)
Treatment discomfort or adverse effects	3 (1.8)	2 (1.4)	2 (0.7)	0 (0)
Device lost	4 (2.5)	2 (1.4)	3 (1.0)	1 (0.4)
Technical issues (eg, battery)	2 (1.2)	2 (1.4)	1 (0.3)	1 (0.4)

Abbreviation: RIC, remote ischemic conditioning.

^a^ Treatment compliance was electronically recorded on each RIC and sham device, which were returned after completed treatment. A readout was performed, and any known reason for deviation in treatment compliance was registered. In 28 patients, no device adherence data were available.

**Figure.**
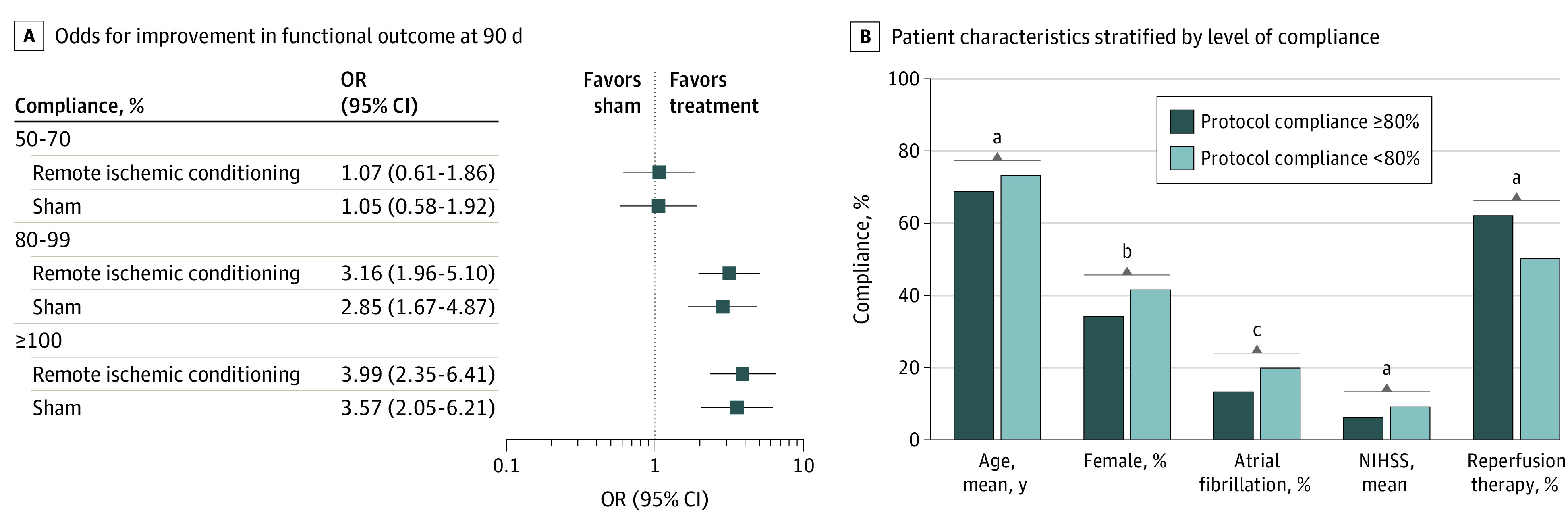
Compliance With Protocol, Patient Characteristics, and Functional Outcome A, Odds ratios (ORs) for a beneficial improvement in the modified Rankin Scale score (reference, compliance below 50%). B, Patient characteristics stratified by level of compliance with protocol. Stroke severity at admission was measured on the National Institutes of Health Stroke Scale (NIHSS; range 0-42, with higher scores indicating more severe symptoms). ^a^*P* < .001. ^b^*P* = .03. ^c^*P* = .01.

## Discussion

Treatment effect was associated with compliance for both the RIC and sham treatment in a dose-response manner. In some clinical trials, it may be impractical or unethical to implement a sham comparator, and this must be weighed against the risk of mistaking the sham effect for a beneficial therapeutic effect. Some of the observed sham effects may be explained by regression to the mean.^5^ If the current trial had not included a sham procedure, the results would have suggested a benefit of the intervention on functional outcome when compliance to treatment was good (dose-response effect). But patients with high protocol compliance may be different and prone to do better.^6^ Hence, the effect of compliance with a new treatment should be compared only for patients with the same level of compliance to sham or placebo treatment to avoid selection bias. Caution is warranted when interpreting the effect of treatment compliance on outcome in nonsham-controlled trials. The study was conducted in a predominantly White population, with no information on race and ethnicity collected, limiting the generalizability of the findings.

## Notes

Supplement 1.
Trial Protocol

Supplement 2.
Data Sharing Statement
