# Supplementary material for: Outcomes Following Adherence to a Randomized Stroke Trial Protocol
Source: JAMA Netw Open. 2024 Jan 2;7(1):e2349730. doi: 10.1001/jamanetworkopen.2023.49730 (PMC10762582; doi:10.1001/jamanetworkopen.2023.49730)
Supplement: Supplement 1. — Trial Protocol [file jamanetwopen-e2349730-s001.pdf]

Supplement

1. Study Flowchart
2. CONSORT checklist
3. Study protocols and changes
4. Statistical analysis plan (SAP) and changes

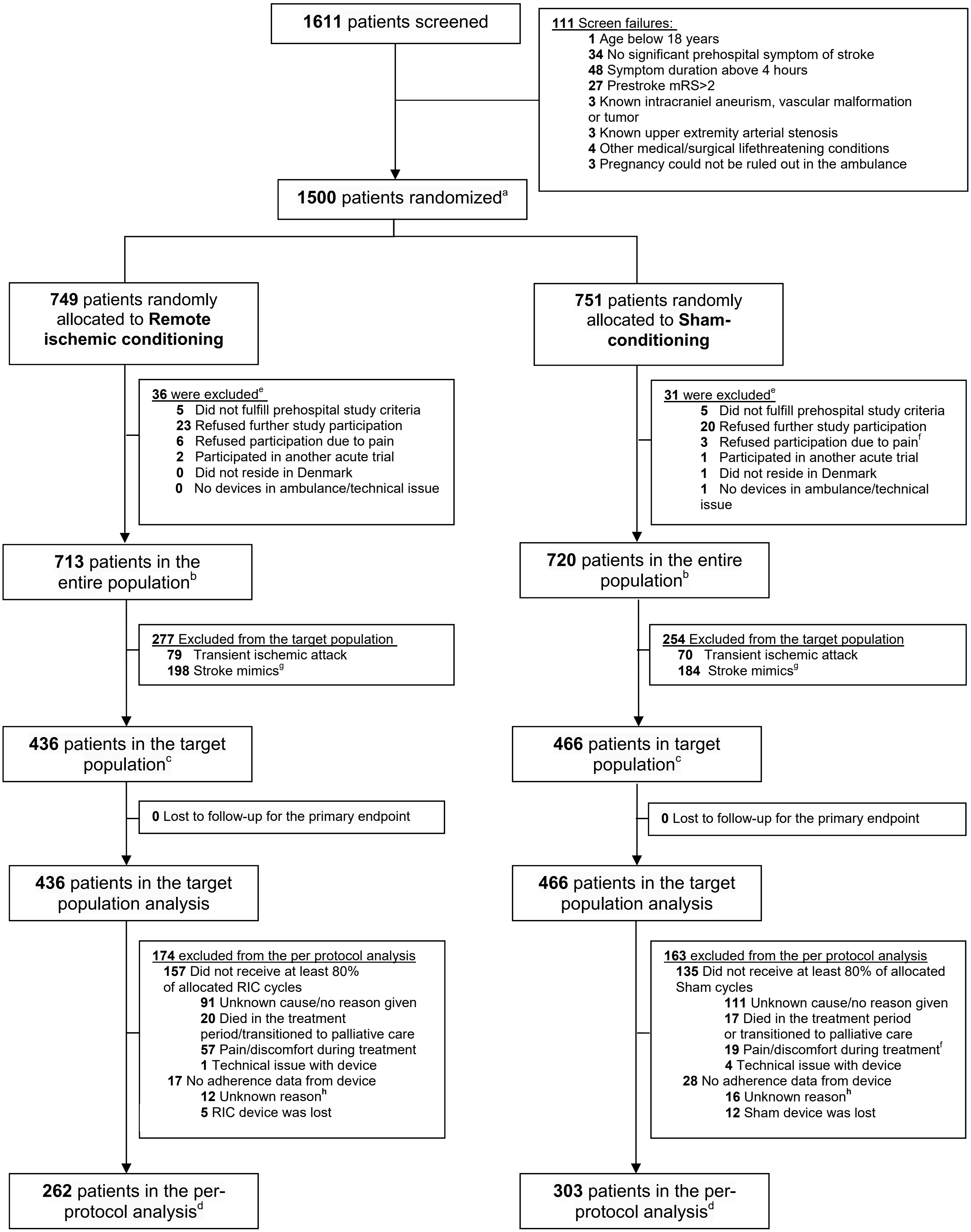
1611 patients screened
111 Screen failures:
1 Age below 18 years
34 No significant prehospital symptom of stroke
48 Symptom duration above 4 hours
27 Prestroke mRS>2
3 Known intracraniel aneurism, vascular malformation or tumor
3 Known upper extremity arterial stenosis
4 Other medical/surgical lifethreatening conditions
3 Pregnancy could not be ruled out in the ambulance
1500 patients randomized[a]
749 patients randomly allocated to **Remote ischemic conditioning**
751 patients randomly allocated to **Sham-conditioning**
36 were excluded[e]
5 Did not fulfill prehospital study criteria
23 Refused further study participation
6 Refused participation due to pain
2 Participated in another acute trial
0 Did not reside in Denmark
0 No devices in ambulance/technical issue
31 were excluded[e]
5 Did not fulfill prehospital study criteria
20 Refused further study participation
3 Refused participation due to pain[f]
1 Participated in another acute trial
1 Did not reside in Denmark
1 No devices in ambulance/technical issue
713 patients in the entire population[b]
720 patients in the entire population[b]
277 Excluded from the target population
79 Transient ischemic attack
198 Stroke mimics[g]
254 Excluded from the target population
70 Transient ischemic attack
184 Stroke mimics[g]
436 patients in the target population[c]
466 patients in target population[c]
0 Lost to follow-up for the primary endpoint
0 Lost to follow-up for the primary endpoint
436 patients in the target population analysis
466 patients in the target population analysis
174 excluded from the per protocol analysis
157 Did not receive at least 80% of allocated RIC cycles
91 Unknown cause/no reason given
20 Died in the treatment period/transitioned to palliative care
57 Pain/discomfort during treatment
1 Technical issue with device
17 No adherence data from device
12 Unknown reason[h]
5 RIC device was lost
163 excluded from the per protocol analysis
135 Did not receive at least 80% of allocated Sham cycles
111 Unknown cause/no reason given
17 Died in the treatment period or transitioned to palliative care
19 Pain/discomfort during treatment[f]
4 Technical issue with device
28 No adherence data from device
16 Unknown reason[h]
12 Sham device was lost
262 patients in the per-protocol analysis[d]
303 patients in the per-protocol analysis[d]

Print Form

CONSORT CHECKLIST

Table. CONSORT 2010 Checklist of Information to Include When Reporting a Randomized Trial[a]

Section and Topic	Item No.	Checklist Item	Reported on Page No.
**Title and abstract**			
	1a	Identification as a randomized trial in the title	1
	1b	Structured summary of trial design, methods, results, and conclusions (for specific guidance see CONSORT for abstracts)	N/A
**Introduction**			
Background and objectives	2a	Scientific background and explanation of rationale	2
	2b	Specific objectives or hypotheses	2
**Methods**			
Trial design	3a	Description of trial design (such as parallel, factorial) including allocation ratio	2
	3b	Important changes to methods after trial commencement (such as eligibility criteria), with reasons	N/A
Participants	4a	Eligibility criteria for participants	2
	4b	Settings and locations where the data were collected	2
Interventions	5	The interventions for each group with sufficient details to allow replication, including how and when they were actually administered	2
Outcomes	6a	Completely defined prespecified primary and secondary outcome measures, including how and when they were assessed	2
	6b	Any changes to trial outcomes after the trial commenced, with reasons	2
Sample size	7a	How sample size was determined	N/A
	7b	When applicable, explanation of any interim analyses and stopping guidelines	2
Randomization			
Sequence generation	8a	Method used to generate the random allocation sequence	N/A
	8b	Type of randomization; details of any restriction (such as blocking and block size)	N/A
Allocation concealment mechanism	9	Mechanism used to implement the random allocation sequence (such as sequentially numbered containers), describing any steps taken to conceal the sequence until interventions were assigned	N/A
Implementation	10	Who generated the random allocation sequence, who enrolled participants, and who assigned participants to interventions	N/A
Blinding	11a	If done, who was blinded after assignment to interventions (for example, participants, care providers, those assessing outcomes) and how	2
	11b	If relevant, description of the similarity of interventions	2
Statistical methods	12a	Statistical methods used to compare groups for primary and secondary outcomes	2
	12b	Methods for additional analyses, such as subgroup analyses and adjusted analyses	2
**Results**			
Participant flow (a diagram is strongly recommended)	13a	For each group, the numbers of participants who were randomly assigned, received intended treatment, and were analyzed for the primary outcome	2
	13b	For each group, losses and exclusions after randomization, together with reasons	2
Recruitment	14a	Dates defining the periods of recruitment and follow-up	2
	14b	Why the trial ended or was stopped	2
Baseline data	15	A table showing baseline demographic and clinical characteristics for each group	2
Numbers analyzed	16	For each group, number of participants (denominator) included in each analysis and whether the analysis was by original assigned groups	2
Outcomes and estimation	17a	For each primary and secondary outcome, results for each group, and the estimated effect size and its precision (such as 95% confidence interval)	2
	17b	For binary outcomes, presentation of both absolute and relative effect sizes is recommended	2
Ancillary analyses	18	Results of any other analyses performed, including subgroup analyses and adjusted analyses, distinguishing prespecified from exploratory	2
Harms	19	All important harms or unintended effects in each group (for specific guidance see CONSORT for harms)	2
**Comment**			
Limitations	20	Trial limitations, addressing sources of potential bias, imprecision, and, if relevant, multiplicity of analyses	3
Generalizability	21	Generalizability (external validity, applicability) of the trial findings	3
Interpretation	22	Interpretation consistent with results, balancing benefits and harms, and considering other relevant evidence	3
**Other information**			
Registration	23	Registration number and name of trial registry	2
Protocol	24	Where the full trial protocol can be accessed, if available	2
Funding	25	Sources of funding and other support (such as supply of drugs), role of funders	2

[a] We strongly recommend reading this statement in conjunction with the CONSORT 2010 Explanation and Elaboration for important clarifications on all the items. If relevant, we also recommend reading CONSORT extensions for cluster randomized trials, noninferiority and equivalence trials, nonpharmacological treatments, herbal interventions, and pragmatic trials. Additional extensions are forthcoming: for those and for up-to-date references relevant to this checklist, see http://www.consort-statement.org.

©2010 American Medical Association. All rights reserved.

This supplement contains:

1. **Final protocol** (Clinical Investigation Plan version 8.2, March 31, 2020)
2. **Summary of changes** (From version 7.0, February 9, 2018 to version 8.2, March 31, 2020)
3. **Original protocol** (Clinical Investigation Plan version 7.0, February 9, 2018)
4. **Protocol amendment** (Clinical Investigation Plan version 8.0, November 29, 2018)
5. **Protocol amendment** (Clinical Investigation Plan version 8.1, August 29, 2019)

1. Final protocol

(Clinical Investigation Plan version 8.2, March 31, 2020)

Clinical Investigation Plan

Remote ischemic conditioning in patients with acute stroke: a multicenter randomized, patient-assessor blinded, sham-controlled study.

A multicenter, prospective, randomized, patient-assessor blinded, sham controlled study investigating whether remote ischemic conditioning (RIC) can improve recovery.

**Project acronym**	**RESIST stroke trial**
	**RE**mote i**S**chemic conditioning **I**n patients with acute **ST**roke: a multicenter randomized, patient-assessor blinded, sham controlled study examining the effect on recovery
**Clinical investigation plan**	**Version 8.2, 31.03.2020**
**Sponsor/Principal investigator (Aarhus)**	Grethe Andersen, Senior Consultant, MD, DMSc, Professor of Neurology. Department of Neurology, Aarhus University Hospital, DK-8200, Denmark. Phone:+4523886653, e-mail: greander@rm.dk
**Study Coordinator & investigator**	Rolf Blauenfeldt, MD, PhD Student. Department of Neurology, Aarhus University Hospital, DK-8200, Denmark. Phone: +4520774053, e-mail: rolfblau@rm.dk
**Principle Investigator (Holstebro)**	Birgitte Forsom Sandal, Senior Consultant, MD. Department of Neurology, Regional Hospital West Jutland (Holstebro), DK-7500, Denmark. Phone: +45 78437280, e-mail: Birgitte.Forsom.Sandal@vest.rm.dk
**Principle Investigator (Odense)**	Anne-Mette Homburg, Senior Consultant, MD. Department of Neurology. Odense University Hospital, DK-5000, Denmark. Phone: +45 65414691, e-mail: anne-mette.homburg@rsyd.dk
**Principle Investigator (Aalborg)**	Boris Modrau, Senior Consultant, PhD-student, MD. Department of Neurology. Aalborg University Hospital, DK-9000, Aalborg. Phone: +45 97662239, e-mail: boris.modrau@rn.dk

RESIST trial is conducted according to the clinical investigation plan and current Danish legislation

The Clinical Investigation Plan will be approved and signed by all investigators before study start.

Contents

1. Synopsis 3
2. Abbreviations 7
3. Introduction 9
4. Hypothesis 10
5. Objectives 10
6. Trial design 11
7. Selection and withdrawal of subjects 13
8. Study procedure and assessment 16
9. Endpoints 20
10. Benefit of the study 32
11. Assessment of safety 32
12. Project timetable and recruitment feasibility 37
13. Ethical considerations 37
14. Data handling and record keeping 37
15. Publications policy 38
16. Statistics 38
17. Source data access and monitoring 43
18. Economy 43

47

1. Synopsis

**Name of the Sponsor/principal investigator**<br>Grethe Andersen, Senior Consultant, MD, DMSc, Professor of Neurology, Department of Neurology, Aarhus University Hospital, DK-8000, Denmark
**Name of investigational medical device: "Stroke RIC" & "Sham RIC"**<br>The investigational devices is developed in collaboration with the Faculty of Biomedical Engineering, Aarhus University, 8200-DK, Aarhus N, Denmark; Seagull Aps, 4160-DK, Herlufmagle, Denmark, and the Department of Neurology, Aarhus University Hospital, 8000-DK, Aarhus C, Denmark.
**Title of study: RESIST**<br>Remote ischemic conditioning in patients with acute stroke: a multicenter randomized, patient-assessor blinded, sham-controlled study, examining the effect on recovery.
**<u>Trial Management Groups (TMG):</u>**<br>**Principal investigator/Sponsor (Aarhus University Hospital):**<br>Grethe Andersen, Senior Consultant, MD, DMSc, Professor of Neurology, Department of Neurology, Aarhus University Hospital, Palle Juul Jensens Boulevard 165, 8200 Aarhus C, Denmark<br><br>**Study Coordinator/Investigator**<br>Rolf Ankerlund Blauenfeldt, MD, PhD student. Department of Neurology, Aarhus University Hospital, Palle Juul Jensens Boulevard 165, 8200 Aarhus C, Denmark<br><br>**Trial Steering Committee (TMC):**<br>Grethe Andersen (Aarhus University Hospital), Rolf Ankerlund Blauenfeldt (Aarhus University Hospital), Niels Hjort (Aarhus University Hospital), Hans Erik Bøtker (Aarhus University Hospital), David C. Hess (Medical College of Georgia, USA), Hans Kirkegaard (Aarhus University Hospital). Rikke Bay Thomsen (Aarhus University Hospital), Birgitte Forsom Sandal (Regional Hospital West Jutland, Holstebro) and Marc Fisher (Beth Israel Deaconess Medical Center, Harvard Medical School, USA), Claus Simonsen (Aarhus University Hospital), Anne-Mette Homburg (Odense University Hospital) and Boris Modrau (Aalborg University Hospital)<br><br>**Data Monitoring Committee (and Trial Safety Committee):**<br>***The DMSC comprises of:***<br>Jesper Petersson (Skåne University Hospital, Sweden) – chair.<br>Jan Brink Valentin (Aalborg University Hospital, Denmark)<br>Thomas Christensen (Nordsjællands Hospital, Denmark)<br><br>**Trial Endpoints Validation Committee:**<br>EVC comprises of Independent senior consultants in Neurology and Cardiology<br>**Trial monitoring:**<br>During the entire study period and at all participating centres the regional GCP unit will perform quality assurance control including source data verification (GCP Unit Aarhus/Aalborg, ID 2017-718)
**Study centers:**

Department of Neurology, Aarhus University Hospital, DK-8000 Aarhus C, Denmark
Department of Neurology, Holstebro Hospital, DK-7500 Holstebro, Denmark
Department of Neurology, Odense University Hospital, DK-5000 Odense, Denmark
(Future study center, 2019 or 2020)
Department of Neurology. Aalborg University Hospital, DK-9000, Aalborg, Denmark
(Future study center, 2019 or 2020)

All Danish stroke centers will be invited to participate.

Planned study period: 2018-2022.

Phase of development:
Improve routine care of patients with acute stroke and transitory ischemic attack (TIA).

Objectives:
To determine whether combined remote ischemic per- and postconditioning can improve long-term recovery in acute stroke patients as an adjunct to standard treatment.

Diagnosis:
Acute ischemic stroke (AIS) and intracerebral hemorrhage (ICH).

Methodology:
A multicenter, investigator-driven, prospective, randomized, parallel assignment, patient-assessor blinded, sham-controlled clinical efficacy trial.

Randomization:

- Eligible patients will be randomized prior to the arrival at the hospital (via a secure web site).
- The patients and the clinical outcome assessor will be blinded to the treatment allocation.

Number and subjects (planned):

- 1,500 patients

Inclusion criteria (prehospital):
The initial treatment regime will be applied in the acute prehospital phase in setting of an acute research study

- Male and female patients (≥ 18 years)
- Prehospital putative stroke
- Onset of stroke symptoms < 4 hours before remote ischemic conditioning (RIC)
- Independent in daily living before symptom onset (mRS ≤ 2)

Final in-hospital inclusion criteria

- acute ischemic stroke including documented TIA

  or

- ICH

TIA without documented ischemic lesion and non-vascular diagnosis will only have register-based long term follow-up only.

Exclusion criteria

Exclusion criteria, to be established during the teleconference between ambulance and on call neurologist

- Intracranial aneurisms, arteriovenous (AV) malformation, cerebral neoplasm abscess or progressive neurodegenerative disease
- Pregnancy
- Severe peripheral arterial disease in the upper extremities
- AV shunt in the arm selected for RIC
- Concomitant acute life-threatening medical or surgical condition

Criteria for evaluation

Primary endpoints

- Clinical outcome (mRS) at 3 months in acute stroke (AIS and ICH)

Secondary endpoints

- Difference in prehospital stroke score (PreSS) during 24 hours in all randomized patients
- Clinical outcome (mRS) at 3 months in subgroups: AIS, IV tPA/EVT treated AIS and ICH
- Difference in prehospital stroke score (PreSS) during 24 hours in subgroups: IV tPA/EVT treated AIS and ICH.
- Difference in proportion of patients with complete remission of symptoms within 24 hours (TIA)
- Major Adverse Cardiac and Cerebral Events (MACCE) and recurrent ischemic events based on registry data at 3 and 12 months in ICH, AIS patients, TIA and non-vascular diagnosis
- Three-month and one-year mortality in AIS, ICH, and overall
- Early and very early neurological improvement in AIS and ICH patients
- Bed-day use at 3 and 12 months and quality of life measures at 3 months

Sub-studies at Aarhus University Hospital (secondary endpoints)

- Clinical outcome (mRS) at 3 months in patients with AIS and ICH and extended remote ischemic postconditioning protocol (*substudy at Aarhus University Hospital*)
- Proportion of RIC/Sham-RIC treated AIS patients with large vessel occlusion (LVO) eligible to EVT treatment (*substudy at Aarhus University Hospital*)
- Difference in acute (24-hour) hematoma expansion in patients with ICH

- Difference in hematoma reabsorption rate (7 days) in patients with ICH
- Infarct growth in AIS patients and hematoma growth in ICH patients
- Predictive abilities of Glial Fibrillary Acidic Protein (GFAP) and occludin in prehospital obtained blood samples combined with prehospital stroke severity to differentiate hemorrhagic from ischemic stroke and to grade ischemic stroke severity (*substudy at Aarhus University Hospital*)
- Diagnostic abilities of a prehospital microRNA and extracellular vesicles blood samples profile combined with prehospital stroke severity on the differentiation of hemorrhagic from ischemic stroke and to grade ischemic stroke severity (*substudy at Aarhus University Hospital*)
- microRNA and extracellular vesicle profile of RIC-induced neuroprotection at baseline (*substudy at Aarhus University Hospital*)
- Coagulation profile of putative stroke patients in prehospital obtained blood samples (*substudy at Aarhus University Hospital*)
- Modulation of coagulation by RIC (*substudy at Aarhus University Hospital*)
- Characterization of rheoerythrocrine dysfunction (RBC deformability, eryNOS3 and plasma nitrite) in RIC vs Sham-RIC treated stroke patients and its possible association to improved short term (24 hour) or long term (90 day mRS) clinical outcome or imaging biomarkers (DWI infarct growth) (*substudy at Aarhus University Hospital*)
- Prestroke physical activity level (PASE) as a predictor for early and long-term recovery (*substudy at Aarhus University Hospital*)
- One week blood-pressure reduction in AIS and ICH patients (*substudy at Aarhus University Hospital)*

Study flow chart

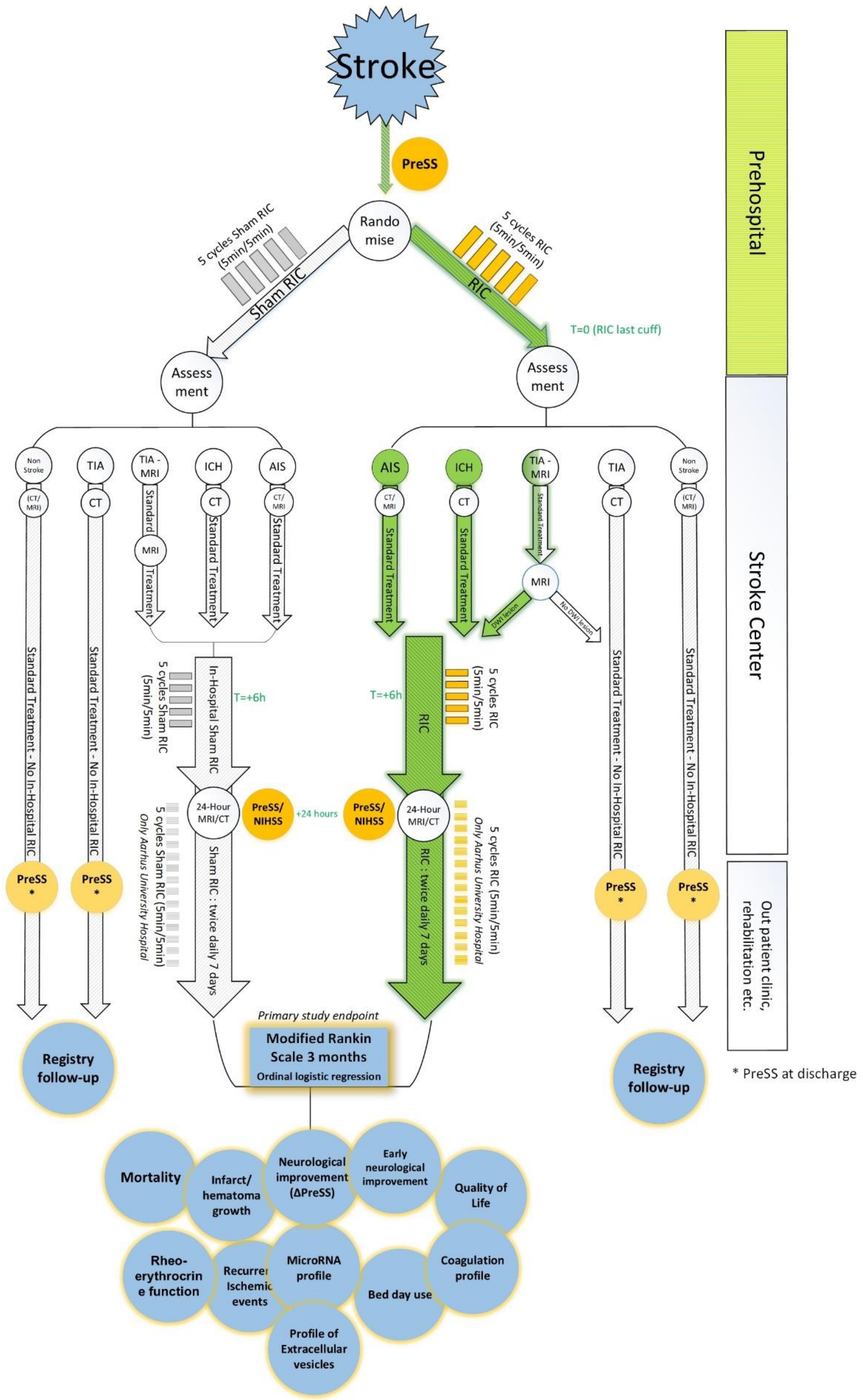

Abbreviations

AE	Adverse event
AIS	Acute ischemic stroke
AR	Adverse reaction
CT	Computed tomography
CPSS	Cincinnati Prehospital Stroke Scale
DWI	Diffusion-weighted imaging
DMC	Data Monitoring Committee and Endpoints Validation Committee
ICH	Intracerebral hemorrhage
END	Early neurological deterioration
EVT	Endovascular treatment
LVO	Large Vessel Occlusion
miRNA	Micro ribonucleic acid
mPTP	Mitochondrial permeability transition pore
MRI	Magnetic resonance imaging
NPR	Danish National Patient Register
PreSS	Prehospital Stroke Score
PWI	Perfusion-weighted imaging
RIC	Remote ischemic Conditioning
RIPerC	Remote Ischemic perconditioning
RIPreC	Remote ischemic preconditioning
RIPostC	Remote ischemic postconditioning
IV tPA	Intravenous thrombolysis/recombinant tissue-type plasminogen activator
SAE	Serious adverse event
SAR	Serious adverse reaction
TIA	Transient ischemic attack
TIA (DWI+)	Transient ischemic attack with a DWI-positive lesion on MRI
TMG	Trial Management Group
TSC	Trial Steering Group

2. Introduction

Our primary aim is to investigate whether remote ischemic conditioning (RIC) as an adjunctive treatment can improve long-term recovery in acute stroke patients as an adjunct to standard treatment.

Stroke is the second-leading cause of death worldwide and a leading cause of serious, long-term disability. The most common type is AIS which occurs in 85% of cases. Acute cerebral thromboembolism leads to an area of permanent damage (infarct core) in the most severely hypoperfused area and a surrounding area of impaired, yet salvageable tissue known as the "ischemic penumbra"[1]. Intravenous alteplase (IV tPA) and endovascular treatment (EVT) are approved acute reperfusion treatments of AIS to be started within the first 4½-6 hours and as soon as possible after symptom onset to prevent the evolution of the infarct core[2,3,4,5]. The prognosis has improved overall for ischemic stroke and the one-month mortality rate has declined to an approx. 10% [6]. However, reperfusion itself may paradoxically result in tissue damage (reperfusion injury) and may contribute to infarct growth[7,8,9]. Infarct progression can continue for days following a stroke, and failure of the collateral flow is a critical factor determining infarct growth[10]. The pre-eminent therapeutic aim is to restore blood flow, improve collateral perfusion, and prevent reperfusion injury[11]. In transient ischemic attack (TIA), the ischemia and stroke symptoms are only temporary, but these patients possess a high risk of re-stroke – especially patients with evidence of infarction on neuroimaging[12]. TIA patients with a documented ischemic lesion are, per definition, handled as ischemic stroke in this study.

On the other hand, in ICH the culprit is an eruption of blood into the brain parenchyma causing tissue destruction with a massive effect on adjacent brain tissues. Hematoma expansion as well as inflammatory pathways that are activated lead to further tissue damage, edema, and penumbral hypoperfusion[13]. The prognosis after ICH is poor with a one-month mortality of 40%. Acute blood pressure lowering is recommended to prevent further hematoma growth, but the mechanisms behind this effect are not understood[14].

Novel therapeutics and neuroprotective strategies that can be started ultra-early after symptom onset are urgently needed to reduce disability in both AIS and ICH.

Ischemic conditioning is one of the most potent activators of endogenous protection against ischemia-reperfusion injury[15]. RIC can be applied as repeated short-lasting ischemia in a distant tissue that results in protection against subsequent long-lasting ischemic injury in the target organ[16]. This protection can be applied prior to or during a prolonged ischemic event as remote ischemic preconditioning (RIPreC) and perconditioning (RIPerC), respectively, or immediate after reperfusion as remote ischemic postconditioning (RIPostC)[17]. RIC is commonly achieved by inflation of a blood pressure cuff to induce 5-minute cycles of limb ischemia alternating with 5 minutes of reperfusion. RIC activates several protective mechanisms, through humoral and neuronal pathways[18]. Circulating microRNA appears to be intimately involved in the RIC stimulus and might act as possible effector molecules[19,20].

Preclinical studies show that RIC induces a promising infarct reduction in an experimental stroke model[21,22]. It has been demonstrated that RIC protects against ischemia-reperfusion injury in the heart, lung, kidney, and the brain[22]. Results from a recent proof-of-concept study at our institution indicate that RIPerC applied during ambulance transportation as an adjunctive to in-hospital IV tPA increases brain tissue survival after one month[23]. Furthermore, RIPerC patients had less severe neurological symptoms at admission and tended to have decreased perfusion deficits[23]. Another study using RIPreC found a decreased stroke recurrence and shorter time to recovery in patients with intracranial arterial stenosis[24, 25]. Furthermore,

emerging preclinical data indicate a significant increased hematoma reabsorption rate and an improved functional outcome after RIC-treated ICH[26].

To-date, no serious adverse events have been documented in RIC. The procedure has been applied in numerous cardiovascular ischemic patients and in patients suffering from cerebral hemorrhage (ICH/SAH)[23,27,28,29,30].

RIC is a non-pharmacologic and non-invasive treatment without noticeable discomfort that has first-aid potential worldwide[31]. However, whether combined remote ischemic per- and postconditioning can improve long-term recovery in AIS and ICH has never been investigated in a randomized controlled trial.

3. Hypothesis

RIC as an adjunctive treatment improves long-term functional recovery in AIS and ICH patients.

4. Objectives

Primary objectives

- To determine whether RIC improves the clinical outcome (mRS) at 3 months in AIS and ICH

Secondary objectives

- To determine whether RIC reduces neurological impairment (PreSS) in all randomized patients
- To determine whether RIC improves the clinical outcome (mRS) at 3 months in subgroups of AIS, IV tPA/EVT-treated AIS patients and ICH patients
- To determine whether RIC reduces neurological impairment (PreSS) at 24 hours in subgroups of IV tPA/EVT treated AIS and ICH.
- To determine whether RIC increases the proportion of patients with complete remission of symptoms within 24 hours (TIA; both with and without DWI lesion)
- To determine whether RIC reduces major adverse cardiac and cerebral events (MACCE) and reduces recurrent ischemic events at 3 and 12 months
- To determine whether RIC reduces the 3-month and 1-year mortality in patients with AIS, ICH, and overall
- To determine whether RIC reduces bed-day use at 3 and 12 months and affects quality of life at 3 months
- To determine whether RIC increases the occurrence of early and very early neurological improvement in AIS and ICH patients

Secondary endpoints (only Aarhus University Hospital)

- To determine whether an extended RIC protocol (one week) improves clinical outcome (mRS) at 3 months in patients with AIS and ICH (*substudy at Aarhus University Hospital*)
- To determine whether RIC Increased the proportion of AIS patients with large vessel occlusion (LVO) eligible to EVT treatment (*substudy at Aarhus University Hospital*)

- To determine whether RIC reduces infarct growth in AIS patients and hematoma growth in ICH patients (s*ubstudy at Aarhus University Hospital*)
- To determine whether RIC increases the hematoma reabsorption rate (7 days) in patients with ICH (s*ubstudy at Aarhus University Hospital*)
- To determine whether prehospital obtained Glial Fibrillary Acidic Protein (GFAP) and occludin in blood samples combined with prehospital stroke severity can differentiate hemorrhagic from ischemic stroke and grade ischemic stroke severity (*substudy at Aarhus University Hospital*)
- To determine whether prehospital obtained microRNA and extracellular vesicles blood samples combined with prehospital stroke severity can differentiate hemorrhagic from ischemic stroke and grade ischemic stroke severity (*substudy at Aarhus University Hospital*)
- To determine whether a circulating microRNA and/or extracellular vesicle profile of RIC-induced neuroprotection can be established (s*ubstudy at Aarhus University Hospital*)
- To determine whether the prehospital determined coagulation profile from putative stroke patients differs between hemorrhagic, ischemic stroke and non-stroke (s*ubstudy at Aarhus University Hospital*)
- To determine whether RIC modulates coagulation in hemorrhagic and ischemic stroke, and explore links between coagulation and infarct growth in AIS, hematoma expansion in ICH, and MACCE and recurrent ischemic events at 3 and 12 months in both AIS and ICH (s*ubstudy at Aarhus University Hospital*)
- To characterize the rheoerythrocrine dysfunction (RBC deformability, eryNOS3 and plasma nitrite) in RIC vs Sham-RIC treated stroke patients and its possible association to improved short term (24 hour) or long term (90 day mRS) clinical outcome or imaging biomarkers (DWI infarct growth) (s*ubstudy at Aarhus University Hospital*)
- To determine whether prestroke physical activity level (PASE) is a predictor for early and long-term recovery (*substudy at Aarhus University Hospital*)
- To determine whether RIC reduces one week blood-pressure in AIS and ICH patients (*substudy at Aarhus University Hospital)*

5. Trial design

Trial design

This is a multicenter, prospective, randomized, patient-assessor blinded, sham-controlled trial investigating whether RIC improve long-term recovery in acute stroke.

Number of centers

Patients with putative stroke from participating stroke centers in Denmark will be recruited.

Number of subjects

We estimate that a sample size of 1500 prehospital putative stroke patients will be required to achieve 1000 eligible AIS and ICH patients (primary study endpoint).

Sample size determination

Primary clinical endpoint

The treatment effect of RIC on long-term functional outcome is unknown. We have assumed a small but clinical significant neuroprotective effect corresponding to a 7% increased odds for a beneficial shift on the modified Rankin Scale. The sample size calculation was based on a simulation-based approach to the analysis of statistical power when ordinal logistic regression analysis is performed (significans level of 5%)

The statistical power was simulated at different hypothetical sample sizes (on the target population) (ranging from 200 to 1900) with 2000 simulation-runs performed at each step. Unpublished data on IV-tPA and/or EVT treated AIS patients and ICH patients from our institution were used:

3 months modified Rankin Scale distribution (proportions) in 2017 for patients with ICH or IV tPA/EVT treated AIS at Aarhus University Hospital.

**Modfied Rankin Scale score**	**mRS 0**	**mRS 1**	**mRS 2**	**mRS 3**	**mRS 4**	**mRS 5**	**mRS 6**
Proportion	0.139	0.273	0.141	0.110	0.145	0.07	0.126

Based on our previous trial experience with prehospital remote ischemic conditioning we estimate that a sample of 1000 subjects with target diagnosis (AIS and ICH) will be feasible to include during the study period.

Including 1000 patients with target diagnosis provide sufficient power at a alfa-level (significans) of 5% to detect RIC treatment effects of the estimated 7 % *(see table below)*

**Treatment effect**<br>**(assumed neuroprective)**	5%	6%	**7%**
Sample (target diagnosis), *n*	1000	1000	**1000**
Power	66%	80%	**90%**
Alpha level (significans level)	5%	5%	**5%**

The estimated prehospital, randomized, sample:

**Sample size, prehospital**	**Proportion of randomized**	***n =***
**Target diagnosis (AIS and ICH), *n***	**67%**	**1000**
Non-vascular diagnosis	**27%**	**403**
TIA without DWI lesion	**4%**	**60**
Lost to follow-up	**2%**	**30**
Total		**1492**
**Plan to include**		**1500**

We therefor plan to include 1500 patients with a prehospital putative stroke in order to get 1000 patients with the target diagnosis of acute ischemic stroke and intracerebral hemorrhage. There is no planned replacement of patients lost to follow-up.

Randomization

Randomization procedure

The patient will be randomized to standard treatment with RIC or sham-RIC by the on call neurologist/vascular neurologist at the receiving stroke center. The ambulance will contact the on call neurologist by telephone and describe the patient (standard operating procedure (SOP) in Denmark). The randomization is based on a secure web site providing computer-generated blocked randomization lists stratified by the center. The online randomization is stratified by age, strokecenter and the Prehospital Stroke Score (PreSS). The PreSS score consists of the Cincinnati Prehospital Stroke Scale (CPSS) with an additional opportunity to report other neurological symptoms (e.g. ataxia, sensory disturbances and visual field loss), and PASS (Prehospital Acute Stroke Severity Scale)[32,33]. Prehospital personnel participating in RESIST are trained in identifying stroke symptoms included in PreSS. The on call neurologist will make an assessment based on all available information whether the patient is eligible to participate in RESIST. Randomization is performed in the prehospital setting. Each on call neurologist participating in the study will receive unique access and will have no influence on the randomization process. All neurologists on call will be educated and trained in performing evaluation of potential eligible study candidates and to perform the online randomization during the telephone call with the ambulance personnel. There is always one neurologist on call in participating centers (hospital SOP).

Treatment allocation

1:1 Allocation

6. Selection and withdrawal of subjects

Selection of patients

All patients with a putative stroke who meet the study criteria will be included.

- Depending on the prehospital randomization (RIC versus sham-RIC), patients with AIS will continue RIC or sham-RIC treatment for an extended period. This group includes TIA with a documented DWI lesion on magnetic resonance imaging (MRI).
- Patients with ICH will continue in-hospital RIC or sham-RIC (according to randomization).

Informed consent for prehospital enrollment

Background:

Acute temporary cognitive impairment after stroke is very common, even in patients with minor stroke or remission of symptoms (TIA) (54%) [34]. Widespread cognitive deficits have been identified in an acute stroke patient population[35]. This finding is in line with the clinical experience in acute stroke care. Whether the putative stroke patients have major cognitive impairment in the prehospital setting can only be tested under calm and quiet in-hospital circumstances. Delivering acute neuroprotective care, it is of utmost

importance that treatment is started as soon as possible, i.e. without delay. RIC is without any known serious adverse events.

According to the Danish research ethics committees, a patient who is a candidate for inclusion in an acute research study is considered incompetent if that person is not able to care for his or her own affairs due to physical or mental impairment. We believe that the vast majority of stroke patients fulfil these criteria, and it is not possible to select the few who are, indeed, competent in the acute phase without formal cognitive testing and thus missing the purpose of acute neuroprotection intervention. The intervention is associated only with mild to moderate discomfort and minimal risk for adverse events.

Acute Prehospital phase:

The initial RIC/sham-RIC treatment regime will be applied in the acute prehospital phase in setting of an acute research study approved by the regional research ethics committees.

Whether further treatment, according to randomization, will be offered depends on the in-hospital examination.

In-hospital inclusion:

After the initial RIC/sham-RIC treatment, in-hospital diagnostic evaluation, and acute treatment, ***the competent patient*** will be presented with a consent form with information about the study. Before this, the on-duty neurologist/research physician/stroke research nurse will have examined the patient both physically and cognitively and will have assessed whether the patient is competent or not. The competent patient will receive oral information by the on-duty neurologist/research physician/stroke research nurse* based on the written patient information. The written patient information will be handed out (*deltager information*). We will ask for the patient's acceptance to be included in the study. The patient can withdraw consent at any time. In addition, the acute nature of the disease necessitates circumvention of the usual requirements concerning a 24-hour time period for consideration and discussion with a lay representative. This has been done in recent comparable acute studies. This approach is acceptable as the intervention is without any known risk and associated with a potential benefit for the patient. In-hospital inclusion and assessment of cognitive impairment will be undertaken during the admission at the stroke center. Every effort will be made to inform the patients and relatives in quiet and undisturbed settings, this will be at the hospital bedroom or the designated room for conversations. We will prioritize the presence of nearest relatives at time of study information and inclusion.

**Will only be performed by experienced stroke research nurses with at least 5 years of Good Clinical Practice experience and 5 years of clinical experience with neuro- or cardiovascular patients. If any questions from patient and/or family remain a research physician will be called. All patients will be included and randomized by a physician.*

For ***the incompetent patients***, informed consent will be obtained from next of kin and an independent physician. The consenters will be provided information (based on the summary of the protocol for the independent physicians and study participant information for the relatives) on the trial to be able to make an informed decision about the patient's participation in this trial. The independent physician will be the on-duty physician at the local department of neurosurgery, neuroanesthesiology, intensive care, neurophysiology, (neuro)radiology or the on duty physician from another specialty with stroke patient experience. On-duty physicians at these departments will receive detailed study information.

A patient who during the acute phase was legal incompetent and included as such, but during the follow-up period is assessed competent will receive oral and written information and we will ask for his/her's informed consent.

Patients who fulfill both prehospital and in-hospital inclusion criteria will, according to randomization, receive RIC/sham-RIC treatment again after +6 hours (***all centres***).

Participation in the extended remote ischemic conditioning(RIC) or sham-RIC (*for a total of 7 days*) study will take place ***only at Aarhus University Hospital***. The device will follow the patient when he/she is discharged from the stroke center.

TIA patients (without DWI lesion) and non-stroke diagnosis patients will be asked for consent to do registry follow-up until one year after discharge. For these patients end-of-study visit is registered at stroke center discharge.

Inclusion criteria (prehospital)

- Male and female patients (≥ 18 years)
- Prehospital putative stroke
- Onset of stroke symptoms < 4 hours before RIC
- Independent in daily living before symptom onset (mRS ≤ 2)

Exclusion criteria

- Intracranial aneurisms, AV malformation, cerebral neoplasm, abscess or progressive neurodegenereative disease
- Pregnancy*
- Severe peripheral arterial disease in the upper extremities
- Concomitant acute life-threatening medical or surgical condition
- AV shunt in the arm selected for RIC

**Women of child-bearing age should be asked about their use of safe birth control methods (contraceptive pill, intrauterine devices both hormonal and non-hormonal, hormonal implants, hormonal depot injection and transdermal hormonal patch). If pregnancy cannot be ruled out in the prehospital phase the patient can't be included. Women with a safe birth control method should be encouraged to use this method during the entire period of active RIC treatment.*

Final in-hospital inclusion criteria

- AIS (including DWI-positive TIA patients)

  or
- ICH

TIA and non-stroke diagnose will only have long-term register based follow-up.

Method of blinding

Outcome assessment is blinded to the treatment arm and obtained by a clinical or telephone assessment of the level of dependency and need for help in daily activities (**modified Rankin Scale**) **(Primary study endpoint**).
No information regarding randomization status will be recorded in the patient record.
Patients and the assessors of endpoints are blinded.

Discontinuation of study participation

A patient can withdraw from the study at any time. Patients can be withdrawn from the study at the principal investigator's discretion. In case the patient cannot be reached by telephone, every effort will be made to contact the patient or to document the outcome regarding new vascular events via registries/electronic health records. If the patient withdraws from the study, the date and the reason for the patient's withdrawal will be recorded. The patient is encouraged to provide information about his or her reason(s) for withdrawal and any experienced adverse effects (AE) during the study.

7. Study procedure and assessment

Prehospital stroke identification

Prehospital putative stroke patients are identified in collaboration with the ambulance personnel and the on-duty neurologist/vascular neurologist at the nearest stroke center (by telephone), which is standard operating procedure in Denmark. All Danish ambulance personnel are trained in identifying putative stroke in the prehospital setting. In the present study protocol, all patients have a prehospital structured assessment of neurological symptoms/findings (based on items contained in PreSS) before randomization – which will further increase stroke symptom awareness.

Baseline data

Baseline data and process indicators are collected from the Danish Stroke Registry (DSR). Additional information about medical history and medication and clinical and physiological data are collected at baseline. Data are recorded in an electronic case report form (CRF) at discharge from the stroke unit. For a complete list of registered data, see *Supplement B*

Acute treatment:

Patients are treated according to the national clinical guidelines on stroke treatment, intravenous thrombolysis and endovascular therapy. All patients are treated with state-of-the-art multidisciplinary care in the setting of a stroke unit monitored by the DSR.

Blood samples

Patients with putative stroke admitted to the Aarhus University Hospital will have blood samples withdrawn, in the ambulance, from peripheral venous catheter (placed as standard operating procedure, SOP). A total of 19mL (9mL + 10mL) will be drawn in the prehospital phase.

Blood samples will also be drawn upon admission to the Aarhus University Hospital Stroke Center as a part of SOP. Additional 21.5mL (3x3mL + 3x3,5mL + 1x2mL) of blood will be drawn at this time.

Blood samples will be drawn again 2 hours (1-3hours) after RIC/sham-RIC completion. Additional 5mL (1x3mL+ 1x2mL) of blood will be drawn at this time*.

Patients will have blood-samples drawn, at Aarhus University Hospital Stroke Center, again 24 (16-32) hours after randomization. This is not standard operating procedure. 21.5mL (3x3mL + 3x3,5mL + 1x2mL) will be drawn at this time*.

Only patients received at the Stroke Center at Aarhus University Hospital will have blood samples drawn. (Prehospital and in-hospital blood samples)

For patients with non-stroke diagnosis: Only if still admitted at the point of time

Not all ambulances will be equipped with for blood sample withdrawal, thus an estimated 400-500 patients will have blood samples drawn in the prehospital phase and in hospital at Aarhus University Hospital.

Blood samples will be stored until completion of the trial in a research biobank.

Patients not admitted at Aarhus University Hospital will have no additional blood samples drawn (neither prehospital or in-hospital)

MicroRNA, extracellular vesicles, Glial Fibrillary Acidic Protein (GFAP), RBC deformability, NOS3, plasma nitrite and coagulation analysis will be performed on the obtained blood. A research bio bank will be established, and all blood samples obtained during the study are stored here. During the entire study and analysis period, the material will be stored at Aarhus University Hospital. The last patient will be included in December 2022. Analysis of blood samples, according to secondary endpoints, will be performed during the study and until 2 years after inclusion of the last patient (no later than 31. December 2024). Hereafter the remaining material will be transferred to a biobank for future unspecified research purposes. We will submit an application to the Danish Data Protection agency for this additional 20-year storage period of the blood samples (until 31 December 2044), after that, the samples will be destroyed. All analysis will be performed in Denmark. Furthermore, for patients participating in the biomarker sub-study at Aarhus University Hospital we will use biochemical lab results taken as part of routine care upon admission.

Investigational devices and Remote ischemic conditioning protocols

Automatic remote ischemic conditioning devices

Both the RIC and sham-RIC device are developed in collaboration with Aarhus University, Faculty of Biomedical Engineering, 8200-DK, Aarhus N, Denmark; Seagull Aps, 4200-DK, Slagelse, Denmark and the Department of Neurology, Aarhus University Hospital, 8000-DK, Aarhus C, Denmark.

The devices are manufactured by Shenzhen Raycome Health Technology Co., Ltd, 3F, 51 Building, No.5 Qiong Yu Road, Hi-Tech Industrial Park, Nanshan District, Shenzhen, China 518057.

The ***Investigator's Brochure (IB)*** *contains a detailed description of the investigational devices including risk analysis assessment.*

A brief user's manual is found in each device bag. No preexisting experience is necessary in order to operate the device. Written and oral information combined (Healthcare professional at participating hospitals) with RESIST trial e-learning material (prehospital personnel) will be offered to familiarize the clinical personnel in using the investigational devices. Patients and relatives will be instructed using written and oral information.

Remote Ischemic Conditioning device (RIC device)

The device is programmed to five cycles (50 minutes), each consisting of five minutes of cuff inflation followed by five minutes with a deflated cuff. The cuff pressure will be 200mmHg; but if initial systolic blood pressure is above 175 mmHg, the cuff is automatically inflated to 35 mmHg above the systolic blood

pressure. This is done to account for a maximum cuff air leakage of 6mmHg per minute and maximum deviation in bloodpressure measurement of 10mmHg. The maximum cuff pressure is 285 mmHg.

Sham – remote ischemic conditioning device (Sham-RIC device)

The device is programmed to five cycles (50 minutes), each consisting of five minutes of cuff inflation followed by five minutes with a deflated cuff. The cuff pressure during inflation will be 20mmHg.

Timestamps, blood pressure before RIC/sham-RIC and total RIC/sham-RIC cycles are recorded and stored on the device (accessible by *universal serial bus,* USB). The RIC and sham-RIC is pre-programmed before being delivered to collaborating centers/ambulances. No data on conditioning cuff pressure will be displayed on device screen.

In cases of short transport time, the RIC/sham-RIC protocol continues during the initial assessment at the stroke center. RIC/sham-RIC stimulation is discontinued just prior to the MRI and continues afterwards if the protocol was insufficient (< 4 cycles). In the cases of insufficient treatment before MRI a complete RIC/sham-RIC protocol will be performed (5 cycles) as soon as possible after MRI. This is done to ensure that all participants receive at least 4 consecutive cycles of RIC/sham-RIC. In centers using CT, the RIC/sham-RIC protocol is continued (if possible) during the scan until a total of 5 cycles are reached. The device automatically stops treatment when the 5 cycles are completed. Except for RIC/sham-RIC, the prehospital observation and treatment are according to standard procedures in the ambulance.

Stroke RIC/Sham-RIC will be used, only if approved by the Danish Medicines Agency

The blood pressure and heartrate measurement, by RIC and Sham-RIC, will only be used for research purposes.

Initial remote ischemic conditioning

(all patients)

RIC/sham-RIC cuff will be placed on the non-paretic upper arm. The cuff is placed on the upper extremity on the same side of the suspected side of cerebral stroke, e.g. in right hemiparesis the cuff on the left arm, and in monosymptomatic aphasia the cuff is placed on the left arm.

Remote ischemic conditioning at +6 hours

(All patients with AIS, ICH – all centers)

At 6 hours after the first RIC/sham-RIC protocol (6 hours from the last cuff-off, respectively), another series of RIC/sham-RIC is performed; five cycles each consisting of 5 minutes of ischemia are followed by 5 minutes of reperfusion. *In the case of mechanical thrombectomy the +6 hour treatment may be delayed and will be started as soon as possible.*

Remote ischemic postconditioning (twice daily for 7 days)

(AIS and ICH – only at Aarhus university Hospital)

The post-conditioning stimulus will be applied twice daily in the first week. This occurs at pre-specified times: 08.00 PM and AM. However, RIC/sham-RIC can be applied no sooner than 10 hours and not later than 21 hours after the initial perconditioning stimulus (last cuff).

Aarhus University Hospital: RIC/sham-RIC treatment will continue for a total of 7 days. If patients are discharged to their home or to another department, they will continue treatment according to written instructions, including application of the device for 7 days. After RIC/sham-RIC on day 7, the device is then returned to the Stroke Center.

RIC/sham-RIC protocol	Accepted time frame
**Prehospital RIC**	< 4 hours from symptom onset
**RIC at +6 h**	4 to 8h from last cuff of the initial RIC
**RIC twice daily for 7 days** – 08.00 PM and AM*	06.00 to 10.00 PM and 06.00 to 10.00 AM

*RIC/sham-RIC can be applied no sooner than 10 hours and no later than 21 hours after the initial perconditioning stimulus.

Device Accountability log

All devices are registered in a *device accountability log*. When the prehospital personnel arrives at the stroke center, with a patient included in RESIST trial, a new device (of the same color) will be handed out. Device ID, ambulance ID, contact details to the ambulance and date of device hand-out will be registered. It will be recorded on a paper-log in the stroke department. *Further details see Investigators Brochure.*

Adherence to treatment:

At least 4 cycles (out of 5) will be required for each series of RIC treatment to be considered with accepted adherence. For the twice daily treatment for 7 days the number of cycles depends on the starting time of the acute RIC treatment and may vary from patient to patient. For this reason, 80% of planned cycles needs to be completed for the +7 days RIC treatment to be considered with accepted adherence.

**Acute RIC**		**+6 hours**		**+7days RIC** (Only Aarhus University Hospital)	
**Planned**	**Accepted**	**Planned**	**Accepted**	**Planned**	**Accepted**
5 cycles (5/5min) 100%	4 Cycles (5/5min) 80%	5 cycles (5/5min) 100%	4 Cycles (5/5min) 80%	100%	80%

Study procedures table

	Acute prehospital and in-hospital phase					+ 6 hour	+ 24 hour			+ 7days		3 months	12 months
	Prehosp. stroke score (PreSS)[1]	Prehosp blood-samples[1]	RIC/Sham-RIC	In-hospital assessment and blood-samples [2]	CT/MRI baseline	+6 hour RIC/sham-RIC [4]	PreSS 24h [2]	CT/MRI 24h[3]	24-hour blood-samples[2]	Extended (7 days) RIC/sham-RIC [5]	CT- 7 days[3]	mRS 3-months[7]	Clinical events during follow-up[6]
**TIA (DWI-) - all**	X	(X)*	X	X	X		X						X
**Non-stroke - all**	X	(X)*	X	X	X		X						X
**AIS incl. TIA (DWI+)**	X		X	X	X	X	X	X				X	X

**– Other¥**													
**ICH - Other¥**	X		X	X	X (CT)	X	X					X	X
**AIS incl. TIA (DWI+) – AUH***	X	(X)	X	X	X(MRI)	X	X	X (MRI)	~~(X)~~	X		X	X
**ICH – AUH***	X	(X)	X	X	X	X	X	X (CT)	~~(X)~~	X	X (CT)	X	X

**AUH = Aarhus University Hospital , ¥ Other= Other participating stroke centers*

[1] Prehospital stroke score (PreSS) ~~and bloodsamples~~.

[2] Acute assessment at the Stroke Center. NIHSS, blood samples (21,5mL blood)-and ECG (standard). Assessment of neurological symptoms at 24-hour: NIHSS. Patients with non stroke diagnosis and TIA without a DWI-MRI lesion, who are discharged before 24 hours will be scored using PreSS at discharge. Patients, who had blood samples taken in the ambulance and upon stroke center arrival will have additional samples withdrawn at 2 (1-3) hours (5mL) after RIC/Sham-RIC completion and again 24(16-32) hours* after randomization (additional 21,5 mL blood) *RESIST blood samples only at Aarhus University Hospital.*

[3] Baseline CT is preferable for ICH patients (hospital SOP). ICH patients, at Aarhus University Hospital, will receive a 24 hour control CT (hospital SOP). If possible, a 24-hour MRI is performed in all included AIS patients with an MRI baseline scan. Furthermore, a 24-hour control CT will be provided for IV tPA-treated AIS patients, unless MRI can be performed and was the primary investigation. One week control CT will be performed, if possible, on ICH patients included at Aarhus University Hospital. End of study visit for non-vascular diagnosis and TIA (without DWI lesion) at stroke center discharge.

[4] RIC/sham-RIC at +6 hours from last RIC cuff

[5] RIC/sham-RIC twice daily for 7 days *(Only at Aarhus University Hospital)*

[6] Mortality, MACCE, and recurrent ischemic events are recorded using the Danish National Patient Register (LPR) and DSR at 6 and 15 months after the inclusion of the last patient.

[7] Assessments at 3 months by telephone or face-to-face (mRS and WHO-5). End-of-study (telephone or face-to-face) for AIS and ICH patients. WHO-5 will only be assessed by one assessor.

8. Endpoints

An independent end-point committee will adjudicate clinical events.

Primary endpoints

Criteria for evaluation

Clinical outcome (mRS) at 3 months in acute stroke

Primary study endpoint: Clinical outcome (modified Rankin Scale) at 3 months in acute stroke patients (generalized ordinal logistic regression)

The level of dependency and need for help in daily activities will be determined by either face-to-face assessment or based on a structured telephone interviews performed by assessors who are blinded to the intervention at 3 months after the index stroke.

The assessment will be performed by two independent blinded assessors, which can be either face-to-face or telephone based assessment (not same day). The electronic CRF database will alert if disagreement occurs, and the patient will be contacted by a third assessor (face-to-face or telephone) who is blinded to the intervention who will assess the level of dependency.

- If disagreement occurs between **two telephone assessments** – a third, and final, telephone or face-to-face assessment will be made.
- If disagreement occurs between one face-to-face assessment and one telephone assessment – the face-to-face will be considered the final assessment
- If disagreement occurs between **two face-to-face assessments** – a third, and final, telephone or face-to-face assessment will be made.

Every possible effort will be made to assess the outcome in patients who are unable to participate in the interview (this could be due to post stroke neurological impairment (for example aphasia and/or dysarthria), or that the patient cannot be reached by telephone). The outcome will then be assessed by contact to a named relative or the general practitioner. Several attempts to contact the patient by telephone will be made before contacting the named relative or general practitioner.
Death in the follow-up period will be obtained from Danish Civil Registration System (CPR).

The structured telephone interview will be based upon a validated Danish translation of the "slightly revised simplified, modified Rankin Scale questionnaire"[36]. The translation and its validation will be performed according to the AAOS guideline[36, 37]

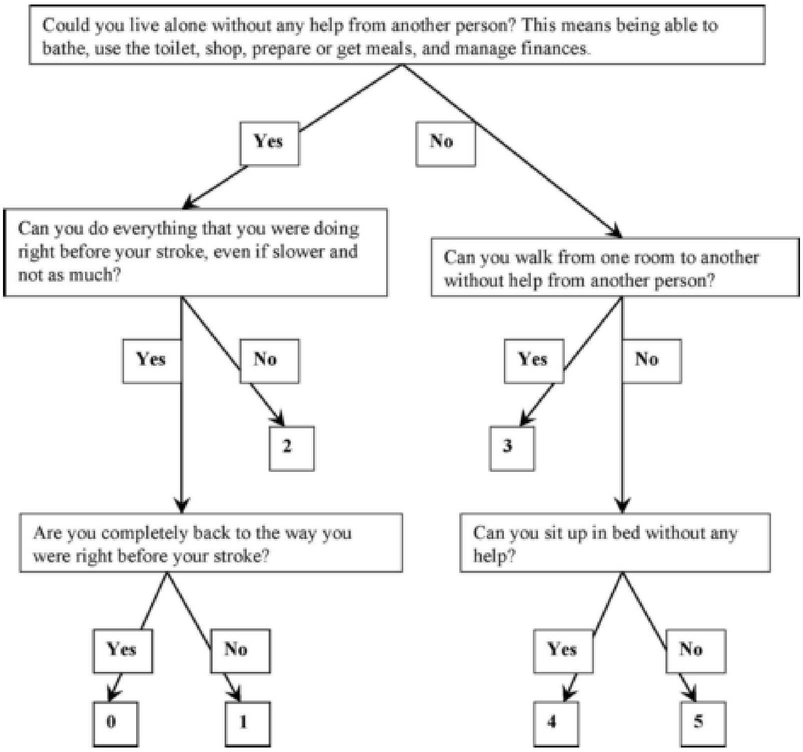

Bruno et al. 2011. Stroke.

Furthermore, modified Rankin Scale assessors are required to have received specific mRS training, which could be an online mRS certification https://secure.trainingcampus.net/uas/modules/trees/windex.aspx?rx=rankin-english.trainingcampus.net

For a **detailed statistical analysis plan**, see **statistics**

Outcome	Accepted time frame (from symptom onset)
3-month follow-up – 1st and 2nd assessment	3 months +/- 2 weeks
3 month follow-up – 3rd assessment	3 months and 2 weeks +/- 2 weeks

Secondary endpoints

Difference neurological impairment during the first 24 hours in all randomized patients

Neurological deficits are documented using PreSS (prehospital and in-hospital). The PreSS score is obtained by the prehospital personnel and registered by the on-call neurologist before randomization. Prehospital Stroke Score is assessed at 24-hour by a trained and blinded general neurology nurse/neurology research nurse/physician/neurologist who is blinded to the treatment intervention. Only information regarding time and place of assessment, patient name, age and social security number will be available. The PreSS score of patients with TIA (without DWI lesion) and non stroke diagnoses, are performed by the on-call physician/neurologist or a research nurse/general neurology nurse, and will be documented as *PreSS at discharge*, if discharge occurs before 24 hours. The outcome assessor will be alerted in advance of the assessment. The PreSS is recorded in the electronic CRF. Clinical assessors are required to complete an online PreSS certification or face-to-face training.

https://ekurser.rm.dk/test_projekter/anhesr/red-hjernen/scorm/Scorm2004%204th%20Edition/index.html

For a **detailed statistical analysis plan**, see **statistics**

Indicator	Accepted timeframe (from symptom onset)
PreSS	Close to symptom onset
$PreSS_{24h}$ (+ 24h)	+ 16 to 32h

Difference in proportion of patients with complete remission of symptoms within 24 hours (TIA; both with and without DWI)

Diagnosis of TIA is documented in the electronic case report form

Endpoint assessment
Difference in proportion of patients with complete remission of symptoms within 24 hours (TIA; both with and without DWI)

Major adverse cardiac and cerebral events (MACCE) and recurrent ischemic events at 3 and 12 months in AIS and all patients

MACCE is defined as:

Cardiovascular events (cardiovascular death, myocardial infarction, acute ischemic or hemorrhagic stroke)

Cardiovascular death: Death from known cardiovascular cause or sudden death from unknown cause (no identified cause of death in medical history and/or autopsy)

Acute myocardial infarction: Admission with a discharge diagnosis of ST-elevation myocardial infarction (STEMI) and non-ST elevation myocardial infarction (NSTEMI) and unstable angina pectoris (UAP)

Stroke: Admission with a discharge diagnosis of acute ischemic or hemorrhagic stroke.
Evaluation is performed using the Danish National Patient Register (LPR) and the DSR at two time points (6 and 15 months after the inclusion of the last patient).

Diagnosis of AIS/TIA, ICH and MI (STEMI, NSTEMI, and UAP) are made according to national clinical practice guidelines.

(http://neuro.dk/wordpress/nnbv/om-iskaemisk-apopleksi/ and http://nbv.cardio.dk/aks)

Recurrent ischemic vascular events at 3 and 12 months in AIS patients

Recurrent ischemic vascular events defined as:

- AIS
- TIA
- MI, STEMI, and NSTEMI

Three-month and one-year mortality

Information about mortality is collected using the CPR and LPR, at two time points (6 and 15 months after the inclusion of the last patient). All-cause mortality is assessed and subdivided into cardiovascular mortality versus non-cardiovascular mortality[40].

Analysis are performed on all randomized patients and according to subgroups.

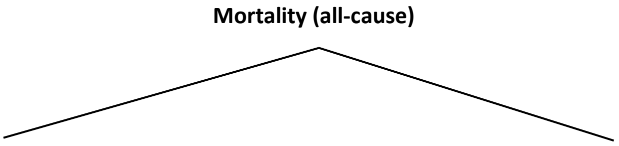

Cardiovascular mortality	Non - cardiovascular mortality
▪ Acute MI<br>▪ Sudden cardiac death<br>▪ Heart failure<br>▪ Stroke: death as a direct consequence or complication of stroke<br>▪ Cardiovascular procedure or procedure<br>▪ Cardiovascular-related hemorrhage: non-stroke intracranial hemorrhage (subdural hematoma), non-traumatic vascular rupture (e.g. aortic aneurysm), or hemorrhage causing cardiac tamponade	▪ Pulmonary, renal, gastrointestinal, hepatobiliary and pancreatic<br>▪ Infection and inflammatory/immune (including autoimmune)<br>▪ Hemorrhage (non-cardiovascular (CV)/non-stroke-related bleeding)<br>▪ Non-CV procedure or surgery, trauma, suicide, drug reaction, or overdose<br>▪ Neurological, malignancy, other non-CV

According to "2014ACC/AHA Key Data Elements and Definitions for Cardiovascular Endpoint Events in Clinical Trial". Circulation 2015;132:302-361.

EVT-eligibility (MRI assessed) in RIC treated AIS patients with large vessel occlusion (LVO)

EVT eligibility is assessed upon arrival at the Aarhus University Hospital in AIS patients with:

- Severe Stroke (NIHSS ≥ 10)
- Groin puncture feasible within 6 hours from stroke onset
- MRI-TOF (time-of-flight) documented internal carotid artery (ICA), Intracranial ICA (ICA-T) and first and second stem of the middle cerebral artery (M1 and M2, respectively)
- No contraindications to MRI (pacemaker, vomiting, respiratory insufficiency, obesity)
- MRI-DWI lesion volume ≤ 70mL

Endpoint assessment

Proportion of RIC treated AIS patients with LVO eligible to EVT treatment compared to standard treatment, adjusted for prehospital stroke severity (PreSS) and symptom duration

Difference in MRI-DWI lesion volume in RIC/sham-RIC treated LVO AIS patients eligible to EVT treatment

Quality of life measures at 3 months in AIS and ICH patients

- **Quality of life and bed-day use measures in AIS and ICH patients**

Quality-of-life measurements are assessed by telephone interview at 3 months after inclusion. The WHO-5 is used. Baseline WHO-5 is only obtained at Aarhus University Hospital.

Link to: Danish National Board of Health - WHO-5.
Information about bed-day use via the LPR and the DSR.

Early neurological improvement in AIS and ICH patients

Neurological deficits are documented with the PreSS and the National Institute of Health Stroke Scale (NIHSS), prehospital and in-hospital, respectively.

- **Very early neurological improvement** is defined as:

Reduction in prehospital stroke score (PreSS) ≥ 1 points or resolution of symptoms at admission: Items on PreSS related to NIHSS baseline $\Delta PreSS = PreSS_{prehospital} -$ same items on $NIHSS_{baseline}$

- **Early neurological improvement** is defined as:

Reduction in NIHSS ≥ 4 (baseline versus 24-Hour NIHSS): $\Delta NIHSS = NIHSS_{baseline} - NIHSS_{24}$

- **Reduction in prehospital stroke score (PreSS) ≥ 1 points or resolution of symptoms after 24 hours in subgroups:** IV tPA/EVT treated AIS and ICH.

Clinical assessors are required to complete an online PreSS certification

PreSS certification: https://ekurser.rm.dk/test_projekter/anhesr/red-hjernen/scorm/Scorm2004%204th%20Edition/index.html

Reduction in NIHSS ≥ 4 (baseline versus 24-Hour NIHSS): $\Delta NIHSS = NIHSS_{baseline} - NIHSS_{24}$

- **Improvement in median percent change in 24-hour NIHSS:**

Median percentage change in NIHSS = $[(NIHSS_{baseline} - NIHSS_{24})/ NIHSS_{baseline}]x100$

Indicator	Accepted timeframe (from symptom onset)
PreSS	Close to symptom onset
NIHSS baseline	0 to 6 hours from admission
NIHSS (+ 24h) – standard operating procedure	+ 16 to 32h
PreSS (+ 24h)	+ 16 to 32h

Secondary endpoints (Only Aarhus University Hospital)

Difference in acute hematoma expansion in patients with ICH

(Substudy only at Aarhus University Hospital)

- Hematoma expansion is defined as the difference in volume between the baseline and the 24-hour CT hematoma volume. Significant hematoma growth is defined as an absolute growth exceeding 6 mL or a relative growth of more than 33% from the initial CT[39].

In all ICH patients, the baseline imaging modality is CT. In cases of initial MRI, a CT is performed afterwards (no later than < 3 hours from last RIC cuff)

Imaging protocol	Accepted time frame
CT baseline – ICH	< 3 hours from last RIC/sham-RIC cuff
MRI baseline – ICH	Perform CT as soon as possible and < 3 hours after the last RIC/sham-RIC cuff
Control CT (24-hour) – ICH	18-30 hours from baseline CT

Information about imaging protocols, see *Supplement A – Neuroimaging protocols*

Difference in 7 days hematoma reabsorption rate in patients with ICH

(Substudy only at Aarhus University Hospital)

- Hematoma reabsorption rate is defined as the difference in hematoma volume between the baseline and the 7-day CT hematoma volume.

In all ICH patients, the preferred baseline imaging modality is CT. In cases of initial MRI, a CT is performed afterwards (no later than < 3 hours from last RIC cuff)

Imaging protocol	Accepted time frame
CT baseline – ICH	< 3 hours from last RIC/sham-RIC cuff
MRI baseline – ICH	Perform CT < 3 hours after the last RIC/sham-RIC cuff if possible
Control CT (7-day) – ICH	5-9 days from baseline CT

Information about imaging protocols, see *Supplement A – Neuroimaging protocols*

Infarct growth in AIS patients

(Substudy only at Aarhus University Hospital)

Acute infarct growth is defined as the difference in infarct volume between baseline and 24 hours on DWI-MRI. All patients with IV-tPA and/or EVT treated ischemic stroke and a baseline MRI will have an additional 24-hour MRI performed.

Imaging protocol	Accepted time frame
MRI baseline – AIS IV tPA eligible	On admission
MRI baseline – AIS not IV tPA eligible	< 6 hours from last RIC/sham-RIC cuff
MRI (24-hour) – AIS IV-tPA/EVT treated	18-30 hours from baseline MRI

Information about imaging protocols, see *Supplement A*

Biochemical profile of stroke subtypes and of RIC-induced neuroprotection

(Substudy only at Aarhus University Hospital)

Prehospital biochemical substudies (Aarhus University Hospital)

- Diagnostic abilities of a prehospital microRNA and extracellular vesicles blood samples profile combined with prehospital stroke severity to differentiate hemorrhagic from ischemic stroke and to grade ischemic stroke severity
- Predictive abilities of Glial Fibrillary Acidic Protein (GFAP) and Occludin in prehospital obtained blood samples combined with a symptom based prehospital stroke score (PreSS – Prehospital Stroke Score). This to differentiate hemorrhagic from ischemic stroke from acute ischemic stroke and to grade ischemic stroke severity to identify patients with large vessel occlusions and to identify patients with a severity that requires admission to a comprehensive stroke center.
- Coagulation profile in prehospital obtained blood samples to differentiate between hemorrhagic stroke, ischemic stroke, and non-stroke
- Red blood cell rheo-erythrocrine dysfunction in acute stroke and its possible association to improved early (24 hour) or long term (90 day mRS) clinical outcome or imaging biomarkers (DWI infarct growth) (*substudy at Aarhus University Hospital*)

In-hospital biochemical substudies

- Characterization of a possible microRNA and extracellular vesicle profile in stroke subtypes
- MicroRNA and extracellular vesicle characterization of a possible RIC treatment profile
- Modulation of coagulation by RIC in hemorrhagic and ischemic stroke
- Characterization of rheoerythrocrine dysfunction (RBC deformability, eryNOS3 and plasma nitrite) in RIC vs Sham-RIC treated in acute stroke patients

Prehospital and in-hospital handling of blood samples:

Blood samples will be collected from patients with a prehospital putative stroke. Thus, blood samples will be drawn in the ambulance and upon arrival at the stroke center (only Aarhus University Hospital). In the ambulance, the blood sample will be obtained through a peripheral venous catheter (placed as standard operating procedure) into a 9mL and 10mL tubes and transported to hospital at ambient temperature (tubes of 9 and 10mL will be drawn). The Thrombosis & Hemostasis Research Unit, Aarhus University Hospital or study personnel will centrifuge the blood and divide the plasma into cryotubes that will be kept at 80 °C for long-term storage. Cryotubes not used to measure GFAP within 5 to 7 days will be stored in a research biobank.

Blood samples will be drawn upon admission to the Aarhus University Hospital Stroke Center as a part of local stroke center SOP. Additional 21,5mL (3 Tubes of 3,5mL, 3 tubes of 3mL and 1 tube of 2mL) of blood will be drawn at this time.

Blood samples will be drawn again 2 hours (1-3hours) after RIC/sham-RIC completion (if the patient is still admitted). Additional 5mL (1x3mL+ 1x2mL) of blood will be drawn at this time.

Patients, who are still admitted will have blood-samples drawn, at Aarhus University Hospital Stroke Center, again 24 (16-32) hours after randomization (21,5mL (3 Tubes of 3,5mL, 3 tubes of 3mL and 1 x 2mL) will be drawn at this time). EDTA, Citrate and Lithium Heparin tubes will be used.

All blood samples obtained during the study will be stored in a research biobank

Blood samples	Accepted time frame
**Acute Prehospital**	As soon as possible
**Acute in-hospital**	As soon as possible
**2-hour in-hospital**	2 hours (1-3 hours) from completed RIC/Sham-RIC
**+24hour in-hospital samples**	< 16-32 hours from randomization and 2 hours (1-3 hours after completion of next day RIC treatment

Blood-samples procedures tables

	Prehospital blood- samples	0-hour in-hospital blood-samples[1]	2-hour in-hospital blood-samples	24-hour in-hospital blood-samples
**Volume**	19mL	21,5mL	5mL	21,5mL
**Non-stroke - AUH**	X	X	X	X*
**ICH, AIS incl. TIA (DWI+) – AUH**	X	X	X	X

[1]0-hour in-hospital blood-samples (21,5mL): Patients who had blood samples taken in the ambulance will upon stroke center arrival will have additional samples withdrawn.

[2]2 (1-3) hours in-hospital blood-samples (5mL): 2 (1-3) hours since completed RIC/sham-RIC (additional 5 mL blood).

[3] 24 (16-32) hours in-hospital blood-samples (21,5mL): 24 (16-32) hours from randomization and 2 hours (1-3) after completion of next day RIC treatment.

*only if still admitted at the time point.

Blood-samples, in the research biobank, will be stored until completion of the trial (no later than 31. December 2024). Hereafter the remaining material will be transferred to a biobank for future unspecified research purposes (see section “Blood Samples” under “study procedure and assessment”)

Use of routine lab results:

The following biochemical entities will be used (lab results performed as a part of standard operating procedure): C-reactive protein, Glucose, HBA1c, Potassium, Natrium, Calcium, Albumin, Creatinine, eGFR (estimated Glomerular Filtration Rate), Total WBC (White Blood Cell count/Leukocytes), Hemoglobin,

Erythrocytes, Erythrocytes (Volume Fraction, EVF), Erythrocytes distribution widths (RDW), Erythrocyte median cell volume (MCV), Hemoglobin concentration (MCHC and MCH), Reticulocyte count, Platelet count, Iron, Transferring, Ferritin, International Normalized Ratio (INR), Activated Partial Tromboplastin Time (APTT), Cholesterol (Total, HDL, LDL, Triglyceride)

Planned biochemical analysis

Glial Acidic Fibrillary Protein (GFAP):

All plasma samples for GFAP and occludin measures will be analyzed with ELISA essay kits from the same batch at the Department of Clinical Biochemistry, Aarhus University Hospital Hospital. Transport and shipment of samples will be done on dry ice to assure stability of the specimens. The samples will be marked with the unique research identification number. Cryotubes not used to measure GFAP and occludin will be stored as part of a biobank.

MicroRNA analysis:
MicroRNAs will be identified with Illumina next-generation sequencing using the TruSeq Small RNA Sample Preparation kit (Illumina) which allows for the addition of unique barcode sequences to each sample. Such barcoding allows pooling and simultaneous sequencing of multiple samples in a single-sequencing run on the Illumina NextSeq500 sequencer thereby significantly reducing the cost of sequencing. Pooling of multiple samples will generate adequate data amounts for detailed miRNA profiling, yet at limited costs. Data generated from the NextSeq500 sequencer will be filtered based on sequence quality as part of our established bioinformatics pipeline. This will also include matching the filtered data to annotated RNA databases such as miRNA sequences from miRBase (mirbase.org).
The output from our bioinformatic pipeline will be quantitative miRNA expression levels for each sample, which will form the basis for an miRNA differential analysis where miRNAs with statistically significant expression changes will be found.
The microRNA (and extraceullar vesicles) analysis is not a mapping of the genome. The microRNA profile is a snapshot of microRNA activity at the specific time of the blood sampling. Levels of microRNA are changing constantly and with the current knowledge on the topic it is impossible to predict a risk of developing specific diseases in the future. Cryotubes not used to microRNA/exosome analysis will be stored as part of a biobank.

Extracellular vesicle analysis:

Extracellular vesicles (EVs, also known as exosomes) will be isolated from plasma samples before characterization of surface markers and content. EVs are isolated by a number of different techniques, ultra centrifugation, precipitation, size exclusion chromatography among others. While protein characterization will be done using classical molecular biological techniques such as ELISA and Western blots in addition to array techniques, EVarray that utilises a panel of antibodies directed against known EV surface markers. These analyses might be supported by proteomic analysis of all proteins as well as post-translational modifications such as phosphorylations and glycosylations. To broaden the feasibility of finding stroke type specific EV surface markers, we will utilise recombinant antibody library techniques to find novel disease binders with the potential of diagnosing stroke types in blood samples.
In addition, the nucleic acid (DNA and RNA including miRNA) content of EVs will be analysed using next generation sequencing (NGS), qRT-PCR, and other nucleic acid detection techniques. Both the protein data and the nucleic acid data will be subjected to bioinformatic analysis using different pipelines and analysis tools depending on the dataset and the purpose of the analysis. A number of validated published databases

will be used for annotation and comparison. EVs do not contain genomic DNA and the analysis are therefore not a mapping of the subjects genome. As EV secretion and nucleic acid content in these are though to change rapidly in the body due to external and internal signals and are considered a snapshot at the specific time of blood sampling. With the current knowledge on the topic it is impossible to predict a risk of developing specific diseases in the future.

Coagulation assays:
Functional and immunologic plasma assays will be employed to analyze proteins and pathways in coagulation and fibrinolysis. The analyses will be performed in the accredited clinical laboratory and the thrombosis and hemostasis research unit at the Department of Clinical Biochemistry, Aarhus University Hospital. Plasma samples will be stored in cryotubes at -80 °C until they will be analyzed in large batches.

Ektacytometry for Erythrocytic Deformability:
The rheological properties (deformability, critical shear stress and aggregation) of RBCs are quantified using a Deformability or Elongation Index (DI or EI). A higher EI at the optimum viscosity (300 Osmolality) indicates highly deformable RBCs indicative of better microcirculation, while a lower EI indicates rigid and fragile RBCs resulting into impaired microcirculation and tissue hypoxia. Briefly, 6-uL of heparinized fresh blood is mixed with 600-uL of PVP solution (300 Osm) and transferred to a disposable kit (RheoMeditech, South Korea). The kit is placed inside the laser-assisted ektacytometer (RheoScan AnD300, RheoMeditech, Seoul, S. Korea) for automated read out, data and image collection as per the vendor's instructions.

Analytical Flow Cytometry (FC) for eryNOS3 phosphorylation (pNOS3Ser1177), APMKα1 phosphorylation and s-nitrosylation (–SNO) in RBCs:
Red blood cells are separated from 100-300-uL of freshly fixed blood samples, using a cocktail of antibodies (mAb) to RBC-specific markers (Glycophorin A, GPA (NovusBio, USA; and Hemoglobin, Hb; Bioss Biotech, USA). To assess the functional features, after fixation and permeabilization, RBCs are incubated with antibodies conjugated to fluorochrome either directly or through secondary antibodies to AMPKα1 phosphorylation (Bioss Biotech, USA) pNOS3Ser1177 (Bioss Biotech, USA) with or without RBC s-nitrosocysteine antibodies. Platelet and leucocyte specific surface antibody markers may be used. Next, RBC samples are run through a flow cytometer (CytoFLEX S Flowcytometer (Cytoflex S (B75408)), Beckman Coulter, USA), and data is collected using CellQuest software to process for FC analysis.
First, a human RBC flowcytometry protocol will be developed and validated against western blot. Second, titration studies will be performed on all specific antibodies to be used in the study. If the human RBC flowcytometry protocol cannot be developed and validated, western blot analysis of target protein phosphorylations will be performed.
Changes in specific details (vendors, dosing, different target antibody, procedures) may be due to change depending on the validation process.

Nitrite
Plasma nitrite will be measured by ozone-chemiluminesence (NOA 280i). These samples are processed, frozen and measured at a later time point. As soon as possible the Lithium Heparin treated samples are stabilized with Zysense®Nitrite Stabilizer Serum. Samples will be batch analysed at Regional Hospital West Jutland, Holstebro.

Biomarker workflow:

The biomarker study will upon initialization only preparing samples for the freezer will be performed and after that 2) acute ektacytometry (RBC deformability) of fresh samples will be performed in addition. Finally, 3) acute preparation of samples for flowcytometry will be added. This "three-tier" approach will be performed to ensure high quality of the handling and preparation of all samples

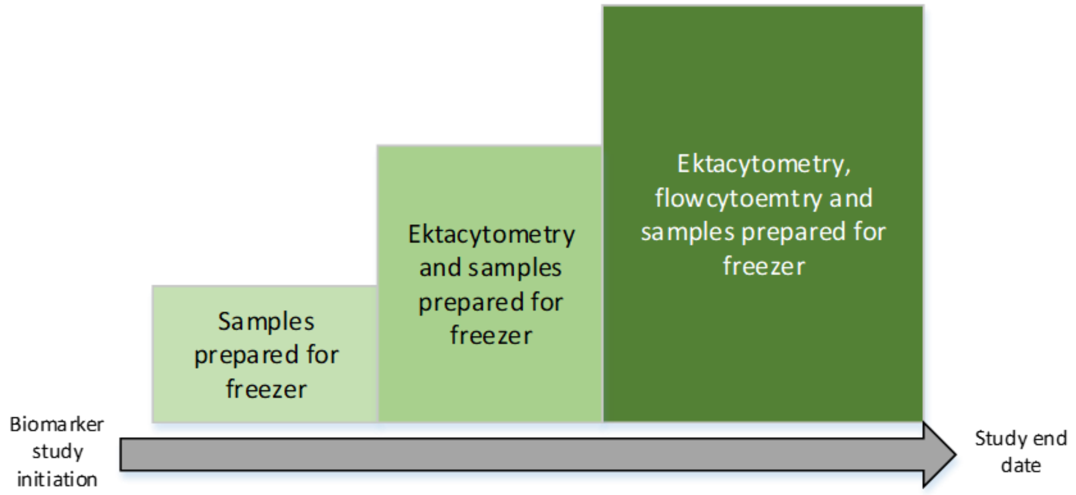

Prestroke physical activity measures in AIS and ICH patients

(Substudy only at Aarhus University Hospital)

Patient and their relatives will be asked to complete the physical activity in the elderly (PASE) questionnaire during the acute hospital admission. In patients who are unable to complete it themselves an structured interview based on the PASE questionnaire will be performed[41,42]
The PASE is a 12-item questionnaire, which quantifies the amount of PA over a 7-day period. The PASE questionnaire was developed with the purpose of assessing the level of PA in middle-aged and elderly individuals. The PASE score is calculated by taking the average number of hours spent on an activity (sports, occupational activity, household activities, and leisure time activities) per day over a 7-day period multiplied by an activity coefficient. Item scores are added to reveal the PASE score. The PASE score may range from zero to more than 400.

One week blood-pressure reduction in AIS and ICH patients

(Substudy at Aarhus University Hospital)

To determine whether RIC can reduce blood pressure (Systolic, diastolic, mean arterial pressure (MAP) and pulse) levels during one week of treatment.

Reduction in BP (baseline measurement (first) versus 7 day measurement (last)):

$$\Delta BP = BP_{baseline} - BP_{7\ Days}$$

9. Benefit of the study

Potential benefits:

Participating patients with AIS and ICH may experience an immediate reduction of their neurological symptoms and a persistent reduction of disability at long-term follow-up.

Disadvantage:

Mild-to-moderate pain and petechiae in the RIC-treated arm may occur during the inflation of the blood pressure cuff. Otherwise, the RIC treatment has been proven safe and without side effects.

Sham-RIC will only be associated with a slight sensation of pressure on the upper extremity.

Blood samples (only patients transported to and admitted at Aarhus University Hospital) of approximately 67mL (19mL prehospital and 2x21,5+1x5mL mL in-hospital) will be drawn.

An additional (compared to standard operating procedure) head CT will be performed at 7 days in ICH patients included at Aarhus University Hospital (If possible, the scan will be performed at the hospital were the patient is admitted at day 7). The estimated added whole-body radiation dose of the 7 days CT-scan is on average 1,5 mSv (range 1,05 to 1,95) corresponding to 6 months of accumulated background radiation in Denmark (Danish Health Authority). This corresponds to an estimated increased life-time cancer risk of 0.005%.

10. Assessment of safety

Emergency unblinding procedure

All on call neurologists will have access to electronic case report form using a personal and password protected login to perform randomization of study participants. In cases where emergency unblinding is necessary the on call neurologist will logon to electronic CRF at ddsc.dk and enter the civil registration number (CPR) of the patient. The treatment can now be unblinded. All changes will leave an audit-trail.

Adverse events

Patients are monitored in the stroke unit with NIHSS scoring and Scandinavian Stroke Scale (SSS) at close intervals, and adverse events are treated according to clinical guidelines.

The Data Monitoring Committee will assess safety according to primary study endpoints, recurrent stroke, myocardial infarction and mortality.

Interim analysis of safety parameters (hematoma expansion at 24 hours and mortality) in ICH will be performed regularly during the study.

During the acute in-hospital phase the patients will be asked if they have experienced any deterioration of health or new symptoms during or after treatment with the investigational device. Furthermore all patients will have a 24/7 contact number to the stroke center and a contact to study research physician and will be instructed to report any deterioration of health or new symptoms. All discharged patients (and relatives) will be given contact details to study personnel and be instructed to report any deterioration of health or new symptoms. The patients with acute ischemic stroke and intracerebral hemorrhage will once again be asked for adverse events at the 3 month telephone interview. In this group, end-of-study visit is the last

telephone interview, whereas end-of-study visit for participants without a vascular diagnosis or TIA(without DWI lesion) is at the time of discharge from the stroke center.

All patients, who have consented, are followed (through Danish Health Registries, LPR and DSR) for new vascular events and mortality for up to 12 months.

Adverse events and adverse device events are defined according to ISO 14155:2011 and European Commission guideline on medical devices (MEDDEV 2.7/3 revision 3).

Adverse event (AE):

Any untoward medical occurrence, unintended disease or injury or any untoward clinical signs (including an abnormal laboratory finding) in subjects, users or other persons whether or not related to the investigational medical device.

Serious Adverse Event (SAE)

Adverse event that:

a) led to a death, injury or permanent impairment to a body structure or a body function.

b) led to a serious deterioration in health of the subject, that either resulted in: - a life-threatening illness or injury, or - a permanent impairment of a body structure or a body function, or - in-patient hospitalization or prolongation of existing hospitalization, or - in medical or surgical intervention to prevent life threatening illness

Device Deficiency

Inadequacy of an investigational medical device related to its identity, quality, durability, reliability, safety or performance. This could be malfunctions, use error, or inadequacy in the information supplied by the manufacturer.

Adverse Device Effect

Adverse event related to the use of an investigational medical device.

This includes any adverse event resulting from insufficiencies or inadequacies in the instructions for use, the deployment, the implantation, the installation, the operation, or any malfunction of the investigational medical device.

This includes any event that is a result of a use error or intentional abnormal use of the investigational medical device.

Serious Adverse Device Effect (SADE)

Adverse device effect that has resulted in any of the consequences characteristic of a serious adverse event.

Unanticipated Serious Adverse Device Effect (USADE)

Serious adverse device effect which by its nature, incidence, severity or outcome has not been identified in the current version of the risk analysis report.

Anticipated SADE (ASADE): an effect which by its nature, incidence, severity or outcome has been previously identified in the risk analysis report

Event report and causality assessment

The relationship between the use of the medical device and the occurrence of each adverse event will be assessed and categorized. All events will be registered in the electronic CRF and reported to the authorities at the interim analysis. A yearly safety report, containing a list of all SAEs/SADEs and near-miss incidents, thorough evaluation of individual events, risk/benefit analysis of the investigational medical device and safety conclusions, will be submitted to the Danish Health and Medicines Authority and the Independent Ethics Committee by the Sponsor as required by Danish law.

Exceptions to and special considerations about reporting procedures are stated below:

Acute stroke is an acute life threatening disease with a high risk of neurological deterioration and mortality. The natural history of stroke is associated with a high risk of complications (stroke in progression, hemorrhagic transformation, dysphagia, cardiac arrhythmia, pneumonia, and other serious infections).

These *complications* all relates to the index stroke (occurring *before* randomization) and is foreseeable. If an event occurs that based on the assessment by the investigator is a result of the natural history of the disease it will not be reported by the sponsor to the Danish medicines agency and regional ethics committee within 7 days/2 days.

Furthermore, *complications* may also occur in patients without stroke and will often be related to the index disease (i.e severe infection/pneumonia). These complications (if forseeable as a result of the index disease) will not be reported will not be reported by the sponsor to the Danish medicines agency and regional ethics committee within 7 days/2 days. This includes a need for further investigations/surgery/admission based on the detailed examination at the stroke ward.

Any well described side effect/complication to medication/procedures performed during the acute admission will not be reported by the sponsor to the Danish medicines agency and regional ethics committee within 7 days/2 days.

For a list of foreseeable events/complications see table below.

All expected serious adverse events will be reported to the Danish Medicines Agency (and regional ethics committee) every 3 months.

All *new* serious events (Serious Adverse Events), complications that are not foreseeable or device near incidents or malpractice will be reported by the sponsor to the authorities (within 7 days, or 2 days if there is a risk of event reoccurrence) according to ISO 14155:2011 and European Commission guideline on medical devices (MEDDEV 2.7/3 revision 3). It includes all events not encompassed by the paragraphs listed above or the list of foreseeable events/complications below. Investigators will report the event to sponsor (within 24 hours). Sponsor will as fast as possible and no later than 7 days, or 2 days if there is a risk of event reoccurrence, report the event to the Danish Medicines Agency and regional ethics committee.

All Serious Adverse Device Effect (SADE) will be reported to the Danish Medicines Agency (and regional ethics committee) within 7 days, or 2 days if there is a risk of event reoccurrence

List of foreseeable adverse events/complications

Foreseeable events/complications that will not be reported by the sponsor to the Danish medicines agency and regional ethics committee within 7 days/2days.

Patient Population	Event	Frequency	Mitigation/treatment

**TIA and AIS**	Recurrent stroke.	10% at 90 days [a]	Standard medical treatment, intravenous thrombolysis/mechanical thrombectomy according to patient characteristics
**ALL**	Symptomatic intracerebral hemorrhage (+/- related to IV tPA or EVT)	7% [b]	Antithrombotic/anticoagulation therapy cessation. Antifibrinolytic agent. Bloodpressure control.
**AIS, ICH**	Early (<24hours) and late (>24hours) neurological deterioration<br>(ΔNIHSS ≥2 or death)	7-22% [c]	According to etiology
**AIS, ICH**	Aspiration pneumonia	5-20% [d]	Antibiotic treatment according to hospital SOP
**AIS, ICH**	Urinary tract infections	12% [e]	Antibiotic treatment according to local hospital SOP
**AIS, ICH**	Severe infections (sepsis)	13% [f]	Antibiotic treatment according to local hospital SOP
**AIS, ICH**	Cardiac arrhythmia	29% [g]	Depending on arrhythmia type. Treatment according to cardio.dk
**AIS, ICH**	Acute myocardial infarction	6% [h]	Treatment according to cardio.dk and conference call with cardiologist
**AIS, ICH**	Deep venous Thrombosis(DVT)/pulmonary embolism(PE)	DVT: 8% [i]<br>PE:1% [j]	Treatment according to cardio.dk
**AIS, ICH**	Falls (in-hospital phase)	14-65% [k, l]	Treatment according to type of injury
**AIS, ICH**	Seizure	AIS:3-10% [m]<br>ICH:8% [n]	Treatment according to local SOP
**AIS/ICH**	Mortality 30 days	AIS:10-20% [o]<br>ICH:30% [p]	
**AIS**	tPA induced angioedema	3% [q]	Treatment according to local SOP
**ALL**	Readmission	ALL: 13-18%[r]	Treatment according to diagnosis and local SOP
**ALL**	Infections in general after acute stroke	6-65% [s]	According to aetiology

a) Wang Y et al. Clopidogrel with Aspirin in Acute Minor Stroke or Transient Ischemic Attack. NEJM. 2013; 369:11-19
b) Berkhemer OA. A Randomized Trial of Intraarterial Treatment for Acute Ischemic Stroke. NEJM. 2015; 372:11-20
c) Kwan J et al. Early neurological deterioration in acute stroke: clinical characteristics and impact on outcome Journal of the Association of Physicians, 2006, Sept; 99:9: 625-633
d) Hannawi Y et al. Stroke-Associated Pneumonia: Major Advances and Obstacles. Cerebrovasc Dis. 2013;35:430-443

e) Bogason E. Urinary Tract Infections in Hospitalized Ischemic Stroke Patients: Source and Impact on Outcome. 2017;9(2):e1014. doi:10.7759/cureus.1014.

f) BergerB. Epidemiologic features, risk factors, and outcome of sepsis in stroke patients treated on a neurologic intensive care unit. Journal of Critical care. 2013;29(2)

g)Daniele O et al. Stroke and cardiac arrhythmias. J Stroke Cerebrovasc Dis. 2002 jan-feb;11(1):28-33

h) Johnston KC et al. Medical and neurological complications of ischemic stroke: experience from the RANTTAS trial. RANTTAS Investigators. Stroke. 1998;29(2):447
i) Bembenek J. Early stroke-related deep venous thrombosis: risk factors and influence on outcome. J thromb Thrombolysis. 2011 Jul; 32(1):96-102
j) Pongmoragot J. et al. Pulmonary Embolism in Ischemic Stroke: Clinical Presentation, Risk Factors, and Outcome. J Am Heart Ass. 2013 Dec;2(6)

k) Davenport RJ, Dennis MS, Wellwood I, Warlow CP. Complications after acute stroke. Stroke. 1996; 27: 415–420.
l) Teasell R, McRae M, Foley N, Bhardwaj A. The incidence and consequences of falls in stroke patients during inpatient rehabilitation: Factors associated with high risk. Arch Phys Med Rehabil. 2002; 83: 329–333.
m) Bladin C, Alexandrov AV, Bellavance A et al: Seizures After Stroke: A Prospective Multicenter Study.Arch Neurol. 2000;57(11):1617-1622.
n) Woo K-M, Yan S-Y, Cho K-T. Seizures after Spontaneous Intracerebral Hemorrhage. J. Korean Neurosurg Soc. 2012 Oct;52(4)312-319
O) Schwartz J et al. Incorporating Stroke Severity Into Hospital Measures of 30-Day Mortality After Ischemic Stroke Hospitalization. Stroke. 2017. Ahead of print: https://doi.org/10.1161/STROKEAHA.117.017960
p) Lichtman JH et al. 30-Day Mortality and Readmission after Hemorrhagic Stroke among Medicare.. Stroke. 2011.42(12)3387-91
q) Sczepanski M. et al. Institutional Incidence of Severe tPA-Induced Angioedema in Ischemic Cerebral Vascular Accidents. Crit Care Res Pract. 2018; 9360918
r) Zuckerman RB et al. Readmissions, Observation, and the Hospital Readmissions Reduction Program. N Engl J Med 2016; 374:1543-1551
s) Chamorro A. Infection After Acute Ischemic Stroke. Stroke. 2007;38:1097–1103

List of anticipated adverse device effects

RIC device	Frequency	Mitigation/treatment
**Local petechiae**	4-5% [a]	none
**Discomfort/pain**	20-30%	Treatment stopped if the non-competent patient verbally or non-verbally demonstrates intolerable discomfort or if the competent patient verbally want to discontinue the treatment
**Near events (device related)**	None known in the literature	
**Acute limb ischemia (upper extremity) – theoretical complication.**	None known in the literature	Treatment stopped. Treatment according local hospital SOP's for acute limb ischemia.<br>Known atherosclerotic stenosis in upper extremities are ***an exclusion criteria.***<br>See Investigators Brochure for further details.
**Sham RIC device**		
**None known**		

a) Hausenloy D et al. Remote Ischemic Preconditioning and Outcomes of Cardiac Surgery. New England J. Medicine. 2015 Oct;373(15):1408-17

Treatment and mitigation of any adverse events will be according regional guidelines.

Adverse events as a result of standard treatment will be handled according to local guidelines. Adverse events may also occur as a suspected reaction to RIC/Sham RIC – in these cases, the conditioning stimulus will be stopped immediately and appropriate treatment will be initiated.

In cases of SAE and device related near events all patients will be followed until resolution of symptoms/signs and/or all clinical relevant treatment have been performed and/or until the symptoms/signs are in a stable phase.

11. Project timetable and recruitment feasibility

Study preparation: January to February 2017

Study start date: March 2018

Month 0-60: Patient recruitment

Expected inclusion end date: 31. December 2022

Month 60-66: Study analysis

Expected end date for last analysis and follow-up: 31. December 2024

Patient inclusion per center:

Aarhus University Hospital: 50%

Regional Hospital West Jutland (Holstebro): 12,5%

Odense University Hospital: 12,5%

Aalborg University Hospital: 12,5%

Zealand University Hospital (Roskilde): 12,5%

12. Ethical considerations

The study will await approval from the Regional Ethical Committee, the Danish Health Authority, and the Danish Data Protection Agency. Enrollment of additional Danish stroke centers will require a new application to the Danish Medicines Agency and the regional ethics committee (amendment).

A large acute stroke results in immediate, major neurological deficits (+/-aphasia) and a substantially increased risk of a long-term reduction of functional capability. This group is assessed as legally incompetent. Even in patients suffering from minor stroke, acute cognitive impairment are common, making individual preference on acute treatment options impossible. In order to preserve brain tissue, the intervention must be applied without delay. Improvement of standard care in patients suffering from acute stroke is of paramount importance. Inclusion of all stroke patients is necessary in order to translate any positive research results into a benefit for all stroke patients. The intervention is without any known risk apart from moderate pain in the upper extremity when the cuff is inflated. We find that the conditions for an acute study are satisfied since the majority of the patients are incapacitated at stroke onset and the treatment has to be initiated as soon as possible and there are no reported serious side effects from RIC.

Study participants are covered in accordance with the Danish Patient Insurance Act.

13. Data handling and record keeping

All study data are recorded in an electronic CRF with blinded data and identification via a study identification number.

The study will apply to the specifications of act on processing of personal data.

The database(e-CRF) is handled by OPUS consult Aps, Egaa, Denmark, who already handles the Danish Stroke Center Database in Aarhus (ddsc.dk).

Results from the biomarker substudies will recorded and managed using REDCap electronic data capture tools hosted at Aarhus University Hospital.

Remote Ischemic Conditioning in Stroke (RISC) is a predefined collaboration in which three large RIC trials have agreed to share anonymized patient data for a large metaanalysis once the individual trials have ended (Principle Investigators of the individual trials are: Grethe Andersen (DK), Philip Bath (UK) and Fernando Pico (France).

Methodological experts from collaborators at Medical College Georgia, Augusta, USA (Dr. Hess' and Dr. Baban's lab) will remotely supervise the technical aspects of flowcytometry and ektacytometry and assist in the interpretation of results (only anonymized patient data). *Flowcytometry and ektacytometry results reflects only snapshots of the function and deformability of the red blood cells and change rapidly.*

Remote Ischemic Conditioning in Stroke (RISC) is a predefined collaboration in which three large RIC trials have agreed to share anonymized patient data for a large metaanalysis once the individual trials have ended (Principle Investigators of the individual trials are: Grethe Andersen (DK), Philip Bath (UK) and Fernando Pico (France).

Patient participation will be recorded in the medical record. Data will be stored at the Department of Neurology, Aarhus University Hospital, for 15 years, after which the documents will be shredded.

A notification to The Danish Data Protection agency will be submitted.

During the entire study period and at all participating centers the local GCP unit will perform quality assurance control including source data verification.

A complete list of all source data will be made and approved by the local GCP unit before study initiation.

The investigator permits direct access to all source data/documents (including electronic patient record) at monitoring visits, audits and/or inspections by the regional ethics committee and Danish Medicines Agency.

14. Publications policy

The results of the study, both negative, inconclusive and positive, will be disseminated as widely as possible - through publication in an international peer-reviewed journal, as conference presentations and on www.clinicaltrials.gov.

The trial is registered on ClinicalTrials.gov with Identifier: NCT03481777

15. Statistics

Primary study endpoint:

Difference in clinical outcome (mRS) at 3 months in acute ischemic stroke *(General ordinal logistic regression analysis. Significance level of 5%)*

The **main analysis of the primary study endpoint** will be performed using the entire range of the modified Rankin Scale **(General ordinal logistic regression, unadjusted)** on the population fulfilling the ***in-hospital inclusion criteria (target population)***. The trial success analysis is performed on the **target population** which consists of prehospital randomized patients with an in-hospital diagnosis of acute ischemic stroke (including TIA with a DWI lesion) and intracerebral hemorrhage (ICH) regardless of adherence to investigational treatment (**modified Intention-To-Treat analysis**).

Supplementary safety analysis:
All patients randomized in RESIST had pre-randomization a focal neurological deficit (documented on the prehospital stroke score). The presence or absence of specific focal neurological symptoms are assessed again after 24 hours using the same score as pre-randomization (PreSS) in all randomized patients (PreSS 24 hour/ at discharge). The primary safety analysis will be made on the **entire randomized population using the difference in prehospital stroke score** (PreSS) to document change in neurological deficits between pre-randomization and 24 hour/discharge **(General ordinal logistic regression, unadjusted)**. There has been no safety concern over RIC treatment in patients with non-vascular diagnosis[23,27,28,29,30]

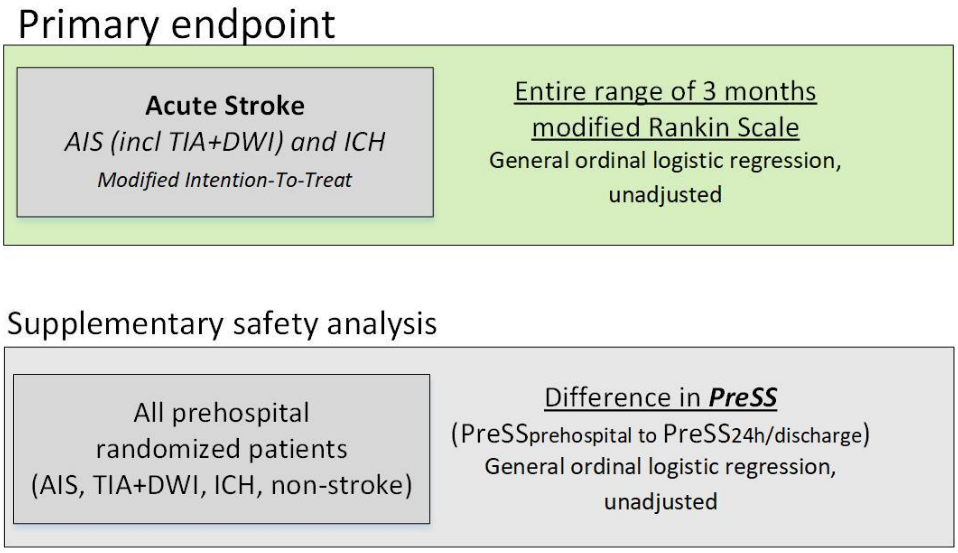

If remote ischemic preconditioning is associated with ultra-early treatment effect and reduced symptom severity (in the ambulances), this could affect the in-hospital indication for IV-tPA and/or EVT (in the case of rapid improvement of symptoms or mild symptoms)

Because of this, a **supplementary analyses** using **general ordinal logistic regression, adjusted for a possible** imbalances in reperfusion treatments (IV-tPA and EVT) between treatment arms will be made.

Secondary study endpoints (selected):

- Difference in prehospital stroke score (PreSS) (Supplementary safety endpoint, see above) (All randomized patients, general ordinal logistic regression, significans level 5%)
- Difference in prehospital stroke score (PreSS) during the first 24 hours: (general ordinal logistic regression analysis in the stroke subtypes, Significance level of 5%)
- Difference in early neurological improvement in AIS and ICH *(general ordinal logistic regression analysis. Significance level of 5%)*
- Difference in prehospital stroke score (PreSS) after 24 hours in subgroups: IV tPA/EVT treated AIS and ICH. (ordinal logistic regression analysis, significance level of 5%)
- Difference in acute hematoma expansion in patients with ICH (Binomial regression analysis, significance level of 5%)
- Difference in acute infarct growth in patients with AIS treated with IV-tPA and/or EVT (Binomial regression analysis, significance level of 5%)
- Major adverse cardiac and cerebral events (MACCE) and recurrent ischemic events at 3 and 12 months in AIS patients *(Cox regression analysis, significance level of 5%)*
- Three-month and one-year mortality *(Two sample test of proportion (Chi-square test), significance level of 5%)*
- EVT-eligibility (MRI assessed) in RIC treated AIS patients with large vessel occlusion (LVO) *(Binomial regression analysis. Significance level of 5%)*

Sample Size

The treatment effect of RIC on long-term functional outcome is unknown. We have assumed a small but clinical significant neuroprotective effect corresponding to a 7% increased odds for a beneficial shift on the modified Rankin Scale. The sample size calculation was based on a simulation-based approach to the analysis of statistical power when ordinal logistic regression analysis is performed (significans level of 5%)

The statistical power was simulated at different hypothetical sample sizes (on the target population) (ranging from 200 to 1900) with 2000 simulation-runs performed at each step. Unpublished data on IV-tPA and/or EVT treated AIS patients and ICH patients from our institution were used:

3 months modified Rankin Scale distribution (proportions) in 2017 for patients with ICH or IV tPA/EVT treated AIS at Aarhus University Hospital.

**Modfied Rankin Scale**	**mRS 0**	**mRS 1**	**mRS 2**	**mRS 3**	**mRS 4**	**mRS 5**	**mRS 6**
Proportion	0.139	0.273	0.141	0.110	0.145	0.07	0.126

Based on our previous trial experience with prehospital remote ischemic conditioning we estimate that a sample of 1000 subjects with target diagnosis (AIS and ICH) will be feasible to include during the study period.

Thus, including 1000 patients with target diagnosis provide sufficient power at a alfa-level (significans) of 5% to detect RIC treatment effects of the estimated 7 % *(see table below)*

**Treatment effect** (assumed neuroprective)	5%	6%	**7%**
Sample (target diagnosis), *n*	1000	1000	**1000**
Power	66%	80%	**90%**
Alfa level (significans level)	5%	5%	**5%**

The estimated prehospital, randomized, sample:

**Sample size, prehospital**	**Proportion of randomized**	***n =***
**Target diagnosis (AIS and ICH), *n***	**67%**	**1000**
Non-vascular diagnosis	**27%**	**403**
TIA without DWI lesion	**4%**	**60**
Lost to follow-up	**2%**	**30**
Total		**1492**
**Plan to include**		**1500**

We therefor plan to include 1500 patients with a prehospital putative stroke in order to get 1000 patients with the target diagnosis of acute ischemic stroke and intracerebral hemorrhage. There is no planned replacement of patients lost to follow-up.

Interim analysis

We will perform an interim analysis for evaluation of the actual event rate after inclusion of 50% of the patients. If this shows a lower good outcome rate than expected, a new sample size calculation will be performed. Furthermore, the Data monitoring committee (DMC) will perform an independent safety analysis and will review the overall safety and efficacy according to the DMC charter (planned in the early phase of the trial, at 12 month and hereafter at least every 12. months) An additional safety interim analysis of mortality and hematoma expansion in ICH patients will be performed. Interim analysis are assessed using a level of significance of 1%.

The trial can be stopped at interim analyses for both futility, efficacy and safety reasons.

Detailed description is found in the DMC charter.

Missing data:

Missing data with be handled using multiple imputation (predictive mean matching imputation).

Deviations from the statistical plan:

All deviations from the statistical plan will first be applied when approved by the regional ethics committee and the Danish Medicines Agency.

Trial decision making and stopping rules

A Data Monitoring Committee (DMC) will be appointed before trial start and include a signed DMC charter. The DMC will review the accumulated data during RESIST trial and provide advice on the conduct of the trial to the Steering Committee.

The DMC should inform the Steering Committee
if, in their view:

1. the results are likely to provide convincing evidence that one trial arm is clearly indicated or contraindicated, and there is a reasonable expectation that this new evidence would materially influence patient management

or

2. it is beyond doubt that no clear outcome would be obtained (futility)

It is not expected that RESIST trial will be stopped for reasons of efficacy or futility based on observed treatment differences. A predefined pooling of individual patient data (Remote Ischemic conditioning in Stroke Collaboration, RISC) is planned. To dispel scepticism, a robust demonstration of efficacy of this simple intervention is required. The primary reason to recommend stopping the trial will be for safety reasons.

For Acute Ischemic Stroke and Intracerebral Hemorrhage:

- For the primary endpoint the DMC will consider stopping if there is a robust improvement in functional outcome after 3 months (modified Rankin Scale) for Acute Ischemic Stroke and Intracerebral haemorrhage in the sham (control) group compared to the intervention (RIC) group, achieving $p<0.01$.
- A substantial number of patients experience serious adverse events ($p<0.01$)
- DMC will consider stopping if there is an increase in mortality or stroke recurrence for Acute Ischemic Stroke and Intracerebral Hemorrhage patients in the intervention (RIC) group compared to the control (sham) group, achieving $p<0.01$.
- For ICH the DMC will consider stopping/modification if there is a clear evidence of hematoma growth during the first 24-hour in the intervention group, achieving $p<0.01$

The DMC will make recommendations, which could include:

- No action needed, trial continues as planned
- Early stopping due, for example, to clear benefit or harm of a treatment, futility, or external evidence
- Stopping recruitment within a subgroup

- Extending recruitment (based on actual control arm response rates being different to predicted other than on emerging differences) or extending follow-up
- Approving and/or proposing protocol changes

16. Source data access and monitoring

Trial-related audits and/or monitoring will be provided by direct access to source data/documents. Local monitors from the Unit for Good Clinical Practice, Aarhus University, will perform the audit and monitoring. Audit and monitoring will, likewise, be perfomed by qualified local GCP-monitors when enrollment at other stroke centers in Denmark is starting. Before enrollment can start a trial monitoring plan must be available. The audit and monitoring process will involve a 100% monitoring of signed consent forms ("samtykkeerklæringer og fuldmagtserklæringer") and serious adverse events.

17. Economy

Study initiator is Grethe Andersen, Senior Consultant, MD, DMSc, Professor of Neurology at Department of Neurology, Aarhus University Hospital.

The Danish foundation Vilhelm Petersens mindelegat has supported the study with 1.240.000 dkr (research nurse salary and "stroke RIC" devices).

The Danish foundation TrygFonden has supported the study with 4.000.000 dkr (research personnel salaries, running costs, micro RNA analysis and investigational devices).

The Danish foundation Novo Nordisk Foundation has supported the study with 2.562.153dkr (Salary for Research nurses, technicians and analysis costs for ektacytometry and flowcytometry)

Aarhus University has supported the study with 550.000 dkr (study investigator/coordinator salary)

The funders have no role in study design, data collection, analysis or interpretation, nor the decision to publish or the preparation, review and approval of the manuscript.

Stroke RIC/Sham-RIC device is developed in collaboration with Aarhus University, Faculty of Biomedical Engineering, 8200-DK, Aarhus N, Denmark; Seagull Aps, 4160-DK, Herlufmagle, Denmark and the Department of Neurology, Aarhus University Hospital, 8000-DK, Aarhus C, Denmark. Seagull Aps has no role in study design, data collection, analysis or interpretation, nor the decision to publish or the preparation, review and approval of the manuscript.

The research grants are transferred directly to a research account administered by the financial department of Aarhus University Hospital. None of the involved doctors or research nurses has any conflict of interest or economic advantages in regards to the study. There is no economic compensation or reimbursement for patients participating in the study.

Supplements

Supplement A - Neuroimaging protocol

On admission, either CT or MRI is performed according to hospital SOP.

All AIS and ICH patients can be included in the in-hospital RIC/sham-RIC based on a baseline native CT. However, patients with complete remission of symptoms after randomization (TIA) require an MRI at baseline demonstrating acute ischemic lesion on DWI in order to continue the randomized treatment. TIA patients with a documented ischemic lesion are, per definition, handled as ischemic stroke in this study. Baseline MRI in TIA patients is performed before the next RIC series (MRI <6 hours from RIC/sham-RIC last cuff). Patients with AIS who are not eligible for IV tPA due to contraindications will have CT/MRI before their next RIC/sham-RIC series (< 6 hours from RIPerC last cuff) according to hospital SOP. All ICH patients will have a baseline and 24-hour CT (hospital SOP)

Non-stroke diagnosis and TIA (only evaluated with CT or TIA without DWI lesion on MRI) will not be included in the in-hospital RIC study and will receive no further RIC treatment or neuroimaging.

**Imaging protocol**	**Accepted time frame**
MRI baseline – AIS IV tPA eligible	Acute on admission
CT baseline – AIS IV tPA eligible	Acute on admission
MRI/CT baseline – AIS non IV tPA eligible	< 6 hours from last RIC cuff
MRI (24-hour) - tPA/EVT treated AIS patients*	18-30 hour from baseline MRI
MRI baseline – ICH	Perform CT < 3 hours from last RIC cuff, if possible
CT baseline – ICH	< 6 hours from last RIC cuff
Control CT (24-hour) – ICH*	18-30 hour from baseline CT
Control CT (7-day)*	5-9 days from baseline CT

*Substudy at Aarhus University Hospital

Computed tomography (CT) protocol
Baseline CT and CT angiography are obtained in centers using CT for primary assessment (according to hospital SOP).

CT analysis

Hematoma volume assessment at baseline and at 24 hours and 7 days is performed by an experienced neuroradiologist. The assessment will be assisted using an automated stroke volume assessment software (e.g. COMBAT stroke APS, Aarhus University Hospital, Denmark) or using the formula *ABC*/2 where *A* is the greatest hematoma diameter by CT, *B* is the diameter perpendicular to *A*, and *C* is the number of CT slices with hematoma multiplied by slice thickness[43].

Magnetic resonance imaging (MRI) protocol
Diffusion-weighted imaging (DWI), apparent diffusion coefficient (ADC), T2* gradient-recalled echo (T2*GRE), and T2 fluid-attenuated inverse recovery (T2-FLAIR). MR angiography time-of-flight (MRA-TOF) is performed according to hospital standard operating procedures (SOP).

The 24-hour MRI is performed on the same MRI scanner used at baseline, if possible. This protocol includes: DWI, T2*, T2 and T2-FLAIR. MRA-TOF at 24 hours is obtained if the baseline CT-angiography or MRA-TOF demonstrated vessel occlusion.

The total acquisition time for this protocol is approximately 7-10 mins (MRI)/10-15 min (MRI + MRA-TOF), depending on the MRI equipment.

MRI analysis
Acute (baseline) and 24-hour follow-up (DWI lesion will be outlined, representing irreversibly damaged tissue *(substudy at Aarhus University Hospital).*

Infarct growth is defined as the difference between baseline DWI and the 24-hour MRI-DWI lesion.

Infarct volume assessment at baseline and at 24 hours is performed by an experienced neuroradiologist. The assessment will be assisted using an automated stroke volume assessment software (e.g. COMBAT stroke APS, Aarhus University Hospital, Denmark) or using the formula $ABC/2$ where A is the greatest hematoma diameter by CT, B is the diameter perpendicular to A, and C is the number of CT slices with hematoma multiplied by slice thickness[43]

Vascular patency and reperfusion are assessed using the Thrombolysis in Cerebral Infarction Perfusion Scale (TICI) criteria[44]

- No perfusion (TICI 0)
- Perfusion past the initial occlusion, but no distal branch filling (TICI 1)
- Perfusion with incomplete or slow distal branch filling (TICI 2)
- Full perfusion with filling of all distal branches, including M3 and M4 (TICI 3)

Supplement B – Baseline data

Age	Platelet inhibitor treatment
Male/female	Anticoagulation therapy
**Medical History**	New oral anticoagulation treatment (NOAC)
Hypertension	Opioid treatment
Smoking	SSRI treatment
Alcohol	**Clinical and physiological data**
Hyperlipidemia	Prehospital Stroke Score (PreSS)
Diabetes	Modified Rankin Scale prestroke
Previous myocardial infarction<br>*Yes: within 3 months, yes: more than 3 months, no, unknown*	NIHSS (baseline)
Angina pectoris	Symptom onset (time)
Recent angina pectoris (< 4 weeks)<br>*If yes, date for latest episode*	Time of admission
Atrial fibrillation	Completed prehospital RIC/sham cycles
Previous ischemic stroke	= 5, 4, 3, 1-2, 0
Stroke < 3 month<br>*If yes, date*	Time (start/stop) for each RIC/sham cycle
Previous TIA	NIHSS (t = 24 hours)
Recent TIA < 4 weeks<br>*If yes, date for latest episode*	Stroke etiology (TOAST)
Previous ICH	Admission blood pressure
Peripheral artery disease	**IV tPA/EVT treated AIS**
Physical activity (PASE interview)<br>Substudy at Aarhus University Hospital	Treatment initiation (time)
WHO-5<br>Substudy at Aarhus University	**Quality indicators (Danish Stroke Registry)**
**Medication on admission**	**Routine lab results***<br>Substudy at Aarhus University Hospital
Statins	
Calcium channel blockers	
ACE inhibitors/angiotensin receptor blockers	
Beta-blockers	

Baseline data obtained from treating physician, database of Danish stroke center (ddsc.dk), Danish stroke registry and electronic health records.

**Routine lab results at Aarhus University Hospital (See Biochemical section page 26-27 for details)*

References

1. Astrup J, Siesjo BK, Symon L. Thresholds in cerebral ischemia - the ischemic penumbra. *Stroke*. 1981;12(6):723-725.
2. Group N study. Tissue plasminogen activator for acute ischemic stroke. *N Engl J Med*. 1995;333(24).
3. Hacke W, Kaste M, Bluhmki E. Thrombolysis with alteplase 3 to 4.5 hours after acute ischemic stroke. *… Engl J …*. 2008:1317-1330. http://www.nejm.org/doi/full/10.1056/NEJMoa0804656. Accessed October 21, 2014.
4. Berkhemer OA, Fransen PSS, Beumer D, et al. A randomized trial of intraarterial treatment for acute ischemic stroke. *N Engl J Med*. 2015;372(1):11-20. doi:10.1056/NEJMoa1411587
5. Touma L, Filion KB, Sterling LH, Atallah R, Windle SB, Eisenberg MJ. Stent Retrievers for the Treatment of Acute Ischemic Stroke: A Systematic Review and Meta-analysis of Randomized Clinical Trials. *JAMA Neurol*. January 2016. doi:10.1001/jamaneurol.2015.4441
6. Prior A, Laursen TM, Larsen KK, et al. Post-Stroke Mortality , Stroke Severity , and Preadmission Antipsychotic Medicine Use – A Population-Based Cohort Study. 2014;9(1). doi:10.1371/journal.pone.0084103
7. Ren C, Gao M, Dornbos D, et al. Remote ischemic post-conditioning reduced brain damage in experimental ischemia/reperfusion injury. *Neurol Res*. 2011;33(5):514-519. doi:10.1179/016164111X13007856084241
8. Hougaard KD, Hjort N, Zeidler D, et al. Remote ischemic perconditioning in thrombolysed stroke patients: randomized study of activating endogenous neuroprotection - design and MRI measurements. *Int J Stroke*. 2013;8(2):141-146. doi:10.1111/j.1747-4949.2012.00786.x
9. Khatri R, McKinney AM, Swenson B, Janardhan V. Blood-brain barrier, reperfusion injury, and hemorrhagic transformation in acute ischemic stroke. *Neurology*. 2012;79(13 Suppl 1):S52-7. doi:10.1212/WNL.0b013e3182697e70
10. Campbell BC V, Christensen S, Tress BM, et al. Failure of collateral blood flow is associated with infarct growth in ischemic stroke. *J Cereb Blood Flow Metab*. 2013;33(8):1168-1172. doi:10.1038/jcbfm.2013.77
11. Ip HL, Liebeskind DS. The future of ischemic stroke: flow from prehospital neuroprotection to definitive reperfusion. *Interv Neurol*. 2014;2(3):105-117. doi:10.1159/000357164
12. Giles MF, Albers GW, Kim AS, Sciolla R. Early stroke risk and ABCD2 score performance in tissue- vs time-defined TIA A multicenter study. *Neurology*. 2011.
13. Kellner CP, Connolly ES. Neuroprotective Strategies for Intracerebral Hemorrhage Trials and Translation. 2010. doi:10.1161/STROKEAHA.110.597476
14. Anderson CS, Heeley E, Huang Y, et al. Rapid blood-pressure lowering in patients with acute intracerebral hemorrhage. *N Engl J Med*. 2013;368(25):2355-2365. doi:10.1056/NEJMoa1214609
15. Murry CE, Jennings RB, Reimer KA. Preconditioning with ischemia: a delay of lethal cell injury in ischemic myocardium. *Circulation*. 1986;74(5):1124-1136.
16. Hahn CD, Manlhiot C, Schmidt MR, Nielsen TT, Redington AN. Remote ischemic per-conditioning: a novel therapy for acute stroke? *Stroke*. 2011;42(10):2960-2962. doi:10.1161/STROKEAHA.111.622340
17. Hess DC, Hoda MN, Bhatia K. Remote limb perconditioning [corrected] and postconditioning: will it

translate into a promising treatment for acute stroke? *Stroke*. 2013;44(4):1191-1197. doi:10.1161/STROKEAHA.112.678482

18. Schmidt MR, Pryds K, Bøtker HE. Novel adjunctive treatments of myocardial infarction. *World J Cardiol*. 2014;6(6):434-443. doi:10.4330/wjc.v6.i6.434
19. Li J, Rohailla S, Gelber N, et al. MicroRNA-144 is a circulating effector of remote ischemic preconditioning. *Basic Res Cardiol*. 2014;109(5):423. doi:10.1007/s00395-014-0423-z
20. Schmidt MR, Redington A, Bøtker HE. Remote conditioning the heart overview - translatability and mechanism. *Br J Pharmacol*. September 2014:1-37. doi:10.1111/bph.12933
21. Hoda MN, Bhatia K, Hafez SS, et al. Remote ischemic perconditioning is effective after embolic stroke in ovariectomized female mice. *Transl Stroke Res*. 2014;5(4):484-490. doi:10.1007/s12975-013-0318-6
22. Lim SY, Hausenloy DJ. Remote ischemic conditioning: from bench to bedside. *Front Physiol*. 2012;3(February):27. doi:10.3389/fphys.2012.00027
23. Hougaard KD, Hjort N, Zeidler D, et al. Remote ischemic perconditioning as an adjunct therapy to thrombolysis in patients with acute ischemic stroke: a randomized trial. *Stroke*. 2014;45(1):159-167. doi:10.1161/STROKEAHA.113.001346
24. Meng R, Asmaro K, Meng L, et al. Upper limb ischemic preconditioning prevents recurrent stroke in intracranial arterial stenosis. *Neurology*. 2012;79(18):1853-1861. doi:10.1212/WNL.0b013e318271f76a
25. Meng R, Ding Y, Asmaro K, et al. Ischemic Conditioning Is Safe and Effective for Octo- and Nonagenarians in Stroke Prevention and Treatment. *Neurotherapeutics*. 2015;12(3):667-677. doi:10.1007/s13311-015-0358-6
26. Vaibhav K, Braun M, Khan MB, et al. Remote Ischemic post-conditioning promotes hematoma resolution via AMPK-dependent immune regulation. Journal of experimental medicine. 2018. 6 sept (online). doi.10.1084/jem.20171905
27. Le Page S, Bejan-Angoulvant T, Angoulvant D, Prunier F. Remote ischemic conditioning and cardioprotection: a systematic review and meta-analysis of randomized clinical trials. *Basic Res Cardiol*. 2015;110(2):11. doi:10.1007/s00395-015-0467-8
28. Bøtker HE, Kharbanda R, Schmidt MR, et al. Remote ischaemic conditioning before hospital admission, as a complement to angioplasty, and effect on myocardial salvage in patients with acute myocardial infarction: a randomised trial. *Lancet*. 2010;375(9716):727-734. doi:10.1016/S0140-6736(09)62001-8
29. Hausenloy DJ, Candilio L, Evans R, et al. Remote Ischemic Preconditioning and Outcomes of Cardiac Surgery. *N Engl J Med*. 2015;373(15):1408-1417. doi:10.1056/NEJMoa1413534
30. Laiwalla AN, Ooi YC, Liou R, Gonzalez NR. Matched Cohort Analysis of the Effects of Limb Remote Ischemic Conditioning in Patients with Aneurysmal Subarachnoid Hemorrhage. *Transl Stroke Res*. 2016;7(1):42-48. doi:10.1007/s12975-015-0437-3
31. Hess DC, Blauenfeldt RA, Andersen G, et al. Remote ischaemic conditioning-a new paradigm of self-protection in the brain. *Nat Rev Neurol*. 2015;11(12):698-710. doi:10.1038/nrneurol.2015.223
32. Hastrup S, Damgaard D, Johnsen SP, Andersen G. Prehospital Acute Stroke Severity Scale to Predict Large Artery Occlusion: Design and Comparison With Other Scales. *Stroke*. 2016;47(7):1772-1776. doi:10.1161/STROKEAHA.115.012482
33. Kothari RU, Pancioli A, Liu T, Brott T, Broderick J. Cincinnati Prehospital Stroke Scale: reproducibility

and validity. *Ann Emerg Med*. 1999;33(4):373-378.

34. Sivakumar L, Kate M, Jeerakathil T, Camicioli R, Buck B, Butcher K. Serial montreal cognitive assessments demonstrate reversible cognitive impairment in patients with acute transient ischemic attack and minor stroke. *Stroke*. 2014;45(6):1709-1715. doi:10.1161/STROKEAHA.114.004726

35. Riepe MW, Riss S, Bittner D, Huber R. Screening for cognitive impairment in patients with acute stroke. *Dement Geriatr Cogn Disord*. 2004;17(1-2):49-53. doi:10.1159/000074082

36. Bruno A, Akinwuntan AE, Lin C, et al. Simplified modified rankin scale questionnaire: reproducibility over the telephone and validation with quality of life. *Stroke*. 2011;42(8):2276-2279. doi:10.1161/STROKEAHA.111.613273

37. Guillemin F, Bombardier C, Beaton D. Cross-cultural adaptation of health-related quality of life measures: literature review and proposed guidelines. *J Clin Epidemiol*. 1993;46(12):1417-1432.

38. Beaton DE, Bombardier C, Guillemin F, Ferraz MB. Guidelines for the process of cross-cultural adaptation of self-report measures. *Spine (Phila Pa 1976)*. 2000;25(24):3186-3191.

39. Demchuk AM, Dowlatshahi D, Rodriguez-Luna D, et al. Prediction of haematoma growth and outcome in patients with intracerebral haemorrhage using the CT-angiography spot sign (PREDICT): a prospective observational study. *Lancet Neurol*. 2012;11(4):307-314. doi:10.1016/S1474-4422(12)70038-8

40. Kappetein AP, Head SJ, Genereux P, et al. Updated standardized endpoint definitions for transcatheter aortic valve implantation: the Valve Academic Research Consortium-2 consensus document. *Eur Heart J*. 2012;33(19):2403-2418. doi:10.1093/eurheartj/ehs255

41. Schuit AJ, Schouten EG, Westerterp KR, Saris WH. Validity of the Physical Activity Scale for the Elderly (PASE): according to energy expenditure assessed by the doubly labeled water method. *J Clin Epidemiol*. 1997;50(5):541-546.

42. Washburn RA, McAuley E, Katula J, Mihalko SL, Boileau RA. The physical activity scale for the elderly (PASE): evidence for validity. *J Clin Epidemiol*. 1999;52(7):643-651.

43. Kothari RU, Brott T, Broderick JP, et al. The ABCs of measuring intracerebral hemorrhage volumes. *Stroke*. 1996;27(8):1304-1305.

44. Khatri P, Neff J, Broderick JP, Khoury JC, Carrozzella J, Tomsick T. Revascularization end points in stroke interventional trials: recanalization versus reperfusion in IMS-I. *Stroke*. 2005;36(11):2400-2403. doi:10.1161/01.STR.0000185698.45720.58

2. Summary of Changes to the Protocol

Three protocol amendments have been approved during the course of the study:

Summary of Changes to the Protocol as approved by the Danish Medicines Agency

1. Rationale for adjustments: From protocol Febr 9, 2018 to Nov 29, 2018

Major changes	Details
Primary endpoint change:<br>**From**<br><br>Difference in prehospital stroke score (PreSS) during the first 24 hours:<br>ΔPreSS = PreSSprehospital – PreSS24-hours (Ordinal logistic regression analysis in the per-protocol population. Significance level of 5%)<br><br>**To**<br><br>**Difference in clinical outcome (mRS) at 3 months in acute ischemic stroke (ordinal logistic regression analysis. Significance level of 5%)**<br><br><br><br><br>Sample size recalculated based on change in primary endpoint	Background:<br>1) The original concern was that the majority of patients would score 0-2 on the modified Rankin Scale (mRS), but have a more even distribution (on the entire range) on ordinal use of PreSS. Blinded assessment of the distribution PreSS and mRS scores showed similar overall distribution and variance in patients included until 27th of August 2018.<br>2) A consensus statement on prehospital stroke trials recommended using mRS as primary study endpoint for all stroke trials.<br>3) Although items fra PreSS was known from other prehospital stroke scores and NIHSS, PreSS itself had not been validated at that particular time.<br>4) Predefined planned individual patient data meta-analysis with other international groups will use mRS as primary endpoint.<br><br>The recommendation was made by senior trial statistician, epidemiologist, sponsor Investigator GA and the trial steering committee.<br><br>A new sample size calculation was performed.<br>Sample size was changed from 2500 prehospital putative stroke patients (1380 target diagnosis of AIS) to 1500 (1000 with a target diagnosis of AIS and ICH) and was now based on a simulation-based approach to the analysis of statistical power when

	ordinal logistic regression analysis is performed (significance level of 5%)<br>The statistical power was simulated at different hypothetical sample sizes (on the target population) (ranging from 200 to 1900) with 2000 simulation-runs performed at each step.
**Secondary endpoints on biochemical analysis added (only Aarhus University Hospital)**	• Coagulation profile of putative stroke patients in prehospital obtained blood samples (*substudy at Aarhus University Hospital*)<br>• Modulation of coagulation by RIC (*substudy at Aarhus University Hospital*)<br>• Characterization of Red blood cell rheo-erythrocrine dysfunction in acute stroke and its possible association to improved short term (24 hour) or long term (90 day mRS) clinical outcome or imaging biomarkers (DWI infarct growth)<br>• Characterization of rheoerythrocrine dysfunction (RBC deformability, increases activated eryNOS3) and/or increases plasma nitrite in RIC vs Sham-RIC treated AIS patients
**24-hour blood samples (only Aarhus University Hospital)**	• 24-hour blood-samples added. Stroke biomarkers in prehospital acute phase for diagnosing and triaging stroke patients
**24 hour MRI only in IV-tPA and EVT treated AIS patients with a baseline MRI (only Aarhus University Hospital)**	• 24-hour MRI in patients not receiving reperfusions therapy will not be performed as the relevance is uncertain.<br>• 24-hour MRI in patients treated with reperfusion therapy will still be performed.
**7 days CT added (only AUH)**	• New secondary endpoint added: Difference in hematoma reabsorption rate (7 days) in patients with ICH.<br>New results from preclinical studies suggests increased hematoma reabsorption rate during the first 7 days after ICH.

1. Rationale for Adjustments: From protocol Nov 29, 2018 to Aug 29, 2019

Major changes	Details
**Major changes**	Consent forms and study information divided in forms/information for Aarhus site and other sites because of blood-samples/biomarker sub study at the Aarhus site

2. Rationale for Adjustments: from protocol August 29, 2019 to March 31, 2020

Major changes	Details
**Blood samples after 2 hours added**	Additional blood-samples after 2 hours (5mL) (only Aarhus) and increase in volume of other blood-samples (from 19,5 to 21,5mL)
**Consent by research nurse**	Experienced research nurses can be delegated to obtain consent. Inclusion/randomization is always performed by a physician.
**Details updated on accepted treatment duration in cause of interrupted treatment**	In the case of acute stroke MRI and discontinued RIC/sham-RIC treatment – a full treatment protocol of 5 cycles are given after MRI (if <4 cycles were given before MRI)

3. Original protocol

(Clinical Investigation Plan version 7.0, February 9, 2018)

Clinical Investigation Plan

Remote ischemic conditioning in patients with acute stroke: a multicenter randomized, patient-assessor blinded, sham-controlled study.

A multicenter, prospective, randomized, patient-assessor blinded, sham controlled study investigating whether remote ischemic conditioning (RIC) can improve recovery.

**Project acronym**	**RESIST stroke trial**
	**RE**mote i**S**chemic conditioning **I**n patients with acute **ST**roke: a multicenter randomized, patient-assessor blinded, sham controlled study examining the effect on recovery
**Clinical investigation plan**	**Version 7.0, 9th of February 2018**
**Sponsor/Principal investigator (Aarhus)**	Grethe Andersen, Senior Consultant, MD, DMSc, Professor of Neurology. Department of Neurology, Aarhus University Hospital, DK-8000, Denmark. Phone:+4578463294, e-mail: greander@rm.dk
**Study Coordinator & investigator**	Rolf Blauenfeldt, MD, PhD Student. Department of Neurology, Aarhus University Hospital, DK-8000, Denmark. Phone: +4520774053, e-mail: rolfblau@rm.dk
**Principle Investigator (Holstebro)**	Birgitte Forsom Sandal, Senior Consultant, MD. Department of Neurology, Regional Hospital West Jutland (Holstebro), DK-7500, Denmark. Phone: +45 78437280, e-mail: Birgitte.Forsom.Sandal@vest.rm.dk
**Principle Investigator (Odense)**	Anne-Mette Homburg, Senior Consultant, MD. Department of Neurology. Odense University Hospital, DK-5000, Denmark. Phone: +45 65414691, e-mail: anne-mette.homburg@rsyd.dk
**Principle Investigator (Aalborg)**	Boris Modrau, Senior Consultant, PhD-student, MD. Department of Neurology. Aalborg University Hospital, DK-9000, Aalborg. Phone: +45 97662239, e-mail: boris.modrau@rn.dk
**Principle Investigator (Roskilde)**	Troels Wienecke, Senior Consultant, Associate professor, PhD, MD. Department of Neurology, Zealand University Hospital, DK-4000 Roskilde, Phone: +45 47322800, e-mail: trw@regionsjaelland.dk

RESIST trial is conducted according to the clinical investigation plan and current Danish legislation

The Clinical Investigation Plan will be approved and signed by all investigators before study start.

Contents

1. Synopsis ... 3
2. Abbreviations ... 8
3. Introduction ... 9
4. Hypothesis ... 10
5. Objectives ... 10
6. Trial design ... 11
7. Selection and withdrawal of subjects ... 12
8. Study procedure and assessment ... 14
9. Endpoints ... 18
10. Benefit of the study ... 25
11. Assessment of safety ... 25
12. Project timetable and recruitment feasibility ... 30
13. Ethical considerations ... 30
14. Data handling and record keeping ... 31
15. Publications policy ... 31
16. Statistics ... 31
17. Source data access and monitoring ... 34
18. Economy ... 34
References ... 38

1. Synopsis

**Name of the Sponsor/principal investigator**<br>Grethe Andersen, Senior Consultant, MD, DMSc, Professor of Neurology, Department of Neurology, Aarhus University Hospital, DK-8000, Denmark
**Name of investigational medical device: "Stroke RIC" & "Sham RIC"**<br>The investigational devices is developed in collaboration with the Faculty of Biomedical Engineering, Aarhus University, 8200-DK, Aarhus N, Denmark; Seagull Aps, 4160-DK, Herlufmagle, Denmark, and the Department of Neurology, Aarhus University Hospital, 8000-DK, Aarhus C, Denmark.
**Title of study: RESIST**<br>Remote ischemic conditioning in patients with acute stroke: a multicenter randomized, patient-assessor blinded, sham-controlled study, examining the effect on recovery.
<u>**Trial Management Groups (TMG):**</u><br>**Principal investigator/Sponsor (Aarhus University Hospital):**<br>Grethe Andersen, Senior Consultant, MD, DMSc, Professor of Neurology, Department of Neurology, Aarhus University Hospital, Nørrebrogade 44, 8000 Aarhus C, Denmark<br><br>**Study Coordinator/Investigator**<br>Rolf Ankerlund Blauenfeldt, MD, PhD student. Department of Neurology, Aarhus University Hospital, Nørrebrogade 44, 8000 Aarhus C, Denmark<br><br>**Trial Steering Committee (TMC):**<br>Grethe Andersen (Aarhus University Hospital), Rolf Ankerlund Blauenfeldt (Aarhus University Hospital), Niels Hjort (Aarhus University Hospital), Hans Erik Bøtker (Aarhus University Hospital), David C. Hess (Medical College of Georgia, USA), Hans Kirkegaard (Aarhus University Hospital). Rikke Bay Thomsen (Aarhus University Hospital), Birgitte Forsom Sandal (Regional Hospital West Jutland, Holstebro), Marc Fisher (Beth Israel Deaconess Medical Center, Harvard Medical School, USA), Ingunn Riddervold (Aarhus University Hospital),<br><br>**Data Monitoring Committee (and Trial Safety Committee):**<br>The DMC comprises of<br>Independent Senior Consultant (Neurologist) – chair.<br>Independent Statistician<br>Independent Senior Consultant (Neurologist)<br><br>**Trial Endpoints Validation Committee:**<br>EVC comprises of Independent senior consultants in Neurology and Cardiology<br>**Trial monitoring:**<br>During the entire study period and at all participating centres the regional GCP unit will perform quality assurance control including source data verification (GCP Unit Aarhus/Aalborg, ID 2017-718)
**Study centers:**<br>Department of Neurology, Aarhus University Hospital, DK-8000 Aarhus C, Denmark

Department of Neurology, Holstebro Hospital, DK-7500 Holstebro, Denmark<br>Department of Neurology, Odense University Hospital, DK-5000 Odense, Denmark<br>(Future study center, 2019 or 2020)<br>Department of Neurology. Aalborg University Hospital, DK-9000, Aalborg, Denmark<br>(Future study center, 2019 or 2020)<br>Department of Neurology, Zealand University Hospital, DK-4000 Roskilde, Denmark<br>(Future study center, in 2019 or 2020)<br><br>All Danish stroke centers will be invited to participate.	
**Planned study period:** 2018-2022.	
**Phase of development:**<br>Improve routine care of patients with acute stroke and transitory ischemic attack (TIA).	
**Objectives:**<br>To determine whether combined remote ischemic per- and postconditioning can reduce neurological impairment at 24 hours and improve long-term recovery in acute stroke patients as an adjunct to standard treatment.	
**Diagnosis:**<br>Acute ischemic stroke and intracerebral hemorrhage (ICH).	
**Methodology:**<br>A multicenter, investigator-driven, prospective, randomized, parallel assignment, patient-assessor blinded, sham-controlled clinical efficacy trial.	
**Randomization:**<br>• Eligible patients will be randomized prior to the arrival at the hospital (via a secure web site).<br>• The patients and the clinical outcome assessor will be blinded to the treatment allocation.	
**Number and subjects (planned):**<br>• 2,500 patients	
**Inclusion criteria (prehospital):**<br>The initial treatment regime will be applied in the acute prehospital phase in setting of an acute research study<br>• Male and female patients (≥ 18 years)<br>• Prehospital putative stroke<br>• Onset of stroke symptoms < 4 hours before remote ischemic conditioning (RIC)<br>• Independent in daily living before symptom onset (mRS ≤ 2)<br>**Final in-hospital inclusion criteria**<br>• acute ischemic stroke including documented TIA<br>*or*<br>• ICH	

or

- TIA without documentation and non-vascular diagnosis (register-based follow-up only)

Exclusion criteria (pre-hospital)

Exclusion criteria, to be established during the teleconference between ambulance and on call neurologist

- Intracranial aneurisms, arteriovenous (AV) malformation, cerebral neoplasm, or abscess
- Pregnancy
- Severe peripheral arterial disease in the upper extremities
- AV shunt in the arm selected for RIC
- Concomitant acute life-threatening medical or surgical condition

Criteria for evaluation

Primary endpoints

- Difference in prehospital stroke score (PreSS) during 24 hours

  ($\Delta PreSS = PreSS_{prehospital} - PreSS_{24\text{-}hours}$)
- Difference in acute hematoma expansion in patients with ICH (safety outcome)

Secondary endpoints

- Clinical outcome (mRS) at 3 months in acute ischemic stroke (AIS)
- Clinical outcome (mRS) at 3 months in subgroups: AIS, IV tPA/EVT treated AIS and ICH
- Clinical outcome (mRS) at 3 months in patients with AIS and ICH and extended remote ischemic postconditioning protocol (*substudy at Aarhus University Hospital*)
- Difference in prehospital stroke score (PreSS) during 24 hours in subgroups: IV tPA/EVT treated AIS and ICH.
- Proportion of RIC/Sham-RIC treated AIS patients with large vessel occlusion (LVO) eligible to EVT treatment (*substudy at Aarhus University Hospital*)
- Major Adverse Cardiac and Cerebral Events (MACCE) and recurrent ischemic events based on registry data at 3 and 12 months in ICH, AIS patients, TIA and non-vascular diagnosis
- Three-month and one-year mortality in AIS, ICH, and overall
- Early and very early neurological improvement in AIS and ICH patients
- Infarct growth in AIS patients and hematoma growth in ICH patients

- Predictive abilities of Glial Fibrillary Acidic Protein (GFAP) in prehospital obtained blood samples combined with prehospital stroke severity to differentiate hemorrhagic from ischemic stroke and to grade ischemic stroke severity (*substudy at Aarhus University Hospital*)
- Diagnostic abilities of a prehospital microRNA and extracellular vesicles blood samples profile combined with prehospital stroke severity on the differentiation of hemorrhagic from ischemic stroke and to grade ischemic stroke severity (*substudy at Aarhus University Hospital*)
- microRNA and extracellular vesicle profile of RIC-induced neuroprotection at baseline (*substudy at Aarhus University Hospital*)
- Bed-day use at 3 and 12 months and quality of life measures at 3 months

Study flow chart

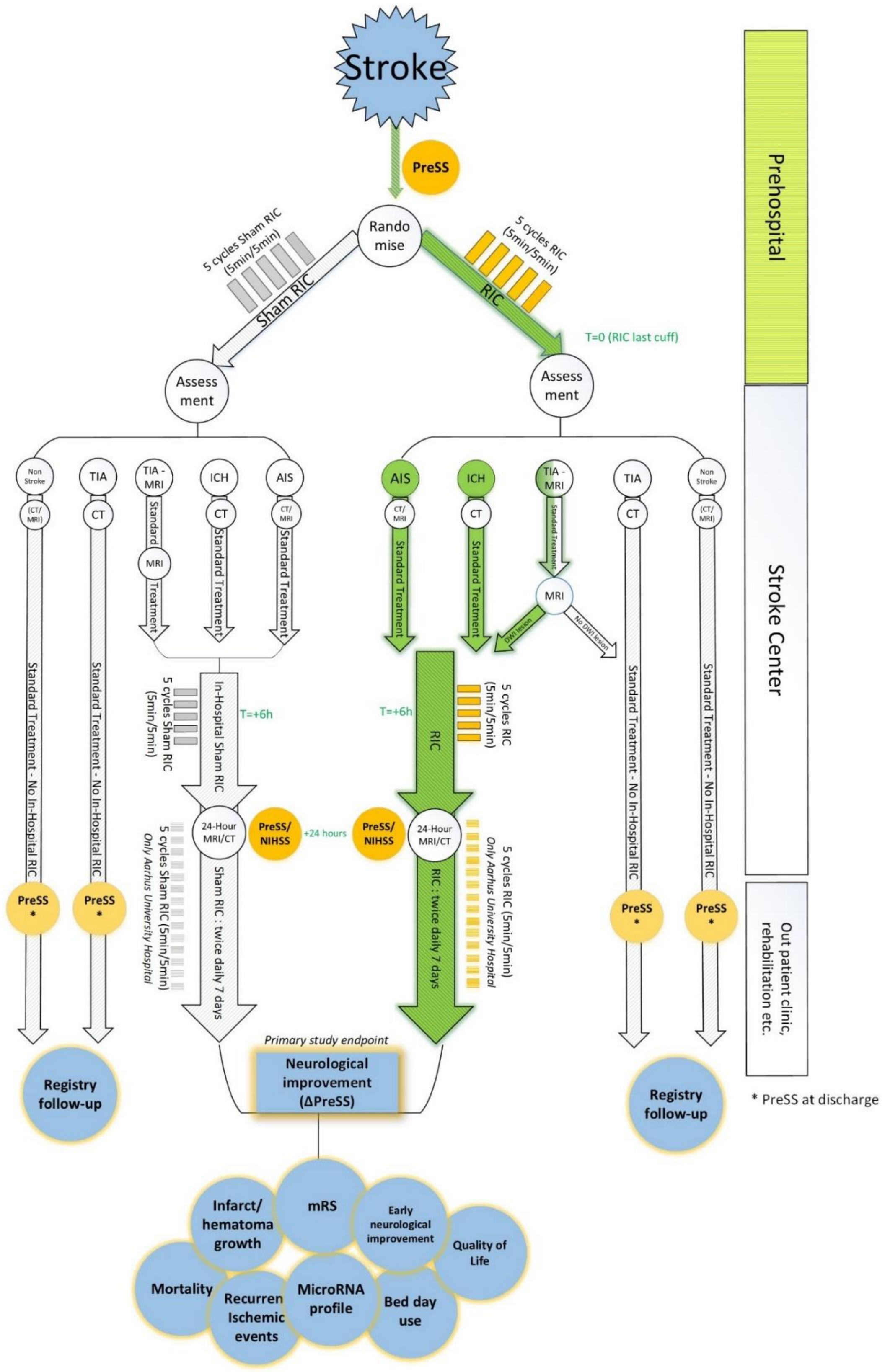

Abbreviations

AE	Adverse event
AIS	Acute ischemic stroke
AR	Adverse reaction
CT	Computed tomography
CPSS	Cincinnati Prehospital Stroke Scale
DWI	Diffusion-weighted imaging
DMC	Data Monitoring Committee and Endpoints Validation Committee
ICH	Intracerebral hemorrhage
END	Early neurological deterioration
EVT	Endovascular treatment
LVO	Large Vessel Occlusion
miRNA	Micro ribonucleic acid
mPTP	Mitochondrial permeability transition pore
MRI	Magnetic resonance imaging
NPR	Danish National Patient Register
PreSS	Prehospital Stroke Score
PWI	Perfusion-weighted imaging
RIC	Remote ischemic Conditioning
RIPerC	Remote Ischemic perconditioning
RIPreC	Remote ischemic preconditioning
RIPostC	Remote ischemic postconditioning
IV tPA	Intravenous thrombolysis/recombinant tissue-type plasminogen activator
SAE	Serious adverse event
SAR	Serious adverse reaction
TIA	Transient ischemic attack
TIA (DWI+)	Transient ischemic attack with a DWI-positive lesion on MRI
TMG	Trial Management Group
TSC	Trial Steering Group

2. Introduction

Our primary aim is to investigate whether remote ischemic conditioning (RIC) as an adjunctive treatment reduce neurological impairment at 24 hours and improve long-term recovery in acute stroke patients as an adjunct to standard treatment.

Stroke is the second-leading cause of death worldwide and a leading cause of serious, long-term disability. The most common type is AIS which occurs in 85% of cases. Acute cerebral thromboembolism leads to an area of permanent damage (infarct core) in the most severely hypoperfused area and a surrounding area of impaired, yet salvageable tissue known as the "ischemic penumbra"[1]. Intravenous alteplase (IV tPA) and endovascular treatment (EVT) are approved acute reperfusion treatments of AIS to be started within the first 4½-6 hours and as soon as possible after symptom onset to prevent the evolution of the infarct core[2,3,4,5]. The prognosis has improved overall for ischemic stroke and the one-month mortality rate has declined to an approx. 10% [6]. However, reperfusion itself may paradoxically result in tissue damage (reperfusion injury) and may contribute to infarct growth[7,8,9]. Infarct progression can continue for days following a stroke, and failure of the collateral flow is a critical factor determining infarct growth[10]. The pre-eminent therapeutic aim is to restore blood flow, improve collateral perfusion, and prevent reperfusion injury[11]. In transient ischemic attack (TIA), the ischemia and stroke symptoms are only temporary, but these patients possess a high risk of re-stroke – especially patients with evidence of infarction on neuroimaging[12]. TIA patients with a documented ischemic lesion are, per definition, handled as ischemic stroke in this study.

On the other hand, in ICH the culprit is an eruption of blood into the brain parenchyma causing tissue destruction with a massive effect on adjacent brain tissues. Hematoma expansion as well as inflammatory pathways that are activated lead to further tissue damage, edema, and penumbral hypoperfusion[13]. The prognosis after ICH is poor with a one-month mortality of 40%. Acute blood pressure lowering is recommended to prevent further hematoma growth, but the mechanisms behind this effect are not understood[14].

Novel therapeutics and neuroprotective strategies that can be started ultra-early after symptom onset are urgently needed to reduce disability in both AIS and ICH.

Ischemic conditioning is one of the most potent activators of endogenous protection against ischemia-reperfusion injury[15]. RIC can be applied as repeated short-lasting ischemia in a distant tissue that results in protection against subsequent long-lasting ischemic injury in the target organ[16]. This protection can be applied prior to or during a prolonged ischemic event as remote ischemic preconditioning (RIPreC) and perconditioning (RIPerC), respectively, or immediate after reperfusion as remote ischemic postconditioning (RIPostC)[17]. RIC is commonly achieved by inflation of a blood pressure cuff to induce 5-minute cycles of limb ischemia alternating with 5 minutes of reperfusion. RIC activates several protective mechanisms, through humoral and neuronal pathways[18]. Circulating microRNA appears to be intimately involved in the RIC stimulus and might act as possible effector molecules[19,20].

Preclinical studies show that RIC induces a promising infarct reduction in an experimental stroke model[21,22]. It has been demonstrated that RIC protects against ischemia-reperfusion injury in the heart, lung, kidney, and the brain[22]. Results from a recent proof-of-concept study at our institution indicate that RIPerC applied during ambulance transportation as an adjunctive to in-hospital IV tPA increases brain tissue survival after one month[23]. Furthermore, RIPerC patients had less severe neurological symptoms at admission and tended to have decreased perfusion deficits[23]. Another study using RIPreC found a decreased stroke

recurrence and shorter time to recovery in patients with intracranial arterial stenosis[24, 25]. Furthermore, emerging preclinical data indicate a significant acceleration of hematoma size reduction and an improved functional outcome after RIC-treated ICH[26].

To-date, no serious adverse events have been documented in RIC. The procedure has been applied in numerous cardiovascular ischemic patients and in patients suffering from cerebral hemorrhage (ICH/SAH)[23,27,28,29,30].

RIC is a non-pharmacologic and non-invasive treatment without noticeable discomfort that has first-aid potential worldwide[31]. However, whether combined remote ischemic per- and postconditioning can reduce acute neurological impairment and improve long-term recovery in AIS and ICH has never been investigated in a randomized controlled trial.

3. Hypothesis

RIC as an adjunctive treatment reduces neurological impairment during the first 24 hours and improve long-term functional recovery in AIS and ICH patients.

4. Objectives

Primary objectives

- To determine whether RIC reduces neurological impairment (PreSS) during the first 24 hours in AIS
- To examine the safety of RIC in ICH patients

Secondary objectives

- To determine whether RIC improves the clinical outcome (mRS) at 3 months in AIS
- To determine whether RIC improves the clinical outcome (mRS) at 3 months in subgroups of AIS, IV tPA/EVT-treated AIS patients and ICH patients
- To determine whether an extended RIC protocol (one week) improves clinical outcome (mRS) at 3 months in patients with AIS and ICH (*substudy at Aarhus University Hospital*)
- To determine whether RIC reduces neurological impairment (PreSS) at 24 hours in subgroups of IV tPA/EVT treated AIS and ICH.
- To determine whether RIC Increased the proportion of AIS patients with large vessel occlusion (LVO) eligible to EVT treatment (*substudy at Aarhus University Hospital*)
- To determine whether RIC reduces major adverse cardiac and cerebral events (MACCE) and reduces recurrent ischemic events at 3 and 12 months
- To determine whether RIC reduces the 3-month and 1-year mortality in patients with AIS, ICH, and overall
- To determine whether RIC increases the occurrence of early and very early neurological improvement in AIS and ICH patients
- To determine whether RIC reduces infarct growth in AIS patients and hematoma growth in ICH patients

- To determine whether prehospital obtained Glial Fibrillary Acidic Protein (GFAP) in blood samples combined with prehospital stroke severity can differentiate hemorrhagic from ischemic stroke and grade ischemic stroke severity (*substudy at Aarhus University Hospital*)
- To determine whether prehospital obtained microRNA and extracellular vesicles blood samples combined with prehospital stroke severity can differentiate hemorrhagic from ischemic stroke and grade ischemic stroke severity (*substudy at Aarhus University Hospital*)
- To determine whether a circulating microRNA and/or extracellular vesicle profile of RIC-induced neuroprotection can be established (s*ubstudy at Aarhus University Hospital*)
- To determine whether RIC reduces bed-day use at 3 and 12 months and affects quality of life at 3 months

5. Trial design

Trial design

This is a multicenter, prospective, randomized, patient-assessor blinded, sham-controlled trial investigating whether RIC can reduce neurological impairment at 24 hours and improve long-term recovery in acute stroke.

Number of centers

Patients with putative stroke from participating stroke centers in Denmark will be recruited.

Number of subjects

We estimate that a sample size of 2,500 prehospital putative stroke patients will be recruited to achieve 1,380 eligible AIS patients (primary study endpoint).

Sample size determination

Primary clinical endpoint

The sample size calculation is based on data from IV tPA-treated AIS patients from our institution using an estimated baseline and 24-hour PreSS (from NIHSS baseline and 24 hour). The proportion of AIS patients with a significant reduction of neurological impairment during 24 hours (PreSS score reduction of ≥ 1 point) was 0.43. To detect a 7 % absolute increase in neurological improvement in the RIC-treated AIS group at a power of 80% and a significance level of 5%, a sample size of at least 1,380 will be required. To account for non-stroke diagnosis (estimated 24%), ICH (estimated 8%), TIA (estimated 8%), withdrawal and loss to follow-up (estimated 4%)[8], we therefore plan to include 2,500 patients with a prehospital putative stroke. Lost to follow-up rate is estimated to be less 5%. There is no planned replacement of patients lost to follow-up.

Randomization

Randomization procedure

The patient will be randomized to standard treatment with RIC or sham-RIC by the on call neurologist/vascular neurologist at the receiving stroke center. The ambulance will contact the on call neurologist by telephone and describe the patient (standard operating procedure (SOP) in Denmark). The randomization is based on a secure web site providing computer-generated blocked randomization lists stratified by the center. The online randomization is stratified by age, strokecenter and he Prehospital

Stroke Score (PreSS). The PreSS score consists of the Cincinnati Prehospital Stroke Scale (CPSS) with an additional opportunity to report other neurological symptoms (e.g. ataxia, sensory disturbances and visual field loss), and PASS (Prehospital Acute Stroke Severity Scale)[32,33]. The on call neurologist will make an assessment based on all available information whether the patient is eligible to participate in RESIST. Randomization is performed in the prehospital setting. Each on call neurologist participating in the study will receive unique access and will have no influence on the randomization process. All neurologists on call will be educated and trained in performing evaluation of potential eligible study candidates and to perform the online randomization during the telephone call with the ambulance personnel. There is always one neurologist on call in participating centers (hospital SOP).

Treatment allocation

1:1 Allocation

6. Selection and withdrawal of subjects

Selection of patients

All patients with a putative stroke who meet the study criteria will be included.

- Depending on the prehospital randomization (RIC versus sham-RIC), patients with AIS will continue RIC or sham-RIC treatment for an extended period. This group includes TIA with a documented DWI lesion on magnetic resonance imaging (MRI).
- Patients with ICH will continue in-hospital RIC or sham-RIC (according to randomization).

Informed consent for prehospital admission

Background:

Acute temporary cognitive impairment after stroke is very common, even in patients with minor stroke or remission of symptoms (TIA) (54%) [34]. Widespread cognitive deficits have been identified in an acute stroke patient population[35]. This finding is in line with the clinical experience in acute stroke care. Whether the putative stroke patients have major cognitive impairment in the prehospital setting can only be tested under calm and quiet in-hospital circumstances. Delivering acute neuroprotective care, it is of utmost importance that treatment is started as soon as possible, i.e. without delay. RIC is without any known serious adverse events.

According to the Danish research ethics committees, a patient who is a candidate for inclusion in an acute research study is considered incompetent if that person is not able to care for his or her own affairs due to physical or mental impairment. We believe that the vast majority of stroke patients fulfil these criteria, and it is not possible to select the few who are, indeed, competent in the acute phase without formal cognitive testing and thus missing the purpose of acute neuroprotection intervention. The intervention is associated only with mild to moderate discomfort and minimal risk for adverse events.

Acute Prehospital phase:

The initial RIC/sham-RIC treatment regime will be applied in the acute prehospital phase in setting of an acute research study approved by the regional research ethics committees.

Whether further treatment, according to randomization, will be offered depends on the in-hospital examination.

In-hospital inclusion:

After the initial RIC/sham-RIC treatment, in-hospital diagnostic evaluation, and acute treatment, ***the competent patient*** will be presented with a consent form with information about the study. Before this, the on-duty neurologist will have examined the patient both physically and cognitively and will have assessed whether the patient is competent or not. The competent patient will receive oral information by the on-duty neurologist based on the written patient information. The written patient information will be handed out, (*deltager information*). We will ask for the patient's acceptance to be included in the study. The patient can withdraw consent at any time. In addition, the acute nature of the disease necessitates circumvention of the usual requirements concerning a 24-hour time period for consideration and discussion with a lay representative. This has been done in recent comparable acute studies. This approach is acceptable as the intervention is without any known risk and associated with a potential benefit for the patient. In-hospital inclusion and assessment of cognitive impairment will be undertaken during the admission at the stroke center. Every effort will be made to inform the patients and relatives in quiet and undisturbed settings, this will be at the hospital bedroom or the designated room for conversations. We will prioritize the presence of nearest relatives at time of study information and inclusion.

For ***the incompetent patients***, informed consent will be obtained from next of kin and an independent physician. The consenters will be provided information (based on the summary of the protocol for the independent physicians and study participant information for the relatives) on the trial to be able to make an informed decision about the patient's participation in this trial. The independent physician will be the on-duty physician at the local department of neurosurgery, neuroanesthesiology, intensive care, neurophysiology, (neuro)radiology or the on duty physician from another specialty with stroke patient experience. On-duty physicians at these departments will receive detailed study information before start of the study.

A patient who during the acute phase was legal incompetent and included as such, but during the follow-up period is assessed competent will receive oral and written information and we will ask for his/her's informed consent.

Patients who fulfill both prehospital and in-hospital inclusion criteria will, according to randomization, receive RIC/sham-RIC treatment again after +6 hours (***all centres***).

Participation in the extended remote ischemic conditioning(RIC) or sham-RIC (*for a total of 7 days*) study will take place ***only at Aarhus University Hospital***. The device will follow the patient when he/she is discharged from the stroke center.

TIA patients and non-stroke diagnosis patients will be asked for consent to do registry follow-up until one year after discharge.

Inclusion criteria (prehospital)

- Male and female patients (≥ 18 years)
- Prehospital putative stroke
- Onset of stroke symptoms < 4 hours before RIC
- Independent in daily living before symptom onset (mRS ≤ 2)

Exclusion criteria (prehospital)

- Intracranial aneurisms, AV malformation, cerebral neoplasm or abscess
- Pregnancy*
- Severe peripheral arterial disease in the upper extremities

- Concomitant acute life-threatening medical or surgical condition
- AV shunt in the arm selected for RIC

**Women of child-bearing age should be asked about their use of safe birth control methods (contraceptive pill, intrauterine devices both hormonal and non-hormonal, hormonal implants, hormonal depot injection and transdermal hormonal patch). If pregnancy cannot be ruled out in the prehospital phase the patient can't be included. Women with a safe birth control method should be encouraged to use this method during the entire period of active RIC treatment.*

Final in-hospital inclusion criteria

- AIS (including DWI-positive TIA patients)

  or

- ICH

  or

- TIA and non-stroke diagnose (register follow-up only)

Method of blinding

Outcome assessment is blinded to the treatment arm and obtained by a clinical examination (PreSS) in the prehospital setting and again after 24 hour at the stroke center (PreSS) (Primary study endpoint).
No information regarding randomization status will be recorded in the patient record.
Patients and the assessors of endpoints are blinded. Endpoints assessors will not take part of the daily routine at the stroke center, and are blinded to randomization status. Only information on name and age of the patient and time and place of the assessment will be available.

Discontinuation of study participation

A patient can withdraw from the study at any time. Patients can be withdrawn from the study at the principal investigator's discretion. In case the patient cannot be reached by telephone, every effort will be made to contact the patient or to document the outcome regarding new vascular events via registries. If the patient withdraws from the study, the date and the reason for the patient's withdrawal will be recorded. The patient is encouraged to provide information about his or her reason(s) for withdrawal and any experienced adverse effects (AE) during the study.

7. Study procedure and assessment

Prehospital stroke identification

Prehospital putative stroke patients are identified in collaboration with the ambulance personnel and the on-duty neurologist/vascular neurologist at the nearest stroke center (by telephone), which is standard operating procedure in Denmark. All Danish ambulance personnel are trained in identifying putative stroke in the prehospital setting. In the present study protocol, all patients are scored using the PreSS (before randomization) – which will further increase stroke symptom awareness.

Baseline data

Baseline data and process indicators are collected from the Danish Stroke Registry (DSR). Additional information about medical history and medication and clinical and physiological data are collected at baseline. Data are recorded in an electronic case report form (CRF) at discharge from the stroke unit. For a complete list of registered data, see *Supplement B*

Acute treatment:

Patients are treated according to the national clinical guidelines on stroke treatment, intravenous thrombolysis and endovascular therapy. All patients are treated with state-of-the-art multidisciplinary care in the setting of a stroke unit monitored by the DSR.

Blood samples

Patients with putative stroke admitted to the Aarhus University Hospital will have blood samples withdrawn, in the ambulance, from peripheral venous catheter (placed as standard operating procedure, SOP). A total of 2x5,5mL will be drawn in the prehospital phase.

Blood samples will also be drawn upon admission to the Aarhus University Hospital Stroke Center as a part of SOP. Additional 2 x 5.5mL of blood will be drawn at this time.

Only patients received at the Stroke Center at Aarhus University Hospital will have blood samples drawn. (Prehospital and in-hospital blood samples)

Blood samples will be stored until completion of the trial in a research biobank.

Patients not admitted at Aarhus University Hospital will have no additional blood samples drawn (neither prehospital or in-hospital)

MicroRNA, extracellular vesicles and Glial Fibrillary Acidic Protein (GFAP) analysis will be performed on the obtained blood. A research bio bank will be established and all blood samples obtained during the study are stored here. During the entire study and analysis period, the material will be stored at Aarhus University Hospital. The last patient will be included in December 2022. Analysis of blood samples, according to secondary endpoints, will be performed during the study and until 2 years after inclusion of the last patient (no later than 31. December 2024). Hereafter the remaining material will be transferred to a biobank for future unspecified research purposes. We will submit an application to the Danish Data Protection agency for this additional 20-year storage period of the blood samples (until 31 December 2044), after that, the samples will be destroyed. All analysis will be performed in Denmark.

Investigational devices and Remote ischemic conditioning protocols

Automatic remote ischemic conditioning devices

Both the RIC and sham-RIC device are developed in collaboration with Aarhus University, Faculty of Biomedical Engineering, 8200-DK, Aarhus N, Denmark; Seagull Aps, 4200-DK, Slagelse, Denmark and the Department of Neurology, Aarhus University Hospital, 8000-DK, Aarhus C, Denmark.

The devices are manufactured by Shenzhen Raycome Health Technology Co., Ltd, 3F, 51 Building, No.5 Qiong Yu Road, Hi-Tech Industrial Park, Nanshan District, Shenzhen, China 518057.

The ***Investigator's Brochure (IB)*** *contains a detailed description of the investigational devices including risk analysis assessment.*

A brief user's manual is found in each device bag. No preexisting experience is necessary in order to operate the device. Written and oral information combined (Healthcare professional at participating hospitals) with RESIST trial e-learning material (prehospital personnel) will be offered to familiarize the clinical personnel in using the investigational devices. Patients and relatives will be instructed using written and oral information.

Remote Ischemic Conditioning device (RIC device)

The device is programmed to five cycles (50 minutes), each consisting of five minutes of cuff inflation followed by five minutes with a deflated cuff. The cuff pressure will be 200mmHg; but if initial systolic blood pressure is above 175 mmHg, the cuff is automatically inflated to 35 mmHg above the systolic blood pressure. This is done to account for a maximum cuff air leakage of 6mmHg per minute and maximum deviation in bloodpressure measurement of 10mmHg. The maximum cuff pressure is 285 mmHg.

Sham – remote ischemic conditioning device (Sham-RIC device)

The device is programmed to five cycles (50 minutes), each consisting of five minutes of cuff inflation followed by five minutes with a deflated cuff. The cuff pressure during inflation will be 20mmHg.

Timestamps, blood pressure before RIC/sham-RIC and total RIC/sham-RIC cycles are recorded and stored on the device (accessible by *universal serial bus,* USB). The RIC and sham-RIC is pre-programmed before being delivered to collaborating centers/ambulances. No data on conditioning cuff pressure will be displayed on device screen.

In cases of short transport time, the RIC/sham-RIC protocol continues during the initial assessment at the stroke center. RIC/sham-RIC stimulation is discontinued just prior to the MRI and continues afterwards if the protocol was incomplete (< 5 cycles). In centers using CT, the RIC/sham-RIC protocol is continued during the scan until a total of 5 cycles are reached. The device automatically stops treatment when the 5 cycles are completed. Except for RIC/sham-RIC, the prehospital observation and treatment are according to standard procedures in the ambulance.

Stroke RIC/Sham-RIC will be used, only if approved by the Danish Medicines Agency
The bloodpressure and heartrate measurement, by RIC and Sham-RIC, will only be used for research purposes.

Initial remote ischemic conditioning
(all patients)

RIC/sham-RIC cuff will be placed on the non-paretic upper arm. The cuff is placed on the upper extremity on the same side of the suspected side of cerebral stroke, e.g. in right hemiparesis the cuff on the left arm, and in monosymptomatic aphasia the cuff is placed on the left arm.

Remote ischemic conditioning at +6 hours
(All patients with AIS, ICH – all centers)

At 6 hours after the first RIC/sham-RIC protocol (6 hours from the last cuff-off, respectively), another series of RIC/sham-RIC is performed; five cycles each consisting of 5 minutes of ischemia are followed by 5 minutes of reperfusion.

Remote ischemic postconditioning (twice daily for 7 days)
(AIS and ICH – only at Aarhus university Hospital)

The post-conditioning stimulus will be applied twice daily in the first week. This occurs at pre-specified times: 08.00 PM and AM. However, RIC/sham-RIC can be applied no sooner than 10 hours and not later than 21 hours after the initial perconditioning stimulus (last cuff).

Aarhus University Hospital: RIC/sham-RIC treatment will continue for a total of 7 days. If patients are discharged to their home or to another department, they will continue treatment according to written instructions, including application of the device for 7 days. After RIC/sham-RIC on day 7, the device is then returned to the Stroke Center.

RIC/sham-RIC protocol	Accepted time frame
**Prehospital RIC**	< 4 hours from symptom onset
**RIC at +6 h**	4 to 8h from last cuff of the initial RIC
**RIC twice daily for 7 days** – 08.00 PM and AM*	06.00 to 10.00 PM and 06.00 to 10.00 AM

*RIC/sham-RIC can be applied no sooner than 10 hours and no later than 21 hours after the initial perconditioning stimulus.

Device Accountability log

All devices are registered in a *device accountability log*. When the prehospital personnel arrives at the stroke center, with a patient included in RESIST trial, a new device (of the same color) will be handed out. Device ID, ambulance ID, contact details to the ambulance and date of device hand-out will be registered. It will be recorded on a paper-log in the stroke department. These data are transferred to an electronic log keeping track of device location and time to service/battery charge (1 year). *Further details see Investigators Brochure.*

Study procedures table

	Prehosp. stroke score (PreSS)[1]	Prehosp Blood samples[1]	RIC/Sham-RIC	In-hospital assessment[2]	CT/MRI baseline	+6 hour RIC/sham-RIC [4]	PreSS 24h [2]	CT/MRI 24h[3]	Extended (7 days) RIC/sham-RIC [5]	Clinical events during follow-up[6]	Functional outcome assessment at 3- months[7]
**AIS incl. TIA (DWI+) – Other¥**	X		X	X	X	X	X	X		X	X
**AIS incl. TIA (DWI+) – AUH***	X	X*	X	X	X(MRI)	X	X	X (MRI)	X	X	X
**ICH**	X	X*	X	X	X (CT)	X	X	X (CT)	(X)	X	X
**TIA (DWI-)**	X	X*	X	X	X		X			X	
**Non-stroke**	X	X*	X	X	X		X			X	

**AUH = Aarhus University Hospital. , ¥ Other= Other participating stroke centers*

[1] Prehospital stroke score (PreSS) and bloodsamples. *Prehospital blood samples: only patients transported to Aarhus University Hospital)*

[2] Acute assessment at the Stroke Center. NIHSS, blood samples (2 x 5.5mL blood) and ECG (standard). Assessment of neurological symptoms at 24-hour: NIHSS. Patients with non stroke diagnosis and TIA without a DWI-MRI lesion, who are discharged before 24 hours will be scored using PreSS at discharge (performed by the on call neurologist). *In-hospital RESIST blood samples only at Aarhus University Hospital*

[3] Baseline CT is preferable for ICH patients (hospital SOP). ICH patients will receive a 24 hour control CT (hospital SOP). If possible, a 24-hour MRI is performed in all included AIS patients with an MRI baseline scan. Furthermore, a 24-hour control CT will be provided for IV tPA-treated AIS patients, unless MRI can be performed and was the primary investigation.

[4] RIC/sham-RIC at +6 hours from last RIC cuff

[5] RIC/sham-RIC twice daily for 7 days *(Only at Aarhus University Hospital)*

[6] Mortality, MACCE, and recurrent ischemic events are recorded using the Danish National Patient Register (LPR) and DSR at 6 and 15 months after the inclusion of the last patient.

[7] Secondary endpoint assessments at 3 months by telephone interview (mRS and WHO-5).

8. Endpoints

An independent end-point committee will adjudicate clinical events.

Primary endpoints

Criteria for evaluation

Difference neurological impairment during the first 24 hours in AIS

Neurological deficits are documented using PreSS (prehospital and in-hospital). The PreSS score is obtained by the prehospital personnel and registered by the on-call neurologist before randomization. Prehospital Stroke Score is assessed at 24-hour by a certified and trained general neurology nurse/non-stroke research nurse who is blinded to the treatment intervention and who is not a part of the daily stroke ward routine. The PreSS score of patients with TIA (without DWI lesion) and non stroke diagnoses, are performed by the on-call neurologist, will be documented as *PreSS at discharge*, if discharge occurs before 24 hours. The outcome assessor will be alerted in advance of the assessment. Only information regarding time and place of assessment, patient name, age and social security number will be available. The PreSS is recorded in the electronic CRF. Clinical assessors are required to complete an online PreSS certification.

https://ekurser.rm.dk/test_projekter/anhesr/red-hjernen/scorm/Scorm2004%204th%20Edition/index.html

Primary study endpoint: Difference in prehospital stroke score (PreSS) during the first 24 hours (Ordinal logistic regression)

For a **detailed statistical analysis plan**, see **statistics**

Indicator	Accepted timeframe (from symptom onset)
PreSS	Close to symptom onset
$PreSS_{24h}$ (+ 24h)	+ 16 to 32h

Difference in acute hematoma expansion in patients with ICH

- Hematoma expansion is defined as the difference in volume between the baseline and the 24-hour CT hematoma volume. Significant hematoma growth is defined as an absolute growth exceeding 6 mL or a relative growth of more than 33% from the initial CT[36].

In all ICH patients, the baseline imaging modality is CT. In cases of initial MRI, a CT is performed afterwards (no later than < 3 hours from last RIC cuff)

Imaging protocol	Accepted time frame
CT baseline – ICH	< 3 hours from last RIC/sham-RIC cuff
MRI baseline – ICH	Perform CT as soon as possible and < 3 hours after the last RIC/sham-RIC cuff
Control CT (24-hour) – ICH	18-30 hours from baseline CT

Accepted time frame

Information about imaging protocols, see *Supplement A – Neuroimaging protocols*

Secondary endpoints

Clinical outcome (mRS) at 3 months in acute ischemic stroke

The level of dependency and need for help in daily activities will be determined by telephone interviews based on a systematic questionnaire performed by assessors who are blinded to the intervention at 3 and 12 months after the index stroke. The assessment will performed by two independent assessors (one week apart). The electronic CRF database will alert if disagreement occurs, and the patient will be contacted by a senior stroke neurologist who is blinded to the intervention who will assess the level of dependency. Every possible effort will be made to assess the outcome in patients who are unable to participate in the interview (this could be due to post stroke neurological impairment (for example aphasia and/or dysarthria), or that the patient cannot be reached by telephone). The outcome will then be assessed by contact to a named relative or the general practitioner. Several attempts to contact the patient by telephone will be made before contacting the named relative or general practitioner.
Death in the follow-up period will be obtained from Danish Civil Registration System (CPR).

The structured telephone interview will be based upon a validated Danish translation of the "slightly revised simplified, modified Rankin Scale questionnaire"[37]. The translation and its validation will be performed according to the AAOS guideline[36, 37]

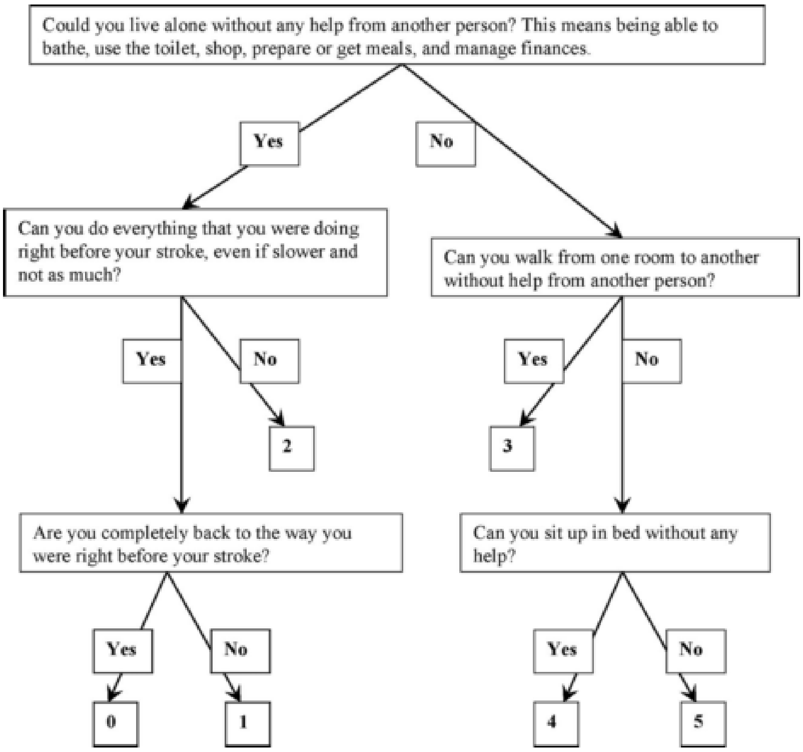

Bruno et al. 2011. Stroke.

Furthermore, clinical modified Rankin Scale assessors are required to complete an online mRS certification https://secure.trainingcampus.net/uas/modules/trees/windex.aspx?rx=rankin-english.trainingcampus.net

**Outcome**	**Accepted time frame (from symptom onset)**
3-month follow-up	3 months +/- 2 weeks

EVT-eligibility (MRI assessed) in RIC treated AIS patients with large vessel occlusion(LVO)

EVT eligibility is assessed upon arrival at the Aarhus University Hospital in AIS patients with:

- Severe Stroke (NIHSS ≥ 10)
- Groin puncture feasible within 6 hours from stroke onset
- MRI-TOF (time-of-flight) documented internal carotid artery (ICA), Intracranial ICA (ICA-T) and first and second stem of the middle cerebral artery (M1 and M2, respectively)
- No contraindications to MRI (pacemaker, vomiting, respiratory insufficiency, obesity)
- MRI-DWI lesion volume ≤ 70mL

Endpoint assessment
Proportion of RIC treated AIS patients with LVO eligible to EVT treatment compared to standard treatment, adjusted for prehospital stroke severity (PreSS) and symptom duration
Difference in MRI-DWI lesion volume in RIC/sham-RIC treated LVO AIS patients eligible to EVT treatment

Major adverse cardiac and cerebral events (MACCE) and recurrent ischemic events at 3 and 12 months in AIS patients

MACCE is defined as:

Cardiovascular events (cardiovascular death, myocardial infarction, acute ischemic or hemorrhagic stroke)

Cardiovascular death: Death from known cardiovascular cause or sudden death from unknown cause (no identified cause of death in medical history and/or autopsy)

Acute myocardial infarction: Admission with a discharge diagnosis of ST-elevation myocardial infarction (STEMI) and non-ST elevation myocardial infarction (NSTEMI) and unstable angina pectoris (UAP)

Stroke: Admission with a discharge diagnosis of acute ischemic or hemorrhagic stroke.
Evaluation is performed using the Danish National Patient Register (LPR) and the DSR at two time points (6 and 15 months after the inclusion of the last patient).

Diagnosis of AIS/TIA, ICH and MI (STEMI, NSTEMI, and UAP) are made according to national clinical practice guidelines.

(http://neuro.dk/wordpress/nnbv/om-iskaemisk-apopleksi/ and http://nbv.cardio.dk/aks)

Recurrent ischemic vascular events at 3 and 12 months in AIS patients
Recurrent ischemic vascular events defined as:

- AIS
- TIA
- MI, STEMI, and NSTEMI

Three-month and one-year mortality

Information about mortality is collected using the CPR and LPR, at two time points (6 and 15 months after the inclusion of the last patient). All-cause mortality is assessed and subdivided into cardiovascular mortality versus non-cardiovascular mortality[40].

Mortality (all-cause)

Cardiovascular mortality

- Acute MI
- Sudden cardiac death
- Heart failure
- Stroke: death as a direct consequence or complication of stroke
- Cardiovascular procedure or procedure
- Cardiovascular-related hemorrhage: non-stroke intracranial hemorrhage (subdural hematoma), non-traumatic vascular rupture (e.g. aortic aneurysm), or hemorrhage causing cardiac tamponade

Non - cardiovascular mortality

- Pulmonary, renal, gastrointestinal, hepatobiliary and pancreatic
- Infection and inflammatory/immune (including autoimmune)
- Hemorrhage (non-cardiovascular (CV)/non-stroke-related bleeding)
- Non-CV procedure or surgery, trauma, suicide, drug reaction, or overdose
- Neurological, malignancy, other non-CV

According to “2014ACC/AHA Key Data Elements and Definitions for Cardiovascular Endpoint Events in Clinical Trial”. Circulation 2015;132:302-361.

Early neurological improvement in AIS and ICH patients

Neurological deficits are documented with the PreSS and the National Institute of Health Stroke Scale (NIHSS), prehospital and in-hospital, respectively.

- **Very early neurological improvement** is defined as:

Reduction in prehospital stroke score (PreSS) ≥ 1 points or resolution of symptoms at admission: Items on PreSS related to NIHSS baseline $\Delta PreSS = PreSS_{prehospital} -$ same items on $NIHSS_{baseline}$

- **Early neurological improvement** is defined as:

Reduction in NIHSS ≥ 4 (baseline versus 24-Hour NIHSS): $\Delta NIHSS = NIHSS_{baseline} - NIHSS_{24}$

- **Reduction in prehospital stroke score (PreSS) ≥ 1 points or resolution of symptoms after 24 hours in subgroups:** IV tPA/EVT treated AIS and ICH.

Clinical assessors are required to complete an online PreSS certification

PreSS certification: https://ekurser.rm.dk/test_projekter/anhesr/red-hjernen/scorm/Scorm2004%204th%20Edition/index.html

Indicator	Accepted timeframe (from symptom onset)
PreSS	Close to symptom onset
NIHSS baseline	0 to 6 hours from admission
NIHSS (+ 24h) – standard operating procedure	+ 16 to 32h
PreSS (+ 24h)	+ 16 to 32h

Infarct growth in AIS patients

(Substudy at Aarhus University Hospital)
Acute infarct growth is defined as the difference in infarct volume between baseline and 24 hours on DWI-MRI. All patients with an ischemic stroke (including DWI+ TIA patients) and a baseline MRI will have an additional 24-hour MRI performed.

**Imaging protocol**	**Accepted time frame**
MRI baseline – AIS IV tPA eligible	On admission
MRI baseline – AIS not IV tPA eligible	< 6 hours from last RIC/sham-RIC cuff
MRI (24-hour) - AIS	18-30 hours from baseline MRI

Accepted time frame for baseline and 24-hour MRI

Information about imaging protocols, see *Supplement A*

Biochemical profile of stroke subtypes and of RIC-induced neuroprotection

(Substudy only at Aarhus University Hospital)

Prehospital biochemical substudies

- Predictive abilities of Glial Fibrillary Acidic Protein (GFAP) in prehospital obtained blood samples combined with prehospital stroke severity to differentiate hemorrhagic from ischemic stroke and to grade ischemic stroke severity
- Diagnostic abilities of a prehospital microRNA and extracellular vesicles blood samples profile combined with prehospital stroke severity to differentiate hemorrhagic from ischemic stroke and to grade ischemic stroke severity

In-hospital biochemical substudies

- Characterization of a possible microRNA and extracellular vesicle profile in stroke subtypes
- MicroRNA and extracellular vesicle characterization of a possible RIC treatment profile

Prehospital and in-hospital handling of blood samples:

Blood samples will be collected from patients with a prehospital putative stroke. Thus, blood samples will be drawn in the ambulance and upon arrival at the stroke center (only Aarhus University Hospital). In the ambulance, the blood sample will be obtained through a peripheral venous catheter (placed as standard operating procedure) into a 5.5 mL Sarstedt Monovette® sodium citrate tubes for plasma and transported to hospital at ambient temperature (Two tubes of 5,5mL will be drawn). The local biochemical department or study personnel will centrifuge the blood and divide the plasma into cryotubes (expected 0,5ml in each tube = 4 cryotubes) that will be kept at 80 °C for long-term storage. Cryotubes not used to measure GFAP within 5 to 7 days will be stored in a research biobank.

Blood samples will be drawn upon admission to the Aarhus University Hospital Stroke Center as a part of local stroke center SOP. Additional 2 x 5.5mL of blood will be drawn at this time.

All blood samples obtained during the study will be stored in a research biobank

Blood-samples, in the research biobank, will be stored until completion of the trial (no later than 31. December 2024). Hereafter the remaining material will be transferred to a biobank for future unspecified research purposes (see section “Blood Samples” under “study procedure and assessment”)

Planned biochemical analysis

Glial Acidic Fibrillary Protein (GFAP):

All plasma samples for GFAP measures will be analyzed with ELISA essay kits from the same batch at Clinical Biochemical Department, Regional Hospital Herning. Transport and shipment of samples will be done on dry ice to assure stability of the specimens. The samples will be marked with the unique research identification number. Cryotubes not used to measure GFAP will be stored as part of a biobank.

MicroRNA analysis:

MicroRNAs will be identified with Illumina next-generation sequencing using the TruSeq Small RNA Sample Preparation kit (Illumina) which allows for the addition of unique barcode sequences to each sample. Such barcoding allows pooling and simultaneous sequencing of multiple samples in a single-sequencing run on the Illumina NextSeq500 sequencer thereby significantly reducing the cost of sequencing. Pooling of multiple samples will generate adequate data amounts for detailed miRNA profiling, yet at limited costs. Data generated from the NextSeq500 sequencer will be filtered based on sequence quality as part of our established bioinformatics pipeline. This will also include matching the filtered data to annotated RNA databases such as miRNA sequences from miRBase (mirbase.org).

The output from our bioinformatic pipeline will be quantitative miRNA expression levels for each sample, which will form the basis for an miRNA differential analysis where miRNAs with statistically significant expression changes will be found.

The microRNA (and extraceullar vesicles) analysis is not a mapping of the genome. The microRNA profile is a snapshot of microRNA activity at the specific time of the blood sampling. Levels of microRNA are changing constantly and with the current knowledge on the topic it is impossible to predict a risk of developing specific diseases in the future. Cryotubes not used to microRNA/exosome analysis will be stored as part of a biobank.

Extracellular vesicle analysis:

Extracellular vesicles (EVs, also know as exosomes) will be isolated from plasma samples before characterization of surface markers and content. EVs are isolated by a number of different techniques, ultra centrifugation, precipitation, size exclusion chromatography among others. While protein characterization will be done using classical molecular biological techniques such as ELISA and Western blots in addition to array techniques, EVarray that utilises a panel of antibodies directed against known EV surface markers. These analyses might be supported by proteomic analysis of all proteins as well as post-translational modifications such as phosphorylations and glycosylations.

In addition, the nucleic acid (DNA and RNA including miRNA) content of EVs will be analysed using next generation sequencing (NGS), qRT-PCR, and other nucleic acid detection techniques. Both the protein data and the nucleic acid data will be subjected to bioinformatic analysis using different pipelines and analysis tools depending on the dataset and the purpose of the analysis. A number of validated published databases will be used for annotation and comparison. EVs do not contain genomic DNA and the analysis are therefore not a mapping of the subjects genome. As EV secretion and nucleic acid content in these are though to change rapidly in the body due to external and internal signals and are considered a snapshot at

the specific time of blood sampling. With the current knowledge on the topic it is impossible to predict a risk of developing specific diseases in the future.

Quality of life measures at 3 months in AIS and ICH patients

- **Quality of life and bed-day use measures in AIS and ICH patients**

Quality-of-life measurements are assessed by telephone interview at 3 months after inclusion. The WHO-5 is used.

Link to: Danish National Board of Health - WHO-5.
Information about bed-day use via the LPR and the DSR.

9. Benefit of the study

Potential benefits:

Participating patients with AIS and ICH may experience an immediate reduction of their neurological symptoms and a persistent reduction of disability at long-term follow-up.

Disadvantage:

Mild-to-moderate pain and petechiae in the RIC-treated arm may occur during the inflation of the blood pressure cuff. Otherwise, the RIC treatment has been proven safe and without side effects.

Sham-RIC will only be associated with a slight sensation of pressure on the upper extremity.

Blood samples (only patients transported to and admitted at Aarhus University Hospital) of approximately 22mL (11mL prehospital and 11 mL in-hospital) will be drawn.

10. Assessment of safety

Emergency unblinding procedure

All on call neurologists will have access to electronic case report form using a personal and password protected login to perform randomization of study participants. In cases where emergency unblinding is necessary the on call neurologist will logon to electronic CRF at ddsc.dk and enter the civil registration number (CPR) of the patient. The treatment can now be unblinded. All changes will leave an audit-trail.

Adverse events

Patients are monitored in the stroke unit with NIHSS scoring and Scandinavian Stroke Scale (SSS) at close intervals, and adverse events are treated according to clinical guidelines.

The Data Monitoring Committee will assess safety according to primary study endpoints, recurrent stroke, myocardial infarction and mortality.

Interim analysis of safety parameters (hematoma expansion at 24 hours and mortality) in ICH will be performed regularly during the study.

During the acute in-hospital phase the patients will be asked if they have experienced any deterioration of health or new symptoms during or after treatment with the investigational device. Furthermore patients treated with RIC for 7 days will have a 24/7 contact number to the stroke center and a contact to study research physician and will be instructed to report any deterioration of health or new symptoms. All discharged patients (and relatives) will be given contact details to study personnel and be instructed to report any deterioration of health or new symptoms. The patients will once again be asked for adverse events at the 3 month telephone interview.

Adverse events and adverse device events are defined according to ISO 14155:2011 and European Commission guideline on medical devices (MEDDEV 2.7/3 revision 3).

Adverse event (AE):

Any untoward medical occurrence, unintended disease or injury or any untoward clinical signs (including an abnormal laboratory finding) in subjects, users or other persons whether or not related to the investigational medical device.

Serious Adverse Event (SAE)

Adverse event that:

a) led to a death, injury or permanent impairment to a body structure or a body function.

b) led to a serious deterioration in health of the subject, that either resulted in: - a life-threatening illness or injury, or - a permanent impairment of a body structure or a body function, or - in-patient hospitalization or prolongation of existing hospitalization, or - in medical or surgical intervention to prevent life threatening illness

Device Deficiency

Inadequacy of an investigational medical device related to its identity, quality, durability, reliability, safety or performance. This could be malfunctions, use error, or inadequacy in the information supplied by the manufacturer.

Adverse Device Effect

Adverse event related to the use of an investigational medical device.

This includes any adverse event resulting from insufficiencies or inadequacies in the instructions for use, the deployment, the implantation, the installation, the operation, or any malfunction of the investigational medical device.

This includes any event that is a result of a use error or intentional abnormal use of the investigational medical device.

Serious Adverse Device Effect (SADE)

Adverse device effect that has resulted in any of the consequences characteristic of a serious adverse event.

Unanticipated Serious Adverse Device Effect (USADE)

Serious adverse device effect which by its nature, incidence, severity or outcome has not been identified in the current version of the risk analysis report.

Anticipated SADE (ASADE): an effect which by its nature, incidence, severity or outcome has been previously identified in the risk analysis report

Event report and causality assessment

The relationship between the use of the medical device and the occurrence of each adverse event will be assessed and categorized. All events will be registered in the electronic CRF and reported to the authorities at the interim analysis. A yearly safety report, containing a list of all SAEs/SADEs and near-miss incidents, thorough evaluation of individual events, risk/benefit analysis of the investigational medical device and safety conclusions, will be submitted to the Danish Health and Medicines Authority and the Independent Ethics Committee by the Sponsor as required by Danish law.

Exceptions to and special considerations about reporting procedures are stated below:

Acute stroke is an acute life threatening disease with a high risk of neurological deterioration and mortality. The natural history of stroke is associated with a high risk of complications (stroke in progression, hemorrhagic transformation, dysphagia, cardiac arrhythmia, pneumonia, and other serious infections). These *complications* all relates to the index stroke (occurring *before* randomization) and is foreseeable. If an event occurs that based on the assessment by the investigator is a result of the natural history of the disease it will not be reported by the sponsor to the Danish medicines agency and regional ethics committee within 7 days/2 days (see list of foreseeable events/complications below).

All expected serious adverse events will be reported to the Danish Medicines Agency (and regional ethics committee) every 3 months.

All *new* serious events (Serious Adverse Events), complications that are not foreseeable or device near incidents or malpractice will be reported by the sponsor to the authorities (within 7 days, or 2 days if there is a risk of event reoccurrence) according to ISO 14155:2011 and European Commission guideline on medical devices (MEDDEV 2.7/3 revision 3). Investigators will report the event to sponsor (within 24 hours). Sponsor will as fast as possible and no later than 7 days, or 2 days if there is a risk of event reoccurrence, report the event to the Danish Medicines Agency and regional ethics committee.

All Serious Adverse Device Effect (SADE) will be reported to the Danish Medicines Agency (and regional ethics committee) within 7 days, or 2 days if there is a risk of event reoccurrence

List of foreseeable adverse events/complications

Foreseeable events/complications that will not be reported by the sponsor to the Danish medicines agency and regional ethics committee within 7 days/2days.

Patient Population	Event	Frequency	Mitigation/treatment
**TIA and AIS**	Recurrent stroke.	10% at 90 days [a]	Standard medical treatment, intravenous thrombolysis/mechanical thrombectomy according to patient characteristics

**AIS**	Symptomatic intracerebral hemorrhage (related to IV tPA or EVT)	7% [b]	Antithrombotic/anticoagulation therapy cessation. Antifibrinolytic agent. Bloodpressure control.
**AIS, ICH**	Early (<24hours) and late (>24hours) neurological deterioration<br>(ΔNIHSS ≥2 or death)	7-22% [c]	According to etiology
**AIS, ICH**	Aspiration pneumonia	5-20% [d]	Antibiotic treatment according to hospital SOP
**AIS, ICH**	Urinary tract infections	12% [e]	Antibiotic treatment according to local hospital SOP
**AIS, ICH**	Severe infections (sepsis)	13% [f]	Antibiotic treatment according to local hospital SOP
**AIS, ICH**	Cardiac arrhythmia	29% [g]	Depending on arrhythmia type. Treatment according to cardio.dk
**AIS, ICH**	Acute myocardial infarction	6% [h]	Treatment according to cardio.dk and conference call with cardiologist
**AIS, ICH**	Deep venous Thrombosis(DVT)/pulmonary embolism(PE)	DVT: 8% [i]<br>PE:1% [j]	Treatment according to cardio.dk
**AIS, ICH**	Falls (in-hospital phase)	14-65% [k, l]	Treatment according to type of injury
**AIS/ICH**	Mortality 30 days	AIS:10-20% [m]<br>ICH:30% [n]	

a) Wang Y et al. Clopidogrel with Aspirin in Acute Minor Stroke or Transient Ischemic Attack. NEJM. 2013; 369:11-19

b) Berkhemer OA. A Randomized Trial of Intraarterial Treatment for Acute Ischemic Stroke. NEJM. 2015; 372:11-20

c) Kwan J et al. Early neurological deterioration in acute stroke: clinical characteristics and impact on outcome Journal of the Association of Physicians, 2006, Sept; 99:9: 625-633

d) Hannawi Y et al. Stroke-Associated Pneumonia: Major Advances and Obstacles. Cerebrovasc Dis. 2013;35:430-443

e) Bogason E. Urinary Tract Infections in Hospitalized Ischemic Stroke Patients: Source and Impact on Outcome. 2017;9(2):e1014. doi:10.7759/cureus.1014.

f) BergerB. Epidemiologic features, risk factors, and outcome of sepsis in stroke patients treated on a neurologic intensive care unit. Journal of Critical care. 2013;29(2)

g)Daniele O et al. Stroke and cardiac arrhythmias. J Stroke Cerebrovasc Dis. 2002 jan-feb;11(1):28-33

h) Johnston KC et al. Medical and neurological complications of ischemic stroke: experience from the RANTTAS trial. RANTTAS Investigators. Stroke. 1998;29(2):447

i) Bembenek J. Early stroke-related deep venous thrombosis: risk factors and influence on outcome. J thromb Thrombolysis. 2011 Jul; 32(1):96-102

j) Pongmoragot J. et al. Pulmonary Embolism in Ischemic Stroke: Clinical Presentation, Risk Factors, and Outcome. J Am Heart Ass. 2013 Dec;2(6)

k) Davenport RJ, Dennis MS, Wellwood I, Warlow CP. Complications after acute stroke. Stroke. 1996; 27: 415–420.

l Teasell R, McRae M, Foley N, Bhardwaj A. The incidence and consequences of falls in stroke patients during inpatient rehabilitation: Factors associated with high risk. Arch Phys Med Rehabil. 2002; 83: 329–333.

[m] Schwartz J et al. Incorporating Stroke Severity Into Hospital Measures of 30-Day Mortality After Ischemic Stroke Hospitalization. Stroke. 2017. Ahead of print: https://doi.org/10.1161/STROKEAHA.117.017960
[n)] Lichtman JH et al. 30-Day Mortality and Readmission after Hemorrhagic Stroke among Medicare

List of anticipated adverse device effects

RIC device	Frequency	Mitigation/treatment
**Local petechiae**	4-5% [a]	none
**Discomfort/pain**	20-30%	Treatment stopped if the non-competent patient verbally or non-verbally demonstrates intolerable discomfort or if the competent patient verbally want to discontinue the treatment
**Near events (device related)**	None known in the literature	
**Acute limb ischemia (upper extremity) – theoretical complication.**	None known in the literature	Treatment stopped. Treatment according local hospital SOP's for acute limb ischemia.<br>Known atherosclerotic stenosis in upper extremities are ***an exclusion criteria.***<br>See Investigators Brochure for further details.
**Sham RIC device**		
**None known**		

[a)] Hausenloy D et al. Remote Ischemic Preconditioning and Outcomes of Cardiac Surgery. New England J. Medicine. 2015 Oct;373(15):1408-17

Treatment and mitigation of any adverse events will be according regional guidelines.

Adverse events as a result of standard treatment will be handled according to local guidelines. Adverse events may also occur as a suspected reaction to RIC/Sham RIC – in these cases, the conditioning stimulus will be stopped immediately and appropriate treatment will be initiated.

In cases of SAE and device related near events all patients will be followed until resolution of symptoms/signs and/or all clinical relevant treatment have been performed and/or until the symptoms/signs are in a stable phase.

11. Project timetable and recruitment feasibility

Study preparation January to February 2017

Study start date: March 2018

Month 0-60: Patient recruitment

Expected inclusion end date: 31. December 2022

Month 60-66: Study analysis

Expected end date for last analysis and follow-up: 31. December 2024

Patient inclusion per center:

Aarhus University Hospital: 50%

Regional Hospital West Jutland (Holstebro): 12,5%

Odense University Hospital: 12,5%

Aalborg University Hospital: 12,5%

Zealand University Hospital (Roskilde): 12,5%

12. Ethical considerations

The study will await approval from the Regional Ethical Committee, the Danish Health Authority, and the Danish Data Protection Agency. Enrollment of additional Danish stroke centers will require a new application to the Danish Medicines Agency and the regional ethics committee (amendment).

A large acute stroke results in immediate, major neurological deficits (+/-aphasia) and a substantially increased risk of a long-term reduction of functional capability. This group is assessed as legally incompetent. Even in patients suffering from minor stroke, acute cognitive impairment are common, making individual preference on acute treatment options impossible. In order to preserve brain tissue, the intervention must be applied without delay. Improvement of standard care in patients suffering from acute stroke is of paramount importance. Inclusion of all stroke patients is necessary in order to translate any positive research results into a benefit for all stroke patients. The intervention is without any known risk apart from moderate pain in the upper extremity when the cuff is inflated. We find that the conditions for an acute study are satisfied since the majority of the patients are incapacitated at stroke onset and the treatment has to be initiated as soon as possible and there are no reported serious side effects from RIC.

Study participants are covered in accordance with the Danish Patient Insurance Act.

13. Data handling and record keeping

All study data are recorded in an electronic CRF with blinded data and identification via a study identification number. The study will apply to the specifications of act on processing of personal data.

The database(e-CRF) is handled by OPUS consult Aps, Egaa, Denmark, who already handles the Danish Stroke Center Database in Aarhus (ddsc.dk)

Remote Ischemic Conditioning in Stroke (RISC) is a predefined collaboration in which three large RIC trials have agreed to share anonymized patient data for a large metaanalysis once the individual trials have ended (Principle Investigators of the individual trials are: Grethe Andersen (DK), Philip Bath (UK) and Fernando Pico (France).

Patient participation will be recorded in the medical record. Data will be stored at the Department of Neurology, Aarhus University Hospital, for 15 years, after which the documents will be shredded.

A notification to The Danish Data Protection agency will be submitted.

During the entire study period and at all participating centers the local GCP unit will perform quality assurance control including source data verification.

A complete list of all source data will be made and approved by the local GCP unit before study initiation.

The investigator permits direct access to all source data/documents (including electronic patient record) at monitoring visits, audits and/or inspections by the regional ethics committee and Danish Medicines Agency.

14. Publications policy

The results of the study, both negative, inconclusive and positive, will be disseminated as widely as possible - through publication in an international peer-reviewed journal, as conference presentations and on www.clinicaltrials.gov.

The trial will be registered on www.clinicaltrials.gov and www.strokecenter.org/trials.

15. Statistics

Primary study endpoint:

- Difference in prehospital stroke score (PreSS) during the first 24 hours:

  ΔPreSS = $PreSS_{prehospital}$ – $PreSS_{24\text{-}hours}$ ***(Ordinal logistic regression analysis in the per-protocol population. Significance level of 5%)***

  The **main analysis of the primary study endpoint** will be performed using the entire range of the difference in PreSS score **(ordinal logistic regression, unadjusted)** on the ***per-protocol population.*** The per-protocol cohort consists of prehospital randomized patients with an in-hospital diagnosis of acute

ischemic stroke with complete investigational treatment and endpoint assessment as defined in the clinical investigational plan.

A supplementary analysis will be made on the **Intention-to-treat (ITT) population**. The ITT cohort includes all randomized subjects. In non-stroke diagnosis and TIA patients without MRI-DWI lesion, who are discharged before 24-hours, the stroke severity will be documented as *PreSS at discharge (performed by the on call neurologist)*
Furthermore, a supplementary analyses using multivariable regression will be done in order to account for any imbalances in prognostic factors including the distribution of patients receiving reperfusion therapy between the two treatment arms

- Difference in acute hematoma expansion in patients with ICH (safety outcome) (Binomial regression analysis. Intention-to-treat analysis (all randomized patients with an intracerebral hemorrhage, ICH) Significance level of 5%)

Secondary study endpoints (selected):

- Difference in clinical outcome (mRS) at 3 months in acute ischemic stroke *(ordinal logistic regression analysis. Significance level of 5%)*
- Difference in Early neurological improvement in AIS and ICH *(ordinal logistic regression analysis. Significance level of 5%)*
- Difference in prehospital stroke score (PreSS) after 24 hours in subgroups: IV tPA/EVT treated AIS and ICH. (ordinal logistic regression analysis, significance level of 5%)
- Major adverse cardiac and cerebral events (MACCE) and recurrent ischemic events at 3 and 12 months in AIS patients *(Cox regression analysis, significance level of 5%)*
- Three-month and one-year mortality *(Two sample test of proportion (Chi-square test), significance level of 5%)*
- EVT-eligibility (MRI assessed) in RIC treated AIS patients with large vessel occlusion (LVO) *(Binomial regression analysis. Significance level of 5%)*

Sample Size

The sample size calculation is based on data from IV tPA-treated AIS patients from our institution using an estimated baseline and 24-hour PreSS (from NIHSS baseline and 24 hour). The proportion of AIS patients with a significant reduction of neurological impairment during 24 hours (PreSS score reduction of ≥ 1 point) was 0.43. To detect a 7% absolute increase in neurological improvement in the RIC-treated AIS group at a power of 80% and a significance level of 5%, a sample size of at least 1,380 will be required. To account for non-stroke diagnosis (estimated 24%), ICH (estimated 8%), TIA (estimated 8%), withdrawal and loss to follow-up (estimated 4%)[8], we therefore plan to include 2,500 patients with a prehospital putative stroke.

Lost to follow-up rate is estimated to be less 5%. There is no planned replacement of patients lost to follow-up.

The primary endpoint analysis will be performed on the ***per-protocol population***.

Interim analysis

We will perform an interim analysis for evaluation of the actual event rate after inclusion of 50% of the patients. If this shows a lower good outcome rate than expected, a new sample size calculation will be performed. Furthermore, the Data monitoring committee (DMC) will perform an independent safety analysis and will review the overall safety and efficacy according to the DMC charter (planned in the early phase of the trial, at 12 month and hereafter at least every 18. months) An additional safety interim analysis of mortality and hematoma expansion in ICH patients will be performed. Interim analysis are assessed using a level of significance of 1%.

The trial can be stopped at interim analyses for both futility, efficacy and safety reasons.

Detailed description is found in the DMC charter.

Missing data:

Missing data with be handled using multiple imputation, in which missing cases are first filled in by several sets of plausible values to create multiple completed datasets and then the multiple sets of results are combined to yield a single inference.

Deviations from the statistical plan:

All deviations from the statistical plan will first be applied when approved by the regional ethics committee and the Danish Medicines Agency.

Trial decision making and stopping rules

A Data Monitoring Committee (DMC) will be appointed before trial start and include a signed DMC charter. The DMC will review the accumulated data during RESIST trial and provide advice on the conduct of the trial to the Steering Committee.

The DMC should inform the Steering Committee
if, in their view:

1. the results are likely to provide convincing evidence that one trial arm is clearly indicated or contraindicated, and there is a reasonable expectation that this new evidence would materially influence patient management

or

2. it is beyond doubt that no clear outcome would be obtained (futility)

It is not expected that RESIST trial will be stopped for reasons of efficacy or futility based on observed treatment differences. A predefined pooling of individual patient data (Remote Ischemic conditioning in Stroke Collaboration, RISC) is planned. To dispel scepticism, a robust demonstration of efficacy of this simple intervention is required. The primary reason to recommend stopping the trial will be for safety reasons.

For Acute Ischemic Stroke and Intracerebral Hemorrhage:

- For the primary endpoint the DMC will consider stopping if there is a robust reduction in prehospital stroke score (PreSS), for Acute Ischemic Stroke and Intracerebral Hemorrhage patients, during the first 24-hours in the sham (control) group compared to the intervention (RIC) group, achieving $p<0.01$.
- A substantial number of patients experience serious adverse events ($p<0.01$)
- For the primary endpoint the DMC will consider stopping if there is an increase in mortality or stroke recurrence for Acute Ischemic Stroke and Intracerebral Hemorrhage patients in the intervention (RIC) group compared to the control (sham) group, achieving $p<0.01$.
- For the primary safety endpoint in ICH the DMC will consider stopping/modification if there is a clear evidence hematoma growth during the first 24-hour in the intervention group, achieving $p<0.01$

The DMC will make recommendations, which could include:

- No action needed, trial continues as planned
- Early stopping due, for example, to clear benefit or harm of a treatment, futility, or external evidence
- Stopping recruitment within a subgroup
- Extending recruitment (based on actual control arm response rates being different to predicted other than on emerging differences) or extending follow-up
- Stopping a single arm of a multi-arm trial
- Approving and/or proposing protocol changes

16. Source data access and monitoring

Trial-related audits and/or monitoring will be provided by direct access to source data/documents. A local monitors from the Unit for Good Clinical Practice, Aarhus University, will perform the audit and monitoring. Audit and monitoring will, likewise, be perfomed by qualified local GCP-monitors when enrollment at other stroke centers in Denmark is starting. Before enrollment can start a trial monitoring plan must be available. The audit and monitoring process will involve a 100% monitoring of signed consent forms ("samtykkeerklæringer og fuldmagtserklæringer") and serious adverse events.

17. Economy

Study initiator is Grethe Andersen, Senior Consultant, MD, DMSc, Professor of Neurology at Department of Neurology, Aarhus University Hospital.

The Danish foundation Vilhelm Petersens mindelegat has supported the study with 1.240.000 dkr (research nurse salary and "stroke RIC" devices).

The Danish foundation TrygFonden has supported the study with 4.000.000 dkr (research personnel salaries, running costs, micro RNA analysis and investigational devices).

Aarhus University has supported the study with 550.000 dkr (study investigator/coordinator salary)

The funders have no role in study design, data collection, analysis or interpretation, nor the decision to publish or the preparation, review and approval of the manuscript.

Stroke RIC/Sham-RIC device is developed in collaboration with Aarhus University, Faculty of Biomedical Engineering, 8200-DK, Aarhus N, Denmark; Seagull Aps, 4160-DK, Herlufmagle, Denmark and the Department of Neurology, Aarhus University Hospital, 8000-DK, Aarhus C, Denmark. Seagull Aps has no role in study design, data collection, analysis or interpretation, nor the decision to publish or the preparation, review and approval of the manuscript.

The research grants are transferred directly to a research account administered by the financial department of Aarhus University Hospital. None of the involved doctors or research nurses has any conflict of interest or economic advantages in regards to the study. There is no economic compensation or reimbursement for patients participating in the study.

Supplements

Supplement A - Neuroimaging protocol

On admission, either CT or MRI is performed according to hospital SOP.

All AIS and ICH patients can be included in the in-hospital RIC/sham-RIC based on a baseline native CT. However, patients with complete remission of symptoms after randomization (TIA) require an MRI at baseline demonstrating acute ischemic lesion on DWI in order to continue the randomized treatment. TIA patients with a documented ischemic lesion are, per definition, handled as ischemic stroke in this study. Baseline MRI in TIA patients is performed before the next RIC series (MRI <6 hours from RIC/sham-RIC last cuff). Patients with AIS who are not eligible for IV tPA due to contraindications will have CT/MRI before their next RIC/sham-RIC series (< 6 hours from RIPerC last cuff) according to hospital SOP. All ICH patients will have a baseline and 24-hour CT (hospital SOP)

Non-stroke diagnosis and TIA (only evaluated with CT or TIA without DWI lesion on MRI) will not be included in the in-hospital RIC study and will receive no further RIC treatment or neuroimaging.

**Imaging protocol**	**Accepted time frame**
MRI baseline – AIS IV tPA eligible	Acute on admission
CT baseline – AIS IV tPA eligible	Acute on admission
MRI/CT baseline – AIS non IV tPA eligible	< 6 hours from last RIC cuff
MRI/CT (24-hour) – IV tPA/EVT treated AIS	18-30 hour from baseline MRI/CT
MRI (24-hour) - AIS non IV tPA/EVT eligible*	18-30 hour from baseline MRI
MRI baseline – ICH	Perform CT as soon as possible and < 3 hours from last RIC cuff
CT baseline – ICH	< 6 hours from last RIC cuff
Control CT (24-hour) – ICH	18-30 hour from baseline CT

*Substudy at Aarhus University Hospital

Computed tomography (CT) protocol

Baseline CT and CT angiography are obtained in centers using CT for primary assessment (according to hospital SOP).

CT analysis

Hematoma volume assessment at baseline and at 24 hours is performed by an experienced neuroradiologist. The assessment will be assisted using an automated stroke volume assessment software (e.g. COMBAT stroke APS, Aarhus University Hospital, Denmark) or using the formula *ABC*/2 where *A* is the greatest hematoma diameter by CT, *B* is the diameter perpendicular to *A*, and *C* is the number of CT slices with hematoma multiplied by slice thickness[41].

Magnetic resonance imaging (MRI) protocol

Diffusion-weighted imaging (DWI), apparent diffusion coefficient (ADC), T2* gradient-recalled echo (T2*GRE), and T2 fluid-attenuated inverse recovery (T2-FLAIR). MR angiography time-of-flight (MRA-TOF) is performed according to hospital standard operating procedures (SOP).

The 24-hour MRI is performed on the same MRI scanner used at baseline, if possible. This protocol includes: DWI, T2*, T2 and T2-FLAIR. MRA-TOF at 24 hours is obtained if the baseline CT-angiography or MRA-TOF demonstrated vessel occlusion.

The total acquisition time for this protocol is approximately 7-10 mins (MRI)/10-15 min (MRI + MRA-TOF), depending on the MRI equipment.

MRI analysis

Acute (baseline) and 24-hour follow-up (DWI lesion will be outlined, representing irreversibly damaged tissue *(substudy at Aarhus University Hospital).*

Infarct growth is defined as the difference between baseline DWI and the 24-hour MRI-DWI lesion.

Infarct volume assessment at baseline and at 24 hours is performed by an experienced neuroradiologist. The assessment will be assisted using an automated stroke volume assessment software (e.g. COMBAT stroke APS, Aarhus University Hospital, Denmark) or using the formula *ABC*/2 where *A* is the greatest hematoma diameter by CT, *B* is the diameter perpendicular to *A*, and *C* is the number of CT slices with hematoma multiplied by slice thickness[41]

Vascular patency and reperfusion are assessed using the Thrombolysis in Cerebral Infarction Perfusion Scale (TICI) criteria[42]

- No perfusion (TICI 0)
- Perfusion past the initial occlusion, but no distal branch filling (TICI 1)
- Perfusion with incomplete or slow distal branch filling (TICI 2)
- Full perfusion with filling of all distal branches, including M3 and M4 (TICI 3)

Supplement B – Baseline data

Age	Platelet inhibitor treatment
Male/female	Anticoagulation therapy
**Medical History**	New oral anticoagulation treatment (NOAC)
Hypertension	Opioid treatment
Smoking	SSRI treatment
Alcohol	**Clinical and physiological data**
Hyperlipidemia	Prehospital Stroke Score (PreSS)
Diabetes	Modified Rankin Scale prestroke
Previous myocardial infarction<br>*Yes: within 3 months, yes: more than 3 months, no, unknown*	NIHSS (baseline)
Angina pectoris	Symptom onset (time)
Recent angina pectoris (< 4 weeks)<br>*If yes, date for latest episode*	Time of admission
Atrial fibrillation	Completed prehospital RIC/sham cycles
Previous ischemic stroke	= 5, 4, 3, 1-2, 0
Stroke < 3 month<br>*If yes, date*	Time (start/stop) for each RIC/sham cycle
Previous TIA	NIHSS (t = 24 hours)
Recent TIA < 4 weeks<br>*If yes, date for latest episode*	Stroke etiology (TOAST)
Previous ICH	Admission blood pressure
Peripheral artery disease	**TIA patients**
Physical activity (PASE interview)	Symptom duration (minutes)
**Medication on admission**	**IV tPA/EVT treated AIS**
Statins	Treatment initiation (time)
Calcium channel blockers	**Quality indicators (Danish Stroke Registry)**
ACE inhibitors/angiotensin receptor blockers	
Beta-blockers	

References

1. Astrup J, Siesjo BK, Symon L. Thresholds in cerebral ischemia - the ischemic penumbra. *Stroke*. 1981;12(6):723-725.
2. Group N study. Tissue plasminogen activator for acute ischemic stroke. *N Engl J Med*. 1995;333(24).
3. Hacke W, Kaste M, Bluhmki E. Thrombolysis with alteplase 3 to 4.5 hours after acute ischemic stroke. *… Engl J …*. 2008:1317-1330.
4. Berkhemer OA, Fransen PSS, Beumer D, et al. A randomized trial of intraarterial treatment for acute ischemic stroke. *N Engl J Med*. 2015;372(1):11-20. doi:10.1056/NEJMoa1411587.
5. Touma L, Filion KB, Sterling LH, Atallah R, Windle SB, Eisenberg MJ. Stent Retrievers for the Treatment of Acute Ischemic Stroke: A Systematic Review and Meta-analysis of Randomized Clinical Trials. *JAMA Neurol*. January 2016. doi:10.1001/jamaneurol.2015.4441.
6. Prior A, Laursen TM, Larsen KK, et al. Post-Stroke Mortality , Stroke Severity , and Preadmission Antipsychotic Medicine Use – A Population-Based Cohort Study. 2014;9(1). doi:10.1371/journal.pone.0084103.
7. Ren C, Gao M, Dornbos D, et al. Remote ischemic post-conditioning reduced brain damage in experimental ischemia/reperfusion injury. *Neurol Res*. 2011;33(5):514-519. doi:10.1179/016164111X13007856084241.
8. Hougaard KD, Hjort N, Zeidler D, et al. Remote ischemic perconditioning in thrombolysed stroke patients: randomized study of activating endogenous neuroprotection - design and MRI measurements. *Int J Stroke*. 2013;8(2):141-146. doi:10.1111/j.1747-4949.2012.00786.x.
9. Khatri R, McKinney AM, Swenson B, Janardhan V. Blood-brain barrier, reperfusion injury, and hemorrhagic transformation in acute ischemic stroke. *Neurology*. 2012;79(13 Suppl 1):S52-7. doi:10.1212/WNL.0b013e3182697e70.
10. Campbell BC V, Christensen S, Tress BM, et al. Failure of collateral blood flow is associated with infarct growth in ischemic stroke. *J Cereb Blood Flow Metab*. 2013;33(8):1168-1172. doi:10.1038/jcbfm.2013.77.
11. Ip HL, Liebeskind DS. The future of ischemic stroke: flow from prehospital neuroprotection to definitive reperfusion. *Interv Neurol*. 2014;2(3):105-117. doi:10.1159/000357164.
12. Giles MF, Albers GW, Kim AS, Sciolla R. Early stroke risk and ABCD2 score performance in tissue- vs time-defined TIA A multicenter study. *Neurology*. 2011.
13. Kellner CP, Connolly ES. Neuroprotective Strategies for Intracerebral Hemorrhage Trials and Translation. 2010. doi:10.1161/STROKEAHA.110.597476.
14. Anderson CS, Heeley E, Huang Y, et al. Rapid blood-pressure lowering in patients with acute intracerebral hemorrhage. *N Engl J Med*. 2013;368(25):2355-2365. doi:10.1056/NEJMoa1214609.
15. Murry CE, Jennings RB, Reimer KA. Preconditioning with ischemia: a delay of lethal cell injury in ischemic myocardium. *Circulation*. 1986;74(5):1124-1136.
16. Hahn CD, Manlhiot C, Schmidt MR, Nielsen TT, Redington AN. Remote ischemic per-conditioning: a novel therapy for acute stroke? *Stroke*. 2011;42(10):2960-2962. doi:10.1161/STROKEAHA.111.622340.
17. Hess DC, Hoda MN, Bhatia K. Remote limb perconditioning [corrected] and postconditioning: will it

translate into a promising treatment for acute stroke? *Stroke*. 2013;44(4):1191-1197. doi:10.1161/STROKEAHA.112.678482.

18. Schmidt MR, Pryds K, Bøtker HE. Novel adjunctive treatments of myocardial infarction. *World J Cardiol*. 2014;6(6):434-443. doi:10.4330/wjc.v6.i6.434.

19. Li J, Rohailla S, Gelber N, et al. MicroRNA-144 is a circulating effector of remote ischemic preconditioning. *Basic Res Cardiol*. 2014;109(5):423. doi:10.1007/s00395-014-0423-z.

20. Schmidt MR, Redington A, Bøtker HE. Remote conditioning the heart overview - translatability and mechanism. *Br J Pharmacol*. September 2014:1-37. doi:10.1111/bph.12933.

21. Hoda MN, Bhatia K, Hafez SS, et al. Remote ischemic perconditioning is effective after embolic stroke in ovariectomized female mice. *Transl Stroke Res*. 2014;5(4):484-490. doi:10.1007/s12975-013-0318-6.

22. Lim SY, Hausenloy DJ. Remote ischemic conditioning: from bench to bedside. *Front Physiol*. 2012;3(February):27. doi:10.3389/fphys.2012.00027.

23. Hougaard KD, Hjort N, Zeidler D, et al. Remote ischemic perconditioning as an adjunct therapy to thrombolysis in patients with acute ischemic stroke: a randomized trial. *Stroke*. 2014;45(1):159-167. doi:10.1161/STROKEAHA.113.001346.

24. Meng R, Asmaro K, Meng L, et al. Upper limb ischemic preconditioning prevents recurrent stroke in intracranial arterial stenosis. *Neurology*. 2012;79(18):1853-1861. doi:10.1212/WNL.0b013e318271f76a.

25. Meng R, Ding Y, Asmaro K, et al. Ischemic Conditioning Is Safe and Effective for Octo- and Nonagenarians in Stroke Prevention and Treatment. *Neurotherapeutics*. 2015;12(3):667-677. doi:10.1007/s13311-015-0358-6.

26. Vaibhav K, Khan MB, Baban B, et al. Abstract W P253: Repeated Remote Ischemic Conditioning (RIC) after Intracerebral Hemorrhage Regulates Macrophage Polarization and CD36 Expression to Promote Hematoma Resolution. *Stroke*. 2015;46(Suppl 1):AWP253-AWP253.

27. Le Page S, Bejan-Angoulvant T, Angoulvant D, Prunier F. Remote ischemic conditioning and cardioprotection: a systematic review and meta-analysis of randomized clinical trials. *Basic Res Cardiol*. 2015;110(2):11. doi:10.1007/s00395-015-0467-8.

28. Bøtker HE, Kharbanda R, Schmidt MR, et al. Remote ischaemic conditioning before hospital admission, as a complement to angioplasty, and effect on myocardial salvage in patients with acute myocardial infarction: a randomised trial. *Lancet*. 2010;375(9716):727-734. doi:10.1016/S0140-6736(09)62001-8.

29. Hausenloy DJ, Candilio L, Evans R, et al. Remote Ischemic Preconditioning and Outcomes of Cardiac Surgery. *N Engl J Med*. 2015;373(15):1408-1417. doi:10.1056/NEJMoa1413534.

30. Laiwalla AN, Ooi YC, Liou R, Gonzalez NR. Matched Cohort Analysis of the Effects of Limb Remote Ischemic Conditioning in Patients with Aneurysmal Subarachnoid Hemorrhage. *Transl Stroke Res*. 2016;7(1):42-48. doi:10.1007/s12975-015-0437-3.

31. Hess DC, Blauenfeldt RA, Andersen G, et al. Remote ischaemic conditioning-a new paradigm of self-protection in the brain. *Nat Rev Neurol*. 2015;11(12):698-710. doi:10.1038/nrneurol.2015.223.

32. Hastrup S, Damgaard D, Johnsen SP, Andersen G. Prehospital Acute Stroke Severity Scale to Predict Large Artery Occlusion: Design and Comparison With Other Scales. *Stroke*. 2016;47(7):1772-1776. doi:10.1161/STROKEAHA.115.012482.

33. Kothari RU, Pancioli A, Liu T, Brott T, Broderick J. Cincinnati Prehospital Stroke Scale: reproducibility and validity. *Ann Emerg Med*. 1999;33(4):373-378.

34. Sivakumar L, Kate M, Jeerakathil T, Camicioli R, Buck B, Butcher K. Serial montreal cognitive assessments demonstrate reversible cognitive impairment in patients with acute transient ischemic attack and minor stroke. *Stroke*. 2014;45(6):1709-1715. doi:10.1161/STROKEAHA.114.004726.

35. Riepe MW, Riss S, Bittner D, Huber R. Screening for cognitive impairment in patients with acute stroke. *Dement Geriatr Cogn Disord*. 2004;17(1-2):49-53. doi:10.1159/000074082.

36. Demchuk AM, Dowlatshahi D, Rodriguez-Luna D, et al. Prediction of haematoma growth and outcome in patients with intracerebral haemorrhage using the CT-angiography spot sign (PREDICT): a prospective observational study. *Lancet Neurol*. 2012;11(4):307-314. doi:10.1016/S1474-4422(12)70038-8.

37. Bruno A, Akinwuntan AE, Lin C, et al. Simplified modified rankin scale questionnaire: reproducibility over the telephone and validation with quality of life. *Stroke*. 2011;42(8):2276-2279. doi:10.1161/STROKEAHA.111.613273.

38. Guillemin F, Bombardier C, Beaton D. Cross-cultural adaptation of health-related quality of life measures: literature review and proposed guidelines. *J Clin Epidemiol*. 1993;46(12):1417-1432.

39. Beaton DE, Bombardier C, Guillemin F, Ferraz MB. Guidelines for the process of cross-cultural adaptation of self-report measures. *Spine (Phila Pa 1976)*. 2000;25(24):3186-3191.

40. Kappetein AP, Head SJ, Genereux P, et al. Updated standardized endpoint definitions for transcatheter aortic valve implantation: the Valve Academic Research Consortium-2 consensus document. *Eur Heart J*. 2012;33(19):2403-2418. doi:10.1093/eurheartj/ehs255.

41. Kothari RU, Brott T, Broderick JP, et al. The ABCs of measuring intracerebral hemorrhage volumes. *Stroke*. 1996;27(8):1304-1305.

42. Khatri P, Neff J, Broderick JP, Khoury JC, Carrozzella J, Tomsick T. Revascularization end points in stroke interventional trials: recanalization versus reperfusion in IMS-I. *Stroke*. 2005;36(11):2400-2403. doi:10.1161/01.STR.0000185698.45720.58.

4. Protocol amendment

(Clinical Investigation Plan version 8.0, November 29, 2018)

Clinical Investigation Plan

Remote ischemic conditioning in patients with acute stroke: a multicenter randomized, patient-assessor blinded, sham-controlled study.

A multicenter, prospective, randomized, patient-assessor blinded, sham controlled study investigating whether remote ischemic conditioning (RIC) can improve recovery.

**Project acronym**	**RESIST stroke trial**<br>**RE**mote i**S**chemic conditioning **I**n patients with acute **ST**roke: a multicenter randomized, patient-assessor blinded, sham controlled study examining the effect on recovery
**Clinical investigation plan**	**Version 8.0, 29th of November 2018**
**Sponsor/Principal investigator (Aarhus)**	Grethe Andersen, Senior Consultant, MD, DMSc, Professor of Neurology. Department of Neurology, Aarhus University Hospital, DK-8000, Denmark. Phone:+4578463294, e-mail: greander@rm.dk
**Study Coordinator & investigator**	Rolf Blauenfeldt, MD, PhD Student. Department of Neurology, Aarhus University Hospital, DK-8000, Denmark. Phone: +4520774053, e-mail: rolfblau@rm.dk
**Principle Investigator (Holstebro)**	Birgitte Forsom Sandal, Senior Consultant, MD. Department of Neurology, Regional Hospital West Jutland (Holstebro), DK-7500, Denmark. Phone: +45 78437280, e-mail: Birgitte.Forsom.Sandal@vest.rm.dk
**Principle Investigator (Odense)**	Anne-Mette Homburg, Senior Consultant, MD. Department of Neurology. Odense University Hospital, DK-5000, Denmark. Phone: +45 65414691, e-mail: anne-mette.homburg@rsyd.dk
**Principle Investigator (Aalborg)**	Boris Modrau, Senior Consultant, PhD-student, MD. Department of Neurology. Aalborg University Hospital, DK-9000, Aalborg. Phone: +45 97662239, e-mail: boris.modrau@rn.dk
**Principle Investigator (Roskilde)**	Troels Wienecke, Senior Consultant, Associate professor, PhD, MD. Department of Neurology, Zealand University Hospital, DK-4000 Roskilde, Phone: +45 47322800, e-mail: trw@regionsjaelland.dk

RESIST trial is conducted according to the clinical investigation plan and current Danish legislation

The Clinical Investigation Plan will be approved and signed by all investigators before study start.

Contents

1. Synopsis 3
2. Abbreviations 7
3. Introduction 9
4. Hypothesis 10
5. Objectives 10
6. Trial design 11
7. Selection and withdrawal of subjects 13
8. Study procedure and assessment 16
9. Endpoints 20
10. Benefit of the study 29
11. Assessment of safety 30
12. Project timetable and recruitment feasibility 34
13. Ethical considerations 35
14. Data handling and record keeping 35
15. Publications policy 36
16. Statistics 36
17. Source data access and monitoring 40
18. Economy 40

References 44

1. Synopsis

**Name of the Sponsor/principal investigator**<br>Grethe Andersen, Senior Consultant, MD, DMSc, Professor of Neurology, Department of Neurology, Aarhus University Hospital, DK-8000, Denmark
**Name of investigational medical device: "Stroke RIC" & "Sham RIC"**<br>The investigational devices is developed in collaboration with the Faculty of Biomedical Engineering, Aarhus University, 8200-DK, Aarhus N, Denmark; Seagull Aps, 4160-DK, Herlufmagle, Denmark, and the Department of Neurology, Aarhus University Hospital, 8000-DK, Aarhus C, Denmark.
**Title of study: RESIST**<br>Remote ischemic conditioning in patients with acute stroke: a multicenter randomized, patient-assessor blinded, sham-controlled study, examining the effect on recovery.
**Trial Management Groups (TMG):**<br>**Principal investigator/Sponsor (Aarhus University Hospital):**<br>Grethe Andersen, Senior Consultant, MD, DMSc, Professor of Neurology, Department of Neurology, Aarhus University Hospital, Nørrebrogade 44, 8000 Aarhus C, Denmark<br><br>**Study Coordinator/Investigator**<br>Rolf Ankerlund Blauenfeldt, MD, PhD student. Department of Neurology, Aarhus University Hospital, Nørrebrogade 44, 8000 Aarhus C, Denmark<br><br>**Trial Steering Committee (TMC):**<br>Grethe Andersen (Aarhus University Hospital), Rolf Ankerlund Blauenfeldt (Aarhus University Hospital), Niels Hjort (Aarhus University Hospital), Hans Erik Bøtker (Aarhus University Hospital), David C. Hess (Medical College of Georgia, USA), Hans Kirkegaard (Aarhus University Hospital). Rikke Bay Thomsen (Aarhus University Hospital), Birgitte Forsom Sandal (Regional Hospital West Jutland, Holstebro) and Marc Fisher (Beth Israel Deaconess Medical Center, Harvard Medical School, USA).<br><br>**Data Monitoring Committee (and Trial Safety Committee):**<br>***The DMSC comprises of:***<br>Jesper Petersson (Skåne University Hospital, Sweden) – chair.<br>Jan Brink Valentin (Aalborg University Hospital, Denmark)<br>Thomas Christensen (Nordsjællands Hospital, Denmark)<br><br>**Trial Endpoints Validation Committee:**<br>EVC comprises of Independent senior consultants in Neurology and Cardiology<br>**Trial monitoring:**<br>During the entire study period and at all participating centres the regional GCP unit will perform quality assurance control including source data verification (GCP Unit Aarhus/Aalborg, ID 2017-718)
**Study centers:**<br>Department of Neurology, Aarhus University Hospital, DK-8000 Aarhus C, Denmark<br>Department of Neurology, Holstebro Hospital, DK-7500 Holstebro, Denmark

Department of Neurology, Odense University Hospital, DK-5000 Odense, Denmark<br>(Future study center, 2019 or 2020)<br>Department of Neurology. Aalborg University Hospital, DK-9000, Aalborg, Denmark<br>(Future study center, 2019 or 2020)<br>Department of Neurology, Zealand University Hospital, DK-4000 Roskilde, Denmark<br>(Future study center, in 2019 or 2020)<br><br>All Danish stroke centers will be invited to participate.
**Planned study period:** 2018-2022.
**Phase of development:**<br>Improve routine care of patients with acute stroke and transitory ischemic attack (TIA).
**Objectives:**<br>To determine whether combined remote ischemic per- and postconditioning can improve long-term recovery in acute stroke patients as an adjunct to standard treatment.
**Diagnosis:**<br>Acute ischemic stroke (AIS) and intracerebral hemorrhage (ICH).
**Methodology:**<br>A multicenter, investigator-driven, prospective, randomized, parallel assignment, patient-assessor blinded, sham-controlled clinical efficacy trial.
**Randomization:**<br>• Eligible patients will be randomized prior to the arrival at the hospital (via a secure web site).<br>• The patients and the clinical outcome assessor will be blinded to the treatment allocation.
**Number and subjects (planned):**<br>• 1,500 patients
**Inclusion criteria (prehospital):**<br>The initial treatment regime will be applied in the acute prehospital phase in setting of an acute research study<br>• Male and female patients (≥ 18 years)<br>• Prehospital putative stroke<br>• Onset of stroke symptoms < 4 hours before remote ischemic conditioning (RIC)<br>• Independent in daily living before symptom onset (mRS ≤ 2)<br>**Final in-hospital inclusion criteria**<br>• acute ischemic stroke including documented TIA<br>*or*<br>• ICH

TIA without documented ischemic lesion and non-vascular diagnosis will only have register-based long term follow-up only.

Exclusion criteria (pre-hospital)

Exclusion criteria, to be established during the teleconference between ambulance and on call neurologist

- Intracranial aneurisms, arteriovenous (AV) malformation, cerebral neoplasm, or abscess
- Pregnancy
- Severe peripheral arterial disease in the upper extremities
- AV shunt in the arm selected for RIC
- Concomitant acute life-threatening medical or surgical condition

Criteria for evaluation

Primary endpoints

- Clinical outcome (mRS) at 3 months in acute stroke (AIS and ICH)

Secondary endpoints

- Difference in prehospital stroke score (PreSS) during 24 hours in all randomized patients
- Clinical outcome (mRS) at 3 months in subgroups: AIS, IV tPA/EVT treated AIS and ICH
- Difference in prehospital stroke score (PreSS) during 24 hours in subgroups: IV tPA/EVT treated AIS and ICH.
- Difference in proportion of patients with complete remission of symptoms within 24 hours (TIA)
- Major Adverse Cardiac and Cerebral Events (MACCE) and recurrent ischemic events based on registry data at 3 and 12 months in ICH, AIS patients, TIA and non-vascular diagnosis
- Three-month and one-year mortality in AIS, ICH, and overall
- Early and very early neurological improvement in AIS and ICH patients
- Bed-day use at 3 and 12 months and quality of life measures at 3 months

Sub-studies at Aarhus University Hospital (secondary endpoints)

- Clinical outcome (mRS) at 3 months in patients with AIS and ICH and extended remote ischemic postconditioning protocol (*substudy at Aarhus University Hospital*)
- Proportion of RIC/Sham-RIC treated AIS patients with large vessel occlusion (LVO) eligible to EVT treatment (*substudy at Aarhus University Hospital*)
- Difference in acute (24-hour) hematoma expansion in patients with ICH
- Difference in hematoma reabsorption rate (7 days) in patients with ICH

- Infarct growth in AIS patients and hematoma growth in ICH patients
- Predictive abilities of Glial Fibrillary Acidic Protein (GFAP) in prehospital obtained blood samples combined with prehospital stroke severity to differentiate hemorrhagic from ischemic stroke and to grade ischemic stroke severity (*substudy at Aarhus University Hospital*)
- Diagnostic abilities of a prehospital microRNA and extracellular vesicles blood samples profile combined with prehospital stroke severity on the differentiation of hemorrhagic from ischemic stroke and to grade ischemic stroke severity (*substudy at Aarhus University Hospital*)
- microRNA and extracellular vesicle profile of RIC-induced neuroprotection at baseline (*substudy at Aarhus University Hospital*)
- Coagulation profile of putative stroke patients in prehospital obtained blood samples (*substudy at Aarhus University Hospital*)
- Modulation of coagulation by RIC (*substudy at Aarhus University Hospital*)
- Characterization of rheoerythrocrine dysfunction (RBC deformability, eryNOS3 and plasma nitrite) in RIC vs Sham-RIC treated stroke patients and its possible association to improved short term (24 hour) or long term (90 day mRS) clinical outcome or imaging biomarkers (DWI infarct growth) (*substudy at Aarhus University Hospital*)
- Prestroke physical activity level (PASE) as a predictor for early and long-term recovery. (*substudy at Aarhus University Hospital*)

Study flow chart

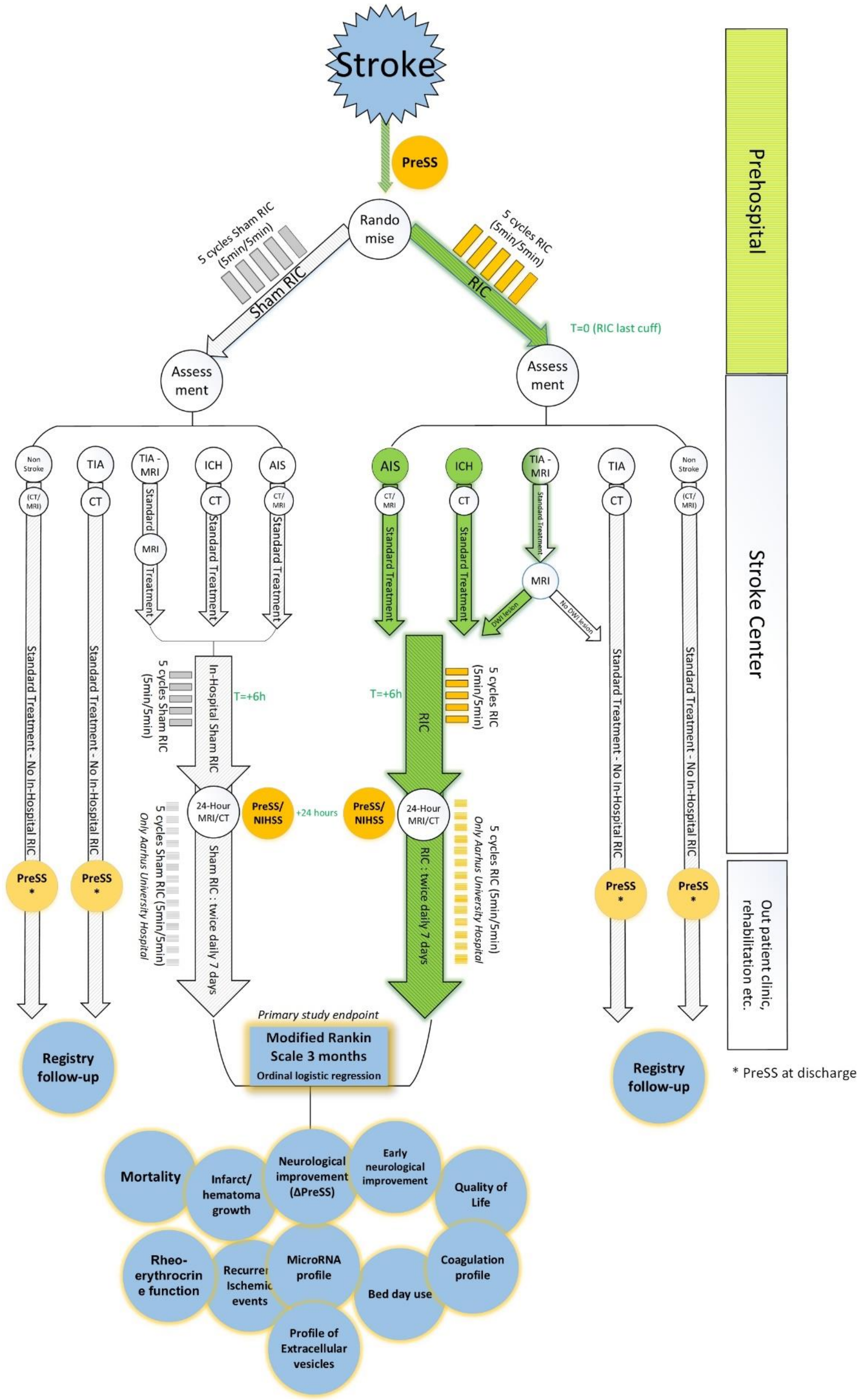

Abbreviations

AE	Adverse event
AIS	Acute ischemic stroke
AR	Adverse reaction
CT	Computed tomography
CPSS	Cincinnati Prehospital Stroke Scale
DWI	Diffusion-weighted imaging
DMC	Data Monitoring Committee and Endpoints Validation Committee
ICH	Intracerebral hemorrhage
END	Early neurological deterioration
EVT	Endovascular treatment
LVO	Large Vessel Occlusion
miRNA	Micro ribonucleic acid
mPTP	Mitochondrial permeability transition pore
MRI	Magnetic resonance imaging
NPR	Danish National Patient Register
PreSS	Prehospital Stroke Score
PWI	Perfusion-weighted imaging
RIC	Remote ischemic Conditioning
RIPerC	Remote Ischemic perconditioning
RIPreC	Remote ischemic preconditioning
RIPostC	Remote ischemic postconditioning
IV tPA	Intravenous thrombolysis/recombinant tissue-type plasminogen activator
SAE	Serious adverse event
SAR	Serious adverse reaction
TIA	Transient ischemic attack
TIA (DWI+)	Transient ischemic attack with a DWI-positive lesion on MRI
TMG	Trial Management Group
TSC	Trial Steering Group

2. Introduction

Our primary aim is to investigate whether remote ischemic conditioning (RIC) as an adjunctive treatment can improve long-term recovery in acute stroke patients as an adjunct to standard treatment.

Stroke is the second-leading cause of death worldwide and a leading cause of serious, long-term disability. The most common type is AIS which occurs in 85% of cases. Acute cerebral thromboembolism leads to an area of permanent damage (infarct core) in the most severely hypoperfused area and a surrounding area of impaired, yet salvageable tissue known as the "ischemic penumbra"[1]. Intravenous alteplase (IV tPA) and endovascular treatment (EVT) are approved acute reperfusion treatments of AIS to be started within the first 4½-6 hours and as soon as possible after symptom onset to prevent the evolution of the infarct core[2,3,4,5]. The prognosis has improved overall for ischemic stroke and the one-month mortality rate has declined to an approx. 10% [6]. However, reperfusion itself may paradoxically result in tissue damage (reperfusion injury) and may contribute to infarct growth[7,8,9]. Infarct progression can continue for days following a stroke, and failure of the collateral flow is a critical factor determining infarct growth[10]. The pre-eminent therapeutic aim is to restore blood flow, improve collateral perfusion, and prevent reperfusion injury[11]. In transient ischemic attack (TIA), the ischemia and stroke symptoms are only temporary, but these patients possess a high risk of re-stroke – especially patients with evidence of infarction on neuroimaging[12]. TIA patients with a documented ischemic lesion are, per definition, handled as ischemic stroke in this study.

On the other hand, in ICH the culprit is an eruption of blood into the brain parenchyma causing tissue destruction with a massive effect on adjacent brain tissues. Hematoma expansion as well as inflammatory pathways that are activated lead to further tissue damage, edema, and penumbral hypoperfusion[13]. The prognosis after ICH is poor with a one-month mortality of 40%. Acute blood pressure lowering is recommended to prevent further hematoma growth, but the mechanisms behind this effect are not understood[14].

Novel therapeutics and neuroprotective strategies that can be started ultra-early after symptom onset are urgently needed to reduce disability in both AIS and ICH.

Ischemic conditioning is one of the most potent activators of endogenous protection against ischemia-reperfusion injury[15]. RIC can be applied as repeated short-lasting ischemia in a distant tissue that results in protection against subsequent long-lasting ischemic injury in the target organ[16]. This protection can be applied prior to or during a prolonged ischemic event as remote ischemic preconditioning (RIPreC) and perconditioning (RIPerC), respectively, or immediate after reperfusion as remote ischemic postconditioning (RIPostC)[17]. RIC is commonly achieved by inflation of a blood pressure cuff to induce 5-minute cycles of limb ischemia alternating with 5 minutes of reperfusion. RIC activates several protective mechanisms, through humoral and neuronal pathways[18]. Circulating microRNA appears to be intimately involved in the RIC stimulus and might act as possible effector molecules[19,20].

Preclinical studies show that RIC induces a promising infarct reduction in an experimental stroke model[21,22]. It has been demonstrated that RIC protects against ischemia-reperfusion injury in the heart, lung, kidney, and the brain[22]. Results from a recent proof-of-concept study at our institution indicate that RIPerC applied during ambulance transportation as an adjunctive to in-hospital IV tPA increases brain tissue survival after one month[23]. Furthermore, RIPerC patients had less severe neurological symptoms at admission and tended to have decreased perfusion deficits[23]. Another study using RIPreC found a decreased stroke recurrence and shorter time to recovery in patients with intracranial arterial stenosis[24, 25]. Furthermore,

emerging preclinical data indicate a significant ~~acceleration of hematoma size reduction~~ increased hematoma reabsorption rate and an improved functional outcome after RIC-treated ICH[26].

To-date, no serious adverse events have been documented in RIC. The procedure has been applied in numerous cardiovascular ischemic patients and in patients suffering from cerebral hemorrhage (ICH/SAH)[23,27,28,29,30].

RIC is a non-pharmacologic and non-invasive treatment without noticeable discomfort that has first-aid potential worldwide[31]. However, whether combined remote ischemic per- and postconditioning can improve long-term recovery in AIS and ICH has never been investigated in a randomized controlled trial.

3. Hypothesis

RIC as an adjunctive treatment improves long-term functional recovery in AIS and ICH patients.

4. Objectives

Primary objectives

- To determine whether RIC improves the clinical outcome (mRS) at 3 months in AIS and ICH

Secondary objectives

- To determine whether RIC reduces neurological impairment (PreSS) in all randomized patients
- To determine whether RIC improves the clinical outcome (mRS) at 3 months in subgroups of AIS, IV tPA/EVT-treated AIS patients and ICH patients
- To determine whether RIC reduces neurological impairment (PreSS) at 24 hours in subgroups of IV tPA/EVT treated AIS and ICH.
- To determine whether RIC increases the proportion of patients with complete remission of symptoms within 24 hours (TIA; both with and without DWI lesion)
- To determine whether RIC reduces major adverse cardiac and cerebral events (MACCE) and reduces recurrent ischemic events at 3 and 12 months
- To determine whether RIC reduces the 3-month and 1-year mortality in patients with AIS, ICH, and overall
- To determine whether RIC reduces bed-day use at 3 and 12 months and affects quality of life at 3 months
- To determine whether RIC increases the occurrence of early and very early neurological improvement in AIS and ICH patients

Secondary endpoints (only Aarhus University Hospital)

- To determine whether an extended RIC protocol (one week) improves clinical outcome (mRS) at 3 months in patients with AIS and ICH (*substudy at Aarhus University Hospital*)
- To determine whether RIC Increased the proportion of AIS patients with large vessel occlusion (LVO) eligible to EVT treatment (*substudy at Aarhus University Hospital*)

- To determine whether RIC reduces infarct growth in AIS patients and hematoma growth in ICH patients (*substudy at Aarhus University Hospital*)
- To determine whether RIC increases the hematoma reabsorption rate (7 days) in patients with ICH (*substudy at Aarhus University Hospital*)
- To determine whether prehospital obtained Glial Fibrillary Acidic Protein (GFAP) in blood samples combined with prehospital stroke severity can differentiate hemorrhagic from ischemic stroke and grade ischemic stroke severity (*substudy at Aarhus University Hospital*)
- To determine whether prehospital obtained microRNA and extracellular vesicles blood samples combined with prehospital stroke severity can differentiate hemorrhagic from ischemic stroke and grade ischemic stroke severity (*substudy at Aarhus University Hospital*)
- To determine whether a circulating microRNA and/or extracellular vesicle profile of RIC-induced neuroprotection can be established (*substudy at Aarhus University Hospital*)
- To determine whether the prehospital determined coagulation profile from putative stroke patients differs between hemorrhagic, ischemic stroke and non-stroke (*substudy at Aarhus University Hospital*)
- To determine whether RIC modulates coagulation in hemorrhagic and ischemic stroke, and explore links between coagulation and infarct growth in AIS, hematoma expansion in ICH, and MACCE and recurrent ischemic events at 3 and 12 months in both AIS and ICH (*substudy at Aarhus University Hospital*)
- To characterize the rheoerythrocrine dysfunction (RBC deformability, eryNOS3 and plasma nitrite) in RIC vs Sham-RIC treated stroke patients and its possible association to improved short term (24 hour) or long term (90 day mRS) clinical outcome or imaging biomarkers (DWI infarct growth) (*substudy at Aarhus University Hospital*)
- To determine whether prestroke physical activity level (PASE) is a predictor for early and long-term recovery (*substudy at Aarhus University Hospital*)

5. Trial design

Trial design

This is a multicenter, prospective, randomized, patient-assessor blinded, sham-controlled trial investigating whether RIC improve long-term recovery in acute stroke.

Number of centers

Patients with putative stroke from participating stroke centers in Denmark will be recruited.

Number of subjects

We estimate that a sample size of 1500 prehospital putative stroke patients will be required to achieve 1000 eligible AIS and ICH patients (primary study endpoint).

Sample size determination

Primary clinical endpoint

The treatment effect of RIC on long-term functional outcome is unknown. We have assumed a small but clinical significant neuroprotective effect corresponding to a 6-7% increased odds for a beneficial shift on the modified Rankin Scale. The sample size calculation was based on a simulation-based approach to the analysis of statistical power when ordinal logistic regression analysis is performed (significans level of 5%)

The statistical power was simulated at different hypothetical sample sizes (on the target population) (ranging from 200 to 1900) with 2000 simulation-runs performed at each step. Unpublished data on IV-tPA and/or EVT treated AIS patients and ICH patients from our institution were used:

3 months modified Rankin Scale distribution (proportions) in 2017 for patients with ICH or IV tPA/EVT treated AIS at Aarhus University Hospital.

**Modfied Rankin Scale score**	**mRS 0**	**mRS 1**	**mRS 2**	**mRS 3**	**mRS 4**	**mRS 5**	**mRS 6**
Proportion	0.139	0.273	0.141	0.110	0.145	0.07	0.126

Based on our previous trial experience with prehospital remote ischemic conditioning we estimate that a sample of 1000 subjects with target diagnosis (AIS and ICH) will be feasible to include during the study period.

Including 1000 patients with target diagnosis provide sufficient power at a alfa-level (significans) of 5% to detect RIC treatment effects of the estimated 6-7 % *(see table below)*

**Treatment effect**<br>**(assumed neuroprective)**	5%	**6%**	**7%**
Sample (target diagnosis), *n*	1000	**1000**	**1000**
Power	66%	**80%**	**90%**
Alpha level (significans level)	5%	**5%**	**5%**

The estimated prehospital, randomized, sample:

**Sample size, prehospital**	**Proportion of randomized**	***n =***
**Target diagnosis (AIS and ICH), *n***	**67%**	**1000**
Non-vascular diagnosis	**27%**	**403**
TIA without DWI lesion	**4%**	**60**
Lost to follow-up	**2%**	**30**
Total		**1492**
**Plan to include**		**1500**

We therefor plan to include 1500 patients with a prehospital putative stroke in order to get 1000 patients with the target diagnosis of acute ischemic stroke and intracerebral hemorrhage. There is no planned replacement of patients lost to follow-up.

Randomization

Randomization procedure

The patient will be randomized to standard treatment with RIC or sham-RIC by the on call neurologist/vascular neurologist at the receiving stroke center. The ambulance will contact the on call neurologist by telephone and describe the patient (standard operating procedure (SOP) in Denmark). The randomization is based on a secure web site providing computer-generated blocked randomization lists stratified by the center. The online randomization is stratified by age, strokecenter and the Prehospital Stroke Score (PreSS). The PreSS score consists of the Cincinnati Prehospital Stroke Scale (CPSS) with an additional opportunity to report other neurological symptoms (e.g. ataxia, sensory disturbances and visual field loss), and PASS (Prehospital Acute Stroke Severity Scale)[32,33]. Prehospital personnel participating in RESIST are trained in identifying stroke symptoms included in PreSS. The on call neurologist will make an assessment based on all available information whether the patient is eligible to participate in RESIST. Randomization is performed in the prehospital setting. Each on call neurologist participating in the study will receive unique access and will have no influence on the randomization process. All neurologists on call will be educated and trained in performing evaluation of potential eligible study candidates and to perform the online randomization during the telephone call with the ambulance personnel. There is always one neurologist on call in participating centers (hospital SOP).

Treatment allocation

1:1 Allocation

6. Selection and withdrawal of subjects

Selection of patients

All patients with a putative stroke who meet the study criteria will be included.

- Depending on the prehospital randomization (RIC versus sham-RIC), patients with AIS will continue RIC or sham-RIC treatment for an extended period. This group includes TIA with a documented DWI lesion on magnetic resonance imaging (MRI).
- Patients with ICH will continue in-hospital RIC or sham-RIC (according to randomization).

Informed consent for prehospital enrollment

Background:

Acute temporary cognitive impairment after stroke is very common, even in patients with minor stroke or remission of symptoms (TIA) (54%) [34]. Widespread cognitive deficits have been identified in an acute stroke patient population[35]. This finding is in line with the clinical experience in acute stroke care. Whether the putative stroke patients have major cognitive impairment in the prehospital setting can only be tested under calm and quiet in-hospital circumstances. Delivering acute neuroprotective care, it is of utmost importance that treatment is started as soon as possible, i.e. without delay. RIC is without any known serious adverse events.

According to the Danish research ethics committees, a patient who is a candidate for inclusion in an acute research study is considered incompetent if that person is not able to care for his or her own affairs due to physical or mental impairment. We believe that the vast majority of stroke patients fulfil these criteria, and it is not possible to select the few who are, indeed, competent in the acute phase without formal cognitive testing and thus missing the purpose of acute neuroprotection intervention. The intervention is associated only with mild to moderate discomfort and minimal risk for adverse events.

Acute Prehospital phase:

The initial RIC/sham-RIC treatment regime will be applied in the acute prehospital phase in setting of an acute research study approved by the regional research ethics committees.

Whether further treatment, according to randomization, will be offered depends on the in-hospital examination.

In-hospital inclusion:

After the initial RIC/sham-RIC treatment, in-hospital diagnostic evaluation, and acute treatment, ***the competent patient*** will be presented with a consent form with information about the study. Before this, the on-duty neurologist/research physician will have examined the patient both physically and cognitively and will have assessed whether the patient is competent or not. The competent patient will receive oral information by the on-duty neurologist/research physician based on the written patient information. The written patient information will be handed out (*deltager information*). We will ask for the patient's acceptance to be included in the study. The patient can withdraw consent at any time. In addition, the acute nature of the disease necessitates circumvention of the usual requirements concerning a 24-hour time period for consideration and discussion with a lay representative. This has been done in recent comparable acute studies. This approach is acceptable as the intervention is without any known risk and associated with a potential benefit for the patient. In-hospital inclusion and assessment of cognitive impairment will be undertaken during the admission at the stroke center. Every effort will be made to inform the patients and relatives in quiet and undisturbed settings, this will be at the hospital bedroom or the designated room for conversations. We will prioritize the presence of nearest relatives at time of study information and inclusion.

For ***the incompetent patients***, informed consent will be obtained from next of kin and an independent physician. The consenters will be provided information (based on the summary of the protocol for the independent physicians and study participant information for the relatives) on the trial to be able to make an informed decision about the patient's participation in this trial. The independent physician will be the on-duty physician at the local department of neurosurgery, neuroanesthesiology, intensive care, neurophysiology, (neuro)radiology or the on duty physician from another specialty with stroke patient experience. On-duty physicians at these departments will receive detailed study information.

A patient who during the acute phase was legal incompetent and included as such, but during the follow-up period is assessed competent will receive oral and written information and we will ask for his/her's informed consent.

Patients who fulfill both prehospital and in-hospital inclusion criteria will, according to randomization, receive RIC/sham-RIC treatment again after +6 hours (***all centres***).

Participation in the extended remote ischemic conditioning(RIC) or sham-RIC (*for a total of 7 days*) study will take place ***only at Aarhus University Hospital***. The device will follow the patient when he/she is discharged from the stroke center.

TIA patients (without DWI lesion) and non-stroke diagnosis patients will be asked for consent to do registry follow-up until one year after discharge. For these patients end-of-study visit is registered at stroke center discharge.

Inclusion criteria (prehospital)

- Male and female patients (≥ 18 years)
- Prehospital putative stroke
- Onset of stroke symptoms < 4 hours before RIC
- Independent in daily living before symptom onset (mRS ≤ 2)

Exclusion criteria (prehospital)

- Intracranial aneurisms, AV malformation, cerebral neoplasm or abscess
- Pregnancy*
- Severe peripheral arterial disease in the upper extremities
- Concomitant acute life-threatening medical or surgical condition
- AV shunt in the arm selected for RIC

**Women of child-bearing age should be asked about their use of safe birth control methods (contraceptive pill, intrauterine devices both hormonal and non-hormonal, hormonal implants, hormonal depot injection and transdermal hormonal patch). If pregnancy cannot be ruled out in the prehospital phase the patient can't be included. Women with a safe birth control method should be encouraged to use this method during the entire period of active RIC treatment.*

Final in-hospital inclusion criteria

- AIS (including DWI-positive TIA patients)

  or

- ICH

TIA and non-stroke diagnose will only have long-term register based follow-up.

Method of blinding

Outcome assessment is blinded to the treatment arm and obtained by a clinical or telephone assessment of the level of dependency and need for help in daily activities (**modified Rankin Scale**) **(Primary study endpoint**).
No information regarding randomization status will be recorded in the patient record.
Patients and the assessors of endpoints are blinded.

Discontinuation of study participation

A patient can withdraw from the study at any time. Patients can be withdrawn from the study at the principal investigator's discretion. In case the patient cannot be reached by telephone, every effort will be made to contact the patient or to document the outcome regarding new vascular events via

registries/electronic health records. If the patient withdraws from the study, the date and the reason for the patient's withdrawal will be recorded. The patient is encouraged to provide information about his or her reason(s) for withdrawal and any experienced adverse effects (AE) during the study.

7. Study procedure and assessment

Prehospital stroke identification

Prehospital putative stroke patients are identified in collaboration with the ambulance personnel and the on-duty neurologist/vascular neurologist at the nearest stroke center (by telephone), which is standard operating procedure in Denmark. All Danish ambulance personnel are trained in identifying putative stroke in the prehospital setting. In the present study protocol, all patients have a prehospital structured assessment of neurological symptoms/findings (based on items contained in PreSS) before randomization – which will further increase stroke symptom awareness.

Baseline data

Baseline data and process indicators are collected from the Danish Stroke Registry (DSR). Additional information about medical history and medication and clinical and physiological data are collected at baseline. Data are recorded in an electronic case report form (CRF) at discharge from the stroke unit. For a complete list of registered data, see *Supplement B*

Acute treatment:

Patients are treated according to the national clinical guidelines on stroke treatment, intravenous thrombolysis and endovascular therapy. All patients are treated with state-of-the-art multidisciplinary care in the setting of a stroke unit monitored by the DSR.

Blood samples

Patients with putative stroke admitted to the Aarhus University Hospital will have blood samples withdrawn, in the ambulance, from peripheral venous catheter (placed as standard operating procedure, SOP). A total of 19mL (9mL + 10mL) will be drawn in the prehospital phase.

Blood samples will also be drawn upon admission to the Aarhus University Hospital Stroke Center as a part of SOP. Additional 19mL (9mL + 10mL) of blood will be drawn at this time.

Patients with AIS and ICH diagnosis will have blood-samples drawn, at Aarhus University Hospital Stroke Center, again 24 (16-32) hours after randomization. This is not standard operating procedure. 19mL (9mL + 10mL) will be drawn at this time.

Only patients received at the Stroke Center at Aarhus University Hospital will have blood samples drawn. (Prehospital and in-hospital blood samples)

Not all ambulances will be equipped with for blood sample withdrawal, thus an estimated 500 patients will have blood samples drawn in the prehospital phase and in hospital at Aarhus University Hospital.

Blood samples will be stored until completion of the trial in a research biobank.

Patients not admitted at Aarhus University Hospital will have no additional blood samples drawn (neither prehospital or in-hospital)

MicroRNA, extracellular vesicles, Glial Fibrillary Acidic Protein (GFAP), RBC deformability, NOS3, plasma nitrite and coagulation analysis will be performed on the obtained blood. A research bio bank will be established, and all blood samples obtained during the study are stored here. During the entire study and analysis period, the material will be stored at Aarhus University Hospital. The last patient will be included in December 2022. Analysis of blood samples, according to secondary endpoints, will be performed during the study and until 2 years after inclusion of the last patient (no later than 31. December 2024). Hereafter the remaining material will be transferred to a biobank for future unspecified research purposes. We will submit an application to the Danish Data Protection agency for this additional 20-year storage period of the blood samples (until 31 December 2044), after that, the samples will be destroyed. All analysis will be performed in Denmark.

Investigational devices and Remote ischemic conditioning protocols

Automatic remote ischemic conditioning devices

Both the RIC and sham-RIC device are developed in collaboration with Aarhus University, Faculty of Biomedical Engineering, 8200-DK, Aarhus N, Denmark; Seagull Aps, 4200-DK, Slagelse, Denmark and the Department of Neurology, Aarhus University Hospital, 8000-DK, Aarhus C, Denmark.

The devices are manufactured by Shenzhen Raycome Health Technology Co., Ltd, 3F, 51 Building, No.5 Qiong Yu Road, Hi-Tech Industrial Park, Nanshan District, Shenzhen, China 518057.

The ***Investigator's Brochure (IB)*** *contains a detailed description of the investigational devices including risk analysis assessment.*

A brief user's manual is found in each device bag. No preexisting experience is necessary in order to operate the device. Written and oral information combined (Healthcare professional at participating hospitals) with RESIST trial e-learning material (prehospital personnel) will be offered to familiarize the clinical personnel in using the investigational devices. Patients and relatives will be instructed using written and oral information.

Remote Ischemic Conditioning device (RIC device)

The device is programmed to five cycles (50 minutes), each consisting of five minutes of cuff inflation followed by five minutes with a deflated cuff. The cuff pressure will be 200mmHg; but if initial systolic blood pressure is above 175 mmHg, the cuff is automatically inflated to 35 mmHg above the systolic blood pressure. This is done to account for a maximum cuff air leakage of 6mmHg per minute and maximum deviation in bloodpressure measurement of 10mmHg. The maximum cuff pressure is 285 mmHg.

Sham – remote ischemic conditioning device (Sham-RIC device)

The device is programmed to five cycles (50 minutes), each consisting of five minutes of cuff inflation followed by five minutes with a deflated cuff. The cuff pressure during inflation will be 20mmHg.

Timestamps, blood pressure before RIC/sham-RIC and total RIC/sham-RIC cycles are recorded and stored on the device (accessible by *universal serial bus,* USB). The RIC and sham-RIC is pre-programmed before

being delivered to collaborating centers/ambulances. No data on conditioning cuff pressure will be displayed on device screen.

In cases of short transport time, the RIC/sham-RIC protocol continues during the initial assessment at the stroke center. RIC/sham-RIC stimulation is discontinued just prior to the MRI and continues afterwards if the protocol was incomplete (< 5 cycles). In centers using CT, the RIC/sham-RIC protocol is continued during the scan until a total of 5 cycles are reached. The device automatically stops treatment when the 5 cycles are completed. Except for RIC/sham-RIC, the prehospital observation and treatment are according to standard procedures in the ambulance.

Stroke RIC/Sham-RIC will be used, only if approved by the Danish Medicines Agency

The blood pressure and heartrate measurement, by RIC and Sham-RIC, will only be used for research purposes.

Initial remote ischemic conditioning

(all patients)

RIC/sham-RIC cuff will be placed on the non-paretic upper arm. The cuff is placed on the upper extremity on the same side of the suspected side of cerebral stroke, e.g. in right hemiparesis the cuff on the left arm, and in monosymptomatic aphasia the cuff is placed on the left arm.

Remote ischemic conditioning at +6 hours

(All patients with AIS, ICH – all centers)

At 6 hours after the first RIC/sham-RIC protocol (6 hours from the last cuff-off, respectively), another series of RIC/sham-RIC is performed; five cycles each consisting of 5 minutes of ischemia are followed by 5 minutes of reperfusion.

Remote ischemic <u>post</u>conditioning (twice daily for 7 days)

(AIS and ICH – only at Aarhus university Hospital)

The post-conditioning stimulus will be applied twice daily in the first week. This occurs at pre-specified times: 08.00 PM and AM. However, RIC/sham-RIC can be applied no sooner than 10 hours and not later than 21 hours after the initial perconditioning stimulus (last cuff).

Aarhus University Hospital: RIC/sham-RIC treatment will continue for a total of 7 days. If patients are discharged to their home or to another department, they will continue treatment according to written instructions, including application of the device for 7 days. After RIC/sham-RIC on day 7, the device is then returned to the Stroke Center.

**RIC/sham-RIC protocol**	**Accepted time frame**
**Prehospital RIC**	< 4 hours from symptom onset
**RIC at +6 h**	4 to 8h from last cuff of the initial RIC
**RIC twice daily for 7 days** – 08.00 PM and AM*	06.00 to 10.00 PM and 06.00 to 10.00 AM

*RIC/sham-RIC can be applied no sooner than 10 hours and no later than 21 hours after the initial perconditioning stimulus.

Device Accountability log

All devices are registered in a *device accountability log*. When the prehospital personnel arrives at the stroke center, with a patient included in RESIST trial, a new device (of the same color) will be handed out. Device ID, ambulance ID, contact details to the ambulance and date of device hand-out will be registered. It will be recorded on a paper-log in the stroke department. These data are transferred to an electronic log keeping track of device location and time to service/battery charge (1 year). *Further details see Investigators Brochure.*

Study procedures table

	Acute prehospital and in-hospital phase					+ 6 hour	+ 24 hour			+ 7days		+3 months	12 months
	Prehosp. stroke score (PreSS)[1]	Prehosp Blood-samples[1]	RIC/Sham-RIC	In-hospital assessment[2]	CT/MRI baseline	+6 hour RIC/sham-RIC [4]	PreSS 24h [2]	CT/MRI 24h[3]	24-hour Blood-samples	Extended (7 days) RIC/sham-RIC [5]	CT- 7 days[3]	mRS 3-months[7]	Clinical events during follow-up[6]
TIA (DWI-) - all	X	(X)*	X	X	X		X						X
Non-stroke - all	X	(X)*	X	X	X		X						X
AIS incl. TIA (DWI+) – Other¥	X		X	X	X	X	X	X				X	X
ICH - Other¥	X		X	X	X (CT)	X	X					X	X
AIS incl. TIA (DWI+) – AUH*	X	(X)	X	X	X(MRI)	X	X	X (MRI)	(X)	X		X	X
ICH – AUH*	X	(X)	X	X	X	X	X	X (CT)	(X)	X	X (CT)	X	X

***AUH = Aarhus University Hospital , ¥ Other= Other participating stroke centers*

[1] Prehospital stroke score (PreSS) and bloodsamples. *Prehospital blood samples (*19mL*): only patients transported to Aarhus University Hospital)*

[2] Acute assessment at the Stroke Center. NIHSS, blood samples (19mL blood) and ECG (standard). Assessment of neurological symptoms at 24-hour: NIHSS. Patients with non stroke diagnosis and TIA without a DWI-MRI lesion, who are discharged before 24 hours will be scored using PreSS at discharge. Patients with vascular diagnosis, who had blood samples taken in the ambulance and upon stroke center arrival will have additional samples withdrawn at 24 hour (additional 19mL blood). *In-hospital RESIST blood samples only at Aarhus University Hospital*

[3] Baseline CT is preferable for ICH patients (hospital SOP). ICH patients, at Aarhus University Hospital, will receive a 24 hour control CT (hospital SOP). If possible, a 24-hour MRI is performed in all included AIS patients with an MRI baseline scan. Furthermore, a 24-hour control CT will be provided for IV tPA-treated AIS patients, unless MRI can be performed and was the primary investigation. One week control CT will be performed, if possible, on ICH patients included at Aarhus University Hospital. End of study visit for non-vascular diagnosis and TIA (without DWI lesion) at stroke center discharge.

[4] RIC/sham-RIC at +6 hours from last RIC cuff

[5] RIC/sham-RIC twice daily for 7 days *(Only at Aarhus University Hospital)*

[6] Mortality, MACCE, and recurrent ischemic events are recorded using the Danish National Patient Register (LPR) and DSR at 6 and 15 months after the inclusion of the last patient.

[7] Assessments at 3 months by telephone or face-to-face (mRS and WHO-5). End-of-study (telephone or face-to-face) for AIS and ICH patients. WHO-5 will only be assessed by one assessor.

8. Endpoints

An independent end-point committee will adjudicate clinical events.

Primary endpoints

Criteria for evaluation

Clinical outcome (mRS) at 3 months in acute stroke

Primary study endpoint: Clinical outcome (modified Rankin Scale) at 3 months in acute stroke patients (generalized ordinal logistic regression)

The level of dependency and need for help in daily activities will be determined by either face-to-face assessment or based on a structured telephone interviews performed by assessors who are blinded to the intervention at 3 months after the index stroke.

The assessment will be performed by two independent blinded assessors, which can be either face-to-face or telephone based assessment (not same day). The electronic CRF database will alert if disagreement occurs, and the patient will be contacted by a third assessor (face-to-face or telephone) who is blinded to the intervention who will assess the level of dependency.

- If disagreement occurs between **two telephone assessments** – a third, and final, telephone or face-to-face assessment will be made.
- If disagreement occurs between one face-to-face assessment and one telephone assessment – the face-to-face will be considered the final assessment
- If disagreement occurs between **two face-to-face assessments** – a third, and final, telephone or face-to-face assessment will be made.

Every possible effort will be made to assess the outcome in patients who are unable to participate in the interview (this could be due to post stroke neurological impairment (for example aphasia and/or dysarthria), or that the patient cannot be reached by telephone). The outcome will then be assessed by contact to a named relative or the general practitioner. Several attempts to contact the patient by telephone will be made before contacting the named relative or general practitioner.
Death in the follow-up period will be obtained from Danish Civil Registration System (CPR).

The structured telephone interview will be based upon a validated Danish translation of the "slightly revised simplified, modified Rankin Scale questionnaire"[36]. The translation and its validation will be performed according to the AAOS guideline[36, 37]

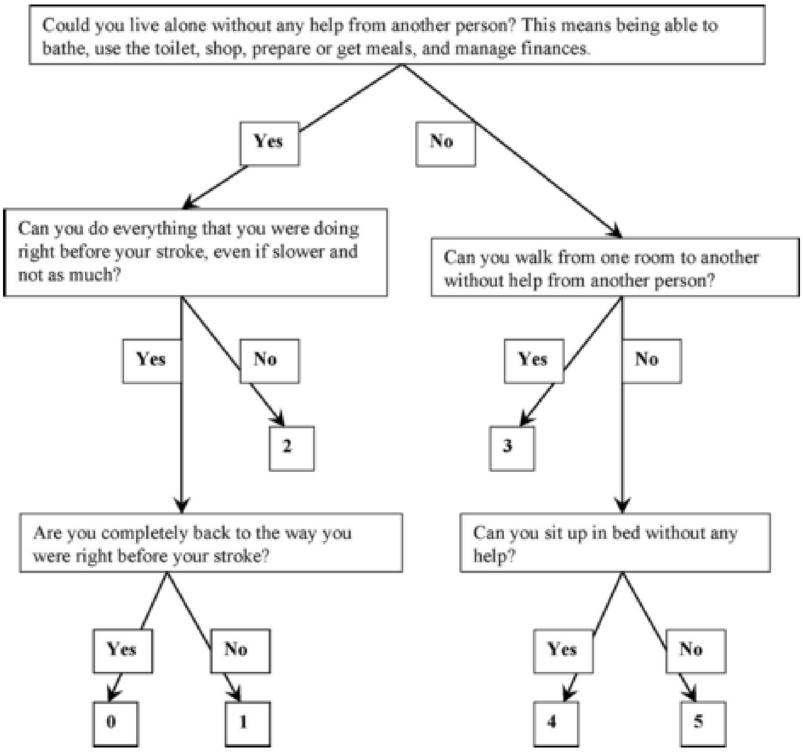

Bruno et al. 2011. Stroke.

Furthermore, modified Rankin Scale assessors are required to have received specific mRS training, which could be an online mRS certification https://secure.trainingcampus.net/uas/modules/trees/windex.aspx?rx=rankin-english.trainingcampus.net

For a **detailed statistical analysis plan**, see **statistics**

Outcome	Accepted time frame (from symptom onset)
3-month follow-up – 1st and 2nd assessment	3 months +/- 2 weeks
3 month follow-up – 3rd assessment	3 months and 2 weeks +/- 2 weeks

Secondary endpoints

Difference neurological impairment during the first 24 hours in all randomized patients

Neurological deficits are documented using PreSS (prehospital and in-hospital). The PreSS score is obtained by the prehospital personnel and registered by the on-call neurologist before randomization. Prehospital Stroke Score is assessed at 24-hour by a certified, trained and blinded general neurology nurse/neurology research nurse/physician/neurologist who is blinded to the treatment intervention and who is not a part of the daily stroke ward routine. Only information regarding time and place of assessment, patient name, age

and social security number will be available. The PreSS score of patients with TIA (without DWI lesion) and non stroke diagnoses, are performed by the on-call physician/neurologist or a research nurse/general neurology nurse, and will be documented as *PreSS at discharge*, if discharge occurs before 24 hours. The outcome assessor will be alerted in advance of the assessment. The PreSS is recorded in the electronic CRF. Clinical assessors are required to complete an online PreSS certification or face-to-face training.

https://ekurser.rm.dk/test_projekter/anhesr/red-hjernen/scorm/Scorm2004%204th%20Edition/index.html

For a **detailed statistical analysis plan**, see **statistics**

**Indicator**	**Accepted timeframe (from symptom onset)**
PreSS	Close to symptom onset
$PreSS_{24h}$ (+ 24h)	+ 16 to 32h

Difference in proportion of patients with complete remission of symptoms within 24 hours (TIA; both with and without DWI)

Diagnosis of TIA is documented in the electronic case report form

**Endpoint assessment**
Difference in proportion of patients with complete remission of symptoms within 24 hours (TIA; both with and without DWI)

Major adverse cardiac and cerebral events (MACCE) and recurrent ischemic events at 3 and 12 months in AIS and all patients

MACCE is defined as:

Cardiovascular events (cardiovascular death, myocardial infarction, acute ischemic or hemorrhagic stroke)

Cardiovascular death: Death from known cardiovascular cause or sudden death from unknown cause (no identified cause of death in medical history and/or autopsy)

Acute myocardial infarction: Admission with a discharge diagnosis of ST-elevation myocardial infarction (STEMI) and non-ST elevation myocardial infarction (NSTEMI) and unstable angina pectoris (UAP)

Stroke: Admission with a discharge diagnosis of acute ischemic or hemorrhagic stroke.
Evaluation is performed using the Danish National Patient Register (LPR) and the DSR at two time points (6 and 15 months after the inclusion of the last patient).

Diagnosis of AIS/TIA, ICH and MI (STEMI, NSTEMI, and UAP) are made according to national clinical practice guidelines.

(http://neuro.dk/wordpress/nnbv/om-iskaemisk-apopleksi/ and http://nbv.cardio.dk/aks)

Recurrent ischemic vascular events at 3 and 12 months in AIS patients

Recurrent ischemic vascular events defined as:

- AIS
- TIA
- MI, STEMI, and NSTEMI

Three-month and one-year mortality

Information about mortality is collected using the CPR and LPR, at two time points (6 and 15 months after the inclusion of the last patient). All-cause mortality is assessed and subdivided into cardiovascular mortality versus non-cardiovascular mortality[40].

Analysis are performed on all randomized patients and according to subgroups.

Mortality (all-cause)

Cardiovascular mortality

- Acute MI
- Sudden cardiac death
- Heart failure
- Stroke: death as a direct consequence or complication of stroke
- Cardiovascular procedure or procedure
- Cardiovascular-related hemorrhage: non-stroke intracranial hemorrhage (subdural hematoma), non-traumatic vascular rupture (e.g. aortic aneurysm), or hemorrhage causing cardiac tamponade

Non - cardiovascular mortality

- Pulmonary, renal, gastrointestinal, hepatobiliary and pancreatic
- Infection and inflammatory/immune (including autoimmune)
- Hemorrhage (non-cardiovascular (CV)/non-stroke-related bleeding)
- Non-CV procedure or surgery, trauma, suicide, drug reaction, or overdose
- Neurological, malignancy, other non-CV

According to “2014ACC/AHA Key Data Elements and Definitions for Cardiovascular Endpoint Events in Clinical Trial”. Circulation 2015;132:302-361.

EVT-eligibility (MRI assessed) in RIC treated AIS patients with large vessel occlusion(LVO)

EVT eligibility is assessed upon arrival at the Aarhus University Hospital in AIS patients with:

- Severe Stroke (NIHSS ≥ 10)
- Groin puncture feasible within 6 hours from stroke onset
- MRI-TOF (time-of-flight) documented internal carotid artery (ICA), Intracranial ICA (ICA-T) and first and second stem of the middle cerebral artery (M1 and M2, respectively)
- No contraindications to MRI (pacemaker, vomiting, respiratory insufficiency, obesity)
- MRI-DWI lesion volume ≤ 70mL

Endpoint assessment

Proportion of RIC treated AIS patients with LVO eligible to EVT treatment compared to standard treatment, adjusted for prehospital stroke severity (PreSS) and symptom duration

Difference in MRI-DWI lesion volume in RIC/sham-RIC treated LVO AIS patients eligible to EVT treatment

Quality of life measures at 3 months in AIS and ICH patients

- **Quality of life and bed-day use measures in AIS and ICH patients**

Quality-of-life measurements are assessed by telephone interview at 3 months after inclusion. The WHO-5 is used. Baseline WHO-5 is only obtained at Aarhus University Hospital.

Link to: Danish National Board of Health - WHO-5.
Information about bed-day use via the LPR and the DSR.

Early neurological improvement in AIS and ICH patients

Neurological deficits are documented with the PreSS and the National Institute of Health Stroke Scale (NIHSS), prehospital and in-hospital, respectively.

- **Very early neurological improvement** is defined as:

Reduction in prehospital stroke score (PreSS) ≥ 1 points or resolution of symptoms at admission: Items on PreSS related to NIHSS baseline $\Delta PreSS = PreSS_{prehospital}$ – same items on $NIHSS_{baseline}$

- **Early neurological improvement** is defined as:

Reduction in NIHSS ≥ 4 (baseline versus 24-Hour NIHSS): $\Delta NIHSS = NIHSS_{baseline} – NIHSS_{24}$

- **Reduction in prehospital stroke score (PreSS) ≥ 1 points or resolution of symptoms after 24 hours in subgroups:** IV tPA/EVT treated AIS and ICH.

Clinical assessors are required to complete an online PreSS certification

PreSS certification: https://ekurser.rm.dk/test_projekter/anhesr/red-hjernen/scorm/Scorm2004%204th%20Edition/index.html

**Indicator**	**Accepted timeframe (from symptom onset)**
PreSS	Close to symptom onset
NIHSS baseline	0 to 6 hours from admission
NIHSS (+ 24h) – standard operating procedure	+ 16 to 32h
PreSS (+ 24h)	+ 16 to 32h

Secondary endpoints (Only Aarhus University Hospital)

Difference in acute hematoma expansion in patients with ICH

(Substudy only at Aarhus University Hospital)

- Hematoma expansion is defined as the difference in volume between the baseline and the 24-hour CT hematoma volume. Significant hematoma growth is defined as an absolute growth exceeding 6 mL or a relative growth of more than 33% from the initial CT[39].

In all ICH patients, the baseline imaging modality is CT. In cases of initial MRI, a CT is performed afterwards (no later than < 3 hours from last RIC cuff)

**Imaging protocol**	**Accepted time frame**
CT baseline – ICH	< 3 hours from last RIC/sham-RIC cuff
MRI baseline – ICH	Perform CT as soon as possible and < 3 hours after the last RIC/sham-RIC cuff
Control CT (24-hour) – ICH	18-30 hours from baseline CT

Information about imaging protocols, see *Supplement A – Neuroimaging protocols*

Difference in 7 days hematoma reabsorption rate in patients with ICH

(Substudy only at Aarhus University Hospital)

- Hematoma reabsorption rate is defined as the difference in hematoma volume between the baseline and the 7-day CT hematoma volume.

In all ICH patients, the baseline imaging modality is CT. In cases of initial MRI, a CT is performed afterwards (no later than < 3 hours from last RIC cuff)

Imaging protocol	Accepted time frame
CT baseline – ICH	< 3 hours from last RIC/sham-RIC cuff
MRI baseline – ICH	Perform CT as soon as possible and < 3 hours after the last RIC/sham-RIC cuff
Control CT (7-day) – ICH	5-9 days from baseline CT

Information about imaging protocols, see *Supplement A – Neuroimaging protocols*

Infarct growth in AIS patients

(Substudy only at Aarhus University Hospital)

Acute infarct growth is defined as the difference in infarct volume between baseline and 24 hours on DWI-MRI. All patients with IV-tPA and/or EVT treated ischemic stroke and a baseline MRI will have an additional 24-hour MRI performed.

Imaging protocol	Accepted time frame
MRI baseline – AIS IV tPA eligible	On admission
MRI baseline – AIS not IV tPA eligible	< 6 hours from last RIC/sham-RIC cuff
MRI (24-hour) – AIS IV-tPA/EVT treated	18-30 hours from baseline MRI

Information about imaging protocols, see *Supplement A*

Biochemical profile of stroke subtypes and of RIC-induced neuroprotection

(Substudy only at Aarhus University Hospital)

Prehospital biochemical substudies (Aarhus University Hospital)

- Diagnostic abilities of a prehospital microRNA and extracellular vesicles blood samples profile combined with prehospital stroke severity to differentiate hemorrhagic from ischemic stroke and to grade ischemic stroke severity
- Predictive abilities of Glial Fibrillary Acidic Protein (GFAP) in prehospital obtained blood samples combined with prehospital stroke severity to differentiate hemorrhagic from ischemic stroke and to grade ischemic stroke severity

- Coagulation profile in prehospital obtained blood samples to differentiate between hemorrhagic stroke, ischemic stroke, and non-stroke
- Red blood cell rheo-erythrocrine dysfunction in acute stroke and its possible association to improved early (24 hour) or long term (90 day mRS) clinical outcome or imaging biomarkers (DWI infarct growth) (*substudy at Aarhus University Hospital*)

In-hospital biochemical substudies

- Characterization of a possible microRNA and extracellular vesicle profile in stroke subtypes
- MicroRNA and extracellular vesicle characterization of a possible RIC treatment profile
- Modulation of coagulation by RIC in hemorrhagic and ischemic stroke
- Characterization of rheoerythrocrine dysfunction (RBC deformability, eryNOS3 and plasma nitrite) in RIC vs Sham-RIC treated in acute stroke patients

Prehospital and in-hospital handling of blood samples:

Blood samples will be collected from patients with a prehospital putative stroke. Thus, blood samples will be drawn in the ambulance and upon arrival at the stroke center (only Aarhus University Hospital). In the ambulance, the blood sample will be obtained through a peripheral venous catheter (placed as standard operating procedure) into a 9mL and 10mL Sarstedt Monovette® tubes and transported to hospital at ambient temperature (Tubes of 9 and 10mL will be drawn). The local biochemical department or study personnel will centrifuge the blood and divide the plasma into cryotubes that will be kept at 80 °C for long-term storage. Cryotubes not used to measure GFAP within 5 to 7 days will be stored in a research biobank.

Blood samples will be drawn upon admission to the Aarhus University Hospital Stroke Center as a part of local stroke center SOP. Additional 19mL (9+10mL) of blood will be drawn at this time.

Patients with AIS and ICH diagnosis will have blood-samples drawn, at Aarhus University Hospital Stroke Center, again 24 (16-32) hours after randomization (19mL (9+10mL)) will be drawn at this time).

All blood samples obtained during the study will be stored in a research biobank

Blood-samples, in the research biobank, will be stored until completion of the trial (no later than 31. December 2024). Hereafter the remaining material will be transferred to a biobank for future unspecified research purposes (see section "Blood Samples" under "study procedure and assessment")

Planned biochemical analysis

Glial Acidic Fibrillary Protein (GFAP):

All plasma samples for GFAP measures will be analyzed with ELISA essay kits from the same batch at Clinical Biochemical Department, Regional Hospital Herning. Transport and shipment of samples will be done on dry ice to assure stability of the specimens. The samples will be marked with the unique research identification number. Cryotubes not used to measure GFAP will be stored as part of a biobank.

MicroRNA analysis:

MicroRNAs will be identified with Illumina next-generation sequencing using the TruSeq Small RNA Sample Preparation kit (Illumina) which allows for the addition of unique barcode sequences to each sample. Such barcoding allows pooling and simultaneous sequencing of multiple samples in a single-sequencing run on

the Illumina NextSeq500 sequencer thereby significantly reducing the cost of sequencing. Pooling of multiple samples will generate adequate data amounts for detailed miRNA profiling, yet at limited costs. Data generated from the NextSeq500 sequencer will be filtered based on sequence quality as part of our established bioinformatics pipeline. This will also include matching the filtered data to annotated RNA databases such as miRNA sequences from miRBase (mirbase.org).
The output from our bioinformatic pipeline will be quantitative miRNA expression levels for each sample, which will form the basis for an miRNA differential analysis where miRNAs with statistically significant expression changes will be found.
The microRNA (and extraceullar vesicles) analysis is not a mapping of the genome. The microRNA profile is a snapshot of microRNA activity at the specific time of the blood sampling. Levels of microRNA are changing constantly and with the current knowledge on the topic it is impossible to predict a risk of developing specific diseases in the future. Cryotubes not used to microRNA/exosome analysis will be stored as part of a biobank.

Extracellular vesicle analysis:

Extracellular vesicles (EVs, also known as exosomes) will be isolated from plasma samples before characterization of surface markers and content. EVs are isolated by a number of different techniques, ultra centrifugation, precipitation, size exclusion chromatography among others. While protein characterization will be done using classical molecular biological techniques such as ELISA and Western blots in addition to array techniques, EVarray that utilises a panel of antibodies directed against known EV surface markers. These analyses might be supported by proteomic analysis of all proteins as well as post-translational modifications such as phosphorylations and glycosylations.
In addition, the nucleic acid (DNA and RNA including miRNA) content of EVs will be analysed using next generation sequencing (NGS), qRT-PCR, and other nucleic acid detection techniques. Both the protein data and the nucleic acid data will be subjected to bioinformatic analysis using different pipelines and analysis tools depending on the dataset and the purpose of the analysis. A number of validated published databases will be used for annotation and comparison. EVs do not contain genomic DNA and the analysis are therefore not a mapping of the subjects genome. As EV secretion and nucleic acid content in these are though to change rapidly in the body due to external and internal signals and are considered a snapshot at the specific time of blood sampling. With the current knowledge on the topic it is impossible to predict a risk of developing specific diseases in the future.

Coagulation assays:
Functional and immunologic plasma assays will be employed to analyze proteins and pathways in coagulation and fibrinolysis. The analyses will be performed in the accredited clinical laboratory and the thrombosis and hemostasis research unit at the Department of Clinical Biochemistry, Aarhus University Hospital. Plasma samples will be stored in cryotubes at -80 °C until they will be analyzed in large batches.

Ektacytometry for Erythrocytic Deformability:
The rheological properties of RBCs are quantified using a Deformability or Elongation Index (DI or EI). A higher EI at the optimum viscosity (300 Osmolality) indicates highly deformable RBCs indicative of better microcirculation, while a lower EI indicates rigid and fragile RBCs resulting into impaired microcirculation and tissue hypoxia. Briefly, 6-uL of heparinized fresh blood is mixed with 600-uL of PVP solution (300 Osm)

and transferred to a disposable kit (K01 Deformability Test Kit; RheoMeditech, South Korea). The kit is placed inside the laser-assisted ektacytometer (RheoScan AnD300, RheoMeditech, Seoul, S. Korea) for automated read out, data and image collection as per the vendor's instructions.

Analytical Flow Cytometry (FC) for eryNOS3 phosphorylation (pNOS3Ser1177) and s-nitrosylation (–SNO) in RBCs:
Red blood cells are separated from 50-uL of freshly fixed blood samples, using a cocktail of monoclonal antibodies (mAb) to RBC-specific markers (Glycophorin A, GPA; and Hemoglobin, Hb; Bioss Biotech, USA). To assess the functional features, after fixation and permeabilization, RBCs are incubated with antibodies conjugated to fluorochrome either directly or through secondary antibodies to s-nitrosocysteine (–SNO; Abcam), and pNOS3Ser1177 (Bioss). Next, RBC samples are run through a flow cytometer (FACS Calibur, BD Biosciences, San Diego, CA), and data is collected using CellQuest software to process for FC analysis.

Prestroke physical activity measures in AIS and ICH patients

(Substudy only at Aarhus University Hospital)

Patient and their relatives will be asked to complete the physical activity in the elderly (PASE) questionnaire during the acute hospital admission. In patients who are unable to complete it themselves an structured interview based on the PASE questionnaire will be performed[41,42]
The PASE is a 12-item questionnaire, which quantifies the amount of PA over a 7-day period. The PASE questionnaire was developed with the purpose of assessing the level of PA in middle-aged and elderly individuals. The PASE score is calculated by taking the average number of hours spent on an activity (sports, occupational activity, household activities, and leisure time activities) per day over a 7-day period multiplied by an activity coefficient. Item scores are added to reveal the PASE score. The PASE score may range from zero to more than 400.

9. Benefit of the study

Potential benefits:

Participating patients with AIS and ICH may experience an immediate reduction of their neurological symptoms and a persistent reduction of disability at long-term follow-up.

Disadvantage:

Mild-to-moderate pain and petechiae in the RIC-treated arm may occur during the inflation of the blood pressure cuff. Otherwise, the RIC treatment has been proven safe and without side effects.

Sham-RIC will only be associated with a slight sensation of pressure on the upper extremity.

Blood samples (only patients transported to and admitted at Aarhus University Hospital) of approximately ~~22~~ 57mL (19mL prehospital and 2x19 ~~11~~ mL in-hospital) will be drawn.

An additional (compared to standard operating procedure) head CT will be performed at 7 days in ICH patients included at Aarhus University Hospital (If possible, the scan will be performed at the hospital were the patient is admitted at day 7). The estimated added whole-body radiation dose of the 7 days CT-scan is

on average 1,5 mSv (range 1,05 to 1,95) corresponding to 6 months of accumulated background radiation in Denmark (Danish Health Authority). This corresponds to an estimated increased life-time cancer risk of 0.005%.

10. Assessment of safety

Emergency unblinding procedure

All on call neurologists will have access to electronic case report form using a personal and password protected login to perform randomization of study participants. In cases where emergency unblinding is necessary the on call neurologist will logon to electronic CRF at ddsc.dk and enter the civil registration number (CPR) of the patient. The treatment can now be unblinded. All changes will leave an audit-trail.

Adverse events

Patients are monitored in the stroke unit with NIHSS scoring and Scandinavian Stroke Scale (SSS) at close intervals, and adverse events are treated according to clinical guidelines.

The Data Monitoring Committee will assess safety according to primary study endpoints, recurrent stroke, myocardial infarction and mortality.

Interim analysis of safety parameters (hematoma expansion at 24 hours and mortality) in ICH will be performed regularly during the study.

During the acute in-hospital phase the patients will be asked if they have experienced any deterioration of health or new symptoms during or after treatment with the investigational device. Furthermore all patients will have a 24/7 contact number to the stroke center and a contact to study research physician and will be instructed to report any deterioration of health or new symptoms. All discharged patients (and relatives) will be given contact details to study personnel and be instructed to report any deterioration of health or new symptoms. The patients with acute ischemic stroke and intracerebral hemorrhage will once again be asked for adverse events at the 3 month telephone interview. In this group, end-of-study visit is the last telephone interview, whereas end-of-study visit for participants without a vascular diagnosis or TIA(without DWI lesion) is at the time of discharge from the stroke center.

All patients, who have consented, are followed (through Danish Health Registries, LPR and DSR) for new vascular events and mortality for up to 12 months.

Adverse events and adverse device events are defined according to ISO 14155:2011 and European Commission guideline on medical devices (MEDDEV 2.7/3 revision 3).

Adverse event (AE):

Any untoward medical occurrence, unintended disease or injury or any untoward clinical signs (including an abnormal laboratory finding) in subjects, users or other persons whether or not related to the investigational medical device.

Serious Adverse Event (SAE)

Adverse event that:

a) led to a death, injury or permanent impairment to a body structure or a body function.

b) led to a serious deterioration in health of the subject, that either resulted in: - a life-threatening illness or injury, or - a permanent impairment of a body structure or a body function, or - in-patient hospitalization or prolongation of existing hospitalization, or - in medical or surgical intervention to prevent life threatening illness

Device Deficiency

Inadequacy of an investigational medical device related to its identity, quality, durability, reliability, safety or performance. This could be malfunctions, use error, or inadequacy in the information supplied by the manufacturer.

Adverse Device Effect

Adverse event related to the use of an investigational medical device.

This includes any adverse event resulting from insufficiencies or inadequacies in the instructions for use, the deployment, the implantation, the installation, the operation, or any malfunction of the investigational medical device.

This includes any event that is a result of a use error or intentional abnormal use of the investigational medical device.

Serious Adverse Device Effect (SADE)

Adverse device effect that has resulted in any of the consequences characteristic of a serious adverse event.

Unanticipated Serious Adverse Device Effect (USADE)

Serious adverse device effect which by its nature, incidence, severity or outcome has not been identified in the current version of the risk analysis report.

Anticipated SADE (ASADE): an effect which by its nature, incidence, severity or outcome has been previously identified in the risk analysis report

Event report and causality assessment

The relationship between the use of the medical device and the occurrence of each adverse event will be assessed and categorized. All events will be registered in the electronic CRF and reported to the authorities at the interim analysis. A yearly safety report, containing a list of all SAEs/SADEs and near-miss incidents, thorough evaluation of individual events, risk/benefit analysis of the investigational medical device and safety conclusions, will be submitted to the Danish Health and Medicines Authority and the Independent Ethics Committee by the Sponsor as required by Danish law.

Exceptions to and special considerations about reporting procedures are stated below:

Acute stroke is an acute life threatening disease with a high risk of neurological deterioration and mortality. The natural history of stroke is associated with a high risk of complications (stroke in progression, hemorrhagic transformation, dysphagia, cardiac arrhythmia, pneumonia, and other serious infections). These *complications* all relates to the index stroke (occurring *before* randomization) and is foreseeable. If an event occurs that based on the assessment by the investigator is a result of the natural history of the

disease it will not be reported by the sponsor to the Danish medicines agency and regional ethics committee within 7 days/2 days (see list of foreseeable events/complications below).

All expected serious adverse events will be reported to the Danish Medicines Agency (and regional ethics committee) every 3 months.

All *new* serious events (Serious Adverse Events), complications that are not foreseeable or device near incidents or malpractice will be reported by the sponsor to the authorities (within 7 days, or 2 days if there is a risk of event reoccurrence) according to ISO 14155:2011 and European Commission guideline on medical devices (MEDDEV 2.7/3 revision 3). Investigators will report the event to sponsor (within 24 hours). Sponsor will as fast as possible and no later than 7 days, or 2 days if there is a risk of event reoccurrence, report the event to the Danish Medicines Agency and regional ethics committee.

All Serious Adverse Device Effect (SADE) will be reported to the Danish Medicines Agency (and regional ethics committee) within 7 days, or 2 days if there is a risk of event reoccurrence

List of foreseeable adverse events/complications

Foreseeable events/complications that will not be reported by the sponsor to the Danish medicines agency and regional ethics committee within 7 days/2days.

Patient Population	Event	Frequency	Mitigation/treatment
**TIA and AIS**	Recurrent stroke.	10% at 90 days [a]	Standard medical treatment, intravenous thrombolysis/mechanical thrombectomy according to patient characteristics
**AIS**	Symptomatic intracerebral hemorrhage (related to IV tPA or EVT)	7% [b]	Antithrombotic/anticoagulation therapy cessation. Antifibrinolytic agent. Bloodpressure control.
**AIS, ICH**	Early (<24hours) and late (>24hours) neurological deterioration<br>(ΔNIHSS ≥2 or death)	7-22% [c]	According to etiology
**AIS, ICH**	Aspiration pneumonia	5-20% [d]	Antibiotic treatment according to hospital SOP
**AIS, ICH**	Urinary tract infections	12% [e]	Antibiotic treatment according to local hospital SOP
**AIS, ICH**	Severe infections (sepsis)	13% [f]	Antibiotic treatment according to local hospital SOP
**AIS, ICH**	Cardiac arrhythmia	29% [g]	Depending on arrhythmia type. Treatment according to cardio.dk

**AIS, ICH**	Acute myocardial infarction	6% [h]	Treatment according to cardio.dk and conference call with cardiologist
**AIS, ICH**	Deep venous Thrombosis(DVT)/pulmonary embolism(PE)	DVT: 8% [i]<br>PE:1% [j]	Treatment according to cardio.dk
**AIS, ICH**	Falls (in-hospital phase)	14-65% [k, l]	Treatment according to type of injury
**AIS, ICH**	Seizure	AIS:3-10% [m]<br>ICH:8% [n]	Treatment according to local SOP
**AIS/ICH**	Mortality 30 days	AIS:10-20% [m]<br>ICH:30% [n]	

[a)] Wang Y et al. Clopidogrel with Aspirin in Acute Minor Stroke or Transient Ischemic Attack. NEJM. 2013; 369:11-19

[b)] Berkhemer OA. A Randomized Trial of Intraarterial Treatment for Acute Ischemic Stroke. NEJM. 2015; 372:11-20

[c)] Kwan J et al. Early neurological deterioration in acute stroke: clinical characteristics and impact on outcome Journal of the Association of Physicians, 2006, Sept; 99:9: 625-633

[d)] Hannawi Y et al. Stroke-Associated Pneumonia: Major Advances and Obstacles. Cerebrovasc Dis. 2013;35:430-443

[e)] Bogason E. Urinary Tract Infections in Hospitalized Ischemic Stroke Patients: Source and Impact on Outcome. 2017;9(2):e1014. doi:10.7759/cureus.1014.

[f)] BergerB. Epidemiologic features, risk factors, and outcome of sepsis in stroke patients treated on a neurologic intensive care unit. Journal of Critical care. 2013;29(2)

g)Daniele O et al. Stroke and cardiac arrhythmias. J Stroke Cerebrovasc Dis. 2002 jan-feb;11(1):28-33

[h)] Johnston KC et al. Medical and neurological complications of ischemic stroke: experience from the RANTTAS trial. RANTTAS Investigators. Stroke. 1998;29(2):447

[i)] Bembenek J. Early stroke-related deep venous thrombosis: risk factors and influence on outcome. J thromb Thrombolysis. 2011 Jul; 32(1):96-102

[j)] Pongmoragot J. et al. Pulmonary Embolism in Ischemic Stroke: Clinical Presentation, Risk Factors, and Outcome. J Am Heart Ass. 2013 Dec;2(6)

[k)] Davenport RJ, Dennis MS, Wellwood I, Warlow CP. Complications after acute stroke. Stroke. 1996; 27: 415–420.

[l] Teasell R, McRae M, Foley N, Bhardwaj A. The incidence and consequences of falls in stroke patients during inpatient rehabilitation: Factors associated with high risk. Arch Phys Med Rehabil. 2002; 83: 329–333.

[m)] Bladin C, Alexandrov AV, Bellavance A et al: Seizures After Stroke: A Prospective Multicenter Study.Arch Neurol. 2000;57(11):1617-1622.

[n)] Woo K-M, Yan S-Y, Cho K-T. Seizures after Spontaneous Intracerebral Hemorrhage. J. Korean Neurosurg Soc. 2012 Oct;52(4)312-319

[O)]Schwartz J et al. Incorporating Stroke Severity Into Hospital Measures of 30-Day Mortality After Ischemic Stroke Hospitalization. Stroke. 2017. Ahead of print: https://doi.org/10.1161/STROKEAHA.117.017960

[p)] Lichtman JH et al. 30-Day Mortality and Readmission after Hemorrhagic Stroke among Medicare

List of anticipated adverse device effects

**RIC device**	**Frequency**	**Mitigation/treatment**
**Local petechiae**	4-5% [a]	none
**Discomfort/pain**	20-30%	Treatment stopped if the non-competent patient verbally or non-verbally demonstrates intolerable discomfort or if the competent patient verbally want to discontinue the treatment

**Near events (device related)**	None known in the literature	
**Acute limb ischemia (upper extremity) – theoretical complication.**	None known in the literature	Treatment stopped. Treatment according local hospital SOP's for acute limb ischemia.<br>Known atherosclerotic stenosis in upper extremities are ***an exclusion criteria.***<br>See Investigators Brochure for further details.
**Sham RIC device**		
**None known**		

[a)] Hausenloy D et al. Remote Ischemic Preconditioning and Outcomes of Cardiac Surgery. New England J. Medicine. 2015 Oct;373(15):1408-17

Treatment and mitigation of any adverse events will be according regional guidelines.

Adverse events as a result of standard treatment will be handled according to local guidelines. Adverse events may also occur as a suspected reaction to RIC/Sham RIC – in these cases, the conditioning stimulus will be stopped immediately and appropriate treatment will be initiated.

In cases of SAE and device related near events all patients will be followed until resolution of symptoms/signs and/or all clinical relevant treatment have been performed and/or until the symptoms/signs are in a stable phase.

11. Project timetable and recruitment feasibility

Study preparation: January to February 2017

Study start date: March 2018

Month 0-60: Patient recruitment

Expected inclusion end date: 31. December 2022

Month 60-66: Study analysis

Expected end date for last analysis and follow-up: 31. December 2024

Patient inclusion per center:

Aarhus University Hospital: 50%

Regional Hospital West Jutland (Holstebro): 12,5%

Odense University Hospital: 12,5%

Aalborg University Hospital: 12,5%

Zealand University Hospital (Roskilde): 12,5%

12. Ethical considerations

The study will await approval from the Regional Ethical Committee, the Danish Health Authority, and the Danish Data Protection Agency. Enrollment of additional Danish stroke centers will require a new application to the Danish Medicines Agency and the regional ethics committee (amendment).

A large acute stroke results in immediate, major neurological deficits (+/-aphasia) and a substantially increased risk of a long-term reduction of functional capability. This group is assessed as legally incompetent. Even in patients suffering from minor stroke, acute cognitive impairment are common, making individual preference on acute treatment options impossible. In order to preserve brain tissue, the intervention must be applied without delay. Improvement of standard care in patients suffering from acute stroke is of paramount importance. Inclusion of all stroke patients is necessary in order to translate any positive research results into a benefit for all stroke patients. The intervention is without any known risk apart from moderate pain in the upper extremity when the cuff is inflated. We find that the conditions for an acute study are satisfied since the majority of the patients are incapacitated at stroke onset and the treatment has to be initiated as soon as possible and there are no reported serious side effects from RIC.

Study participants are covered in accordance with the Danish Patient Insurance Act.

13. Data handling and record keeping

All study data are recorded in an electronic CRF with blinded data and identification via a study identification number. The study will apply to the specifications of act on processing of personal data.

The database(e-CRF) is handled by OPUS consult Aps, Egaa, Denmark, who already handles the Danish Stroke Center Database in Aarhus (ddsc.dk)

Remote Ischemic Conditioning in Stroke (RISC) is a predefined collaboration in which three large RIC trials have agreed to share anonymized patient data for a large metaanalysis once the individual trials have ended (Principle Investigators of the individual trials are: Grethe Andersen (DK), Philip Bath (UK) and Fernando Pico (France).

Patient participation will be recorded in the medical record. Data will be stored at the Department of Neurology, Aarhus University Hospital, for 15 years, after which the documents will be shredded.

A notification to The Danish Data Protection agency will be submitted.

During the entire study period and at all participating centers the local GCP unit will perform quality assurance control including source data verification.

A complete list of all source data will be made and approved by the local GCP unit before study initiation.

The investigator permits direct access to all source data/documents (including electronic patient record) at monitoring visits, audits and/or inspections by the regional ethics committee and Danish Medicines Agency.

14. Publications policy

The results of the study, both negative, inconclusive and positive, will be disseminated as widely as possible - through publication in an international peer-reviewed journal, as conference presentations and on www.clinicaltrials.gov.

The trial is registered on ClinicalTrials.gov with Identifier: NCT03481777

15. Statistics

Primary study endpoint:

Difference in clinical outcome (mRS) at 3 months in acute ischemic stroke *(General ordinal logistic regression analysis. Significance level of 5%)*

The **main analysis of the primary study endpoint** will be performed using the entire range of the modified Rankin Scale **(General ordinal logistic regression, unadjusted)** on the population fulfilling the ***in-hospital inclusion criteria (target population)***. The trial success analysis is performed on the **target population** which consists of prehospital randomized patients with an in-hospital diagnosis of acute ischemic stroke (including TIA with a DWI lesion) and intracerebral hemorrhage (ICH) regardless of adherence to investigational treatment (**modified Intention-To-Treat analysis**).

Supplementary safety analysis:

All patients randomized in RESIST had pre-randomization a focal neurological deficit (documented on the prehospital stroke score). The presence or absence of specific focal neurological symptoms are assessed again after 24 hours using the same score as pre-randomization (PreSS) in all randomized patients (PreSS 24 hour/ at discharge). The primary safety analysis will be made on the **entire randomized population using the difference in prehospital stroke score** (PreSS) to document change in neurological deficits between pre-randomization and 24 hour/discharge **(General ordinal logistic regression, unadjusted)**. There has been no safety concern over RIC treatment in patients with non-vascular diagnosis[23,27,28,29,30]

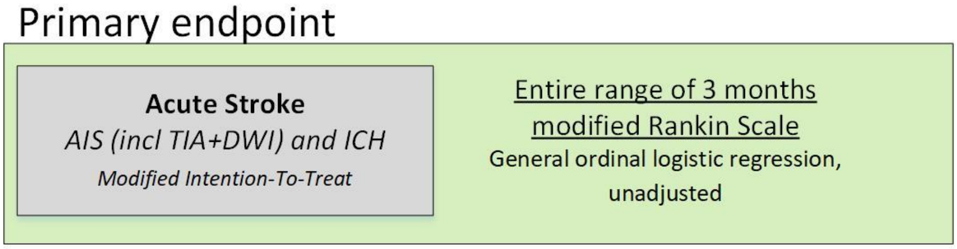

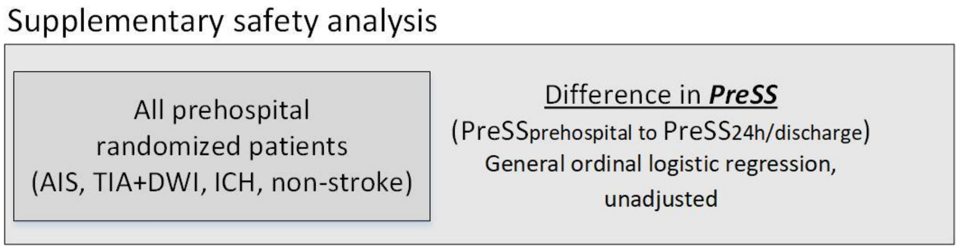

If remote ischemic preconditioning is associated with ultra-early treatment effect and reduced symptom severity (in the ambulances), this could affect the in-hospital indication for IV-tPA and/or EVT (in the case of rapid improvement of symptoms or mild symptoms)

Because of this, a **supplementary analyses** using **general ordinal logistic regression, adjusted for a possible** imbalances in reperfusion treatments (IV-tPA and EVT) between treatment arms will be made.

Secondary study endpoints (selected):

- Difference in prehospital stroke score (PreSS) (Supplementary safety endpoint, see above) (All randomized patients, general ordinal logistic regression, significans level 5%)
- Difference in prehospital stroke score (PreSS) during the first 24 hours: (general ordinal logistic regression analysis in the stroke subtypes, Significance level of 5%)
- Difference in early neurological improvement in AIS and ICH *(general ordinal logistic regression analysis. Significance level of 5%)*
- Difference in prehospital stroke score (PreSS) after 24 hours in subgroups: IV tPA/EVT treated AIS and ICH. (ordinal logistic regression analysis, significance level of 5%)
- Difference in acute hematoma expansion in patients with ICH (Binomial regression analysis, significance level of 5%)
- Difference in acute infarct growth in patients with AIS treated with IV-tPA and/or EVT (Binomial regression analysis, significance level of 5%)
- Major adverse cardiac and cerebral events (MACCE) and recurrent ischemic events at 3 and 12 months in AIS patients *(Cox regression analysis, significance level of 5%)*
- Three-month and one-year mortality *(Two sample test of proportion (Chi-square test), significance level of 5%)*

- EVT-eligibility (MRI assessed) in RIC treated AIS patients with large vessel occlusion (LVO) *(Binomial regression analysis. Significance level of 5%)*

Sample Size

The treatment effect of RIC on long-term functional outcome is unknown. We have assumed a small but clinical significant neuroprotective effect corresponding to a 6-7% increased odds for a beneficial shift on the modified Rankin Scale. The sample size calculation was based on a simulation-based approach to the analysis of statistical power when ordinal logistic regression analysis is performed (significans level of 5%)

The statistical power was simulated at different hypothetical sample sizes (on the target population) (ranging from 200 to 1900) with 2000 simulation-runs performed at each step. Unpublished data on IV-tPA and/or EVT treated AIS patients and ICH patients from our institution were used:

3 months modified Rankin Scale distribution (proportions) in 2017 for patients with ICH or IV tPA/EVT treated AIS at Aarhus University Hospital.

**Modfied Rankin Scale**	**mRS 0**	**mRS 1**	**mRS 2**	**mRS 3**	**mRS 4**	**mRS 5**	**mRS 6**
Proportion	0.139	0.273	0.141	0.110	0.145	0.07	0.126

Based on our previous trial experience with prehospital remote ischemic conditioning we estimate that a sample of 1000 subjects with target diagnosis (AIS and ICH) will be feasible to include during the study period.

Thus, including 1000 patients with target diagnosis provide sufficient power at a alfa-level (significans) of 5% to detect RIC treatment effects of the estimated 6-7 % *(see table below)*

**Treatment effect**<br>**(assumed neuroprective)**	5%	**6%**	**7%**
Sample (target diagnosis), *n*	1000	**1000**	**1000**
Power	66%	**80%**	**90%**
Alfa level (significans level)	5%	**5%**	**5%**

The estimated prehospital, randomized, sample:

**Sample size, prehospital**	**Proportion of randomized**	***n =***
**Target diagnosis (AIS and ICH), *n***	**67%**	**1000**
Non-vascular diagnosis	**27%**	**403**
TIA without DWI lesion	**4%**	**60**
Lost to follow-up	**2%**	**30**
Total		**1492**

**Plan to include**		**1500**

We therefor plan to include 1500 patients with a prehospital putative stroke in order to get 1000 patients with the target diagnosis of acute ischemic stroke and intracerebral hemorrhage. There is no planned replacement of patients lost to follow-up.

Interim analysis

We will perform an interim analysis for evaluation of the actual event rate after inclusion of 50% of the patients. If this shows a lower good outcome rate than expected, a new sample size calculation will be performed. Furthermore, the Data monitoring committee (DMC) will perform an independent safety analysis and will review the overall safety and efficacy according to the DMC charter (planned in the early phase of the trial, at 12 month and hereafter at least every 18. months) An additional safety interim analysis of mortality and hematoma expansion in ICH patients will be performed. Interim analysis are assessed using a level of significance of 1%.

The trial can be stopped at interim analyses for both futility, efficacy and safety reasons.

Detailed description is found in the DMC charter.

Missing data:

Missing data with be handled using multiple imputation (predictive mean matching imputation).

Deviations from the statistical plan:

All deviations from the statistical plan will first be applied when approved by the regional ethics committee and the Danish Medicines Agency.

Trial decision making and stopping rules

A Data Monitoring Committee (DMC) will be appointed before trial start and include a signed DMC charter. The DMC will review the accumulated data during RESIST trial and provide advice on the conduct of the trial to the Steering Committee.

The DMC should inform the Steering Committee
if, in their view:

1. the results are likely to provide convincing evidence that one trial arm is clearly indicated or contraindicated, and there is a reasonable expectation that this new evidence would materially influence patient management

or

2. it is beyond doubt that no clear outcome would be obtained (futility)

It is not expected that RESIST trial will be stopped for reasons of efficacy or futility based on observed treatment differences. A predefined pooling of individual patient data (Remote Ischemic conditioning in Stroke Collaboration, RISC) is planned. To dispel scepticism, a robust demonstration of efficacy of this simple intervention is required. The primary reason to recommend stopping the trial will be for safety reasons.

For Acute Ischemic Stroke and Intracerebral Hemorrhage:

- For the primary endpoint the DMC will consider stopping if there is a robust improvement in functional outcome after 3 months (modified Rankin Scale) for Acute Ischemic Stroke and Intracerebral haemorrhage in the sham (control) group compared to the intervention (RIC) group, achieving $p<0.01$.
- A substantial number of patients experience serious adverse events ($p<0.01$)
- DMC will consider stopping if there is an increase in mortality or stroke recurrence for Acute Ischemic Stroke and Intracerebral Hemorrhage patients in the intervention (RIC) group compared to the control (sham) group, achieving $p<0.01$.
- For ICH the DMC will consider stopping/modification if there is a clear evidence of hematoma growth during the first 24-hour in the intervention group, achieving $p<0.01$

The DMC will make recommendations, which could include:

- No action needed, trial continues as planned
- Early stopping due, for example, to clear benefit or harm of a treatment, futility, or external evidence
- Stopping recruitment within a subgroup
- Extending recruitment (based on actual control arm response rates being different to predicted other than on emerging differences) or extending follow-up
- Approving and/or proposing protocol changes

16. Source data access and monitoring

Trial-related audits and/or monitoring will be provided by direct access to source data/documents. Local monitors from the Unit for Good Clinical Practice, Aarhus University, will perform the audit and monitoring. Audit and monitoring will, likewise, be perfomed by qualified local GCP-monitors when enrollment at other stroke centers in Denmark is starting. Before enrollment can start a trial monitoring plan must be available. The audit and monitoring process will involve a 100% monitoring of signed consent forms ("samtykkeerklæringer og fuldmagtserklæringer") and serious adverse events.

17. Economy

Study initiator is Grethe Andersen, Senior Consultant, MD, DMSc, Professor of Neurology at Department of Neurology, Aarhus University Hospital.

The Danish foundation Vilhelm Petersens mindelegat has supported the study with 1.240.000 dkr (research nurse salary and "stroke RIC" devices).

The Danish foundation TrygFonden has supported the study with 4.000.000 dkr (research personnel salaries, running costs, micro RNA analysis and investigational devices).

Aarhus University has supported the study with 550.000 dkr (study investigator/coordinator salary)

The funders have no role in study design, data collection, analysis or interpretation, nor the decision to publish or the preparation, review and approval of the manuscript.

Stroke RIC/Sham-RIC device is developed in collaboration with Aarhus University, Faculty of Biomedical Engineering, 8200-DK, Aarhus N, Denmark; Seagull Aps, 4160-DK, Herlufmagle, Denmark and the Department of Neurology, Aarhus University Hospital, 8000-DK, Aarhus C, Denmark. Seagull Aps has no role in study design, data collection, analysis or interpretation, nor the decision to publish or the preparation, review and approval of the manuscript.

The research grants are transferred directly to a research account administered by the financial department of Aarhus University Hospital. None of the involved doctors or research nurses has any conflict of interest or economic advantages in regards to the study. There is no economic compensation or reimbursement for patients participating in the study.

Supplements

Supplement A - Neuroimaging protocol

On admission, either CT or MRI is performed according to hospital SOP.

All AIS and ICH patients can be included in the in-hospital RIC/sham-RIC based on a baseline native CT. However, patients with complete remission of symptoms after randomization (TIA) require an MRI at baseline demonstrating acute ischemic lesion on DWI in order to continue the randomized treatment. TIA patients with a documented ischemic lesion are, per definition, handled as ischemic stroke in this study. Baseline MRI in TIA patients is performed before the next RIC series (MRI <6 hours from RIC/sham-RIC last cuff). Patients with AIS who are not eligible for IV tPA due to contraindications will have CT/MRI before their next RIC/sham-RIC series (< 6 hours from RIPerC last cuff) according to hospital SOP. All ICH patients will have a baseline and 24-hour CT (hospital SOP)

Non-stroke diagnosis and TIA (only evaluated with CT or TIA without DWI lesion on MRI) will not be included in the in-hospital RIC study and will receive no further RIC treatment or neuroimaging.

**Imaging protocol**	**Accepted time frame**
MRI baseline – AIS IV tPA eligible	Acute on admission
CT baseline – AIS IV tPA eligible	Acute on admission
MRI/CT baseline – AIS non IV tPA eligible	< 6 hours from last RIC cuff
MRI (24-hour) - tPA/EVT treated AIS patients*	18-30 hour from baseline MRI
MRI baseline – ICH	Perform CT as soon as possible and < 3 hours from last RIC cuff
CT baseline – ICH	< 6 hours from last RIC cuff
Control CT (24-hour) – ICH*	18-30 hour from baseline CT
Control CT (7-day)*	5-9 days from baseline CT

*Substudy at Aarhus University Hospital

Computed tomography (CT) protocol
Baseline CT and CT angiography are obtained in centers using CT for primary assessment (according to hospital SOP).

CT analysis

Hematoma volume assessment at baseline and at 24 hours and 7 days is performed by an experienced neuroradiologist. The assessment will be assisted using an automated stroke volume assessment software (e.g. COMBAT stroke APS, Aarhus University Hospital, Denmark) or using the formula *ABC*/2 where *A* is the greatest hematoma diameter by CT, *B* is the diameter perpendicular to *A*, and *C* is the number of CT slices with hematoma multiplied by slice thickness[43].

Magnetic resonance imaging (MRI) protocol
Diffusion-weighted imaging (DWI), apparent diffusion coefficient (ADC), T2* gradient-recalled echo (T2*GRE), and T2 fluid-attenuated inverse recovery (T2-FLAIR). MR angiography time-of-flight (MRA-TOF) is performed according to hospital standard operating procedures (SOP).

The 24-hour MRI is performed on the same MRI scanner used at baseline, if possible. This protocol includes: DWI, T2*, T2 and T2-FLAIR. MRA-TOF at 24 hours is obtained if the baseline CT-angiography or MRA-TOF demonstrated vessel occlusion.

The total acquisition time for this protocol is approximately 7-10 mins (MRI)/10-15 min (MRI + MRA-TOF), depending on the MRI equipment.

MRI analysis
Acute (baseline) and 24-hour follow-up (DWI lesion will be outlined, representing irreversibly damaged tissue *(substudy at Aarhus University Hospital).*

Infarct growth is defined as the difference between baseline DWI and the 24-hour MRI-DWI lesion.

Infarct volume assessment at baseline and at 24 hours is performed by an experienced neuroradiologist. The assessment will be assisted using an automated stroke volume assessment software (e.g. COMBAT stroke APS, Aarhus University Hospital, Denmark) or using the formula *ABC*/2 where *A* is the greatest hematoma diameter by CT, *B* is the diameter perpendicular to *A*, and *C* is the number of CT slices with hematoma multiplied by slice thickness[43]

Vascular patency and reperfusion are assessed using the Thrombolysis in Cerebral Infarction Perfusion Scale (TICI) criteria[44]

- No perfusion (TICI 0)
- Perfusion past the initial occlusion, but no distal branch filling (TICI 1)
- Perfusion with incomplete or slow distal branch filling (TICI 2)
- Full perfusion with filling of all distal branches, including M3 and M4 (TICI 3)

Supplement B – Baseline data

Age	Platelet inhibitor treatment
Male/female	Anticoagulation therapy
**Medical History**	New oral anticoagulation treatment (NOAC)
Hypertension	Opioid treatment
Smoking	SSRI treatment
Alcohol	**Clinical and physiological data**
Hyperlipidemia	Prehospital Stroke Score (PreSS)
Diabetes	Modified Rankin Scale prestroke
Previous myocardial infarction<br>*Yes: within 3 months, yes: more than 3 months, no, unknown*	NIHSS (baseline)
Angina pectoris	Symptom onset (time)
Recent angina pectoris (< 4 weeks)<br>*If yes, date for latest episode*	Time of admission
Atrial fibrillation	Completed prehospital RIC/sham cycles
Previous ischemic stroke	= 5, 4, 3, 1-2, 0
Stroke < 3 month<br>*If yes, date*	Time (start/stop) for each RIC/sham cycle
Previous TIA	NIHSS (t = 24 hours)
Recent TIA < 4 weeks<br>*If yes, date for latest episode*	Stroke etiology (TOAST)
Previous ICH	Admission blood pressure
Peripheral artery disease	**IV tPA/EVT treated AIS**
Physical activity (PASE interview)<br>Substudy at Aarhus University Hospital	Treatment initiation (time)
**Medication on admission**	**Quality indicators (Danish Stroke Registry)**
Statins	**WHO-5**<br>Substudy at Aarhus University
Calcium channel blockers	
ACE inhibitors/angiotensin receptor blockers	
Beta-blockers	

Baseline data obtained from treating physician, database of Danish stroke center (ddsc.dk), Danish stroke registry and electronic health records.

References

1. Astrup J, Siesjo BK, Symon L. Thresholds in cerebral ischemia - the ischemic penumbra. *Stroke*. 1981;12(6):723-725.
2. Group N study. Tissue plasminogen activator for acute ischemic stroke. *N Engl J Med*. 1995;333(24).
3. Hacke W, Kaste M, Bluhmki E. Thrombolysis with alteplase 3 to 4.5 hours after acute ischemic stroke. *… Engl J …*. 2008:1317-1330. http://www.nejm.org/doi/full/10.1056/NEJMoa0804656. Accessed October 21, 2014.
4. Berkhemer OA, Fransen PSS, Beumer D, et al. A randomized trial of intraarterial treatment for acute ischemic stroke. *N Engl J Med*. 2015;372(1):11-20. doi:10.1056/NEJMoa1411587
5. Touma L, Filion KB, Sterling LH, Atallah R, Windle SB, Eisenberg MJ. Stent Retrievers for the Treatment of Acute Ischemic Stroke: A Systematic Review and Meta-analysis of Randomized Clinical Trials. *JAMA Neurol*. January 2016. doi:10.1001/jamaneurol.2015.4441
6. Prior A, Laursen TM, Larsen KK, et al. Post-Stroke Mortality , Stroke Severity , and Preadmission Antipsychotic Medicine Use – A Population-Based Cohort Study. 2014;9(1). doi:10.1371/journal.pone.0084103
7. Ren C, Gao M, Dornbos D, et al. Remote ischemic post-conditioning reduced brain damage in experimental ischemia/reperfusion injury. *Neurol Res*. 2011;33(5):514-519. doi:10.1179/016164111X13007856084241
8. Hougaard KD, Hjort N, Zeidler D, et al. Remote ischemic perconditioning in thrombolysed stroke patients: randomized study of activating endogenous neuroprotection - design and MRI measurements. *Int J Stroke*. 2013;8(2):141-146. doi:10.1111/j.1747-4949.2012.00786.x
9. Khatri R, McKinney AM, Swenson B, Janardhan V. Blood-brain barrier, reperfusion injury, and hemorrhagic transformation in acute ischemic stroke. *Neurology*. 2012;79(13 Suppl 1):S52-7. doi:10.1212/WNL.0b013e3182697e70
10. Campbell BC V, Christensen S, Tress BM, et al. Failure of collateral blood flow is associated with infarct growth in ischemic stroke. *J Cereb Blood Flow Metab*. 2013;33(8):1168-1172. doi:10.1038/jcbfm.2013.77
11. Ip HL, Liebeskind DS. The future of ischemic stroke: flow from prehospital neuroprotection to definitive reperfusion. *Interv Neurol*. 2014;2(3):105-117. doi:10.1159/000357164
12. Giles MF, Albers GW, Kim AS, Sciolla R. Early stroke risk and ABCD2 score performance in tissue- vs time-defined TIA A multicenter study. *Neurology*. 2011.
13. Kellner CP, Connolly ES. Neuroprotective Strategies for Intracerebral Hemorrhage Trials and Translation. 2010. doi:10.1161/STROKEAHA.110.597476
14. Anderson CS, Heeley E, Huang Y, et al. Rapid blood-pressure lowering in patients with acute intracerebral hemorrhage. *N Engl J Med*. 2013;368(25):2355-2365. doi:10.1056/NEJMoa1214609
15. Murry CE, Jennings RB, Reimer KA. Preconditioning with ischemia: a delay of lethal cell injury in ischemic myocardium. *Circulation*. 1986;74(5):1124-1136.
16. Hahn CD, Manlhiot C, Schmidt MR, Nielsen TT, Redington AN. Remote ischemic per-conditioning: a novel therapy for acute stroke? *Stroke*. 2011;42(10):2960-2962. doi:10.1161/STROKEAHA.111.622340

17. Hess DC, Hoda MN, Bhatia K. Remote limb perconditioning [corrected] and postconditioning: will it translate into a promising treatment for acute stroke? *Stroke*. 2013;44(4):1191-1197. doi:10.1161/STROKEAHA.112.678482

18. Schmidt MR, Pryds K, Bøtker HE. Novel adjunctive treatments of myocardial infarction. *World J Cardiol*. 2014;6(6):434-443. doi:10.4330/wjc.v6.i6.434

19. Li J, Rohailla S, Gelber N, et al. MicroRNA-144 is a circulating effector of remote ischemic preconditioning. *Basic Res Cardiol*. 2014;109(5):423. doi:10.1007/s00395-014-0423-z

20. Schmidt MR, Redington A, Bøtker HE. Remote conditioning the heart overview - translatability and mechanism. *Br J Pharmacol*. September 2014:1-37. doi:10.1111/bph.12933

21. Hoda MN, Bhatia K, Hafez SS, et al. Remote ischemic perconditioning is effective after embolic stroke in ovariectomized female mice. *Transl Stroke Res*. 2014;5(4):484-490. doi:10.1007/s12975-013-0318-6

22. Lim SY, Hausenloy DJ. Remote ischemic conditioning: from bench to bedside. *Front Physiol*. 2012;3(February):27. doi:10.3389/fphys.2012.00027

23. Hougaard KD, Hjort N, Zeidler D, et al. Remote ischemic perconditioning as an adjunct therapy to thrombolysis in patients with acute ischemic stroke: a randomized trial. *Stroke*. 2014;45(1):159-167. doi:10.1161/STROKEAHA.113.001346

24. Meng R, Asmaro K, Meng L, et al. Upper limb ischemic preconditioning prevents recurrent stroke in intracranial arterial stenosis. *Neurology*. 2012;79(18):1853-1861. doi:10.1212/WNL.0b013e318271f76a

25. Meng R, Ding Y, Asmaro K, et al. Ischemic Conditioning Is Safe and Effective for Octo- and Nonagenarians in Stroke Prevention and Treatment. *Neurotherapeutics*. 2015;12(3):667-677. doi:10.1007/s13311-015-0358-6

26. Vaibhav K, Braun M, Khan MB, et al. Remote Ischemic post-conditioning promotes hematoma resolution via AMPK-dependent immune regulation. Journal of experimental medicine. 2018. 6 sept (online). doi.10.1084/jem.20171905

27. Le Page S, Bejan-Angoulvant T, Angoulvant D, Prunier F. Remote ischemic conditioning and cardioprotection: a systematic review and meta-analysis of randomized clinical trials. *Basic Res Cardiol*. 2015;110(2):11. doi:10.1007/s00395-015-0467-8

28. Bøtker HE, Kharbanda R, Schmidt MR, et al. Remote ischaemic conditioning before hospital admission, as a complement to angioplasty, and effect on myocardial salvage in patients with acute myocardial infarction: a randomised trial. *Lancet*. 2010;375(9716):727-734. doi:10.1016/S0140-6736(09)62001-8

29. Hausenloy DJ, Candilio L, Evans R, et al. Remote Ischemic Preconditioning and Outcomes of Cardiac Surgery. *N Engl J Med*. 2015;373(15):1408-1417. doi:10.1056/NEJMoa1413534

30. Laiwalla AN, Ooi YC, Liou R, Gonzalez NR. Matched Cohort Analysis of the Effects of Limb Remote Ischemic Conditioning in Patients with Aneurysmal Subarachnoid Hemorrhage. *Transl Stroke Res*. 2016;7(1):42-48. doi:10.1007/s12975-015-0437-3

31. Hess DC, Blauenfeldt RA, Andersen G, et al. Remote ischaemic conditioning-a new paradigm of self-protection in the brain. *Nat Rev Neurol*. 2015;11(12):698-710. doi:10.1038/nrneurol.2015.223

32. Hastrup S, Damgaard D, Johnsen SP, Andersen G. Prehospital Acute Stroke Severity Scale to Predict Large Artery Occlusion: Design and Comparison With Other Scales. *Stroke*. 2016;47(7):1772-1776. doi:10.1161/STROKEAHA.115.012482

33. Kothari RU, Pancioli A, Liu T, Brott T, Broderick J. Cincinnati Prehospital Stroke Scale: reproducibility and validity. *Ann Emerg Med*. 1999;33(4):373-378.

34. Sivakumar L, Kate M, Jeerakathil T, Camicioli R, Buck B, Butcher K. Serial montreal cognitive assessments demonstrate reversible cognitive impairment in patients with acute transient ischemic attack and minor stroke. *Stroke*. 2014;45(6):1709-1715. doi:10.1161/STROKEAHA.114.004726

35. Riepe MW, Riss S, Bittner D, Huber R. Screening for cognitive impairment in patients with acute stroke. *Dement Geriatr Cogn Disord*. 2004;17(1-2):49-53. doi:10.1159/000074082

36. Bruno A, Akinwuntan AE, Lin C, et al. Simplified modified rankin scale questionnaire: reproducibility over the telephone and validation with quality of life. *Stroke*. 2011;42(8):2276-2279. doi:10.1161/STROKEAHA.111.613273

37. Guillemin F, Bombardier C, Beaton D. Cross-cultural adaptation of health-related quality of life measures: literature review and proposed guidelines. *J Clin Epidemiol*. 1993;46(12):1417-1432.

38. Beaton DE, Bombardier C, Guillemin F, Ferraz MB. Guidelines for the process of cross-cultural adaptation of self-report measures. *Spine (Phila Pa 1976)*. 2000;25(24):3186-3191.

39. Demchuk AM, Dowlatshahi D, Rodriguez-Luna D, et al. Prediction of haematoma growth and outcome in patients with intracerebral haemorrhage using the CT-angiography spot sign (PREDICT): a prospective observational study. *Lancet Neurol*. 2012;11(4):307-314. doi:10.1016/S1474-4422(12)70038-8

40. Kappetein AP, Head SJ, Genereux P, et al. Updated standardized endpoint definitions for transcatheter aortic valve implantation: the Valve Academic Research Consortium-2 consensus document. *Eur Heart J*. 2012;33(19):2403-2418. doi:10.1093/eurheartj/ehs255

41. Schuit AJ, Schouten EG, Westerterp KR, Saris WH. Validity of the Physical Activity Scale for the Elderly (PASE): according to energy expenditure assessed by the doubly labeled water method. *J Clin Epidemiol*. 1997;50(5):541-546.

42. Washburn RA, McAuley E, Katula J, Mihalko SL, Boileau RA. The physical activity scale for the elderly (PASE): evidence for validity. *J Clin Epidemiol*. 1999;52(7):643-651.

43. Kothari RU, Brott T, Broderick JP, et al. The ABCs of measuring intracerebral hemorrhage volumes. *Stroke*. 1996;27(8):1304-1305.

44. Khatri P, Neff J, Broderick JP, Khoury JC, Carrozzella J, Tomsick T. Revascularization end points in stroke interventional trials: recanalization versus reperfusion in IMS-I. *Stroke*. 2005;36(11):2400-2403. doi:10.1161/01.STR.0000185698.45720.58

5. Protocol amendment

(Clinical Investigation Plan version 8.1, August 29, 2019)

Clinical Investigation Plan

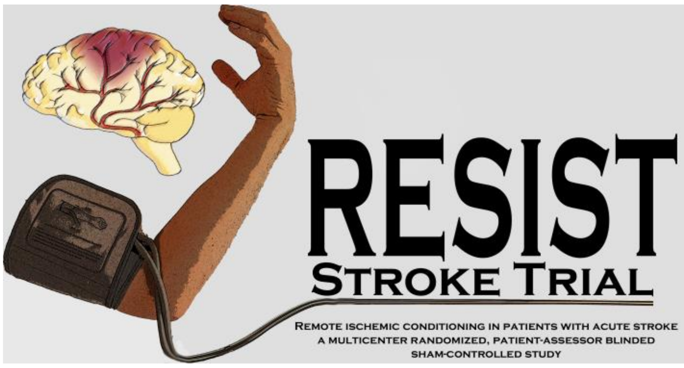

Remote ischemic conditioning in patients with acute stroke: a multicenter randomized, patient-assessor blinded, sham-controlled study.

A multicenter, prospective, randomized, patient-assessor blinded, sham controlled study investigating whether remote ischemic conditioning (RIC) can improve recovery.

<u>**Project acronym**</u>	<u>**RESIST stroke trial**</u><br>**RE**mote i**S**chemic conditioning **I**n patients with acute **ST**roke: a multicenter randomized, patient-assessor blinded, sham controlled study examining the effect on recovery
<u>**Clinical investigation plan**</u>	<u>**Version 8.1, 29th of August 2019**</u>
**Sponsor/Principal investigator (Aarhus)**	Grethe Andersen, Senior Consultant, MD, DMSc, Professor of Neurology. Department of Neurology, Aarhus University Hospital, DK8200, Denmark. Phone:+4523886653, e-mail: greander@rm.dk
**Study Coordinator & investigator**	Rolf Blauenfeldt, MD, PhD Student. Department of Neurology, Aarhus University Hospital, DK8200, Denmark. Phone: +4520774053, e-mail: rolfblau@rm.dk
**Principle Investigator (Holstebro)**	Birgitte Forsom Sandal, Senior Consultant, MD. Department of Neurology, Regional Hospital West Jutland (Holstebro), DK-7500, Denmark. Phone: +45 78437280, e-mail: Birgitte.Forsom.Sandal@vest.rm.dk
**Principle Investigator (Odense)**	Anne-Mette Homburg, Senior Consultant, MD. Department of Neurology. Odense University Hospital, DK-5000, Denmark. Phone: +45 65414691, e-mail: anne-mette.homburg@rsyd.dk
**Principle Investigator (Aalborg)**	Boris Modrau, Senior Consultant, PhD-student, MD. Department of Neurology. Aalborg University Hospital, DK-9000, Aalborg. Phone: +45 97662239, e-mail: boris.modrau@rn.dk
**Principle Investigator (Roskilde)**	Troels Wienecke, Senior Consultant, Associate professor, PhD, MD. Department of Neurology, Zealand University Hospital, DK-4000 Roskilde, Phone: +45 47322800, e-mail: trw@regionsjaelland.dk

RESIST trial is conducted according to the clinical investigation plan and current Danish legislation

The Clinical Investigation Plan will be approved and signed by all investigators before study start.

Contents

1. Synopsis 3
2. Abbreviations 7
3. Introduction 9
4. Hypothesis 10
5. Objectives 10
6. Trial design 11
7. Selection and withdrawal of subjects 13
8. Study procedure and assessment 16
9. Endpoints 20
10. Benefit of the study 30
11. Assessment of safety 31
12. Project timetable and recruitment feasibility 35
13. Ethical considerations 36
14. Data handling and record keeping 36
15. Publications policy 37
16. Statistics 37
17. Source data access and monitoring 41
18. Economy 42
References 46

1. Synopsis

**Name of the Sponsor/principal investigator**<br>Grethe Andersen, Senior Consultant, MD, DMSc, Professor of Neurology, Department of Neurology, Aarhus University Hospital, DK-8000, Denmark
**Name of investigational medical device: "Stroke RIC" & "Sham RIC"**<br>The investigational devices is developed in collaboration with the Faculty of Biomedical Engineering, Aarhus University, 8200-DK, Aarhus N, Denmark; Seagull Aps, 4160-DK, Herlufmagle, Denmark, and the Department of Neurology, Aarhus University Hospital, 8000-DK, Aarhus C, Denmark.
**Title of study: RESIST**<br>Remote ischemic conditioning in patients with acute stroke: a multicenter randomized, patient-assessor blinded, sham-controlled study, examining the effect on recovery.
**Trial Management Groups (TMG):**<br>**Principal investigator/Sponsor (Aarhus University Hospital):**<br>Grethe Andersen, Senior Consultant, MD, DMSc, Professor of Neurology, Department of Neurology, Aarhus University Hospital, Nørrebrogade 44, 8000 Aarhus C, Denmark<br><br>**Study Coordinator/Investigator**<br>Rolf Ankerlund Blauenfeldt, MD, PhD student. Department of Neurology, Aarhus University Hospital, Nørrebrogade 44, 8000 Aarhus C, Denmark<br><br>**Trial Steering Committee (TMC):**<br>Grethe Andersen (Aarhus University Hospital), Rolf Ankerlund Blauenfeldt (Aarhus University Hospital), Niels Hjort (Aarhus University Hospital), Hans Erik Bøtker (Aarhus University Hospital), David C. Hess (Medical College of Georgia, USA), Hans Kirkegaard (Aarhus University Hospital). Rikke Bay Thomsen (Aarhus University Hospital), Birgitte Forsom Sandal (Regional Hospital West Jutland, Holstebro) and Marc Fisher (Beth Israel Deaconess Medical Center, Harvard Medical School, USA).<br><br>**Data Monitoring Committee (and Trial Safety Committee):**<br>***The DMSC comprises of:***<br>Jesper Petersson (Skåne University Hospital, Sweden) – chair.<br>Jan Brink Valentin (Aalborg University Hospital, Denmark)<br>Thomas Christensen (Nordsjællands Hospital, Denmark)<br><br>**Trial Endpoints Validation Committee:**<br>EVC comprises of Independent senior consultants in Neurology and Cardiology<br>**Trial monitoring:**<br>During the entire study period and at all participating centres the regional GCP unit will perform quality assurance control including source data verification (GCP Unit Aarhus/Aalborg, ID 2017-718)
**Study centers:**<br>Department of Neurology, Aarhus University Hospital, DK-8000 Aarhus C, Denmark<br>Department of Neurology, Holstebro Hospital, DK-7500 Holstebro, Denmark

Department of Neurology, Odense University Hospital, DK-5000 Odense, Denmark<br>(Future study center, 2019 or 2020)<br>Department of Neurology. Aalborg University Hospital, DK-9000, Aalborg, Denmark<br>(Future study center, 2019 or 2020)<br>Department of Neurology, Zealand University Hospital, DK-4000 Roskilde, Denmark<br>(Future study center, in 2019 or 2020)<br><br>All Danish stroke centers will be invited to participate.
**Planned study period:** 2018-2022.
**Phase of development:**<br>Improve routine care of patients with acute stroke and transitory ischemic attack (TIA).
**Objectives:**<br>To determine whether combined remote ischemic per- and postconditioning can improve long-term recovery in acute stroke patients as an adjunct to standard treatment.
**Diagnosis:**<br>Acute ischemic stroke (AIS) and intracerebral hemorrhage (ICH).
**Methodology:**<br>A multicenter, investigator-driven, prospective, randomized, parallel assignment, patient-assessor blinded, sham-controlled clinical efficacy trial.
**Randomization:**<br>• Eligible patients will be randomized prior to the arrival at the hospital (via a secure web site).<br>• The patients and the clinical outcome assessor will be blinded to the treatment allocation.
**Number and subjects (planned):**<br>• 1,500 patients
**Inclusion criteria (prehospital):**<br>The initial treatment regime will be applied in the acute prehospital phase in setting of an acute research study<br>• Male and female patients (≥ 18 years)<br>• Prehospital putative stroke<br>• Onset of stroke symptoms < 4 hours before remote ischemic conditioning (RIC)<br>• Independent in daily living before symptom onset (mRS ≤ 2)<br>**Final in-hospital inclusion criteria**<br>• acute ischemic stroke including documented TIA<br>*or*<br>• ICH

TIA without documented ischemic lesion and non-vascular diagnosis will only have register-based long term follow-up only.

Exclusion criteria (pre-hospital)

Exclusion criteria, to be established during the teleconference between ambulance and on call neurologist

- Intracranial aneurisms, arteriovenous (AV) malformation, cerebral neoplasm, or abscess
- Pregnancy
- Severe peripheral arterial disease in the upper extremities
- AV shunt in the arm selected for RIC
- Concomitant acute life-threatening medical or surgical condition

Criteria for evaluation

Primary endpoints

- Clinical outcome (mRS) at 3 months in acute stroke (AIS and ICH)

Secondary endpoints

- Difference in prehospital stroke score (PreSS) during 24 hours in all randomized patients
- Clinical outcome (mRS) at 3 months in subgroups: AIS, IV tPA/EVT treated AIS and ICH
- Difference in prehospital stroke score (PreSS) during 24 hours in subgroups: IV tPA/EVT treated AIS and ICH.
- Difference in proportion of patients with complete remission of symptoms within 24 hours (TIA)
- Major Adverse Cardiac and Cerebral Events (MACCE) and recurrent ischemic events based on registry data at 3 and 12 months in ICH, AIS patients, TIA and non-vascular diagnosis
- Three-month and one-year mortality in AIS, ICH, and overall
- Early and very early neurological improvement in AIS and ICH patients
- Bed-day use at 3 and 12 months and quality of life measures at 3 months

Sub-studies at Aarhus University Hospital (secondary endpoints)

- Clinical outcome (mRS) at 3 months in patients with AIS and ICH and extended remote ischemic postconditioning protocol (*substudy at Aarhus University Hospital*)
- Proportion of RIC/Sham-RIC treated AIS patients with large vessel occlusion (LVO) eligible to EVT treatment (*substudy at Aarhus University Hospital*)
- Difference in acute (24-hour) hematoma expansion in patients with ICH
- Difference in hematoma reabsorption rate (7 days) in patients with ICH

- Infarct growth in AIS patients and hematoma growth in ICH patients
- Predictive abilities of Glial Fibrillary Acidic Protein (GFAP) in prehospital obtained blood samples combined with prehospital stroke severity to differentiate hemorrhagic from ischemic stroke and to grade ischemic stroke severity (*substudy at Aarhus University Hospital*)
- Diagnostic abilities of a prehospital microRNA and extracellular vesicles blood samples profile combined with prehospital stroke severity on the differentiation of hemorrhagic from ischemic stroke and to grade ischemic stroke severity (*substudy at Aarhus University Hospital*)
- microRNA and extracellular vesicle profile of RIC-induced neuroprotection at baseline (*substudy at Aarhus University Hospital*)
- Coagulation profile of putative stroke patients in prehospital obtained blood samples (*substudy at Aarhus University Hospital*)
- Modulation of coagulation by RIC (*substudy at Aarhus University Hospital*)
- Characterization of rheoerythrocrine dysfunction (RBC deformability, eryNOS3 and plasma nitrite) in RIC vs Sham-RIC treated stroke patients and its possible association to improved short term (24 hour) or long term (90 day mRS) clinical outcome or imaging biomarkers (DWI infarct growth) (*substudy at Aarhus University Hospital*)
- Prestroke physical activity level (PASE) as a predictor for early and long-term recovery. (*substudy at Aarhus University Hospital*)
- One week blood-pressure reduction in AIS and ICH patients (*substudy at Aarhus University Hospital)*

Study flow chart

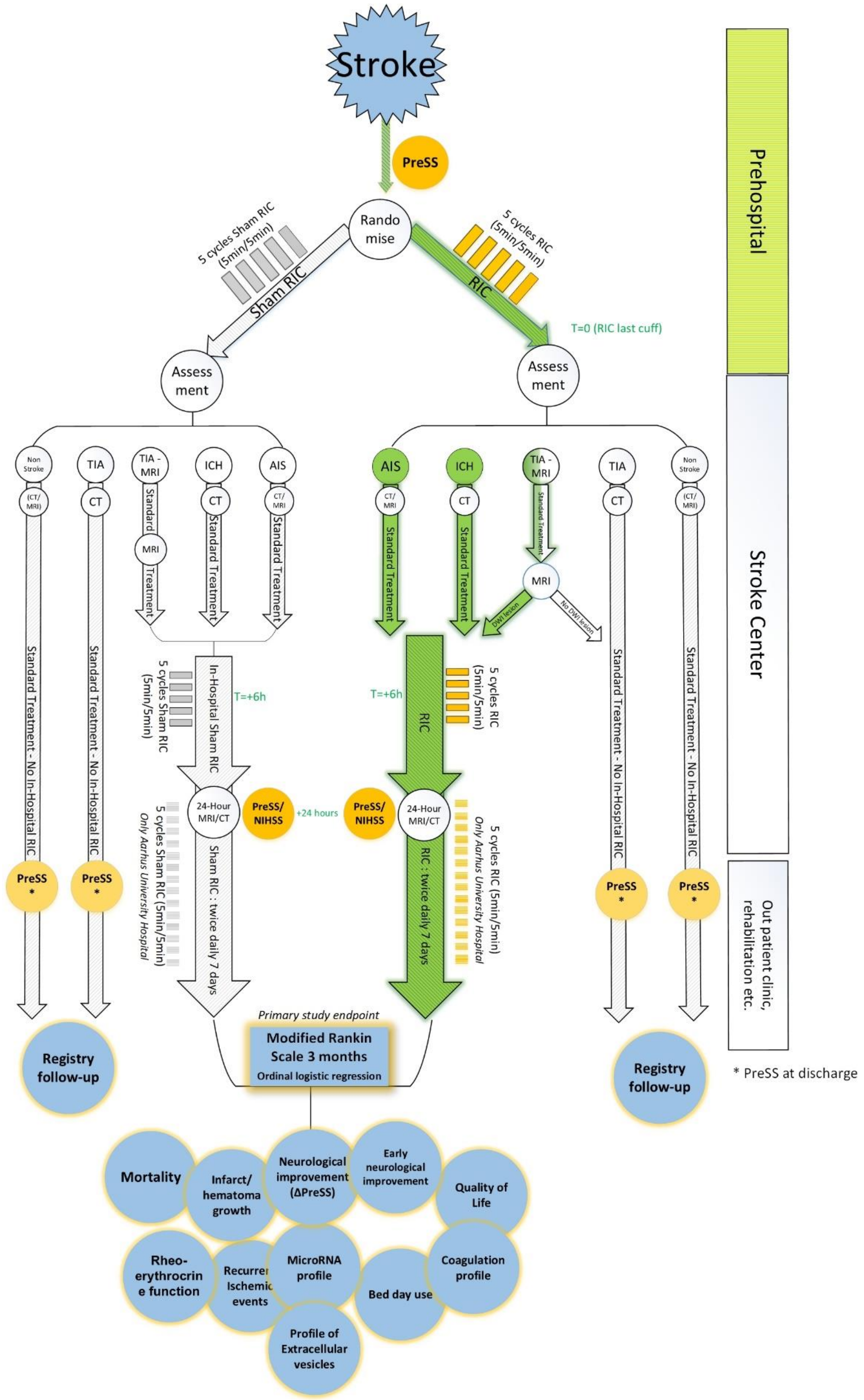

Abbreviations

AE	Adverse event
AIS	Acute ischemic stroke
AR	Adverse reaction
CT	Computed tomography
CPSS	Cincinnati Prehospital Stroke Scale
DWI	Diffusion-weighted imaging
DMC	Data Monitoring Committee and Endpoints Validation Committee
ICH	Intracerebral hemorrhage
END	Early neurological deterioration
EVT	Endovascular treatment
LVO	Large Vessel Occlusion
miRNA	Micro ribonucleic acid
mPTP	Mitochondrial permeability transition pore
MRI	Magnetic resonance imaging
NPR	Danish National Patient Register
PreSS	Prehospital Stroke Score
PWI	Perfusion-weighted imaging
RIC	Remote ischemic Conditioning
RIPerC	Remote Ischemic perconditioning
RIPreC	Remote ischemic preconditioning
RIPostC	Remote ischemic postconditioning
IV tPA	Intravenous thrombolysis/recombinant tissue-type plasminogen activator
SAE	Serious adverse event
SAR	Serious adverse reaction
TIA	Transient ischemic attack
TIA (DWI+)	Transient ischemic attack with a DWI-positive lesion on MRI
TMG	Trial Management Group
TSC	Trial Steering Group

2. Introduction

Our primary aim is to investigate whether remote ischemic conditioning (RIC) as an adjunctive treatment can improve long-term recovery in acute stroke patients as an adjunct to standard treatment.

Stroke is the second-leading cause of death worldwide and a leading cause of serious, long-term disability. The most common type is AIS which occurs in 85% of cases. Acute cerebral thromboembolism leads to an area of permanent damage (infarct core) in the most severely hypoperfused area and a surrounding area of impaired, yet salvageable tissue known as the "ischemic penumbra"[1]. Intravenous alteplase (IV tPA) and endovascular treatment (EVT) are approved acute reperfusion treatments of AIS to be started within the first 4½-6 hours and as soon as possible after symptom onset to prevent the evolution of the infarct core[2,3,4,5]. The prognosis has improved overall for ischemic stroke and the one-month mortality rate has declined to an approx. 10% [6]. However, reperfusion itself may paradoxically result in tissue damage (reperfusion injury) and may contribute to infarct growth[7,8,9]. Infarct progression can continue for days following a stroke, and failure of the collateral flow is a critical factor determining infarct growth[10]. The pre-eminent therapeutic aim is to restore blood flow, improve collateral perfusion, and prevent reperfusion injury[11]. In transient ischemic attack (TIA), the ischemia and stroke symptoms are only temporary, but these patients possess a high risk of re-stroke – especially patients with evidence of infarction on neuroimaging[12]. TIA patients with a documented ischemic lesion are, per definition, handled as ischemic stroke in this study.

On the other hand, in ICH the culprit is an eruption of blood into the brain parenchyma causing tissue destruction with a massive effect on adjacent brain tissues. Hematoma expansion as well as inflammatory pathways that are activated lead to further tissue damage, edema, and penumbral hypoperfusion[13]. The prognosis after ICH is poor with a one-month mortality of 40%. Acute blood pressure lowering is recommended to prevent further hematoma growth, but the mechanisms behind this effect are not understood[14].

Novel therapeutics and neuroprotective strategies that can be started ultra-early after symptom onset are urgently needed to reduce disability in both AIS and ICH.

Ischemic conditioning is one of the most potent activators of endogenous protection against ischemia-reperfusion injury[15]. RIC can be applied as repeated short-lasting ischemia in a distant tissue that results in protection against subsequent long-lasting ischemic injury in the target organ[16]. This protection can be applied prior to or during a prolonged ischemic event as remote ischemic preconditioning (RIPreC) and perconditioning (RIPerC), respectively, or immediate after reperfusion as remote ischemic postconditioning (RIPostC)[17]. RIC is commonly achieved by inflation of a blood pressure cuff to induce 5-minute cycles of limb ischemia alternating with 5 minutes of reperfusion. RIC activates several protective mechanisms, through humoral and neuronal pathways[18]. Circulating microRNA appears to be intimately involved in the RIC stimulus and might act as possible effector molecules[19,20].

Preclinical studies show that RIC induces a promising infarct reduction in an experimental stroke model[21,22]. It has been demonstrated that RIC protects against ischemia-reperfusion injury in the heart, lung, kidney, and the brain[22]. Results from a recent proof-of-concept study at our institution indicate that RIPerC applied during ambulance transportation as an adjunctive to in-hospital IV tPA increases brain tissue survival after one month[23]. Furthermore, RIPerC patients had less severe neurological symptoms at admission and tended to have decreased perfusion deficits[23]. Another study using RIPreC found a decreased stroke recurrence and shorter time to recovery in patients with intracranial arterial stenosis[24, 25]. Furthermore,

emerging preclinical data indicate a significant increased hematoma reabsorption rate and an improved functional outcome after RIC-treated ICH[26].

To-date, no serious adverse events have been documented in RIC. The procedure has been applied in numerous cardiovascular ischemic patients and in patients suffering from cerebral hemorrhage (ICH/SAH)[23,27,28,29,30].

RIC is a non-pharmacologic and non-invasive treatment without noticeable discomfort that has first-aid potential worldwide[31]. However, whether combined remote ischemic per- and postconditioning can improve long-term recovery in AIS and ICH has never been investigated in a randomized controlled trial.

3. Hypothesis

RIC as an adjunctive treatment improves long-term functional recovery in AIS and ICH patients.

4. Objectives

Primary objectives

- To determine whether RIC improves the clinical outcome (mRS) at 3 months in AIS and ICH

Secondary objectives

- To determine whether RIC reduces neurological impairment (PreSS) in all randomized patients
- To determine whether RIC improves the clinical outcome (mRS) at 3 months in subgroups of AIS, IV tPA/EVT-treated AIS patients and ICH patients
- To determine whether RIC reduces neurological impairment (PreSS) at 24 hours in subgroups of IV tPA/EVT treated AIS and ICH.
- To determine whether RIC increases the proportion of patients with complete remission of symptoms within 24 hours (TIA; both with and without DWI lesion)
- To determine whether RIC reduces major adverse cardiac and cerebral events (MACCE) and reduces recurrent ischemic events at 3 and 12 months
- To determine whether RIC reduces the 3-month and 1-year mortality in patients with AIS, ICH, and overall
- To determine whether RIC reduces bed-day use at 3 and 12 months and affects quality of life at 3 months
- To determine whether RIC increases the occurrence of early and very early neurological improvement in AIS and ICH patients

Secondary endpoints (only Aarhus University Hospital)

- To determine whether an extended RIC protocol (one week) improves clinical outcome (mRS) at 3 months in patients with AIS and ICH (*substudy at Aarhus University Hospital*)
- To determine whether RIC Increased the proportion of AIS patients with large vessel occlusion (LVO) eligible to EVT treatment (*substudy at Aarhus University Hospital*)

- To determine whether RIC reduces infarct growth in AIS patients and hematoma growth in ICH patients (s*ubstudy at Aarhus University Hospital*)
- To determine whether RIC increases the hematoma reabsorption rate (7 days) in patients with ICH (s*ubstudy at Aarhus University Hospital*)
- To determine whether prehospital obtained Glial Fibrillary Acidic Protein (GFAP) in blood samples combined with prehospital stroke severity can differentiate hemorrhagic from ischemic stroke and grade ischemic stroke severity (*substudy at Aarhus University Hospital*)
- To determine whether prehospital obtained microRNA and extracellular vesicles blood samples combined with prehospital stroke severity can differentiate hemorrhagic from ischemic stroke and grade ischemic stroke severity (*substudy at Aarhus University Hospital*)
- To determine whether a circulating microRNA and/or extracellular vesicle profile of RIC-induced neuroprotection can be established (s*ubstudy at Aarhus University Hospital*)
- To determine whether the prehospital determined coagulation profile from putative stroke patients differs between hemorrhagic, ischemic stroke and non-stroke (s*ubstudy at Aarhus University Hospital*)
- To determine whether RIC modulates coagulation in hemorrhagic and ischemic stroke, and explore links between coagulation and infarct growth in AIS, hematoma expansion in ICH, and MACCE and recurrent ischemic events at 3 and 12 months in both AIS and ICH (s*ubstudy at Aarhus University Hospital*)
- To characterize the rheoerythrocrine dysfunction (RBC deformability, eryNOS3 and plasma nitrite) in RIC vs Sham-RIC treated stroke patients and its possible association to improved short term (24 hour) or long term (90 day mRS) clinical outcome or imaging biomarkers (DWI infarct growth) (s*ubstudy at Aarhus University Hospital*)
- To determine whether prestroke physical activity level (PASE) is a predictor for early and long-term recovery (*substudy at Aarhus University Hospital*)
- To determine whether RIC reduces one week blood-pressure in AIS and ICH patients (*substudy at Aarhus University Hospital)*

5. Trial design

Trial design

This is a multicenter, prospective, randomized, patient-assessor blinded, sham-controlled trial investigating whether RIC improve long-term recovery in acute stroke.

Number of centers

Patients with putative stroke from participating stroke centers in Denmark will be recruited.

Number of subjects

We estimate that a sample size of 1500 prehospital putative stroke patients will be required to achieve 1000 eligible AIS and ICH patients (primary study endpoint).

Sample size determination

Primary clinical endpoint

The treatment effect of RIC on long-term functional outcome is unknown. We have assumed a small but clinical significant neuroprotective effect corresponding to a 7% increased odds for a beneficial shift on the modified Rankin Scale. The sample size calculation was based on a simulation-based approach to the analysis of statistical power when ordinal logistic regression analysis is performed (significans level of 5%)

The statistical power was simulated at different hypothetical sample sizes (on the target population) (ranging from 200 to 1900) with 2000 simulation-runs performed at each step. Unpublished data on IV-tPA and/or EVT treated AIS patients and ICH patients from our institution were used:

3 months modified Rankin Scale distribution (proportions) in 2017 for patients with ICH or IV tPA/EVT treated AIS at Aarhus University Hospital.

**Modfied Rankin Scale score**	**mRS 0**	**mRS 1**	**mRS 2**	**mRS 3**	**mRS 4**	**mRS 5**	**mRS 6**
Proportion	0.139	0.273	0.141	0.110	0.145	0.07	0.126

Based on our previous trial experience with prehospital remote ischemic conditioning we estimate that a sample of 1000 subjects with target diagnosis (AIS and ICH) will be feasible to include during the study period.

Including 1000 patients with target diagnosis provide sufficient power at a alfa-level (significans) of 5% to detect RIC treatment effects of the estimated 7 % *(see table below)*

**Treatment effect**<br>**(assumed neuroprective)**	5%	6%	**7%**
Sample (target diagnosis), *n*	1000	1000	**1000**
Power	66%	80%	**90%**
Alpha level (significans level)	5%	5%	**5%**

The estimated prehospital, randomized, sample:

**Sample size, prehospital**	**Proportion of randomized**	***n =***
**Target diagnosis (AIS and ICH), *n***	**67%**	**1000**
Non-vascular diagnosis	**27%**	**403**
TIA without DWI lesion	**4%**	**60**
Lost to follow-up	**2%**	**30**
Total		**1492**
**Plan to include**		**1500**

We therefor plan to include 1500 patients with a prehospital putative stroke in order to get 1000 patients with the target diagnosis of acute ischemic stroke and intracerebral hemorrhage. There is no planned replacement of patients lost to follow-up.

Randomization

Randomization procedure

The patient will be randomized to standard treatment with RIC or sham-RIC by the on call neurologist/vascular neurologist at the receiving stroke center. The ambulance will contact the on call neurologist by telephone and describe the patient (standard operating procedure (SOP) in Denmark). The randomization is based on a secure web site providing computer-generated blocked randomization lists stratified by the center. The online randomization is stratified by age, strokecenter and the Prehospital Stroke Score (PreSS). The PreSS score consists of the Cincinnati Prehospital Stroke Scale (CPSS) with an additional opportunity to report other neurological symptoms (e.g. ataxia, sensory disturbances and visual field loss), and PASS (Prehospital Acute Stroke Severity Scale)[32,33]. Prehospital personnel participating in RESIST are trained in identifying stroke symptoms included in PreSS. The on call neurologist will make an assessment based on all available information whether the patient is eligible to participate in RESIST. Randomization is performed in the prehospital setting. Each on call neurologist participating in the study will receive unique access and will have no influence on the randomization process. All neurologists on call will be educated and trained in performing evaluation of potential eligible study candidates and to perform the online randomization during the telephone call with the ambulance personnel. There is always one neurologist on call in participating centers (hospital SOP).

Treatment allocation

1:1 Allocation

6. Selection and withdrawal of subjects

Selection of patients

All patients with a putative stroke who meet the study criteria will be included.

- Depending on the prehospital randomization (RIC versus sham-RIC), patients with AIS will continue RIC or sham-RIC treatment for an extended period. This group includes TIA with a documented DWI lesion on magnetic resonance imaging (MRI).
- Patients with ICH will continue in-hospital RIC or sham-RIC (according to randomization).

Informed consent for prehospital enrollment

Background:

Acute temporary cognitive impairment after stroke is very common, even in patients with minor stroke or remission of symptoms (TIA) (54%) [34]. Widespread cognitive deficits have been identified in an acute stroke patient population[35]. This finding is in line with the clinical experience in acute stroke care. Whether the putative stroke patients have major cognitive impairment in the prehospital setting can only be tested under calm and quiet in-hospital circumstances. Delivering acute neuroprotective care, it is of utmost

importance that treatment is started as soon as possible, i.e. without delay. RIC is without any known serious adverse events.

According to the Danish research ethics committees, a patient who is a candidate for inclusion in an acute research study is considered incompetent if that person is not able to care for his or her own affairs due to physical or mental impairment. We believe that the vast majority of stroke patients fulfil these criteria, and it is not possible to select the few who are, indeed, competent in the acute phase without formal cognitive testing and thus missing the purpose of acute neuroprotection intervention. The intervention is associated only with mild to moderate discomfort and minimal risk for adverse events.

Acute Prehospital phase:

The initial RIC/sham-RIC treatment regime will be applied in the acute prehospital phase in setting of an acute research study approved by the regional research ethics committees.

Whether further treatment, according to randomization, will be offered depends on the in-hospital examination.

In-hospital inclusion:

After the initial RIC/sham-RIC treatment, in-hospital diagnostic evaluation, and acute treatment, ***the competent patient*** will be presented with a consent form with information about the study. Before this, the on-duty neurologist/research physician will have examined the patient both physically and cognitively and will have assessed whether the patient is competent or not. The competent patient will receive oral information by the on-duty neurologist/research physician based on the written patient information. The written patient information will be handed out (*deltager information*). We will ask for the patient's acceptance to be included in the study. The patient can withdraw consent at any time. In addition, the acute nature of the disease necessitates circumvention of the usual requirements concerning a 24-hour time period for consideration and discussion with a lay representative. This has been done in recent comparable acute studies. This approach is acceptable as the intervention is without any known risk and associated with a potential benefit for the patient. In-hospital inclusion and assessment of cognitive impairment will be undertaken during the admission at the stroke center. Every effort will be made to inform the patients and relatives in quiet and undisturbed settings, this will be at the hospital bedroom or the designated room for conversations. We will prioritize the presence of nearest relatives at time of study information and inclusion.

For ***the incompetent patients***, informed consent will be obtained from next of kin and an independent physician. The consenters will be provided information (based on the summary of the protocol for the independent physicians and study participant information for the relatives) on the trial to be able to make an informed decision about the patient's participation in this trial. The independent physician will be the on-duty physician at the local department of neurosurgery, neuroanesthesiology, intensive care, neurophysiology, (neuro)radiology or the on duty physician from another specialty with stroke patient experience. On-duty physicians at these departments will receive detailed study information.

A patient who during the acute phase was legal incompetent and included as such, but during the follow-up period is assessed competent will receive oral and written information and we will ask for his/her's informed consent.

Patients who fulfill both prehospital and in-hospital inclusion criteria will, according to randomization, receive RIC/sham-RIC treatment again after +6 hours (***all centres***).

Participation in the extended remote ischemic conditioning(RIC) or sham-RIC (*for a total of 7 days*) study will take place ***only at Aarhus University Hospital***. The device will follow the patient when he/she is discharged from the stroke center.

TIA patients (without DWI lesion) and non-stroke diagnosis patients will be asked for consent to do registry follow-up until one year after discharge. For these patients end-of-study visit is registered at stroke center discharge.

Inclusion criteria (prehospital)

- Male and female patients (≥ 18 years)
- Prehospital putative stroke
- Onset of stroke symptoms < 4 hours before RIC
- Independent in daily living before symptom onset (mRS ≤ 2)

Exclusion criteria (prehospital)

- Intracranial aneurisms, AV malformation, cerebral neoplasm or abscess
- Pregnancy*
- Severe peripheral arterial disease in the upper extremities
- Concomitant acute life-threatening medical or surgical condition
- AV shunt in the arm selected for RIC

**Women of child-bearing age should be asked about their use of safe birth control methods (contraceptive pill, intrauterine devices both hormonal and non-hormonal, hormonal implants, hormonal depot injection and transdermal hormonal patch). If pregnancy cannot be ruled out in the prehospital phase the patient can't be included. Women with a safe birth control method should be encouraged to use this method during the entire period of active RIC treatment.*

Final in-hospital inclusion criteria

- AIS (including DWI-positive TIA patients)

  or
- ICH

TIA and non-stroke diagnose will only have long-term register based follow-up.

Method of blinding

Outcome assessment is blinded to the treatment arm and obtained by a clinical or telephone assessment of the level of dependency and need for help in daily activities (**modified Rankin Scale**) **(Primary study endpoint**).
No information regarding randomization status will be recorded in the patient record.
Patients and the assessors of endpoints are blinded.

Discontinuation of study participation

A patient can withdraw from the study at any time. Patients can be withdrawn from the study at the principal investigator's discretion. In case the patient cannot be reached by telephone, every effort will be made to contact the patient or to document the outcome regarding new vascular events via registries/electronic health records. If the patient withdraws from the study, the date and the reason for the patient's withdrawal will be recorded. The patient is encouraged to provide information about his or her reason(s) for withdrawal and any experienced adverse effects (AE) during the study.

7. Study procedure and assessment

Prehospital stroke identification

Prehospital putative stroke patients are identified in collaboration with the ambulance personnel and the on-duty neurologist/vascular neurologist at the nearest stroke center (by telephone), which is standard operating procedure in Denmark. All Danish ambulance personnel are trained in identifying putative stroke in the prehospital setting. In the present study protocol, all patients have a prehospital structured assessment of neurological symptoms/findings (based on items contained in PreSS) before randomization – which will further increase stroke symptom awareness.

Baseline data

Baseline data and process indicators are collected from the Danish Stroke Registry (DSR). Additional information about medical history and medication and clinical and physiological data are collected at baseline. Data are recorded in an electronic case report form (CRF) at discharge from the stroke unit. For a complete list of registered data, see *Supplement B*

Acute treatment:

Patients are treated according to the national clinical guidelines on stroke treatment, intravenous thrombolysis and endovascular therapy. All patients are treated with state-of-the-art multidisciplinary care in the setting of a stroke unit monitored by the DSR.

Blood samples

Patients with putative stroke admitted to the Aarhus University Hospital will have blood samples withdrawn, in the ambulance, from peripheral venous catheter (placed as standard operating procedure, SOP). A total of 19mL (9mL + 10mL) will be drawn in the prehospital phase.

Blood samples will also be drawn upon admission to the Aarhus University Hospital Stroke Center as a part of SOP. Additional 19.5mL (3x3mL + 3x3,5mL) of blood will be drawn at this time.

Patients with AIS and ICH diagnosis will have blood-samples drawn, at Aarhus University Hospital Stroke Center, again 24 (16-32) hours after randomization. This is not standard operating procedure. 19.5mL (3x3mL + 3x3,5mL) will be drawn at this time.

Only patients received at the Stroke Center at Aarhus University Hospital will have blood samples drawn. (Prehospital and in-hospital blood samples)

Not all ambulances will be equipped with for blood sample withdrawal, thus an estimated 500 patients will have blood samples drawn in the prehospital phase and in hospital at Aarhus University Hospital.

Blood samples will be stored until completion of the trial in a research biobank.

Patients not admitted at Aarhus University Hospital will have no additional blood samples drawn (neither prehospital or in-hospital)

MicroRNA, extracellular vesicles, Glial Fibrillary Acidic Protein (GFAP), RBC deformability, NOS3, plasma nitrite and coagulation analysis will be performed on the obtained blood. A research bio bank will be established, and all blood samples obtained during the study are stored here. During the entire study and analysis period, the material will be stored at Aarhus University Hospital. The last patient will be included in December 2022. Analysis of blood samples, according to secondary endpoints, will be performed during the study and until 2 years after inclusion of the last patient (no later than 31. December 2024). Hereafter the remaining material will be transferred to a biobank for future unspecified research purposes. We will submit an application to the Danish Data Protection agency for this additional 20-year storage period of the blood samples (until 31 December 2044), after that, the samples will be destroyed. All analysis will be performed in Denmark. Furthermore, for patients participating in the biomarker sub-study at Aarhus University Hospital we will use biochemical lab results taken as part of routine care upon admission.

Investigational devices and Remote ischemic conditioning protocols

Automatic remote ischemic conditioning devices

Both the RIC and sham-RIC device are developed in collaboration with Aarhus University, Faculty of Biomedical Engineering, 8200-DK, Aarhus N, Denmark; Seagull Aps, 4200-DK, Slagelse, Denmark and the Department of Neurology, Aarhus University Hospital, 8000-DK, Aarhus C, Denmark.

The devices are manufactured by Shenzhen Raycome Health Technology Co., Ltd, 3F, 51 Building, No.5 Qiong Yu Road, Hi-Tech Industrial Park, Nanshan District, Shenzhen, China 518057.

*The **Investigator's Brochure (IB)** contains a detailed description of the investigational devices including risk analysis assessment.*

A brief user's manual is found in each device bag. No preexisting experience is necessary in order to operate the device. Written and oral information combined (Healthcare professional at participating hospitals) with RESIST trial e-learning material (prehospital personnel) will be offered to familiarize the clinical personnel in using the investigational devices. Patients and relatives will be instructed using written and oral information.

Remote Ischemic Conditioning device (RIC device)

The device is programmed to five cycles (50 minutes), each consisting of five minutes of cuff inflation followed by five minutes with a deflated cuff. The cuff pressure will be 200mmHg; but if initial systolic blood pressure is above 175 mmHg, the cuff is automatically inflated to 35 mmHg above the systolic blood pressure. This is done to account for a maximum cuff air leakage of 6mmHg per minute and maximum deviation in bloodpressure measurement of 10mmHg. The maximum cuff pressure is 285 mmHg.

Sham – remote ischemic conditioning device (Sham-RIC device)

The device is programmed to five cycles (50 minutes), each consisting of five minutes of cuff inflation followed by five minutes with a deflated cuff. The cuff pressure during inflation will be 20mmHg.

Timestamps, blood pressure before RIC/sham-RIC and total RIC/sham-RIC cycles are recorded and stored on the device (accessible by *universal serial bus,* USB). The RIC and sham-RIC is pre-programmed before being delivered to collaborating centers/ambulances. No data on conditioning cuff pressure will be displayed on device screen.

In cases of short transport time, the RIC/sham-RIC protocol continues during the initial assessment at the stroke center. RIC/sham-RIC stimulation is discontinued just prior to the MRI and continues afterwards if the protocol was incomplete (< 5 cycles). In centers using CT, the RIC/sham-RIC protocol is continued during the scan until a total of 5 cycles are reached. The device automatically stops treatment when the 5 cycles are completed. Except for RIC/sham-RIC, the prehospital observation and treatment are according to standard procedures in the ambulance.

Stroke RIC/Sham-RIC will be used, only if approved by the Danish Medicines Agency

The blood pressure and heartrate measurement, by RIC and Sham-RIC, will only be used for research purposes.

Initial remote ischemic conditioning

(all patients)

RIC/sham-RIC cuff will be placed on the non-paretic upper arm. The cuff is placed on the upper extremity on the same side of the suspected side of cerebral stroke, e.g. in right hemiparesis the cuff on the left arm, and in monosymptomatic aphasia the cuff is placed on the left arm.

Remote ischemic conditioning at +6 hours

(All patients with AIS, ICH – all centers)

At 6 hours after the first RIC/sham-RIC protocol (6 hours from the last cuff-off, respectively), another series of RIC/sham-RIC is performed; five cycles each consisting of 5 minutes of ischemia are followed by 5 minutes of reperfusion.

Remote ischemic postconditioning (twice daily for 7 days)

(AIS and ICH – only at Aarhus university Hospital)

The post-conditioning stimulus will be applied twice daily in the first week. This occurs at pre-specified times: 08.00 PM and AM. However, RIC/sham-RIC can be applied no sooner than 10 hours and not later than 21 hours after the initial perconditioning stimulus (last cuff).

Aarhus University Hospital: RIC/sham-RIC treatment will continue for a total of 7 days. If patients are discharged to their home or to another department, they will continue treatment according to written instructions, including application of the device for 7 days. After RIC/sham-RIC on day 7, the device is then returned to the Stroke Center.

**RIC/sham-RIC protocol**	**Accepted time frame**
**Prehospital RIC**	< 4 hours from symptom onset
**RIC at +6 h**	4 to 8h from last cuff of the initial RIC
**RIC twice daily for 7 days** – 08.00 PM and AM*	06.00 to 10.00 PM and 06.00 to 10.00 AM

*RIC/sham-RIC can be applied no sooner than 10 hours and no later than 21 hours after the initial perconditioning stimulus.

Device Accountability log

All devices are registered in a *device accountability log*. When the prehospital personnel arrives at the stroke center, with a patient included in RESIST trial, a new device (of the same color) will be handed out. Device ID, ambulance ID, contact details to the ambulance and date of device hand-out will be registered. It will be recorded on a paper-log in the stroke department. These data are transferred to an electronic log keeping track of device location and time to service/battery charge (1 year). *Further details see Investigators Brochure.*

Adherence to treatment:

At least 4 cycles (out of 5) will be required for each series of RIC treatment to be considered with accepted adherence. For the twice daily treatment for 7 days the number of cycles depends on the starting time of the acute RIC treatment and may vary from patient to patient. For this reason, 80% of planned cycles needs to be completed for the +7 days RIC treatment to be considered with accepted adherence.

**Acute RIC**		**+6 hours**		**+7days RIC** (Only Aarhus University Hospital)	
**Planned**	**Accepted**	**Planned**	**Accepted**	**Planned**	**Accepted**
5 cycles (5/5min)<br>100%	4 Cycles (5/5min)<br>80%	5 cycles (5/5min)<br>100%	4 Cycles (5/5min)<br>80%	100%	80%

Study procedures table

	**Acute prehospital and in-hospital phase**					**+ 6 hour**	**+ 24 hour**			**+ 7days**		**+3 months**	**12 months**
	**Prehosp. stroke score (PreSS)**[1]	**Prehosp Blood-samples**[1]	**RIC/Sham-RIC**	**In-hospital assessment**[2]	**CT/MRI baseline**	**+6 hour RIC/sham-RIC** [4]	**PreSS 24h** [2]	**CT/MRI 24h**[3]	**24-hour Blood-samples**	**Extended (7 days) RIC/sham-RIC** [5]	**CT- 7 days**[3]	**mRS 3-months**[7]	**Clinical events during follow-up**[6]
**TIA (DWI-) - all**	X	(X)*	X	X	X		X						X
**Non-stroke - all**	X	(X)*	X	X	X		X						X
**AIS incl. TIA (DWI+) – Other¥**	X		X	X	X	X	X	X				X	X
**ICH - Other¥**	X		X	X	X (CT)	X	X					X	X
**AIS incl. TIA (DWI+) – AUH***	X	(X)	X	X	X(MRI)	X	X	X (MRI)	(X)	X		X	X
**ICH – AUH***	X	(X)	X	X	X	X	X	X (CT)	(X)	X	X (CT)	X	X

**AUH = Aarhus University Hospital , ¥ Other= Other participating stroke centers*

[1] Prehospital stroke score (PreSS) and bloodsamples. *Prehospital blood samples (*19mL*): only patients transported to Aarhus University Hospital)*

[2] Acute assessment at the Stroke Center. NIHSS, blood samples (19,5mL blood) and ECG (standard). Assessment of neurological symptoms at 24-hour: NIHSS. Patients with non stroke diagnosis and TIA without a DWI-MRI lesion, who are discharged before 24 hours will be scored using PreSS at discharge. Patients with vascular diagnosis, who had blood samples taken in the ambulance and upon stroke center arrival will have additional samples withdrawn at 24 hour (additional 19,5 mL blood). *In-hospital RESIST blood samples only at Aarhus University Hospital*

[3] Baseline CT is preferable for ICH patients (hospital SOP). ICH patients, at Aarhus University Hospital, will receive a 24 hour control CT (hospital SOP). If possible, a 24-hour MRI is performed in all included AIS patients with an MRI baseline scan. Furthermore, a 24-hour control CT will be provided for IV tPA-treated AIS patients, unless MRI can be performed and was the primary investigation. One week control CT will be performed, if possible, on ICH patients included at Aarhus University Hospital. End of study visit for non-vascular diagnosis and TIA (without DWI lesion) at stroke center discharge.

[4] RIC/sham-RIC at +6 hours from last RIC cuff

[5] RIC/sham-RIC twice daily for 7 days *(Only at Aarhus University Hospital)*

[6] Mortality, MACCE, and recurrent ischemic events are recorded using the Danish National Patient Register (LPR) and DSR at 6 and 15 months after the inclusion of the last patient.

[7] Assessments at 3 months by telephone or face-to-face (mRS and WHO-5). End-of-study (telephone or face-to-face) for AIS and ICH patients. WHO-5 will only be assessed by one assessor.

8. Endpoints

An independent end-point committee will adjudicate clinical events.

Primary endpoints

Criteria for evaluation

Clinical outcome (mRS) at 3 months in acute stroke

Primary study endpoint: Clinical outcome (modified Rankin Scale) at 3 months in acute stroke patients (generalized ordinal logistic regression)

The level of dependency and need for help in daily activities will be determined by either face-to-face assessment or based on a structured telephone interviews performed by assessors who are blinded to the intervention at 3 months after the index stroke.

The assessment will be performed by two independent blinded assessors, which can be either face-to-face or telephone based assessment (not same day). The electronic CRF database will alert if disagreement occurs, and the patient will be contacted by a third assessor (face-to-face or telephone) who is blinded to the intervention who will assess the level of dependency.

- If disagreement occurs between **two telephone assessments** – a third, and final, telephone or face-to-face assessment will be made.
- If disagreement occurs between one face-to-face assessment and one telephone assessment – the face-to-face will be considered the final assessment
- If disagreement occurs between **two face-to-face assessments** – a third, and final, telephone or face-to-face assessment will be made.

Every possible effort will be made to assess the outcome in patients who are unable to participate in the interview (this could be due to post stroke neurological impairment (for example aphasia and/or dysarthria), or that the patient cannot be reached by telephone). The outcome will then be assessed by

contact to a named relative or the general practitioner. Several attempts to contact the patient by telephone will be made before contacting the named relative or general practitioner.
Death in the follow-up period will be obtained from Danish Civil Registration System (CPR).

The structured telephone interview will be based upon a validated Danish translation of the "slightly revised simplified, modified Rankin Scale questionnaire"[36]. The translation and its validation will be performed according to the AAOS guideline[36, 37]

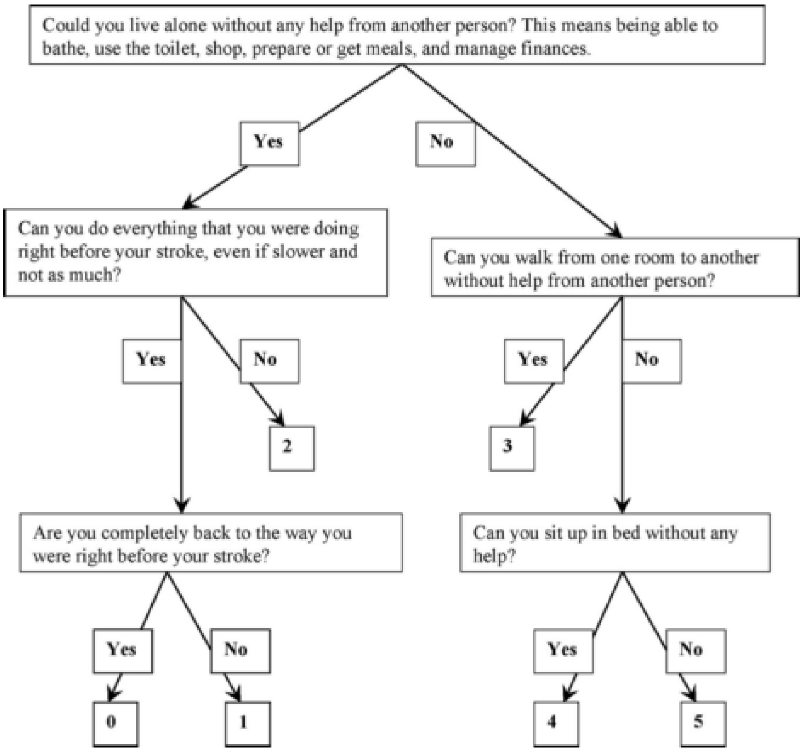

Bruno et al. 2011. Stroke.

Furthermore, modified Rankin Scale assessors are required to have received specific mRS training, which could be an online mRS certification https://secure.trainingcampus.net/uas/modules/trees/windex.aspx?rx=rankin-english.trainingcampus.net

For a **detailed statistical analysis plan**, see **statistics**

Outcome	Accepted time frame (from symptom onset)
3-month follow-up – 1st and 2nd assessment	3 months +/- 2 weeks
3 month follow-up – 3rd assessment	3 months and 2 weeks +/- 2 weeks

Secondary endpoints

Difference neurological impairment during the first 24 hours in all randomized patients

Neurological deficits are documented using PreSS (prehospital and in-hospital). The PreSS score is obtained by the prehospital personnel and registered by the on-call neurologist before randomization. Prehospital Stroke Score is assessed at 24-hour by a trained and blinded general neurology nurse/neurology research nurse/physician/neurologist who is blinded to the treatment intervention. Only information regarding time and place of assessment, patient name, age and social security number will be available. The PreSS score of patients with TIA (without DWI lesion) and non stroke diagnoses, are performed by the on-call physician/neurologist or a research nurse/general neurology nurse, and will be documented as *PreSS at discharge*, if discharge occurs before 24 hours. The outcome assessor will be alerted in advance of the assessment. The PreSS is recorded in the electronic CRF. Clinical assessors are required to complete an online PreSS certification or face-to-face training.

https://ekurser.rm.dk/test_projekter/anhesr/red-hjernen/scorm/Scorm2004%204th%20Edition/index.html

For a **detailed statistical analysis plan**, see **statistics**

**Indicator**	**Accepted timeframe (from symptom onset)**
PreSS	Close to symptom onset
$PreSS_{24h}$ (+ 24h)	+ 16 to 32h

Difference in proportion of patients with complete remission of symptoms within 24 hours (TIA; both with and without DWI)

Diagnosis of TIA is documented in the electronic case report form

**Endpoint assessment**
Difference in proportion of patients with complete remission of symptoms within 24 hours (TIA; both with and without DWI)

Major adverse cardiac and cerebral events (MACCE) and recurrent ischemic events at 3 and 12 months in AIS and all patients

MACCE is defined as:

Cardiovascular events (cardiovascular death, myocardial infarction, acute ischemic or hemorrhagic stroke)

Cardiovascular death: Death from known cardiovascular cause or sudden death from unknown cause (no identified cause of death in medical history and/or autopsy)

Acute myocardial infarction: Admission with a discharge diagnosis of ST-elevation myocardial infarction (STEMI) and non-ST elevation myocardial infarction (NSTEMI) and unstable angina pectoris (UAP)

Stroke: Admission with a discharge diagnosis of acute ischemic or hemorrhagic stroke.
Evaluation is performed using the Danish National Patient Register (LPR) and the DSR at two time points (6 and 15 months after the inclusion of the last patient).

Diagnosis of AIS/TIA, ICH and MI (STEMI, NSTEMI, and UAP) are made according to national clinical practice guidelines.

(http://neuro.dk/wordpress/nnbv/om-iskaemisk-apopleksi/ and http://nbv.cardio.dk/aks)

Recurrent ischemic vascular events at 3 and 12 months in AIS patients

Recurrent ischemic vascular events defined as:

- AIS
- TIA
- MI, STEMI, and NSTEMI

Three-month and one-year mortality

Information about mortality is collected using the CPR and LPR, at two time points (6 and 15 months after the inclusion of the last patient). All-cause mortality is assessed and subdivided into cardiovascular mortality versus non-cardiovascular mortality[40].

Analysis are performed on all randomized patients and according to subgroups.

Mortality (all-cause)

**Cardiovascular mortality**	**Non - cardiovascular mortality**
Acute MI	Pulmonary, renal, gastrointestinal, hepatobiliary and pancreatic
Sudden cardiac death	Infection and inflammatory/immune (including autoimmune)
Heart failure	Hemorrhage (non-cardiovascular (CV)/non-stroke-related bleeding)
Stroke: death as a direct consequence or complication of stroke	Non-CV procedure or surgery, trauma, suicide, drug reaction, or overdose
Cardiovascular procedure or procedure	Neurological, malignancy, other non-CV
Cardiovascular-related hemorrhage: non-stroke intracranial hemorrhage (subdural hematoma), non-traumatic vascular rupture (e.g. aortic aneurysm), or hemorrhage causing cardiac tamponade	

According to "2014ACC/AHA Key Data Elements and Definitions for Cardiovascular Endpoint Events in Clinical Trial". Circulation 2015;132:302-361.

EVT-eligibility (MRI assessed) in RIC treated AIS patients with large vessel occlusion (LVO)

EVT eligibility is assessed upon arrival at the Aarhus University Hospital in AIS patients with:

- Severe Stroke (NIHSS ≥ 10)
- Groin puncture feasible within 6 hours from stroke onset
- MRI-TOF (time-of-flight) documented internal carotid artery (ICA), Intracranial ICA (ICA-T) and first and second stem of the middle cerebral artery (M1 and M2, respectively)
- No contraindications to MRI (pacemaker, vomiting, respiratory insufficiency, obesity)
- MRI-DWI lesion volume ≤ 70mL

**Endpoint assessment**
Proportion of RIC treated AIS patients with LVO eligible to EVT treatment compared to standard treatment, adjusted for prehospital stroke severity (PreSS) and symptom duration
Difference in MRI-DWI lesion volume in RIC/sham-RIC treated LVO AIS patients eligible to EVT treatment

Quality of life measures at 3 months in AIS and ICH patients

- **Quality of life and bed-day use measures in AIS and ICH patients**

Quality-of-life measurements are assessed by telephone interview at 3 months after inclusion. The WHO-5 is used. Baseline WHO-5 is only obtained at Aarhus University Hospital.

Link to: Danish National Board of Health - WHO-5.
Information about bed-day use via the LPR and the DSR.

Early neurological improvement in AIS and ICH patients

Neurological deficits are documented with the PreSS and the National Institute of Health Stroke Scale (NIHSS), prehospital and in-hospital, respectively.

- **Very early neurological improvement** is defined as:

Reduction in prehospital stroke score (PreSS) ≥ 1 points or resolution of symptoms at admission: Items on PreSS related to NIHSS baseline $\Delta PreSS = PreSS_{prehospital} -$ same items on $NIHSS_{baseline}$

- **Early neurological improvement** is defined as:

Reduction in NIHSS ≥ 4 (baseline versus 24-Hour NIHSS): $\Delta NIHSS = NIHSS_{baseline} - NIHSS_{24}$

- **Reduction in prehospital stroke score (PreSS) ≥ 1 points or resolution of symptoms after 24 hours in subgroups:** IV tPA/EVT treated AIS and ICH.

Clinical assessors are required to complete an online PreSS certification

PreSS certification: https://ekurser.rm.dk/test_projekter/anhesr/red-hjernen/scorm/Scorm2004%204th%20Edition/index.html

**Indicator**	**Accepted timeframe (from symptom onset)**

PreSS	Close to symptom onset
NIHSS baseline	0 to 6 hours from admission
NIHSS (+ 24h) – standard operating procedure	+ 16 to 32h
PreSS (+ 24h)	+ 16 to 32h

Secondary endpoints (Only Aarhus University Hospital)

Difference in acute hematoma expansion in patients with ICH

(Substudy only at Aarhus University Hospital)

- Hematoma expansion is defined as the difference in volume between the baseline and the 24-hour CT hematoma volume. Significant hematoma growth is defined as an absolute growth exceeding 6 mL or a relative growth of more than 33% from the initial CT[39].

In all ICH patients, the baseline imaging modality is CT. In cases of initial MRI, a CT is performed afterwards (no later than < 3 hours from last RIC cuff)

Imaging protocol	Accepted time frame
CT baseline – ICH	< 3 hours from last RIC/sham-RIC cuff
MRI baseline – ICH	Perform CT as soon as possible and < 3 hours after the last RIC/sham-RIC cuff
Control CT (24-hour) – ICH	18-30 hours from baseline CT

Information about imaging protocols, see *Supplement A – Neuroimaging protocols*

Difference in 7 days hematoma reabsorption rate in patients with ICH

(Substudy only at Aarhus University Hospital)

- Hematoma reabsorption rate is defined as the difference in hematoma volume between the baseline and the 7-day CT hematoma volume.

In all ICH patients, the preferred baseline imaging modality is CT. In cases of initial MRI, a CT is performed afterwards (no later than < 3 hours from last RIC cuff)

Imaging protocol	Accepted time frame
CT baseline – ICH	< 3 hours from last RIC/sham-RIC cuff
MRI baseline – ICH	Perform CT < 3 hours after the last RIC/sham-RIC cuff if possible
Control CT (7-day) – ICH	5-9 days from baseline CT

Information about imaging protocols, see *Supplement A – Neuroimaging protocols*

Infarct growth in AIS patients

(Substudy only at Aarhus University Hospital)

Acute infarct growth is defined as the difference in infarct volume between baseline and 24 hours on DWI-MRI. All patients with IV-tPA and/or EVT treated ischemic stroke and a baseline MRI will have an additional 24-hour MRI performed.

**Imaging protocol**	**Accepted time frame**
MRI baseline – AIS IV tPA eligible	On admission
MRI baseline – AIS not IV tPA eligible	< 6 hours from last RIC/sham-RIC cuff
MRI (24-hour) – AIS IV-tPA/EVT treated	18-30 hours from baseline MRI

Information about imaging protocols, see *Supplement A*

Biochemical profile of stroke subtypes and of RIC-induced neuroprotection

(Substudy only at Aarhus University Hospital)

Prehospital biochemical substudies (Aarhus University Hospital)

- Diagnostic abilities of a prehospital microRNA and extracellular vesicles blood samples profile combined with prehospital stroke severity to differentiate hemorrhagic from ischemic stroke and to grade ischemic stroke severity
- Predictive abilities of Glial Fibrillary Acidic Protein (GFAP) and Occludin in prehospital obtained blood samples combined with a symptom based prehospital stroke score (PreSS – Prehospital Stroke Score). This to differentiate hemorrhagic from ischemic stroke from acute ischemic stroke and to grade ischemic stroke severity to identify patients with large vessel occlusions and to identify patients with a severity that requires admission to a comprehensive stroke center.
- Coagulation profile in prehospital obtained blood samples to differentiate between hemorrhagic stroke, ischemic stroke, and non-stroke
- Red blood cell rheo-erythrocrine dysfunction in acute stroke and its possible association to improved early (24 hour) or long term (90 day mRS) clinical outcome or imaging biomarkers (DWI infarct growth) (*substudy at Aarhus University Hospital*)

In-hospital biochemical substudies

- Characterization of a possible microRNA and extracellular vesicle profile in stroke subtypes
- MicroRNA and extracellular vesicle characterization of a possible RIC treatment profile
- Modulation of coagulation by RIC in hemorrhagic and ischemic stroke
- Characterization of rheoerythrocrine dysfunction (RBC deformability, eryNOS3 and plasma nitrite) in RIC vs Sham-RIC treated in acute stroke patients

Prehospital and in-hospital handling of blood samples:

Blood samples will be collected from patients with a prehospital putative stroke. Thus, blood samples will be drawn in the ambulance and upon arrival at the stroke center (only Aarhus University Hospital). In the ambulance, the blood sample will be obtained through a peripheral venous catheter (placed as standard operating procedure) into a 9mL and 10mL tubes and transported to hospital at ambient temperature (tubes of 9 and 10mL will be drawn). The Thrombosis & Hemostasis Research Unit, Aarhus University Hospital or study personnel will centrifuge the blood and divide the plasma into cryotubes that will be kept at 80 °C for long-term storage. Cryotubes not used to measure GFAP within 5 to 7 days will be stored in a research biobank.

Blood samples will be drawn upon admission to the Aarhus University Hospital Stroke Center as a part of local stroke center SOP. Additional 19,5mL (3 Tubes of 3,5mL and 3 tubes of 3mL) of blood will be drawn at this time.

Patients with AIS and ICH diagnosis will have blood-samples drawn, at Aarhus University Hospital Stroke Center, again 24 (16-32) hours after randomization (19,5mL (3 Tubes of 3,5mL and 3 tubes of 3mL)) will be drawn at this time). EDTA and citrate tubes will be used.

All blood samples obtained during the study will be stored in a research biobank

**Blood samples**	**Accepted time frame**
**Acute Prehospital**	As soon as possible
**Acute in-hospital**	As soon as possible
**+24hour in-hospital samples (only patients with a target diagnosis)**	< 16-32 hours from randomization and 2 hours (1-3 hours after completion of next day RIC treatment

Blood-samples, in the research biobank, will be stored until completion of the trial (no later than 31. December 2024). Hereafter the remaining material will be transferred to a biobank for future unspecified research purposes (see section “Blood Samples” under “study procedure and assessment”)

Use of routine lab results:

The following biochemical entities will be used (lab results performed as a part of standard operating procedure): C-reactive protein, Glucose, HBA1c, Potassium, Natrium, Calcium, Albumin, Creatinine, eGFR (estimated Glomerular Filtration Rate), Total WBC (White Blood Cell count/Leukocytes), Hemoglobin, Erythrocytes, Erythrocytes (Volume Fraction, EVF), Erythrocytes distribution widths (RDW), Erythrocyte median cell volume (MCV), Hemoglobin concentration (MCHC and MCH), Reticulocyte count, Platelet

count, Iron, Transferring, Ferritin, International Normalized Ratio (INR), Activated Partial Tromboplastin Time (APTT), Cholesterol (Total, HDL, LDL, Triglyceride)

Planned biochemical analysis

Glial Acidic Fibrillary Protein (GFAP):

All plasma samples for GFAP and occludin measures will be analyzed with ELISA essay kits from the same batch at the Department of Clinical Biochemistry, Aarhus University Hospital Hospital. Transport and shipment of samples will be done on dry ice to assure stability of the specimens. The samples will be marked with the unique research identification number. Cryotubes not used to measure GFAP and occludin will be stored as part of a biobank.

MicroRNA analysis:
MicroRNAs will be identified with Illumina next-generation sequencing using the TruSeq Small RNA Sample Preparation kit (Illumina) which allows for the addition of unique barcode sequences to each sample. Such barcoding allows pooling and simultaneous sequencing of multiple samples in a single-sequencing run on the Illumina NextSeq500 sequencer thereby significantly reducing the cost of sequencing. Pooling of multiple samples will generate adequate data amounts for detailed miRNA profiling, yet at limited costs. Data generated from the NextSeq500 sequencer will be filtered based on sequence quality as part of our established bioinformatics pipeline. This will also include matching the filtered data to annotated RNA databases such as miRNA sequences from miRBase (mirbase.org).
The output from our bioinformatic pipeline will be quantitative miRNA expression levels for each sample, which will form the basis for an miRNA differential analysis where miRNAs with statistically significant expression changes will be found.
The microRNA (and extraceullar vesicles) analysis is not a mapping of the genome. The microRNA profile is a snapshot of microRNA activity at the specific time of the blood sampling. Levels of microRNA are changing constantly and with the current knowledge on the topic it is impossible to predict a risk of developing specific diseases in the future. Cryotubes not used to microRNA/exosome analysis will be stored as part of a biobank.

Extracellular vesicle analysis:

Extracellular vesicles (EVs, also known as exosomes) will be isolated from plasma samples before characterization of surface markers and content. EVs are isolated by a number of different techniques, ultra centrifugation, precipitation, size exclusion chromatography among others. While protein characterization will be done using classical molecular biological techniques such as ELISA and Western blots in addition to array techniques, EVarray that utilises a panel of antibodies directed against known EV surface markers. These analyses might be supported by proteomic analysis of all proteins as well as post-translational modifications such as phosphorylations and glycosylations. To broaden the feasibility of finding stroke type specific EV surface markers, we will utilise recombinant antibody library techniques to find novel disease binders with the potential of diagnosing stroke types in blood samples.
In addition, the nucleic acid (DNA and RNA including miRNA) content of EVs will be analysed using next generation sequencing (NGS), qRT-PCR, and other nucleic acid detection techniques. Both the protein data and the nucleic acid data will be subjected to bioinformatic analysis using different pipelines and analysis tools depending on the dataset and the purpose of the analysis. A number of validated published databases will be used for annotation and comparison. EVs do not contain genomic DNA and the analysis are therefore not a mapping of the subjects genome. As EV secretion and nucleic acid content in these are

though to change rapidly in the body due to external and internal signals and are considered a snapshot at the specific time of blood sampling. With the current knowledge on the topic it is impossible to predict a risk of developing specific diseases in the future.

Coagulation assays:
Functional and immunologic plasma assays will be employed to analyze proteins and pathways in coagulation and fibrinolysis. The analyses will be performed in the accredited clinical laboratory and the thrombosis and hemostasis research unit at the Department of Clinical Biochemistry, Aarhus University Hospital. Plasma samples will be stored in cryotubes at -80 °C until they will be analyzed in large batches.

Ektacytometry for Erythrocytic Deformability:
The rheological properties (deformability, critical shear stress and aggregation) of RBCs are quantified using a Deformability or Elongation Index (DI or EI). A higher EI at the optimum viscosity (300 Osmolality) indicates highly deformable RBCs indicative of better microcirculation, while a lower EI indicates rigid and fragile RBCs resulting into impaired microcirculation and tissue hypoxia. Briefly, 6-uL of heparinized fresh blood is mixed with 600-uL of PVP solution (300 Osm) and transferred to a disposable kit (RheoMeditech, South Korea). The kit is placed inside the laser-assisted ektacytometer (RheoScan AnD300, RheoMeditech, Seoul, S. Korea) for automated read out, data and image collection as per the vendor's instructions.

Analytical Flow Cytometry (FC) for eryNOS3 phosphorylation (pNOS3Ser1177), APMKα1 phosphorylation and s-nitrosylation (–SNO) in RBCs:
Red blood cells are separated from 100-300-uL of freshly fixed blood samples, using a cocktail of monoclonal antibodies (mAb) to RBC-specific markers (Glycophorin A, GPA (NovusBio, USA; and Hemoglobin, Hb; Bioss Biotech, USA). To assess the functional features, after fixation and permeabilization, RBCs are incubated with antibodies conjugated to fluorochrome either directly or through secondary antibodies to s-nitrosocysteine (–SNO; Abcam, USA), AMPKα1 phosphorylation (Bioss Biotech, USA) pNOS3Ser1177 (Bioss Biotech, USA). Next, RBC samples are run through a flow cytometer (CytoFLEX S Flowcytometer (Cytoflex S (B75408)), Beckman Coulter, USA), and data is collected using CellQuest software to process for FC analysis. A titration study will be performed to determine the specific amounts of sample/antibodies needed.

Biomarker workflow:

The biomarker study will upon initialization only preparing samples for the freezer will be performed and after that 2) acute ektacytometry (RBC deformability) of fresh samples will be performed in addition. Finally, 3) acute preparation of samples for flowcytometry will be added. This "three-tier" approach will be performed to ensure high quality of the handling and preparation of all samples

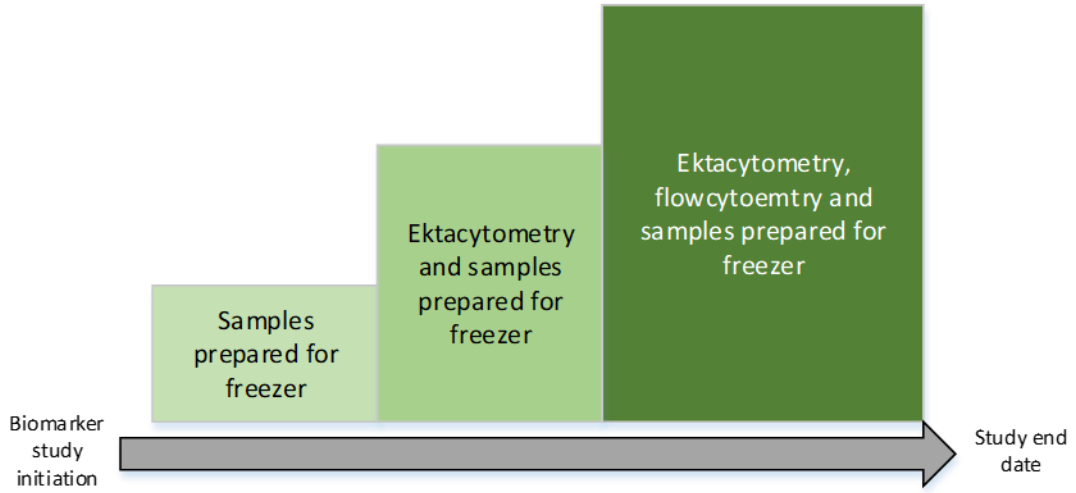

Prestroke physical activity measures in AIS and ICH patients

(Substudy only at Aarhus University Hospital)

Patient and their relatives will be asked to complete the physical activity in the elderly (PASE) questionnaire during the acute hospital admission. In patients who are unable to complete it themselves an structured interview based on the PASE questionnaire will be performed[41,42]
The PASE is a 12-item questionnaire, which quantifies the amount of PA over a 7-day period. The PASE questionnaire was developed with the purpose of assessing the level of PA in middle-aged and elderly individuals. The PASE score is calculated by taking the average number of hours spent on an activity (sports, occupational activity, household activities, and leisure time activities) per day over a 7-day period multiplied by an activity coefficient. Item scores are added to reveal the PASE score. The PASE score may range from zero to more than 400.

One week blood-pressure reduction in AIS and ICH patients

(Substudy at Aarhus University Hospital)

To determine whether RIC can reduce blood pressure (Systolic, diastolic, mean arterial pressure (MAP) and pulse) levels during one week of treatment.

Reduction in BP (baseline measurement (first) versus 7 day measurement (last)):

$$\Delta BP = BP_{baseline} - BP_{7\ Days}$$

9. Benefit of the study

Potential benefits:

Participating patients with AIS and ICH may experience an immediate reduction of their neurological symptoms and a persistent reduction of disability at long-term follow-up.

Disadvantage:

Mild-to-moderate pain and petechiae in the RIC-treated arm may occur during the inflation of the blood pressure cuff. Otherwise, the RIC treatment has been proven safe and without side effects.

Sham-RIC will only be associated with a slight sensation of pressure on the upper extremity.

Blood samples (only patients transported to and admitted at Aarhus University Hospital) of approximately 58mL (19mL prehospital and 2x19,5 mL in-hospital) will be drawn.

An additional (compared to standard operating procedure) head CT will be performed at 7 days in ICH patients included at Aarhus University Hospital (If possible, the scan will be performed at the hospital were the patient is admitted at day 7). The estimated added whole-body radiation dose of the 7 days CT-scan is on average 1,5 mSv (range 1,05 to 1,95) corresponding to 6 months of accumulated background radiation in Denmark (Danish Health Authority). This corresponds to an estimated increased life-time cancer risk of 0.005%.

10. Assessment of safety

Emergency unblinding procedure

All on call neurologists will have access to electronic case report form using a personal and password protected login to perform randomization of study participants. In cases where emergency unblinding is necessary the on call neurologist will logon to electronic CRF at ddsc.dk and enter the civil registration number (CPR) of the patient. The treatment can now be unblinded. All changes will leave an audit-trail.

Adverse events

Patients are monitored in the stroke unit with NIHSS scoring and Scandinavian Stroke Scale (SSS) at close intervals, and adverse events are treated according to clinical guidelines.

The Data Monitoring Committee will assess safety according to primary study endpoints, recurrent stroke, myocardial infarction and mortality.

Interim analysis of safety parameters (hematoma expansion at 24 hours and mortality) in ICH will be performed regularly during the study.

During the acute in-hospital phase the patients will be asked if they have experienced any deterioration of health or new symptoms during or after treatment with the investigational device. Furthermore all patients will have a 24/7 contact number to the stroke center and a contact to study research physician and will be instructed to report any deterioration of health or new symptoms. All discharged patients (and relatives) will be given contact details to study personnel and be instructed to report any deterioration of health or new symptoms. The patients with acute ischemic stroke and intracerebral hemorrhage will once again be asked for adverse events at the 3 month telephone interview. In this group, end-of-study visit is the last telephone interview, whereas end-of-study visit for participants without a vascular diagnosis or TIA(without DWI lesion) is at the time of discharge from the stroke center.

All patients, who have consented, are followed (through Danish Health Registries, LPR and DSR) for new vascular events and mortality for up to 12 months.

Adverse events and adverse device events are defined according to ISO 14155:2011 and European Commission guideline on medical devices (MEDDEV 2.7/3 revision 3).

Adverse event (AE):

Any untoward medical occurrence, unintended disease or injury or any untoward clinical signs (including an abnormal laboratory finding) in subjects, users or other persons whether or not related to the investigational medical device.

Serious Adverse Event (SAE)

Adverse event that:

a) led to a death, injury or permanent impairment to a body structure or a body function.

b) led to a serious deterioration in health of the subject, that either resulted in: - a life-threatening illness or injury, or - a permanent impairment of a body structure or a body function, or - in-patient hospitalization or prolongation of existing hospitalization, or - in medical or surgical intervention to prevent life threatening illness

Device Deficiency

Inadequacy of an investigational medical device related to its identity, quality, durability, reliability, safety or performance. This could be malfunctions, use error, or inadequacy in the information supplied by the manufacturer.

Adverse Device Effect

Adverse event related to the use of an investigational medical device.

This includes any adverse event resulting from insufficiencies or inadequacies in the instructions for use, the deployment, the implantation, the installation, the operation, or any malfunction of the investigational medical device.

This includes any event that is a result of a use error or intentional abnormal use of the investigational medical device.

Serious Adverse Device Effect (SADE)

Adverse device effect that has resulted in any of the consequences characteristic of a serious adverse event.

Unanticipated Serious Adverse Device Effect (USADE)

Serious adverse device effect which by its nature, incidence, severity or outcome has not been identified in the current version of the risk analysis report.

Anticipated SADE (ASADE): an effect which by its nature, incidence, severity or outcome has been previously identified in the risk analysis report

Event report and causality assessment

The relationship between the use of the medical device and the occurrence of each adverse event will be assessed and categorized. All events will be registered in the electronic CRF and reported to the authorities at the interim analysis. A yearly safety report, containing a list of all SAEs/SADEs and near-miss incidents, thorough evaluation of individual events, risk/benefit analysis of the investigational medical device and safety conclusions, will be submitted to the Danish Health and Medicines Authority and the Independent Ethics Committee by the Sponsor as required by Danish law.

Exceptions to and special considerations about reporting procedures are stated below:

Acute stroke is an acute life threatening disease with a high risk of neurological deterioration and mortality. The natural history of stroke is associated with a high risk of complications (stroke in progression, hemorrhagic transformation, dysphagia, cardiac arrhythmia, pneumonia, and other serious infections). These *complications* all relates to the index stroke (occurring *before* randomization) and is foreseeable. If an event occurs that based on the assessment by the investigator is a result of the natural history of the disease it will not be reported by the sponsor to the Danish medicines agency and regional ethics committee within 7 days/2 days (see list of foreseeable events/complications below).

All expected serious adverse events will be reported to the Danish Medicines Agency (and regional ethics committee) every 3 months.

All *new* serious events (Serious Adverse Events), complications that are not foreseeable or device near incidents or malpractice will be reported by the sponsor to the authorities (within 7 days, or 2 days if there is a risk of event reoccurrence) according to ISO 14155:2011 and European Commission guideline on medical devices (MEDDEV 2.7/3 revision 3). Investigators will report the event to sponsor (within 24 hours). Sponsor will as fast as possible and no later than 7 days, or 2 days if there is a risk of event reoccurrence, report the event to the Danish Medicines Agency and regional ethics committee.

All Serious Adverse Device Effect (SADE) will be reported to the Danish Medicines Agency (and regional ethics committee) within 7 days, or 2 days if there is a risk of event reoccurrence

List of foreseeable adverse events/complications

Foreseeable events/complications that will not be reported by the sponsor to the Danish medicines agency and regional ethics committee within 7 days/2days.

Patient Population	Event	Frequency	Mitigation/treatment
**TIA and AIS**	Recurrent stroke.	10% at 90 days [a]	Standard medical treatment, intravenous thrombolysis/mechanical thrombectomy according to patient characteristics
**ALL**	Symptomatic intracerebral hemorrhage (+/- related to IV tPA or EVT)	7% [b]	Antithrombotic/anticoagulation therapy cessation. Antifibrinolytic agent. Bloodpressure control.
**AIS, ICH**	Early (<24hours) and late (>24hours) neurological deterioration<br>(ΔNIHSS ≥2 or death)	7-22% [c]	According to etiology
**AIS, ICH**	Aspiration pneumonia	5-20% [d]	Antibiotic treatment according to hospital SOP
**AIS, ICH**	Urinary tract infections	12% [e]	Antibiotic treatment according to local hospital SOP

**AIS, ICH**	Severe infections (sepsis)	13% [f]	Antibiotic treatment according to local hospital SOP
**AIS, ICH**	Cardiac arrhythmia	29% [g]	Depending on arrhythmia type. Treatment according to cardio.dk
**AIS, ICH**	Acute myocardial infarction	6% [h]	Treatment according to cardio.dk and conference call with cardiologist
**AIS, ICH**	Deep venous Thrombosis(DVT)/pulmonary embolism(PE)	DVT: 8% [i]<br>PE:1% [j]	Treatment according to cardio.dk
**AIS, ICH**	Falls (in-hospital phase)	14-65% [k, l]	Treatment according to type of injury
**AIS, ICH**	Seizure	AIS:3-10% [m]<br>ICH:8% [n]	Treatment according to local SOP
**AIS/ICH**	Mortality 30 days	AIS:10-20% [o]<br>ICH:30% [p]	
**AIS**	tPA induced angioedema	3% [q]	Treatment according to local SOP

[a)] Wang Y et al. Clopidogrel with Aspirin in Acute Minor Stroke or Transient Ischemic Attack. NEJM. 2013; 369:11-19

[b)] Berkhemer OA. A Randomized Trial of Intraarterial Treatment for Acute Ischemic Stroke. NEJM. 2015; 372:11-20

[c)] Kwan J et al. Early neurological deterioration in acute stroke: clinical characteristics and impact on outcome Journal of the Association of Physicians, 2006, Sept; 99:9: 625-633

[d)] Hannawi Y et al. Stroke-Associated Pneumonia: Major Advances and Obstacles. Cerebrovasc Dis. 2013;35:430-443

[e)] Bogason E. Urinary Tract Infections in Hospitalized Ischemic Stroke Patients: Source and Impact on Outcome. 2017;9(2):e1014. doi:10.7759/cureus.1014.

[f)] BergerB. Epidemiologic features, risk factors, and outcome of sepsis in stroke patients treated on a neurologic intensive care unit. Journal of Critical care. 2013;29(2)

g)Daniele O et al. Stroke and cardiac arrhythmias. J Stroke Cerebrovasc Dis. 2002 jan-feb;11(1):28-33

[h)] Johnston KC et al. Medical and neurological complications of ischemic stroke: experience from the RANTTAS trial. RANTTAS Investigators. Stroke. 1998;29(2):447

[i)] Bembenek J. Early stroke-related deep venous thrombosis: risk factors and influence on outcome. J thromb Thrombolysis. 2011 Jul; 32(1):96-102

[j)] Pongmoragot J. et al. Pulmonary Embolism in Ischemic Stroke: Clinical Presentation, Risk Factors, and Outcome. J Am Heart Ass. 2013 Dec;2(6)

[k)] Davenport RJ, Dennis MS, Wellwood I, Warlow CP. Complications after acute stroke. Stroke. 1996; 27: 415–420.

[l] Teasell R, McRae M, Foley N, Bhardwaj A. The incidence and consequences of falls in stroke patients during inpatient rehabilitation: Factors associated with high risk. Arch Phys Med Rehabil. 2002; 83: 329–333.

[m)] Bladin C, Alexandrov AV, Bellavance A et al: Seizures After Stroke: A Prospective Multicenter Study.Arch Neurol. 2000;57(11):1617-1622.

[n)] Woo K-M, Yan S-Y, Cho K-T. Seizures after Spontaneous Intracerebral Hemorrhage. J. Korean Neurosurg Soc. 2012 Oct;52(4)312-319

[O)]Schwartz J et al. Incorporating Stroke Severity Into Hospital Measures of 30-Day Mortality After Ischemic Stroke Hospitalization. Stroke. 2017. Ahead of print: https://doi.org/10.1161/STROKEAHA.117.017960

[p)] Lichtman JH et al. 30-Day Mortality and Readmission after Hemorrhagic Stroke among Medicare.. Stroke. 2011.42(12)3387-91

[q] Sczepanski M. et al. Institutional Incidence of Severe tPA-Induced Angioedema in Ischemic Cerebral Vascular Accidents. Crit Care Res Pract. 2018; 9360918

List of anticipated adverse device effects

RIC device	Frequency	Mitigation/treatment
**Local petechiae**	4-5% [a]	none
**Discomfort/pain**	20-30%	Treatment stopped if the non-competent patient verbally or non-verbally demonstrates intolerable discomfort or if the competent patient verbally want to discontinue the treatment
**Near events (device related)**	None known in the literature	
**Acute limb ischemia (upper extremity) – theoretical complication.**	None known in the literature	Treatment stopped. Treatment according local hospital SOP's for acute limb ischemia.<br>Known atherosclerotic stenosis in upper extremities are ***an exclusion criteria.***<br>See Investigators Brochure for further details.
**Sham RIC device**		
**None known**		

a) Hausenloy D et al. Remote Ischemic Preconditioning and Outcomes of Cardiac Surgery. New England J. Medicine. 2015 Oct;373(15):1408-17

Treatment and mitigation of any adverse events will be according regional guidelines.

Adverse events as a result of standard treatment will be handled according to local guidelines. Adverse events may also occur as a suspected reaction to RIC/Sham RIC – in these cases, the conditioning stimulus will be stopped immediately and appropriate treatment will be initiated.

In cases of SAE and device related near events all patients will be followed until resolution of symptoms/signs and/or all clinical relevant treatment have been performed and/or until the symptoms/signs are in a stable phase.

11. Project timetable and recruitment feasibility

Study preparation: January to February 2017

Study start date: March 2018

Month 0-60: Patient recruitment

Expected inclusion end date: 31. December 2022

Month 60-66: Study analysis

Expected end date for last analysis and follow-up: 31. December 2024

Patient inclusion per center:

Aarhus University Hospital: 50%

Regional Hospital West Jutland (Holstebro): 12,5%

Odense University Hospital: 12,5%

Aalborg University Hospital: 12,5%

Zealand University Hospital (Roskilde): 12,5%

12. Ethical considerations

The study will await approval from the Regional Ethical Committee, the Danish Health Authority, and the Danish Data Protection Agency. Enrollment of additional Danish stroke centers will require a new application to the Danish Medicines Agency and the regional ethics committee (amendment).

A large acute stroke results in immediate, major neurological deficits (+/-aphasia) and a substantially increased risk of a long-term reduction of functional capability. This group is assessed as legally incompetent. Even in patients suffering from minor stroke, acute cognitive impairment are common, making individual preference on acute treatment options impossible. In order to preserve brain tissue, the intervention must be applied without delay. Improvement of standard care in patients suffering from acute stroke is of paramount importance. Inclusion of all stroke patients is necessary in order to translate any positive research results into a benefit for all stroke patients. The intervention is without any known risk apart from moderate pain in the upper extremity when the cuff is inflated. We find that the conditions for an acute study are satisfied since the majority of the patients are incapacitated at stroke onset and the treatment has to be initiated as soon as possible and there are no reported serious side effects from RIC.

Study participants are covered in accordance with the Danish Patient Insurance Act.

13. Data handling and record keeping

All study data are recorded in an electronic CRF with blinded data and identification via a study identification number.

The study will apply to the specifications of act on processing of personal data.

The database(e-CRF) is handled by OPUS consult Aps, Egaa, Denmark, who already handles the Danish Stroke Center Database in Aarhus (ddsc.dk).

Results from the biomarker substudies will recorded and managed using REDCap electronic data capture tools hosted at Aarhus University Hospital.

Remote Ischemic Conditioning in Stroke (RISC) is a predefined collaboration in which three large RIC trials have agreed to share anonymized patient data for a large metaanalysis once the individual trials have ended (Principle Investigators of the individual trials are: Grethe Andersen (DK), Philip Bath (UK) and Fernando Pico (France).

Methodological experts from collaborators at Medical College Georgia, Augusta, USA (Dr. Hess' and Dr. Baban's lab) will remotely supervise the technical aspects of flowcytometry and ektacytometry and assist in the interpretation of results (only anonymized patient data). *Flowcytometry and ektacytometry results reflects only snapshots of the function and deformability of the red blood cells and change rapidly.*

Remote Ischemic Conditioning in Stroke (RISC) is a predefined collaboration in which three large RIC trials have agreed to share anonymized patient data for a large metaanalysis once the individual trials have ended (Principle Investigators of the individual trials are: Grethe Andersen (DK), Philip Bath (UK) and Fernando Pico (France).

Patient participation will be recorded in the medical record. Data will be stored at the Department of Neurology, Aarhus University Hospital, for 15 years, after which the documents will be shredded.

A notification to The Danish Data Protection agency will be submitted.

During the entire study period and at all participating centers the local GCP unit will perform quality assurance control including source data verification.

A complete list of all source data will be made and approved by the local GCP unit before study initiation.

The investigator permits direct access to all source data/documents (including electronic patient record) at monitoring visits, audits and/or inspections by the regional ethics committee and Danish Medicines Agency.

14. Publications policy

The results of the study, both negative, inconclusive and positive, will be disseminated as widely as possible - through publication in an international peer-reviewed journal, as conference presentations and on www.clinicaltrials.gov.

The trial is registered on ClinicalTrials.gov with Identifier: NCT03481777

15. Statistics

Primary study endpoint:

Difference in clinical outcome (mRS) at 3 months in acute ischemic stroke *(General ordinal logistic regression analysis. Significance level of 5%)*

The **main analysis of the primary study endpoint** will be performed using the entire range of the modified Rankin Scale **(General ordinal logistic regression, unadjusted)** on the population fulfilling the ***in-hospital inclusion criteria (target population)***. The trial success analysis is performed on the **target population** which consists of prehospital randomized patients with an in-hospital diagnosis of acute ischemic stroke (including TIA with a DWI lesion) and intracerebral hemorrhage (ICH) regardless of adherence to investigational treatment (**modified Intention-To-Treat analysis**).

Supplementary safety analysis:

All patients randomized in RESIST had pre-randomization a focal neurological deficit (documented on the prehospital stroke score). The presence or absence of specific focal neurological symptoms are assessed again after 24 hours using the same score as pre-randomization (PreSS) in all randomized patients (PreSS 24 hour/ at discharge). The primary safety analysis will be made on the **entire randomized population using the difference in prehospital stroke score** (PreSS) to document change in neurological deficits between pre-randomization and 24 hour/discharge **(General ordinal logistic regression, unadjusted)**. There has been no safety concern over RIC treatment in patients with non-vascular diagnosis[23,27,28,29,30]

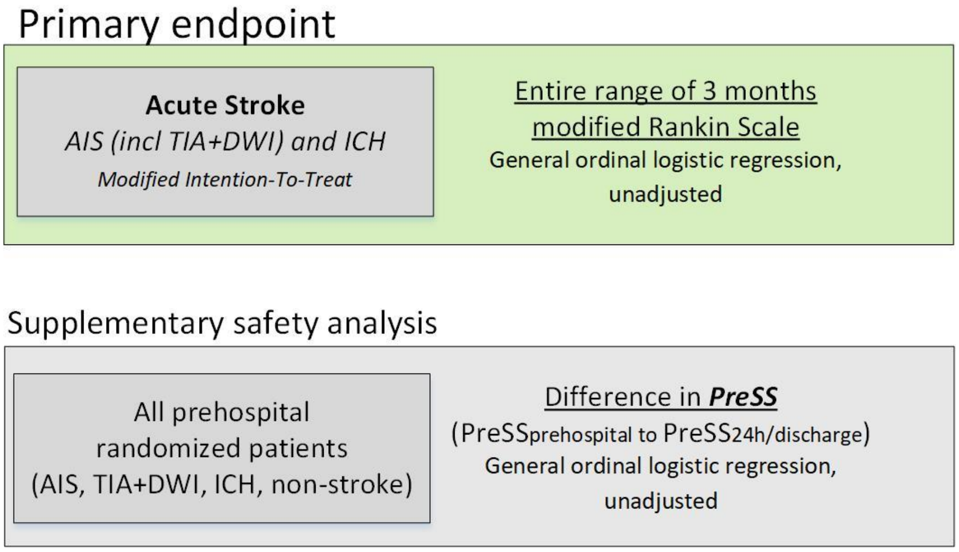

If remote ischemic preconditioning is associated with ultra-early treatment effect and reduced symptom severity (in the ambulances), this could affect the in-hospital indication for IV-tPA and/or EVT (in the case of rapid improvement of symptoms or mild symptoms)

Because of this, a **supplementary analyses** using **general ordinal logistic regression, adjusted for a possible** imbalances in reperfusion treatments (IV-tPA and EVT) between treatment arms will be made.

Secondary study endpoints (selected):

- Difference in prehospital stroke score (PreSS) (Supplementary safety endpoint, see above) (All randomized patients, general ordinal logistic regression, significans level 5%)
- Difference in prehospital stroke score (PreSS) during the first 24 hours: (general ordinal logistic regression analysis in the stroke subtypes, Significance level of 5%)

- Difference in early neurological improvement in AIS and ICH *(general ordinal logistic regression analysis. Significance level of 5%)*
- Difference in prehospital stroke score (PreSS) after 24 hours in subgroups: IV tPA/EVT treated AIS and ICH. (ordinal logistic regression analysis, significance level of 5%)
- Difference in acute hematoma expansion in patients with ICH (Binomial regression analysis, significance level of 5%)
- Difference in acute infarct growth in patients with AIS treated with IV-tPA and/or EVT (Binomial regression analysis, significance level of 5%)
- Major adverse cardiac and cerebral events (MACCE) and recurrent ischemic events at 3 and 12 months in AIS patients *(Cox regression analysis, significance level of 5%)*
- Three-month and one-year mortality *(Two sample test of proportion (Chi-square test), significance level of 5%)*
- EVT-eligibility (MRI assessed) in RIC treated AIS patients with large vessel occlusion (LVO) *(Binomial regression analysis. Significance level of 5%)*

Sample Size

The treatment effect of RIC on long-term functional outcome is unknown. We have assumed a small but clinical significant neuroprotective effect corresponding to a 7% increased odds for a beneficial shift on the modified Rankin Scale. The sample size calculation was based on a simulation-based approach to the analysis of statistical power when ordinal logistic regression analysis is performed (significans level of 5%)

The statistical power was simulated at different hypothetical sample sizes (on the target population) (ranging from 200 to 1900) with 2000 simulation-runs performed at each step. Unpublished data on IV-tPA and/or EVT treated AIS patients and ICH patients from our institution were used:

3 months modified Rankin Scale distribution (proportions) in 2017 for patients with ICH or IV tPA/EVT treated AIS at Aarhus University Hospital.

**Modfied Rankin Scale**	**mRS 0**	**mRS 1**	**mRS 2**	**mRS 3**	**mRS 4**	**mRS 5**	**mRS 6**
Proportion	0.139	0.273	0.141	0.110	0.145	0.07	0.126

Based on our previous trial experience with prehospital remote ischemic conditioning we estimate that a sample of 1000 subjects with target diagnosis (AIS and ICH) will be feasible to include during the study period.

Thus, including 1000 patients with target diagnosis provide sufficient power at a alfa-level (significans) of 5% to detect RIC treatment effects of the estimated 7 % *(see table below)*

**Treatment effect**<br>**(assumed neuroprective)**	5%	6%	**7%**
Sample (target diagnosis), *n*	1000	1000	**1000**
Power	66%	80%	**90%**
Alfa level (significans level)	5%	5%	**5%**

The estimated prehospital, randomized, sample:

Sample size, prehospital	Proportion of randomized	*n =*
**Target diagnosis (AIS and ICH), *n***	**67%**	**1000**
Non-vascular diagnosis	**27%**	**403**
TIA without DWI lesion	**4%**	**60**
Lost to follow-up	**2%**	**30**
Total		**1492**
**Plan to include**		**1500**

We therefor plan to include 1500 patients with a prehospital putative stroke in order to get 1000 patients with the target diagnosis of acute ischemic stroke and intracerebral hemorrhage. There is no planned replacement of patients lost to follow-up.

Interim analysis

We will perform an interim analysis for evaluation of the actual event rate after inclusion of 50% of the patients. If this shows a lower good outcome rate than expected, a new sample size calculation will be performed. Furthermore, the Data monitoring committee (DMC) will perform an independent safety analysis and will review the overall safety and efficacy according to the DMC charter (planned in the early phase of the trial, at 12 month and hereafter at least every 12. months) An additional safety interim analysis of mortality and hematoma expansion in ICH patients will be performed. Interim analysis are assessed using a level of significance of 1%.

The trial can be stopped at interim analyses for both futility, efficacy and safety reasons.

Detailed description is found in the DMC charter.

Missing data:

Missing data with be handled using multiple imputation (predictive mean matching imputation).

Deviations from the statistical plan:

All deviations from the statistical plan will first be applied when approved by the regional ethics committee and the Danish Medicines Agency.

Trial decision making and stopping rules

A Data Monitoring Committee (DMC) will be appointed before trial start and include a signed DMC charter. The DMC will review the accumulated data during RESIST trial and provide advice on the conduct of the trial to the Steering Committee.

The DMC should inform the Steering Committee

if, in their view:

1. the results are likely to provide convincing evidence that one trial arm is clearly indicated or contraindicated, and there is a reasonable expectation that this new evidence would materially influence patient management

or

2. it is beyond doubt that no clear outcome would be obtained (futility)

It is not expected that RESIST trial will be stopped for reasons of efficacy or futility based on observed treatment differences. A predefined pooling of individual patient data (Remote Ischemic conditioning in Stroke Collaboration, RISC) is planned. To dispel scepticism, a robust demonstration of efficacy of this simple intervention is required. The primary reason to recommend stopping the trial will be for safety reasons.

For Acute Ischemic Stroke and Intracerebral Hemorrhage:

- For the primary endpoint the DMC will consider stopping if there is a robust improvement in functional outcome after 3 months (modified Rankin Scale) for Acute Ischemic Stroke and Intracerebral haemorrhage in the sham (control) group compared to the intervention (RIC) group, achieving $p<0.01$.
- A substantial number of patients experience serious adverse events ($p<0.01$)
- DMC will consider stopping if there is an increase in mortality or stroke recurrence for Acute Ischemic Stroke and Intracerebral Hemorrhage patients in the intervention (RIC) group compared to the control (sham) group, achieving $p<0.01$.
- For ICH the DMC will consider stopping/modification if there is a clear evidence of hematoma growth during the first 24-hour in the intervention group, achieving $p<0.01$

The DMC will make recommendations, which could include:

- No action needed, trial continues as planned
- Early stopping due, for example, to clear benefit or harm of a treatment, futility, or external evidence
- Stopping recruitment within a subgroup
- Extending recruitment (based on actual control arm response rates being different to predicted other than on emerging differences) or extending follow-up
- Approving and/or proposing protocol changes

16. Source data access and monitoring

Trial-related audits and/or monitoring will be provided by direct access to source data/documents. Local monitors from the Unit for Good Clinical Practice, Aarhus University, will perform the audit and monitoring. Audit and monitoring will, likewise, be perfomed by qualified local GCP-monitors when enrollment at other stroke centers in Denmark is starting. Before enrollment can start a trial monitoring plan must be available. The audit and monitoring process will involve a 100% monitoring of signed consent forms ("samtykkeerklæringer og fuldmagtserklæringer") and serious adverse events.

17. Economy

Study initiator is Grethe Andersen, Senior Consultant, MD, DMSc, Professor of Neurology at Department of Neurology, Aarhus University Hospital.

The Danish foundation Vilhelm Petersens mindelegat has supported the study with 1.240.000 dkr (research nurse salary and "stroke RIC" devices).

The Danish foundation TrygFonden has supported the study with 4.000.000 dkr (research personnel salaries, running costs, micro RNA analysis and investigational devices).

The Danish foundation Novo Nordisk Foundation has supported the study with 2.562.153dkr (Salary for Research nurses, technicians and analysis costs for ektacytometry and flowcytometry)

Aarhus University has supported the study with 550.000 dkr (study investigator/coordinator salary)

The funders have no role in study design, data collection, analysis or interpretation, nor the decision to publish or the preparation, review and approval of the manuscript.

Stroke RIC/Sham-RIC device is developed in collaboration with Aarhus University, Faculty of Biomedical Engineering, 8200-DK, Aarhus N, Denmark; Seagull Aps, 4160-DK, Herlufmagle, Denmark and the Department of Neurology, Aarhus University Hospital, 8000-DK, Aarhus C, Denmark. Seagull Aps has no role in study design, data collection, analysis or interpretation, nor the decision to publish or the preparation, review and approval of the manuscript.

The research grants are transferred directly to a research account administered by the financial department of Aarhus University Hospital. None of the involved doctors or research nurses has any conflict of interest or economic advantages in regards to the study. There is no economic compensation or reimbursement for patients participating in the study.

Supplements

Supplement A - Neuroimaging protocol

On admission, either CT or MRI is performed according to hospital SOP.

All AIS and ICH patients can be included in the in-hospital RIC/sham-RIC based on a baseline native CT. However, patients with complete remission of symptoms after randomization (TIA) require an MRI at baseline demonstrating acute ischemic lesion on DWI in order to continue the randomized treatment. TIA patients with a documented ischemic lesion are, per definition, handled as ischemic stroke in this study. Baseline MRI in TIA patients is performed before the next RIC series (MRI <6 hours from RIC/sham-RIC last cuff). Patients with AIS who are not eligible for IV tPA due to contraindications will have CT/MRI before their next RIC/sham-RIC series (< 6 hours from RIPerC last cuff) according to hospital SOP. All ICH patients will have a baseline and 24-hour CT (hospital SOP)

Non-stroke diagnosis and TIA (only evaluated with CT or TIA without DWI lesion on MRI) will not be included in the in-hospital RIC study and will receive no further RIC treatment or neuroimaging.

Imaging protocol	Accepted time frame
MRI baseline – AIS IV tPA eligible	Acute on admission
CT baseline – AIS IV tPA eligible	Acute on admission
MRI/CT baseline – AIS non IV tPA eligible	< 6 hours from last RIC cuff
MRI (24-hour) - tPA/EVT treated AIS patients*	18-30 hour from baseline MRI
MRI baseline – ICH	Perform CT < 3 hours from last RIC cuff, if possible
CT baseline – ICH	< 6 hours from last RIC cuff
Control CT (24-hour) – ICH*	18-30 hour from baseline CT
Control CT (7-day)*	5-9 days from baseline CT

*Substudy at Aarhus University Hospital

Computed tomography (CT) protocol
Baseline CT and CT angiography are obtained in centers using CT for primary assessment (according to hospital SOP).

CT analysis

Hematoma volume assessment at baseline and at 24 hours and 7 days is performed by an experienced neuroradiologist. The assessment will be assisted using an automated stroke volume assessment software (e.g. COMBAT stroke APS, Aarhus University Hospital, Denmark) or using the formula *ABC*/2 where *A* is the greatest hematoma diameter by CT, *B* is the diameter perpendicular to *A*, and *C* is the number of CT slices with hematoma multiplied by slice thickness[43].

Magnetic resonance imaging (MRI) protocol
Diffusion-weighted imaging (DWI), apparent diffusion coefficient (ADC), T2* gradient-recalled echo (T2*GRE), and T2 fluid-attenuated inverse recovery (T2-FLAIR). MR angiography time-of-flight (MRA-TOF) is performed according to hospital standard operating procedures (SOP).

The 24-hour MRI is performed on the same MRI scanner used at baseline, if possible. This protocol includes: DWI, T2*, T2 and T2-FLAIR. MRA-TOF at 24 hours is obtained if the baseline CT-angiography or MRA-TOF demonstrated vessel occlusion.

The total acquisition time for this protocol is approximately 7-10 mins (MRI)/10-15 min (MRI + MRA-TOF), depending on the MRI equipment.

MRI analysis
Acute (baseline) and 24-hour follow-up (DWI lesion will be outlined, representing irreversibly damaged tissue *(substudy at Aarhus University Hospital).*

Infarct growth is defined as the difference between baseline DWI and the 24-hour MRI-DWI lesion.

Infarct volume assessment at baseline and at 24 hours is performed by an experienced neuroradiologist. The assessment will be assisted using an automated stroke volume assessment software (e.g. COMBAT stroke APS, Aarhus University Hospital, Denmark) or using the formula *ABC*/2 where *A* is the greatest hematoma diameter by CT, *B* is the diameter perpendicular to *A*, and *C* is the number of CT slices with hematoma multiplied by slice thickness[43]

Vascular patency and reperfusion are assessed using the Thrombolysis in Cerebral Infarction Perfusion Scale (TICI) criteria[44]

- No perfusion (TICI 0)
- Perfusion past the initial occlusion, but no distal branch filling (TICI 1)
- Perfusion with incomplete or slow distal branch filling (TICI 2)
- Full perfusion with filling of all distal branches, including M3 and M4 (TICI 3)

Supplement B – Baseline data

Age	Platelet inhibitor treatment
Male/female	Anticoagulation therapy
**Medical History**	New oral anticoagulation treatment (NOAC)
Hypertension	Opioid treatment
Smoking	SSRI treatment
Alcohol	**Clinical and physiological data**
Hyperlipidemia	Prehospital Stroke Score (PreSS)
Diabetes	Modified Rankin Scale prestroke
Previous myocardial infarction<br>*Yes: within 3 months, yes: more than 3 months, no, unknown*	NIHSS (baseline)
Angina pectoris	Symptom onset (time)
Recent angina pectoris (< 4 weeks)<br>*If yes, date for latest episode*	Time of admission
Atrial fibrillation	Completed prehospital RIC/sham cycles
Previous ischemic stroke	= 5, 4, 3, 1-2, 0
Stroke < 3 month<br>*If yes, date*	Time (start/stop) for each RIC/sham cycle
Previous TIA	NIHSS (t = 24 hours)
Recent TIA < 4 weeks<br>*If yes, date for latest episode*	Stroke etiology (TOAST)
Previous ICH	Admission blood pressure
Peripheral artery disease	**IV tPA/EVT treated AIS**
Physical activity (PASE interview)<br>Substudy at Aarhus University Hospital	Treatment initiation (time)
WHO-5<br>Substudy at Aarhus University	**Quality indicators (Danish Stroke Registry)**
**Medication on admission**	**Routine lab results***<br>Substudy at Aarhus University Hospital
Statins	
Calcium channel blockers	
ACE inhibitors/angiotensin receptor blockers	
Beta-blockers	

Baseline data obtained from treating physician, database of Danish stroke center (ddsc.dk), Danish stroke registry and electronic health records.

**Routine lab results at Aarhus University Hospital (See Biochemical section page 26-27 for details)*

References

1. Astrup J, Siesjo BK, Symon L. Thresholds in cerebral ischemia - the ischemic penumbra. *Stroke*. 1981;12(6):723-725.
2. Group N study. Tissue plasminogen activator for acute ischemic stroke. *N Engl J Med*. 1995;333(24).
3. Hacke W, Kaste M, Bluhmki E. Thrombolysis with alteplase 3 to 4.5 hours after acute ischemic stroke. *… Engl J …*. 2008:1317-1330. http://www.nejm.org/doi/full/10.1056/NEJMoa0804656. Accessed October 21, 2014.
4. Berkhemer OA, Fransen PSS, Beumer D, et al. A randomized trial of intraarterial treatment for acute ischemic stroke. *N Engl J Med*. 2015;372(1):11-20. doi:10.1056/NEJMoa1411587
5. Touma L, Filion KB, Sterling LH, Atallah R, Windle SB, Eisenberg MJ. Stent Retrievers for the Treatment of Acute Ischemic Stroke: A Systematic Review and Meta-analysis of Randomized Clinical Trials. *JAMA Neurol*. January 2016. doi:10.1001/jamaneurol.2015.4441
6. Prior A, Laursen TM, Larsen KK, et al. Post-Stroke Mortality , Stroke Severity , and Preadmission Antipsychotic Medicine Use – A Population-Based Cohort Study. 2014;9(1). doi:10.1371/journal.pone.0084103
7. Ren C, Gao M, Dornbos D, et al. Remote ischemic post-conditioning reduced brain damage in experimental ischemia/reperfusion injury. *Neurol Res*. 2011;33(5):514-519. doi:10.1179/016164111X13007856084241
8. Hougaard KD, Hjort N, Zeidler D, et al. Remote ischemic perconditioning in thrombolysed stroke patients: randomized study of activating endogenous neuroprotection - design and MRI measurements. *Int J Stroke*. 2013;8(2):141-146. doi:10.1111/j.1747-4949.2012.00786.x
9. Khatri R, McKinney AM, Swenson B, Janardhan V. Blood-brain barrier, reperfusion injury, and hemorrhagic transformation in acute ischemic stroke. *Neurology*. 2012;79(13 Suppl 1):S52-7. doi:10.1212/WNL.0b013e3182697e70
10. Campbell BC V, Christensen S, Tress BM, et al. Failure of collateral blood flow is associated with infarct growth in ischemic stroke. *J Cereb Blood Flow Metab*. 2013;33(8):1168-1172. doi:10.1038/jcbfm.2013.77
11. Ip HL, Liebeskind DS. The future of ischemic stroke: flow from prehospital neuroprotection to definitive reperfusion. *Interv Neurol*. 2014;2(3):105-117. doi:10.1159/000357164
12. Giles MF, Albers GW, Kim AS, Sciolla R. Early stroke risk and ABCD2 score performance in tissue- vs time-defined TIA A multicenter study. *Neurology*. 2011.
13. Kellner CP, Connolly ES. Neuroprotective Strategies for Intracerebral Hemorrhage Trials and Translation. 2010. doi:10.1161/STROKEAHA.110.597476
14. Anderson CS, Heeley E, Huang Y, et al. Rapid blood-pressure lowering in patients with acute intracerebral hemorrhage. *N Engl J Med*. 2013;368(25):2355-2365. doi:10.1056/NEJMoa1214609
15. Murry CE, Jennings RB, Reimer KA. Preconditioning with ischemia: a delay of lethal cell injury in ischemic myocardium. *Circulation*. 1986;74(5):1124-1136.
16. Hahn CD, Manlhiot C, Schmidt MR, Nielsen TT, Redington AN. Remote ischemic per-conditioning: a novel therapy for acute stroke? *Stroke*. 2011;42(10):2960-2962. doi:10.1161/STROKEAHA.111.622340
17. Hess DC, Hoda MN, Bhatia K. Remote limb perconditioning [corrected] and postconditioning: will it

translate into a promising treatment for acute stroke? *Stroke*. 2013;44(4):1191-1197. doi:10.1161/STROKEAHA.112.678482

18. Schmidt MR, Pryds K, Bøtker HE. Novel adjunctive treatments of myocardial infarction. *World J Cardiol*. 2014;6(6):434-443. doi:10.4330/wjc.v6.i6.434
19. Li J, Rohailla S, Gelber N, et al. MicroRNA-144 is a circulating effector of remote ischemic preconditioning. *Basic Res Cardiol*. 2014;109(5):423. doi:10.1007/s00395-014-0423-z
20. Schmidt MR, Redington A, Bøtker HE. Remote conditioning the heart overview - translatability and mechanism. *Br J Pharmacol*. September 2014:1-37. doi:10.1111/bph.12933
21. Hoda MN, Bhatia K, Hafez SS, et al. Remote ischemic perconditioning is effective after embolic stroke in ovariectomized female mice. *Transl Stroke Res*. 2014;5(4):484-490. doi:10.1007/s12975-013-0318-6
22. Lim SY, Hausenloy DJ. Remote ischemic conditioning: from bench to bedside. *Front Physiol*. 2012;3(February):27. doi:10.3389/fphys.2012.00027
23. Hougaard KD, Hjort N, Zeidler D, et al. Remote ischemic perconditioning as an adjunct therapy to thrombolysis in patients with acute ischemic stroke: a randomized trial. *Stroke*. 2014;45(1):159-167. doi:10.1161/STROKEAHA.113.001346
24. Meng R, Asmaro K, Meng L, et al. Upper limb ischemic preconditioning prevents recurrent stroke in intracranial arterial stenosis. *Neurology*. 2012;79(18):1853-1861. doi:10.1212/WNL.0b013e318271f76a
25. Meng R, Ding Y, Asmaro K, et al. Ischemic Conditioning Is Safe and Effective for Octo- and Nonagenarians in Stroke Prevention and Treatment. *Neurotherapeutics*. 2015;12(3):667-677. doi:10.1007/s13311-015-0358-6
26. Vaibhav K, Braun M, Khan MB, et al. Remote Ischemic post-conditioning promotes hematoma resolution via AMPK-dependent immune regulation. Journal of experimental medicine. 2018. 6 sept (online). doi.10.1084/jem.20171905
27. Le Page S, Bejan-Angoulvant T, Angoulvant D, Prunier F. Remote ischemic conditioning and cardioprotection: a systematic review and meta-analysis of randomized clinical trials. *Basic Res Cardiol*. 2015;110(2):11. doi:10.1007/s00395-015-0467-8
28. Bøtker HE, Kharbanda R, Schmidt MR, et al. Remote ischaemic conditioning before hospital admission, as a complement to angioplasty, and effect on myocardial salvage in patients with acute myocardial infarction: a randomised trial. *Lancet*. 2010;375(9716):727-734. doi:10.1016/S0140-6736(09)62001-8
29. Hausenloy DJ, Candilio L, Evans R, et al. Remote Ischemic Preconditioning and Outcomes of Cardiac Surgery. *N Engl J Med*. 2015;373(15):1408-1417. doi:10.1056/NEJMoa1413534
30. Laiwalla AN, Ooi YC, Liou R, Gonzalez NR. Matched Cohort Analysis of the Effects of Limb Remote Ischemic Conditioning in Patients with Aneurysmal Subarachnoid Hemorrhage. *Transl Stroke Res*. 2016;7(1):42-48. doi:10.1007/s12975-015-0437-3
31. Hess DC, Blauenfeldt RA, Andersen G, et al. Remote ischaemic conditioning-a new paradigm of self-protection in the brain. *Nat Rev Neurol*. 2015;11(12):698-710. doi:10.1038/nrneurol.2015.223
32. Hastrup S, Damgaard D, Johnsen SP, Andersen G. Prehospital Acute Stroke Severity Scale to Predict Large Artery Occlusion: Design and Comparison With Other Scales. *Stroke*. 2016;47(7):1772-1776. doi:10.1161/STROKEAHA.115.012482
33. Kothari RU, Pancioli A, Liu T, Brott T, Broderick J. Cincinnati Prehospital Stroke Scale: reproducibility

and validity. *Ann Emerg Med*. 1999;33(4):373-378.

34. Sivakumar L, Kate M, Jeerakathil T, Camicioli R, Buck B, Butcher K. Serial montreal cognitive assessments demonstrate reversible cognitive impairment in patients with acute transient ischemic attack and minor stroke. *Stroke*. 2014;45(6):1709-1715. doi:10.1161/STROKEAHA.114.004726

35. Riepe MW, Riss S, Bittner D, Huber R. Screening for cognitive impairment in patients with acute stroke. *Dement Geriatr Cogn Disord*. 2004;17(1-2):49-53. doi:10.1159/000074082

36. Bruno A, Akinwuntan AE, Lin C, et al. Simplified modified rankin scale questionnaire: reproducibility over the telephone and validation with quality of life. *Stroke*. 2011;42(8):2276-2279. doi:10.1161/STROKEAHA.111.613273

37. Guillemin F, Bombardier C, Beaton D. Cross-cultural adaptation of health-related quality of life measures: literature review and proposed guidelines. *J Clin Epidemiol*. 1993;46(12):1417-1432.

38. Beaton DE, Bombardier C, Guillemin F, Ferraz MB. Guidelines for the process of cross-cultural adaptation of self-report measures. *Spine (Phila Pa 1976)*. 2000;25(24):3186-3191.

39. Demchuk AM, Dowlatshahi D, Rodriguez-Luna D, et al. Prediction of haematoma growth and outcome in patients with intracerebral haemorrhage using the CT-angiography spot sign (PREDICT): a prospective observational study. *Lancet Neurol*. 2012;11(4):307-314. doi:10.1016/S1474-4422(12)70038-8

40. Kappetein AP, Head SJ, Genereux P, et al. Updated standardized endpoint definitions for transcatheter aortic valve implantation: the Valve Academic Research Consortium-2 consensus document. *Eur Heart J*. 2012;33(19):2403-2418. doi:10.1093/eurheartj/ehs255

41. Schuit AJ, Schouten EG, Westerterp KR, Saris WH. Validity of the Physical Activity Scale for the Elderly (PASE): according to energy expenditure assessed by the doubly labeled water method. *J Clin Epidemiol*. 1997;50(5):541-546.

42. Washburn RA, McAuley E, Katula J, Mihalko SL, Boileau RA. The physical activity scale for the elderly (PASE): evidence for validity. *J Clin Epidemiol*. 1999;52(7):643-651.

43. Kothari RU, Brott T, Broderick JP, et al. The ABCs of measuring intracerebral hemorrhage volumes. *Stroke*. 1996;27(8):1304-1305.

44. Khatri P, Neff J, Broderick JP, Khoury JC, Carrozzella J, Tomsick T. Revascularization end points in stroke interventional trials: recanalization versus reperfusion in IMS-I. *Stroke*. 2005;36(11):2400-2403. doi:10.1161/01.STR.0000185698.45720.58

This supplement contains:

1. Final SAP (March 20, 2023)
2. Summary of changes to SAP (February 9, 2018 to March 20 2023)
3. Original SAP (February 9, 2018)

Statistical Analysis Plan

TRIAL FULL TITLE	Remote ischemic conditioning in patients with acute stroke: a multicenter randomized, patient-assessor blinded, sham-controlled study.
SAP VERSION	1
SAP VERSION DATE	20.03.2023
TRIAL STATISTICIAN	Jan Brink Valentin
Protocol Version (SAP associated with)	*version 8.2 (31March2020)*
TRIAL PRINCIPAL INVESTIGATOR	Grethe Andersen
SAP AUTHOR(s)	Rolf A. Blauenfeldt

1 SAP Signatures

I give my approval for the attached SAP entitled RESIST dated 20.03.2023

Study coordinator & Investigator

Signature: REDACTED

Date: 21.03.2023

Statistician

Name:

Signature: REDACTED

Date: 22.03.2022

Principal Investigator

Name:

Signature: REDACTED

Date: 21 mar. 2023

2 Table of Contents

1 SAP Signatures 1
2 Table of Contents 2
3 Study Objectives and Endpoints 3
3.1 Study Objectives 3
3.2 Endpoints 4
4 Study Methods 5
4.1 General Study Design and Plan 5
4.2 Inclusion-Exclusion Criteria and General Study Population 7
Inclusion criteria (prehospital) 7
Exclusion criteria 7
Final in-hospital inclusion criteria 7
4.3 Randomization and Blinding 8
4.4 Study Assessments 8
Table 1: Study procedures table 8
5 Sample Size 9
5.1 Timing of Analyses 11
5.2 Analysis Populations 11
5.2.1 Target population (Modified Intention to Treat) 11
5.2.2 Per Protocol Population 11
5.2.3 Safety Population (intention-to-treat) 11
5.2.4 Multi-center Studies 12
5.3 Missing Data 12
5.4 Interim Analyses and Data Monitoring 12
5.5 Source data access and monitoring (good clinical practice) 13
6 Summary of Study Data 14
6.1 Subject Disposition 14
6.2 Demographic and Baseline Variables 14
6.3 Coding system for MACCE and Recurrent ischemic events: 16
Recurrent ischemic vascular events at 3 and 12 months in AIS patients 16
6.4 Treatment protocol for study intervention 16
6.5 Treatment adherence 17
7 Efficacy Analyses 18
7.1 Secondary Efficacy Analyses 19
7.1.1 Secondary Analyses of Primary Efficacy Endpoint 21
8 Safety Analyses 21
9 References 22
10 Listing of Tables, Listings and Figures 23

3 Abbreviations

AE	Adverse event
AIS	Acute ischemic stroke
AR	Adverse reaction
CT	Computed tomography
CPSS	Cincinnati Prehospital Stroke Scale
DWI	Diffusion-weighted imaging
DMC	Data Monitoring Committee and Endpoints Validation Committee
ICH	Intracerebral hemorrhage
END	Early neurological deterioration
EVT	Endovascular treatment
LVO	Large Vessel Occlusion
miRNA	Micro ribonucleic acid
mPTP	Mitochondrial permeability transition pore
MRI	Magnetic resonance imaging
NPR	Danish National Patient Register
PreSS	Prehospital Stroke Score
PWI	Perfusion-weighted imaging
RIC	Remote ischemic Conditioning
RIPerC	Remote Ischemic perconditioning
RIPreC	Remote ischemic preconditioning
RIPostC	Remote ischemic postconditioning
IV tPA	Intravenous thrombolysis/recombinant tissue-type plasminogen activator
SAE	Serious adverse event
SAR	Serious adverse reaction
TIA	Transient ischemic attack
TIA (DWI+)	Transient ischemic attack with a DWI-positive lesion on MRI
TMG	Trial Management Group
TSC	Trial Steering Group

4 Study Objectives and Endpoints

4.1 Study Objectives

To determine whether Remote Ischemic Conditioning (RIC) improves the modified Rankin Scale score (mRS) at 3 months in patients with acute stroke, including acute ischemic stroke (AIS) and intracerebral hemorrhage (ICH)

Secondary aims include the association between RIC and secondary and exploratory endpoints listed below. In addition,

- Predictive abilities of Glial Fibrillary Acidic Protein (GFAP) and occludin in prehospital obtained blood samples combined with prehospital stroke severity to differentiate hemorrhagic from ischemic stroke and to grade ischemic stroke severity (*substudy at Aarhus University Hospital*)

- Diagnostic abilities of a prehospital microRNA and extracellular vesicles blood samples profile combined with prehospital stroke severity on the differentiation of hemorrhagic from ischemic stroke and to grade ischemic stroke severity (*substudy at Aarhus University Hospital*)
- microRNA and extracellular vesicle profile of RIC-induced neuroprotection at baseline (*substudy at Aarhus University Hospital*)
- Characterization of rheoerythrocrine dysfunction (RBC deformability, eryNOS3 and plasma nitrite) in RIC vs Sham-RIC treated stroke patients and its possible association to improved short term (24 hour) or long term (90 day mRS) clinical outcome or imaging biomarkers (DWI infarct growth) (*substudy at Aarhus University Hospital*)

4.2 Endpoints

Primary endpoint

- Clinical outcome (mRS) at 3 months in acute stroke (AIS and ICH)

Secondary endpoints

- Prehospital stroke score (PreSS) during 24 hours in all randomized patients
- Clinical outcome (mRS) at 3 months in subgroups: AIS, IV tPA/EVT treated AIS and ICH
- Prehospital stroke score (PreSS) during 24 hours in subgroups: IV tPA/EVT treated AIS and ICH.
- Complete remission of symptoms within 24 hours (TIA)
- Major Adverse Cardiac and Cerebral Events (MACCE) and recurrent ischemic events based on registry data at 3 and 12 months in ICH, AIS patients, TIA and non-vascular diagnosis
- Three-month and one-year mortality in AIS, ICH, and overall
- Early and very early neurological improvement in AIS and ICH patients
- Bed-day use at 3 and 12 months and quality of life measures at 3 months
- Clinical outcome (mRS) at 3 months in patients with AIS and ICH and extended remote ischemic postconditioning protocol (*substudy at Aarhus University Hospital*)
- Large vessel occlusion (LVO) eligible to EVT treatment in patients with AIS (*substudy at Aarhus University Hospital*)

Exploratory endpoint

- Hematoma reabsorption rate (7 days) in patients with ICH
- Acute (24-hour) hematoma expansion in patients with ICH
- Infarct growth in AIS patients and hematoma growth in ICH patients
- Modulation of coagulation (*substudy at Aarhus University Hospital*)
- Non stroke patients in all randomized patients

- One week blood-pressure reduction in AIS and ICH patients (*substudy at Aarhus University Hospital)*

5 Study Methods

5.1 General Study Design and Plan

Study type: Multicenter, prospective, randomized, patient-assessor blinded, sham-controlled trial.

Type of Comparison: superiority

Type of control: Sham controlled
Level and method of blinding: Patient and assessor blinded.
Randomization: 1:1 with stratification on age, center and the Prehospital Stroke Score (PreSS).
Study procedures are visualized in Figure 1 and Table1

Figure 1: Study flowchart

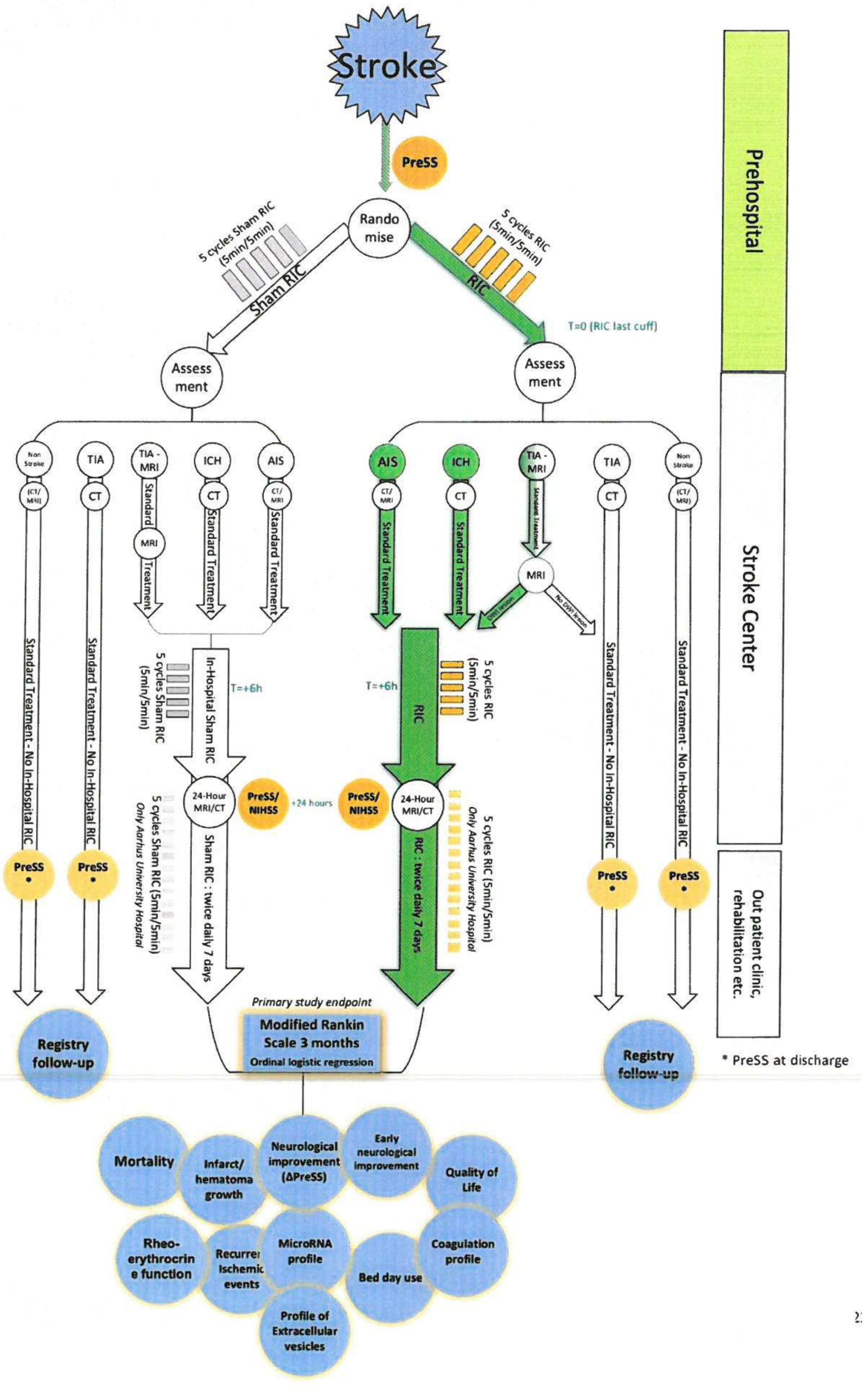
Stroke
PreSS
Rando mise
5 cycles Sham RIC (5min/5min)
Sham RIC
5 cycles RIC (5min/5min)
RIC
T=0 (RIC last cuff)
Assess ment
Assess ment
Prehospital
Non Stroke
TIA
TIA - MRI
ICH
AIS
AIS
ICH
TIA - MRI
TIA
Non Stroke
(CT/ MRI)
CT
CT
CT/ MRI
CT/ MRI
CT
CT
(CT/ MRI)
Standard Treatment
MRI
MRI
Standard Treatment
Stroke Center
Standard Treatment - No In-Hospital RIC
5 cycles Sham RIC (5min/5min)
T=+6h
In-Hospital Sham RIC
T=+6h
RIC
5 cycles RIC (5min/5min)
24-Hour MRI/CT
PreSS/ NIHSS
+24 hours
PreSS/ NIHSS
24-Hour MRI/CT
5 cycles Sham RIC (5min/5min)
Only Aarhus University Hospital
Sham RIC : twice daily 7 days
RIC : twice daily 7 days
5 cycles RIC (5min/5min)
Only Aarhus University Hospital
PreSS *
Out patient clinic, rehabilitation etc.
Primary study endpoint
Modified Rankin Scale 3 months
Ordinal logistic regression
Registry follow-up
Registry follow-up
* PreSS at discharge
Mortality
Infarct/ hematoma growth
Neurological improvement (ΔPreSS)
Early neurological improvement
Quality of Life
Rheo-erythrocrin e function
Recurrent Ischemic events
MicroRNA profile
Bed day use
Coagulation profile
Profile of Extracellular vesicles

5.2 Inclusion-Exclusion Criteria and General Study Population

All patients with a prehospital putative stroke who meet the study criteria will be included.

- Depending on the prehospital randomization (RIC versus sham), patients with AIS* and ICH will continue RIC or sham-RIC treatment for an extended period (after 6 hours at all centers and twice daily for 7 days at Aarhus University Hospital)

**Including patients with a transient ischemic attack and evident acute ischemic lesion on diffusion weighted imaging (DWI) – magnetic resonance imaging (MRI)*

Inclusion criteria (prehospital)

- Male and female patients (≥ 18 years)
- Prehospital putative stroke
- Onset of stroke symptoms < 4 hours before RIC
- Independent in daily living before symptom onset (mRS ≤ 2)

Exclusion criteria

- Intracranial aneurisms, AV malformation, cerebral neoplasm, abscess or progressive neurodegenereative disease
- Pregnancy*
- Severe peripheral arterial disease in the upper extremities
- Concomitant acute life-threatening medical or surgical condition
- AV shunt in the arm selected for RIC

**Women of child-bearing age should be asked about their use of safe birth control methods (contraceptive pill, intrauterine devices both hormonal and non-hormonal, hormonal implants, hormonal depot injection and transdermal hormonal patch). If pregnancy cannot be ruled out in the prehospital phase the patient can't be included. Women with a safe birth control method should be encouraged to use this method during the entire period of active RIC treatment.*

Final in-hospital inclusion criteria

- AIS (including DWI-positive TIA patients)

  or

- ICH

5.3 Randomization and Blinding

The patient will be randomized to standard treatment with RIC or sham-RIC by the on call neurologist/vascular neurologist at the receiving stroke center. The ambulance will contact the on call neurologist by telephone and describe the patient (standard operating procedure (SOP) in Denmark). The randomization is based on a secure web site providing computer-generated blocked randomization lists stratified by the center. The online randomization is stratified by age, stroke center and the Prehospital Stroke Score (PreSS) using a randomized block design. The PreSS score consists of the Cincinnati Prehospital Stroke Scale (CPSS) with an additional opportunity to report other neurological symptoms (e.g. ataxia, sensory disturbances and visual field loss), and PASS (Prehospital Acute Stroke Severity Scale)[1,2]. Prehospital personnel participating in RESIST are trained in identifying stroke symptoms included in PreSS[3]. The on call neurologist will make an assessment based on all available information whether the patient is eligible to participate in RESIST. Randomization is performed in the prehospital setting. Each on call neurologist participating in the study will receive unique access and will have no influence on the randomization process. All neurologists on call will be educated and trained in performing evaluation of potential eligible study candidates and to perform the online randomization during the telephone call with the ambulance personnel. There is always one neurologist on call in participating centers.

Treatment allocation

1:1 allocation

Blinding

Outcome assessment is blinded to the treatment arm and obtained by a clinical or telephone assessment of the level of dependency and need for help in daily activities (mRS) **(Primary study endpoint)**.
No information regarding randomization status will be recorded in the patient record.
Patients and the assessors of endpoints are blinded.

5.4 Study Assessments

Table 1: Study procedures table

	Acute prehospital and in-hospital phase					+ 6 hour	+ 24 hour			+ 7days		3 months	12 months
	**Prehosp. stroke score (PreSS)[1]**	**Prehosp blood-samples[1]**	**RIC/Sham-RIC**	**In-hospital assessment and blood-samples[2]**	**CT/MRI baseline**	**+6 hour RIC/sham-RIC[4]**	**PreSS 24h[2]**	**CT/MRI 24h[3]**	**24-hour blood-samples[2]**	**Extended (7 days) RIC/sham-RIC[5]**	**CT-7 days[3]**	**mRS 3-months[7]**	**Clinical events during follow-up[6]**
**TIA (DWI-) -all**	X	(X)*	X	X	X		X						X
**Non-stroke - all**	X	(X)*	X	X	X		X						X
**AIS incl. TIA**	X		X	X	X	X	X	X				X	X

(DWI+) – Other¥													
ICH - Other¥	X		X	X	X (CT)	X	X					X	X
AIS incl. TIA (DWI+) – AUH*	X	(X)	X	X	X(MRI)	X	X	X (MRI)	~~(X)~~	X		X	X
ICH – AUH*	X	(X)	X	X	X	X	X	X (CT)	~~(X)~~	X	X (CT)	X	X

**AUH = Aarhus University Hospital , ¥ Other= Other participating stroke centers*

[1] Prehospital stroke score (PreSS) and bloodsamples.

[2] Acute assessment at the Stroke Center. NIHSS, blood samples (21,5mL blood)-and ECG (standard). Assessment of neurological symptoms at 24-hour: NIHSS. Patients with non stroke diagnosis and TIA without a DWI-MRI lesion, who are discharged before 24 hours will be scored using PreSS at discharge. Patients, who had blood samples taken in the ambulance and upon stroke center arrival will have additional samples withdrawn at 2 (1-3) hours (5mL) after RIC/Sham-RIC completion and again 24(16-32) hours* after randomization (additional 21,5 mL blood) *RESIST blood samples only at Aarhus University Hospital.*

[3] Baseline CT is preferable for ICH patients (hospital SOP). ICH patients, at Aarhus University Hospital, will receive a 24 hour control CT (hospital SOP). If possible, a 24-hour MRI is performed in all included AIS patients with an MRI baseline scan. Furthermore, a 24-hour control CT will be provided for IV tPA-treated AIS patients, unless MRI can be performed and was the primary investigation. One week control CT will be performed, if possible, on ICH patients included at Aarhus University Hospital. End of study visit for non-vascular diagnosis and TIA (without DWI lesion) at stroke center discharge.

[4] RIC/sham-RIC at +6 hours from last RIC cuff

[5] RIC/sham-RIC twice daily for 7 days *(Only at Aarhus University Hospital)*

[6] Mortality, MACCE, and recurrent ischemic events are recorded using the Danish National Patient Register (LPR) and DSR at 6 and 15 months after the inclusion of the last patient.

[7] Assessments at 3 months by telephone or face-to-face (mRS and WHO-5). End-of-study (telephone or face-to-face) for AIS and ICH patients. WHO-5 will only be assessed by one assessor.

6 Sample Size

We estimate that a sample size of 1500 prehospital putative stroke patients will be required to achieve 1000 eligible AIS and ICH patients (primary study endpoint).

The treatment effect of RIC on long-term functional outcome is unknown. We have assumed a small but clinical significant neuroprotective effect corresponding to a 7% increased odds for a beneficial shift on the modified Rankin Scale. The sample size calculation is based on a simulation-based approach to the analysis of statistical power when ordinal logistic regression analysis is performed (significance level of 5%)

The statistical power was simulated at different hypothetical sample sizes (on the target population) (ranging from 200 to 1900) with 2000 simulation-runs performed at each step. Unpublished data on IV-tPA and/or EVT treated AIS patients and ICH patients from our institution were used:

3 months modified Rankin Scale distribution (proportions) in 2017 for patients with ICH or IV tPA/EVT treated AIS at Aarhus University Hospital.

Modfied Rankin Scale score	mRS 0	mRS 1	mRS 2	mRS 3	mRS 4	mRS 5	mRS 6
Proportion	0.139	0.273	0.141	0.110	0.145	0.07	0.126

Based on our previous trial experience[4] with prehospital remote ischemic conditioning we estimate that a sample of 1000 subjects with target diagnosis (AIS and ICH) will be feasible to include during the study period.

Including 1000 patients with target diagnosis provide sufficient power at an alfa-level (significance) of 5% to detect RIC treatment effects of the estimated 7 % *(see table below)*

**Treatment effect (assumed cerrebroprotective)**	5%	6%	<u>7%</u>
Sample (target diagnosis), *n*	1000	1000	**<u>1000</u>**
Power	66%	80%	**<u>90%</u>**
Alpha level (significance level)	5%	5%	**<u>5%</u>**

The estimated prehospital, randomized, sample:

**Sample size, prehospital**	**Proportion of randomized**	***n* =**
**Target diagnosis (AIS and ICH), *n***	**67%**	**1000**
Non-vascular diagnosis	**27%**	**403**
TIA without DWI lesion	**4%**	**60**
Lost to follow-up	**2%**	**30**
Total		**1492**
**<u>Plan to include</u>**		**<u>1500</u>**

We therefore plan to include 1500 patients with a prehospital putative stroke in order to get 1000 patients with the target diagnosis of acute ischemic stroke and intracerebral hemorrhage. There is no planned replacement of patients lost to follow-up.

6.1 Timing of Analyses

- The final analysis will be performed after last three month visit for the last patients or if recommended by the DMSC due to safety reasons.
  AND
- Monitoring by regional good clinical practice (GCP) unit has completed
  AND
- Closure of database by signature of the PI
  AND
- Approval of the final SAP by the PI and trial statistician

6.2 Analysis Populations

6.2.1 Target population (Modified Intention to Treat)

- *All subjects who were prehospital randomized AND had an in hospital diagnosis of Acute ischemic stroke, Transient ischemic attack with an evident acute ischemic lesion on DWI-MRI or ICH patients.*
- *All subjects disregarding adherence to study intervention*

6.2.2 Per Protocol Population

- *All subjects in the "Target population" who adhere to the major criteria treatment adherence criteria in the protocol (e.g. all subjects who received at least 80% of assigned RIC/Sham cycles (each of 5min of cuff inflation and 5 minutes without inflation = 1 cycle)*
- *All subjects who did not substantially deviate from the protocol as to be determined on a per-subject basis at the trial steering committee immediately before database lock.*

6.2.3 Safety Population (intention-to-treat)

- *All subjects who were prehospital randomized, disregarding in-hospital diagnosis, but excluding subjects who refused to give written consent or withdrew their consent.*

Primary efficacy population: Target population (AIS, TIA +DWI-MRI lesion and ICH) (Modified Intention to Treat)

Predefined subgroup analysis of the primary endpoint:

- AIS patients including TIA with DWI-MRI lesion
- AIS patients treated with intravenous thrombolysis (IVT)
- AIS patients treated with thrombectomy (EVT)
- AIS patients treated with reperfusion therapy (IVT and/or EVT)
- AIS patients not treated with reperfusion therapy (IVT and/or EVT)
- Intracerebral hemorrhage patients

6.2.4 Multi-center Studies

- Data from all centers will be analyzed as a whole
- Center effects will be presented in the subgroup analysis.

6.3 Missing Data

The association between treatment allocation and loss to follow-up is expected to be limited and explained by disease severity, pre-stroke functional level, sex, age, site, etc. The underlying assumption is depicted in the figure below. Thus, missing data incl. outcome is assumed to be missing at random and will be handled using multiple imputation chained equations with appropriate variables as model input.

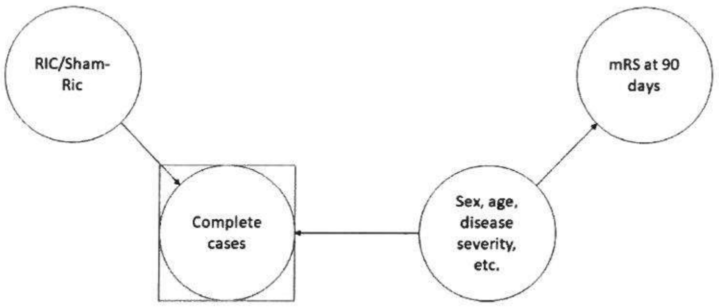

In a complete case analysis selection bias may occur on account of underlying parameters being associated with dropout rate and outcome. We assume that these parameters are available in the data set.

The extent of missing data will be presented for outcome variables to quantify the extent of missing data.

6.4 Interim Analyses and Data Monitoring

We will perform an interim analysis for evaluation of the actual event rate after inclusion of 50% of the patients. If this shows a lower good outcome rate than expected, a new sample size calculation will be performed. Furthermore, the Data monitoring committee (DMC) will perform an independent safety analysis and will review the overall safety and efficacy according to the DMC charter (planned in the early phase of the trial, at 12 month and hereafter at least every 12. Months) An additional safety interim analysis of mortality and hematoma expansion in ICH patients will be performed. Interim analyses will be assessed using a level of significance of 1%.
The trial can be stopped at interim analyses for both futility, efficacy and safety reasons. Detailed description is found in the DMC charter.

Trial decision making and stopping rules

A Data Monitoring Committee (DMC) will be appointed before trial start and include a signed DMC charter. The DMC will review the accumulated data during RESIST trial and provide advice on the conduct of the trial to the Steering Committee.

The DMC should inform the Steering Committee
if, in their view:

- the results are likely to provide convincing evidence that one trial arm is clearly indicated or contraindicated, and there is a reasonable expectation that this new evidence would materially influence patient management

or

- it is beyond doubt that no clear outcome would be obtained (futility)

It is not expected that RESIST trial will be stopped for reasons of efficacy or futility based on observed treatment differences. A predefined pooling of individual patient data (Remote Ischemic conditioning in Stroke Collaboration, RISC) is planned. To dispel scepticism, a robust demonstration of efficacy of this simple intervention is required. The primary reason to recommend stopping the trial will be for safety reasons.

For Acute Ischemic Stroke and Intracerebral Hemorrhage:
For the primary endpoint the DMC will consider stopping if:

- There is a robust improvement in functional outcome after 3 months (modified Rankin Scale) for Acute Ischemic Stroke and Intracerebral haemorrhage in the sham (control) group compared to the intervention (RIC) group, achieving $p<0.01$.
- A substantial number of patients experience serious adverse events ($p<0.01$)
- If there is an increase in mortality or stroke recurrence for Acute Ischemic Stroke and Intracerebral Hemorrhage patients in the intervention (RIC) group compared to the control (sham) group, achieving $p<0.01$.
- If there is a clear evidence of hematoma growth among patients with ICH during the first 24-hour in the intervention group, achieving $p<0.01$

The DMC will make recommendations, which could include:

- No action needed, trial continues as planned
- Early stopping due, for example, to clear benefit or harm of a treatment, futility, or external evidence
- Stopping recruitment within a subgroup
- Extending recruitment (based on actual control arm response rates being different to predicted other than on emerging differences) or extending follow-up
- Approving and/or proposing protocol changes

6.5 Source data access and monitoring (good clinical practice)

Source data access and monitoring (good clinical practice)

Trial-related audits and/or monitoring will be provided by direct access to source data/documents. Local monitors from the Unit for Good Clinical Practice, Aarhus University, will perform the audit and monitoring. Audit and monitoring will, likewise, be perfomed by qualified local GCP-monitors when enrolment at other stroke centers in Denmark is starting. Before enrolment can start a trial monitoring plan must be available. The audit and monitoring process will involve a 100% monitoring of signed consent forms ("samtykkeerklæringer og fuldmagtserklæringer") and serious adverse events.

7 Summary of Study Data

- Summary tables will be structured (columns for each treatment and overall in the order: all subjects, Sham, RIC)
- Descriptive or summary statistics that will be displayed for continuous data and for categorical data.
- The analysis populations upon which the tables and figures will be based.
    - Summary statistics on target group (Table 1) and Safety population (all randomized) in supplemental materials.

All continuous variables will be summarized using the following descriptive statistics: n (non-missing sample size), mean, standard deviation, median, 25% and 75% interquartile range. The frequency and percentages (based on the non-missing sample size) of observed levels will be reported for all categorical measures. All summary tables will be structured with a column for each treatment in the order (Sham, RIC) and will be annotated with the total population size relevant to that table/treatment, including any missing observations.

7.1 Subject Disposition

An overview of the time-dependent rates of recruitment will be provided.
Details on withdrawn consents and any reasons for that will be presented.
The study flowchart will adhere to CONSORT principles and a CONSORT statement will be made.
The reporting requirements of the regulatory agencies in Denmark will be met.

7.2 Demographic and Baseline Variables

<u>All randomized (safety population):</u> Demographic or baseline variables that will be included as a total and in each group (RIC and sham) are listed below and are intended for a supplemental table.

- Age, median (continuous)
- Female, (categorical)
- Prehospital Stroke Score (PreSS)
    - Prehospital Stroke Score item : Facial palsy (categorical, yes/no)
    - Prehospital Stroke Score item : Arm drift (categorical, yes/no)
    - Prehospital Stroke Score item : Incorrect months or age (categorical, yes/no)
    - Prehospital Stroke Score item : Speech difficulty (categorical, yes/no)
    - Prehospital Stroke Score item : eye deviation, eye muscle palsy (categorical, yes/no)
    - Prehospital Stroke Score item : Other (categorical, yes/no)

- Diagnosis (AIS, TIA +DWI-MRI lesion, ICH, TIA -DWI-MRI lesion and stroke mimics by category)

Target population: Demographic or baseline variables that will be included as a total and in each group (RIC and sham) are listed below. The anticipated statistical summary tables for the target population will include:

- Age, median (continuous)
- Female, (categorical, yes/no)
- Hypertension, (categorical, yes/no)
- Diabetes, (categorical, yes/no)
- Atrial fibrillation, (categorical, yes/no)
- Prior AIS, (categorical, yes/no)
- Prior TIA, (categorical, yes/no)
- Prehospital systolic blood pressure, (continuous)
- Prehospital diastolic blood pressure, (continuous)
- PreSS, median (continuous)
- Admission NIHSS, median (continuous)
- Discharge diagnosis (categorical, yes/no)
    - AIS
    - TIA
    - ICH
- Patients with acute ischemic stroke (categorical, yes/no)
- Stroke etiology (categorical, yes/no)
    - Large artery disease
    - Small vessel disease
    - Cardioembolic
    - Other/rare/unknown
- NIHSS, (continuous)
- Intravenous thrombolysis (categorical, yes/no)
- Admission to thrombolysis, minutes (continuous)
- Thrombectomy (categorical, yes/no)
- Door to groin puncture, minutes (continuous)
- Site of occlusion during angiography (categorical, yes/no)
    - Intracranial internal carotid artery
    - Main stem of middle cerebral artery
    - Branch from middle cerebral artery
    - Basilar artery
    - Other
    - No occlusion at time of EVT
- TICI 2b-3 reperfusion (categorical, yes/no)
- Patients with intracerebral hemorrhage (categorical, yes/no)
- Acute Glasgow coma scale score, (continuous)
- Deep hematoma localization (categorical, yes/no)
- Platelet inhibitor use (categorical, yes/no)
- Oral anticoagulation use (categorical, yes/no)

- Hematoma evacuation or external ventricular drainage (categorical, yes/no)

7.3 Coding system for MACCE and Recurrent ischemic events:

MACCE is defined as:

Cardiovascular events (cardiovascular death, myocardial infarction, acute ischemic or hemorrhagic stroke)
Cardiovascular death: Death from known cardiovascular cause or sudden death from unknown cause (no identified cause of death in medical history and/or autopsy)
Acute myocardial infarction: Admission with a discharge diagnosis of ST-elevation myocardial infarction (STEMI) and non-ST elevation myocardial infarction (NSTEMI) and unstable angina pectoris (UAP)

Stroke: Admission with a discharge diagnosis of acute ischemic or hemorrhagic stroke.
Evaluation is performed using the Danish National Patient Register (LPR) and the DSR at two time points (6 and 15 months after the inclusion of the last patient).

Diagnosis of AIS/TIA, ICH and MI (STEMI, NSTEMI, and UAP) are made according to national clinical practice guidelines.
(http://neuro.dk/wordpress/nnbv/om-iskaemisk-apopleksi/ and http://nbv.cardio.dk/aks)

Recurrent ischemic vascular events at 3 and 12 months in AIS patients
Recurrent ischemic vascular events defined as:

AIS
TIA
MI, STEMI, and NSTEMI

See "CEC charter version 1.0 (30.08.2022)" and "event adjudication, definitions and code list version 1.2 (24.11.2022)"

7.4 Treatment protocol for study intervention

Remote Ischemic Conditioning device (RIC device)
The device is programmed to five cycles (50 minutes), each consisting of five minutes of cuff inflation followed by five minutes with a deflated cuff. The cuff pressure will be 200mmHg; but if initial systolic blood pressure is above 175 mmHg, the cuff is automatically inflated to 35 mmHg above the systolic blood pressure. This is done to account for a maximum cuff air leakage of 6mmHg per minute and maximum deviation in blood pressure measurement of 10mmHg. The maximum cuff pressure is 285 mmHg.
Sham – remote ischemic conditioning device (Sham-RIC device)
The device is programmed to five cycles (50 minutes), each consisting of five minutes of cuff inflation followed by five minutes with a deflated cuff. The cuff pressure during inflation will be 20mmHg.
Timestamps, blood pressure before RIC/sham-RIC and total RIC/sham-RIC cycles are recorded and stored on the device (accessible by *universal serial bus,* USB). The RIC and sham-RIC is pre-

programmed before being delivered to collaborating centers/ambulances. No data on conditioning cuff pressure will be displayed on device screen.
In cases of short transport time, the RIC/sham-RIC protocol continues during the initial assessment at the stroke center. RIC/sham-RIC stimulation is discontinued just prior to the MRI and continues afterwards if the protocol was insufficient (< 4 cycles). In the cases of insufficient treatment before MRI a complete RIC/sham-RIC protocol will be performed (5 cycles) as soon as possible after MRI. This is done to ensure that all participants receive at least 4 consecutive cycles of RIC/sham-RIC. In centers using CT, the RIC/sham-RIC protocol is continued (if possible) during the scan until a total of 5 cycles are reached. The device automatically stops treatment when the 5 cycles are completed.
Except for RIC/sham-RIC, the prehospital observation and treatment are according to standard procedures in the ambulance.
The use of *Stroke RIC/Sham-RIC* ***was*** *approved by the Danish Medicines Agency*

Initial remote ischemic conditioning
(all patients)
RIC/sham-RIC cuff will be placed on the non-paretic upper arm. The cuff is placed on the upper extremity on the same side of the suspected side of cerebral stroke, e.g. in right hemiparesis the cuff on the left arm, and in monosymptomatic aphasia the cuff is placed on the left arm.
Remote ischemic conditioning at +6 hours
(All patients with AIS, ICH – all centers)
At 6 hours after the first RIC/sham-RIC protocol (6 hours from the last cuff-off, respectively), another series of RIC/sham-RIC is performed; five cycles each consisting of 5 minutes of ischemia are followed by 5 minutes of reperfusion. *In the case of mechanical thrombectomy the +6 hours treatment may be delayed and will be started as soon as possible.*
Remote ischemic <u>post</u>conditioning (twice daily for 7 days)
(AIS and ICH – only at Aarhus university Hospital)
The post-conditioning stimulus will be applied twice daily in the first week. This occurs at pre-specified times: 08.00 PM and AM. However, RIC/sham-RIC can be applied no sooner than 10 hours and not later than 21 hours after the prehospital conditioning stimulus (last cuff).
Aarhus University Hospital: RIC/sham-RIC treatment will continue for a total of 7 days. If patients are discharged to their home or to another department, they will continue treatment according to written instructions, including application of the device for 7 days. After RIC/sham-RIC on day 7, the device is then returned to the Stroke Center.

7.5 Treatment adherence

Assessment of treatment adherence include:

Defining acceptable treatment adherence, the following definition was used:
At least 80% of the overall assigned RIC/sham cycles that were received was required for acceptable adherence to treatment.
For the practical application of the intervention 80% was accepted in each treatment period. Values below this level prompted questions on reasons for the reduced adherence to treatment.

**Acute RIC**		**+6 hours**		**+7days RIC** (Only Aarhus University Hospital)	
**Planned**	**Accepted**	**Planned**	**Accepted**	**Planned**	**Accepted**

5 cycles (5/5min) 100%	4 Cycles (5/5min) 80%	5 cycles (5/5min) 100%	4 Cycles (5/5min) 80%	100%	80%

Treatment adherence data:
Each device counts the number of complete cycles (5min cuff inflation and 5 minutes of cuff deflation) completed. Each cycle starts with cuff inflation. If the device is stopped prematurely (eg. After 5 minutes of cuff inflation) the current cycle will not be recorded.
If a device is not returned back to the stroke center: Adherence is 0%

Adherence is calculated as:

$$Adherence\ (\%) = \frac{\text{Actual cycles (no.)}}{\text{Planned cycles (no.)}} x\ 100\%$$

8 Efficacy Analyses

Primary study endpoint:

Odds ratio for shift in clinical outcome (mRS) at 3 months in acute stroke *(Ordinal logistic regression analysis. Significance level of 5%)*

The **main analysis of the primary study endpoint** will be performed using the entire range of the modified Rankin Scale **(Ordinal logistic regression with a random effect on randomization blocks, otherwise unadjusted)** on the population fulfilling the ***in-hospital inclusion criteria (target population)***. The trial success analysis is performed on the **target population** which consists of prehospital randomized patients with an in-hospital diagnosis of acute ischemic stroke (including TIA with a DWI lesion) and intracerebral hemorrhage (ICH) regardless of adherence to investigational treatment (**modified Intention-To-Treat analysis**). *Treatment groups will be tested at the 2-sided 5% significance level.*

Supplementary safety analysis:
All patients randomized in RESIST will before randomization have a focal neurological deficit (documented on the prehospital stroke score). The presence or absence of specific focal neurological symptoms are assessed again after 24 hours using the same score as pre-randomization (PreSS) in all randomized patients (PreSS 24 hour/ at discharge). The primary safety analysis will be made on the **entire randomized population using the odds ratio for shift in prehospital stroke score** (PreSS) to document change in neurological deficits between pre-randomization and 24 hour/discharge **(Ordinal logistic regression with a random effect on randomization blocks, otherwise unadjusted)**. There has been no safety concern over RIC treatment in patients with non-vascular diagnosis[4,5,6,7,8]

Primary endpoint

Acute Stroke	Entire range of 3 months modified Rankin Scale
**Acute Stroke**<br>*AIS (incl TIA+DWI) and ICH*<br>*Modified Intention-To-Treat*	Entire range of 3 months modified Rankin Scale<br>General ordinal logistic regression, unadjusted

Supplementary safety analysis

All prehospital randomized patients	Difference in PreSS
All prehospital randomized patients (AIS, TIA+DWI, ICH, non-stroke)	Difference in ***PreSS***<br>($PreSS_{prehospital}$ to $PreSS_{24h/discharge}$)<br>General ordinal logistic regression, unadjusted

If remote ischemic preconditioning is associated with ultra-early treatment effect and reduced symptom severity (in the ambulances), this could affect the in-hospital indication for IV-tPA and/or EVT (in the case of rapid improvement of symptoms or mild symptoms)
Because of this, a **supplementary analyses** using **ordinal logistic regression, adjusted for a possible** imbalances in reperfusion treatments (IV-tPA and EVT) between treatment arms will be made.

All analyses using ordinal logistic regression are supplemented a stacked bar chart (Grotta bar).

8.1 Secondary Efficacy Analyses

- Odds ratio for shift in prehospital stroke score (PreSS) (Supplementary safety endpoint, see above) (All randomized patients, ordinal logistic regression, significans level 5%)
- Odds ratio for shift in prehospital stroke score (PreSS) during the first 24 hours: (ordinal logistic regression analysis in the stroke subtypes, Significance level of 5%)
- Relative and absolute difference in proportion of patients with complete remission of symptoms within 24 hours (TIA; both with and without DWI) (All randomized patients, Binomial regression analysis with identity- and log-link function. Significance level of 5%)
- Odds ratio for shift in early neurological improvement in AIS and ICH *(ordinal logistic regression analysis. Significance level of 5%)*

-Very early neurological improvement is defined as:
Reduction in prehospital stroke score (PreSS) ≥ 1 points or resolution of symptoms at admission: Items on PreSS related to NIHSS baseline $\Delta preSS = PreSS_{prehospital}$ – same items on $NIHSS_{baseline}$

-Early neurological improvement is defined as:

Reduction in NIHSS ≥ 4 (baseline versus 24-Hour NIHSS): $\Delta NIHSS = NIHSS_{baseline} - NIHSS_{24}$

-Reduction in prehospital stroke score (PreSS) ≥ 1 points or resolution of symptoms after 24 hours in subgroups: IV tPA/EVT treated AIS and ICH.
Reduction in NIHSS ≥ 4 (baseline versus 24-Hour NIHSS): $\Delta NIHSS = NIHSS_{baseline} - NIHSS_{24}$

-Improvement in median percent change in 24-hour NIHSS:
Median percentage change in NIHSS = $[(NIHSS_{baseline} - NIHSS_{24}) / NIHSS_{baseline}] x100$

- Odds ratio for shift in prehospital stroke score (PreSS) after 24 hours in subgroups: IV tPA/EVT treated AIS and ICH (ordinal logistic regression analysis, significance level of 5%).
- Relative and absolute difference in acute hematoma expansion in patients with ICH (Binomial regression analysis with identity- and log-link function, significance level of 5%)

  - Acute hematoma expansion in patients with ICH
  Hematoma expansion is defined as the difference in volume between the baseline and the 24-hour CT hematoma volume. Significant hematoma growth is defined as an absolute growth exceeding 6 mL or a relative growth of more than 33% from the initial CT[9]. (Binomial regression analysis with identity- and log-link function, significance level of 5%)

  -Difference in 7 days hematoma reabsorption rate in patients with ICH
  Hematoma reabsorption rate is defined as the difference in hematoma volume between the baseline and the 7-day CT hematoma volume. (Linear regression analysis with robust variance estimation, significance level of 5%)

- Difference in acute infarct growth in patients with AIS treated with IV-tPA and/or EVT (Linear regression analysis with robust variance estimation, significance level of 5%)

  -Acute infarct growth is defined as the difference in infarct volume between baseline and 24 hours on DWI-MRI. All patients with IV-tPA and/or EVT treated ischemic stroke and a baseline MRI will have an additional 24-hour MRI performed.

- Hazard rate ratio (HRR) of major adverse cardiac and cerebral events (MACCE) and recurrent ischemic events at 3 and 12 months in AIS patients *(Cox regression analysis, significance level of 5%)*
  -Definition see 6.3 Coding system for MACCE and Recurrent ischemic events:

- Relative and absolute three-month and one-year mortality (Binomial regression analysis with identity- and log-link function, significance level of 5%)
  -Definition see 6.3 Coding system for MACCE and Recurrent ischemic events:

- EVT-eligibility (MRI assessed) in RIC treated AIS patients with large vessel occlusion (LVO)
  - Relative and absolute difference in proportion of RIC treated AIS patients with LVO eligible to EVT treatment compared to standard treatment, adjusted for prehospital stroke severity (PreSS) and symptom duration (Binomial regression analysis with identity- and log-link function, significance level of 5%).

-Difference in MRI-DWI lesion volume in RIC/sham-RIC treated LVO AIS patients eligible to EVT treatment (Linear regression analysis with robust variance estimation, significance level of 5%).

8.1.1 Secondary Analyses of Primary Efficacy Endpoint

- A subgroup analysis will be performed on:
- Acute ischemic stroke patients including TIA with DWI-MRI lesion
- Acute ischemic stroke patients treated with intravenous thrombolysis (IVT)
- Acute ischemic stroke patients treated with thrombectomy (EVT)
- Acute ischemic stroke patients treated with IVT and/or EVT
- AIS patients not treated with reperfusion therapy (IVT and/or EVT)
- Intracerebral hemorrhage patients

AND the following characteristics will be included in subgroup analysis and imputation model:

- Age <65 and >=65years (categorical, yes/no)
- NIHSS ≤5 and NIHSS >5 (categorical, yes/no)
- Time from stroke onset to randomization (≤ 60 minutes vs >60 minutes)
- Hypertension (categorical, yes/no)
- Female (categorical, yes/no)
- Hypertension (categorical, yes/no)
- Diabetes (categorical, yes/no)
- Atrial fibrillation (categorical, yes/no)
- Discharge diagnosis
  - AIS (categorical, yes/no)
  - TIA (categorical, yes/no)
  - ICH (categorical, yes/no)
- Stroke etiology (categorical)
  - Large artery disease (categorical, yes/no)
  - Small vessel disease (categorical, yes/no)
  - Cardioembolic (categorical, yes/no)
  - Other/rare/unknown (categorical, yes/no)
- Center (AUH, OUH, Gødstrup, AaUH) (categorical, 4 categories)
- Adherence (<80 vs >=80 years) (categorical, yes/no)
- Door to groin puncture, minutes (continuous)
- Deep hematoma localization (categorical, yes/no)

Results will be presented in a forest plot with 95% confidence intervals (CI).

9 Safety Analyses

All adverse events including death will be summarized tables for the target population and the safety population group.

Tables providing an overview on:

One Table with events during the study:

- Serious Adverse Events: A table of all serious adverse events SAE and SADE (Serious Adverse Device Effect) or ASADE (Unanticipated Serious Adverse Device Effect (USADE), (See Adverse Events definition below) and whether it was related (related, probable, possible) or unrelated (unlikely, not related, undetermined, not relevant)
- Adverse Events (all events not included above) and whether it was related (related, probable, possible) or unrelated (unlikely, not related, undetermined, not relevant)
- Tables will grouped by type of event and/or organ system, with number and frequency of such events in each arm/group of the clinical study

One Table with death and cardiovascular events during the study:

Mortality and vascular events (MACCE) and recurrent ischemic events as classified by CEC

- Mortality (all cause) and cardiovascular and non-cardiovascular mortality within the two groups of the clinical study, grouped by event type, with number and frequency of such events in each arm/group of the clinical study.
- Vascular events (MACCE) and recurrent ischemic events Other (Not Including Serious) within the two groups of the clinical study, grouped by event type, with number and frequency of such events in each arm/group of the clinical study.
- Its relation to study intervention will be presented "related" (related, probable, possible) or "unrelated" (unlikely, not related, undetermined, not relevant)

10 References

1. Hastrup S, Damgaard D, Johnsen SP, Andersen G. Prehospital Acute Stroke Severity Scale to Predict Large Artery Occlusion: Design and Comparison With Other Scales. *Stroke*. 2016;47(7):1772-1776. doi:10.1161/STROKEAHA.115.012482
2. Kothari RU, Pancioli A, Liu T, Brott T, Broderick J. Cincinnati Prehospital Stroke Scale: reproducibility and validity. *Ann Emerg Med*. 1999;33(4):373-378.
3. Gude MF, Blauenfeldt RA, Behrndtz AB, et al. The Prehospital Stroke Score and telephone conference: A prospective validation. *Acta Neurol Scand*. 2022;145(5):541-550. doi:10.1111/ANE.13580
4. Hougaard KD, Hjort N, Zeidler D, et al. Remote ischemic perconditioning as an adjunct therapy to thrombolysis in patients with acute ischemic stroke: A randomized trial. *Stroke*. 2014;45(1):159-167. doi:10.1161/STROKEAHA.113.001346
5. Le Page S, Bejan-Angoulvant T, Angoulvant D, Prunier F. Remote ischemic conditioning and cardioprotection: a systematic review and meta-analysis of randomized clinical trials. *Basic Res Cardiol*. 2015;110(2):11. doi:10.1007/s00395-015-0467-8
6. Bøtker HE, Kharbanda R, Schmidt MR, et al. Remote ischaemic conditioning before hospital admission, as a complement to angioplasty, and effect on myocardial salvage in patients with acute myocardial infarction: a randomised trial. *Lancet*. 2010;375(9716):727-734. doi:10.1016/S0140-6736(09)62001-8
7. Hausenloy DJ, Candilio L, Evans R, et al. Remote Ischemic Preconditioning and Outcomes of Cardiac Surgery. *N Engl J Med*. 2015;373(15):1408-1417. doi:10.1056/NEJMoa1413534
8. Laiwalla AN, Ooi YC, Liou R, Gonzalez NR. Matched Cohort Analysis of the Effects of Limb Remote Ischemic Conditioning in Patients with Aneurysmal Subarachnoid Hemorrhage. *Transl Stroke Res*. 2016;7(1):42-48. doi:10.1007/s12975-015-0437-3
9. Demchuk AM, Dowlatshahi D, Rodriguez-Luna D, et al. Prediction of haematoma growth and outcome in patients with intracerebral haemorrhage using the CT-angiography spot sign (PREDICT): a prospective observational study. *Lancet Neurol*. 2012;11(4):307-314.

doi:10.1016/S1474-4422(12)70038-8

11 Listing of Tables, Listings and Figures

Disposition / study flowchart is planned for table of Figure 1.xxx
Primary and secondary efficacy and safety endpoints – Table2 or Figure 2 (horizontal stacked bar chart – "grotta bars")
Safety is planned as Table or Figure 3
Subgroup analysis is planned as Table 4 or Figure 4

The following supplemental tables/figures are planned
Primary Endpoint Analysis without Imputation of Missing Data
Primary Endpoint – Sensitivity Analyses
Safety Analysis – Serious Adverse Events

Supplementary Tables
Table S1: Baseline characteristics of Intention to Treat Population
Table S2: Primary and Secondary Efficacy Outcomes – Per-Protocol Population
Table S3: Additional Efficacy and Safety Outcomes – modified Intention to Treat Population
Table S4: Device treatment adherence
Table S5: Death and Serious Adverse Events
Table S6: Category of diagnosis in the stroke mimics group
Supplementary Figures
Figure S1. Additional Subgroup Analysis.
Figure S2. Subgroup Analysis – Pre-Specified Subgroups (Forestplot)
Figure S3. Investigational device photo.

RESIST - database lock

TRIAL FULL TITLE	Remote ischemic conditioning in patients with acute stroke: a multicenter randomized, patient-assessor blinded, sham-controlled study.
TRIAL STATISTICIAN	Jan Brink Valentin
Protocol Version	*version 8.2 (31March2020)*
SAP version	*Version 1 (27March2023)*
TRIAL PRINCIPAL INVESTIGATOR	Grethe Andersen
AUTHOR(s)	Rolf A. Blauenfeldt
Version	*v1.0 21March2023*

1 Database lock Signatures

I give my approval for the lock of the RESIST database.

Study coordinator & Investigator

Name: Rolf A. Blauenfeldt

Signature: REDACTED

Date: March 28, 2023

Principal Investigator

Name: Grethe Andersen

Signature: REDACTED

Date: 28. marts 2023

Trial Statistician

Name: Jan Brink Valentin

Signature: REDACTED

Date: 2023-03-27

2. Summary of changes to the statistical analysis plan

From Nov 29, 2018 to March 20, 2023

**Changes**	**Date of change**	**Document**
Change of primary endpoint: From difference in Prehospital Stroke Scale (PreSS) score during 24 hour **to difference in 90-day functional outcome (modified Rankin Scale)**	**Nov 29, 2018**	Please see summary of Protocol changes
Secondary endpoint of 7 day hematoma reabsorption rate in ICH was added	**Nov 29, 2018**	Please see summary of Protocol changes
Planned demographics and patient characteristics to include in Table 1	**March 20, 2023**	SAP page 14-15
Detailed description on baseline demographics and patient characteristics to include in the planned subgroup analysis based	**March 20, 2023**	SAP page 21
Description on how adverse events will be presented	**March 20, 2023**	SAP page 21 and 22
Change in planned imputation method for missing data: Change from predictive mean matching to chained equations	**March 20, 2023**	SAP page 12
Overview of planned tables and figures	**March 20, 2023**	SAP page 23

3. Original statistical analysis plan (SAP)

The original statistical analysis plan was included in the protocol (clinical investigation plan February 9, 2018).

The following section contains these elements from the protocol

Statistics

Primary study endpoint:

- Difference in prehospital stroke score (PreSS) during the first 24 hours:

  ΔPreSS = $\text{PreSS}_{\text{prehospital}}$ – $\text{PreSS}_{\text{24-hours}}$ ***(Ordinal logistic regression analysis in the per-protocol population. Significance level of 5%)***

  The **<u>main analysis of the primary study endpoint</u>** will be performed using the entire range of the difference in PreSS score **<u>(ordinal logistic regression, unadjusted)</u>** on the ***<u>per-protocol population</u>.*** The per-protocol cohort consists of prehospital randomized patients with an in-hospital diagnosis of acute ischemic stroke with complete investigational treatment and endpoint assessment as defined in the clinical investigational plan.

  A supplementary analysis will be made on the **Intention-to-treat (ITT) population**. The ITT cohort includes all randomized subjects. In non-stroke diagnosis and TIA patients without MRI-DWI lesion, who are discharged before 24-hours, the stroke severity will be documented as *PreSS at discharge (performed by the on call neurologist)*
  Furthermore, a supplementary analyses using multivariable regression will be done in order to account for any imbalances in prognostic factors including the distribution of patients receiving reperfusion therapy between the two treatment arms

- Difference in acute hematoma expansion in patients with ICH (safety outcome) (Binomial regression analysis. Intention-to-treat analysis (all randomized patients with an intracerebral hemorrhage, ICH) Significance level of 5%)

Secondary study endpoints (selected):

- Difference in clinical outcome (mRS) at 3 months in acute ischemic stroke *(ordinal logistic regression analysis. Significance level of 5%)*
- Difference in Early neurological improvement in AIS and ICH *(ordinal logistic regression analysis. Significance level of 5%)*
- Difference in prehospital stroke score (PreSS) after 24 hours in subgroups: IV tPA/EVT treated AIS and ICH. (ordinal logistic regression analysis, significance level of 5%)
- Major adverse cardiac and cerebral events (MACCE) and recurrent ischemic events at 3 and 12 months in AIS patients *(Cox regression analysis, significance level of 5%)*

- Three-month and one-year mortality *(Two sample test of proportion (Chi-square test), significance level of 5%)*
- EVT-eligibility (MRI assessed) in RIC treated AIS patients with large vessel occlusion (LVO) *(Binomial regression analysis. Significance level of 5%)*

Sample Size

The sample size calculation is based on data from IV tPA-treated AIS patients from our institution using an estimated baseline and 24-hour PreSS (from NIHSS baseline and 24 hour). The proportion of AIS patients with a significant reduction of neurological impairment during 24 hours (PreSS score reduction of ≥ 1 point) was 0.43. To detect a 7% absolute increase in neurological improvement in the RIC-treated AIS group at a power of 80% and a significance level of 5%, a sample size of at least 1,380 will be required. To account for non-stroke diagnosis (estimated 24%), ICH (estimated 8%), TIA (estimated 8%), withdrawal and loss to follow-up (estimated 4%)[8], we therefore plan to include 2,500 patients with a prehospital putative stroke. Lost to follow-up rate is estimated to be less 5%. There is no planned replacement of patients lost to follow-up.

The primary endpoint analysis will be performed on the ***per-protocol population***.

Interim analysis

We will perform an interim analysis for evaluation of the actual event rate after inclusion of 50% of the patients. If this shows a lower good outcome rate than expected, a new sample size calculation will be performed. Furthermore, the Data monitoring committee (DMC) will perform an independent safety analysis and will review the overall safety and efficacy according to the DMC charter (planned in the early phase of the trial, at 12 month and hereafter at least every 18. months) An additional safety interim analysis of mortality and hematoma expansion in ICH patients will be performed. Interim analysis are assessed using a level of significance of 1%.

The trial can be stopped at interim analyses for both futility, efficacy, and safety reasons.

Detailed description is found in the DMC charter.

Missing data:

Missing data with be handled using multiple imputation, in which missing cases are first filled in by several sets of plausible values to create multiple completed datasets and then the multiple sets of results are combined to yield a single inference.

Deviations from the statistical plan:

All deviations from the statistical plan will first be applied when approved by the regional ethics committee and the Danish Medicines Agency.

Trial decision making and stopping rules

A Data Monitoring Committee (DMC) will be appointed before trial start and include a signed DMC charter. The DMC will review the accumulated data during RESIST trial and provide advice on the conduct of the trial to the Steering Committee.

The DMC should inform the Steering Committee
if, in their view:

1. the results are likely to provide convincing evidence that one trial arm is clearly indicated or contraindicated, and there is a reasonable expectation that this new evidence would materially influence patient management

or

2. it is beyond doubt that no clear outcome would be obtained (futility)

It is not expected that RESIST trial will be stopped for reasons of efficacy or futility based on observed treatment differences. A predefined pooling of individual patient data (Remote Ischemic conditioning in Stroke Collaboration, RISC) is planned. To dispel scepticism, a robust demonstration of efficacy of this simple intervention is required. The primary reason to recommend stopping the trial will be for safety reasons.

For Acute Ischemic Stroke and Intracerebral Hemorrhage:

- For the primary endpoint the DMC will consider stopping if there is a robust reduction in prehospital stroke score (PreSS), for Acute Ischemic Stroke and Intracerebral Hemorrhage patients, during the first 24-hours in the sham (control) group compared to the intervention (RIC) group, achieving $p<0.01$.
    - A substantial number of patients experience serious adverse events ($p<0.01$)

- For the primary endpoint the DMC will consider stopping if there is an increase in mortality or stroke recurrence for Acute Ischemic Stroke and Intracerebral Hemorrhage patients in the intervention (RIC) group compared to the control (sham) group, achieving $p<0.01$.

- For the primary safety endpoint in ICH the DMC will consider stopping/modification if there is a clear evidence hematoma growth during the first 24-hour in the intervention group, achieving $p<0.01$

The DMC will make recommendations, which could include:

- No action needed, trial continues as planned
- Early stopping due, for example, to clear benefit or harm of a treatment, futility, or external evidence
- Stopping recruitment within a subgroup
- Extending recruitment (based on actual control arm response rates being different to predicted other than on emerging differences) or extending follow-up
- Stopping a single arm of a multi-arm trial
- Approving and/or proposing protocol changes
