# Supplementary material for: Outcomes Following Adherence to a Randomized Stroke Trial Protocol
Source: JAMA Netw Open. 2024 Jan 2;7(1):e2349730. doi: 10.1001/jamanetworkopen.2023.49730 (PMC10762582; doi:10.1001/jamanetworkopen.2023.49730)
Supplement: Supplement 2. — Data Sharing Statement [file jamanetwopen-e2349730-s002.pdf]

# Data Sharing Statement

Blauenfeldt. Outcomes Following Adherence to a Randomized Stroke Trial Protocol. *JAMA Netw Open*. Published January 02, 2024. doi:10.1001/jamanetworkopen.2023.49730

**Data**
**Data available:** Yes
**Data types:** Participant data with identifiers
**How to access data:** Individual participant data that underlie results in this article will be shared after deidentification. Proposals should be directed at rolfblau@rm.dk. To gain access, data requestors will need to sign a data processing agreement.
**When available:** With publication

**Supporting Documents**
**Document types:** None

**Additional Information**
**Who can access the data:** Individual participant data that underlie results in this article will be shared after deidentification. Proposals should be directed at rolfblau@rm.dk. To gain access, data requestors will need to sign a data processing agreement.
**Types of analyses:** Relevant requests
**Mechanisms of data availability:** To gain access, data requestors will need to sign a data processing agreement.
